# Treatment interventions to maintain abstinence from alcohol in primary care: systematic review and network meta-analysis

**DOI:** 10.1136/bmj.m3934

**Published:** 2020-11-25

**Authors:** Hung-Yuan Cheng, Luke A McGuinness, Roy G Elbers, Georgina J MacArthur, Abigail Taylor, Alexandra McAleenan, Sarah Dawson, José A López-López, Julian P T Higgins, Sean Cowlishaw, Anne Lingford-Hughes, Matthew Hickman, David Kessler

**Affiliations:** 1 Population Health Sciences, Bristol Medical School, University of Bristol, Bristol, UK; 2 Department of Basic Psychology and Methodology, University of Murcia, Spain; 3 National Institute for Health Research Health Protection Research Unit (HPRU) in Behavioural Science and Evaluation, University of Bristol, Bristol, UK; 4 National Institute for Health Research Bristol Biomedical Research Centre, University Hospitals Bristol NHS Foundation Trust and University of Bristol, Bristol, UK; 5 Department of Psychiatry, University of Melbourne, Melbourne, Victoria, Australia; 6 Faculty of Medicine, Department of Brain Sciences, Imperial College London, London, UK; 7 National Institute for Health Research School for Primary Care Research, University of Bristol, Bristol, UK

## Abstract

**Objective:**

To determine the most effective interventions in recently detoxified, alcohol dependent patients for implementation in primary care.

**Design:**

Systematic review and network meta-analysis.

**Data sources:**

Medline, Embase, PsycINFO, Cochrane CENTRAL, ClinicalTrials.gov, and the World Health Organization’s International Clinical Trials Registry Platform.

**Study selection:**

Randomised controlled trials comparing two or more interventions that could be used in primary care. The population was patients with alcohol dependency diagnosed by standardised clinical tools and who became detoxified within four weeks.

**Data extraction:**

Outcomes of interest were continuous abstinence from alcohol (effectiveness) and all cause dropouts (as a proxy for acceptability) at least 12 weeks after start of intervention.

**Results:**

64 trials (43 interventions) were included. The median probability of abstinence across placebo arms was 25%. Compared with placebo, the only intervention associated with increased probability of abstinence and moderate certainty evidence was acamprosate (odds ratio 1.86, 95% confidence interval 1.49 to 2.33, corresponding to an absolute probability of 38%). Of the 62 included trials that reported all cause dropouts, interventions associated with a reduced number of dropouts compared with placebo (probability 50%) and moderate certainty of evidence were acamprosate (0.73, 0.62 to 0.86; 42%), naltrexone (0.70, 0.50 to 0.98; 41%), and acamprosate-naltrexone (0.30, 0.13 to 0.67; 17%). Acamprosate was the only intervention associated with moderate confidence in the evidence of effectiveness and acceptability up to 12 months. It is uncertain whether other interventions can help maintain abstinence and reduce dropouts because of low confidence in the evidence.

**Conclusions:**

Evidence is lacking for benefit from interventions that could be implemented in primary care settings for alcohol abstinence, other than for acamprosate. More evidence from high quality randomised controlled trials is needed, as are strategies using combined interventions (combinations of drug interventions or drug and psychosocial interventions) to improve treatment of alcohol dependency in primary care.

**Systematic review registration:**

PROSPERO CRD42016049779.

## Introduction

In the United Kingdom, the morbidity and mortality burden from alcohol consumption remains high, with 7% of hospital admissions related to alcohol.^1^ Liver disease is the third most common cause of premature death in the UK and the only major cause of death that is on the increase, with about two thirds of such deaths related to alcohol.^2^ Alcohol related harm is estimated to cost the UK National Health Service £3.5bn ($4.5bn; €3.9bn) annually, with the total annual cost to the UK estimated at £21bn.^1^ ^3^

In England, around more than half a million adults are alcohol dependent and need assessment and treatment.^4^ However, only one in four of those with alcohol dependence seek treatment, and relapse is common.^5^ The National Institute for Health and Care Excellence guideline^6^ recommends a comprehensive assessment, followed by either community based or residential medically assisted alcohol withdrawal, and that in particular drug treatments for abstinence should be considered by specialist staff for patients with moderate to severe alcohol dependence. The capacity to offer this level of support in specialist services for the number of patients who need such care is, however, limited; about 82% of people do not receive the specialist treatment needed.^7^ Switching the management of alcohol dependence to within primary care has the potential to improve access to treatment. Here we consider primary care to be a setting where medical services were provided in general practice, the first point of contact for patients, and not by specialist services.^8^ To achieve better long term outcomes, the maintenance of abstinence needs to be followed by medium to long term support. Although such support is currently managed by specialist care, primary care stands in a unique position to provide holistic care.

Evidence is accumulating for several interventions to maintain abstinence that feasibly could be offered in primary care, or at the level of a close collaboration between primary and specialist care.^9^ In this systematic review and network meta-analysis we aimed to determine the most effective interventions for alcohol abstinence with the potential to be delivered in a primary care setting.

## Methods

The protocol for this review has been published previously^10^ and registered in the International Prospective Register of Systematic Reviews (PROSPERO 2016: CRD42016049779). This report complies with the PRISMA (preferred reporting items for systematic reviews and meta-analyses) network meta-analysis extension statement (see supplement 1 for checklist).^11^

### Eligibility criteria

We sought randomised controlled trials that investigated any treatment intervention (drug, psychological, or both) for maintaining abstinence in recently detoxified, alcohol dependent adults. We were interested only in interventions appropriate for primary care settings and only drugs that are available in the UK. Studies were eligible if the participants were older than 18 years with alcohol dependency diagnosed using standardised diagnostic criteria (eg, Diagnostic and Statistical Manual of Mental Disorders, International Classification of Diseases) or the Alcohol Use Disorders Identification Test (AUDIT; score ≥20). To reflect current clinical practice, we sought only studies that provided detoxification to participants, as well as studies that recruited participants who had undergone detoxification less than four weeks before randomisation. To be eligible, studies had to have follow-up periods longer than 12 weeks.

### Outcomes

Our primary effectiveness outcome was continuous abstinence as reported by the trial authors. We chose abstinence over reduced risk drinking as the most appropriate outcome for this population of alcohol dependent patients because abstinence was the preferred goal among patients in a recent large UK trial^12^ and NICE guideline^6^ and because abstainers have a lower rate of relapse over the longer term.^13^ ^14^ For secondary outcomes, we sought data on amount of alcohol consumption, drinking frequency, intervention compliance, adverse events, and withdrawal from the study. These were reported inconsistently and were not amenable to meaningful analysis. Since we were generally interested in acceptability of the interventions, we used the number of dropouts (or number lost to follow-up) for any reason as a proxy for acceptability, as a secondary outcome.

### Search strategy

An information specialist (SD) developed the search strategies and searched four electronic databases: Cochrane Central Register of Controlled Trials (CENTRAL), Ovid Medline, Ovid Embase, and Ovid PsycINFO from inception date to 3 March 2020 (see supplement 2 for search strategies). To supplement the search of the electronic databases we additionally hand searched relevant randomised controlled trials from reference lists of identified systematic reviews, meta-analyses, and studies included in this review (known as snowballing^15^). Two trial registries, ClinicalTrials.gov and WHO International Clinical Trials Registry Platform (ICTRP), were searched from inception date to March 2020 to identify registered trials and relevant reports. No restriction was applied on date, language, or publication status.

### Selection of studies

Search results were managed using Endnote and Microsoft Excel. One author (HC) screened the titles and abstracts of all identified references. A second reviewer (RGE, LAM, SD, AT, or GJM) also independently screened the titles and abstracts identified from the primary source of randomised controlled trials (CENTRAL), comprising more than half of the search results. The main screener missed none of the studies eventually included in the review, indicating a low likelihood that trials were missed in the other sources. The full texts of potentially eligible references were obtained and screened independently in duplicate (by HC and one of RGE, LAM, SD, AT, or GJM). A native speaker assessed or translated studies in non-English languages. Disagreements when screening the title, abstract, and full text were resolved by discussion.

To determine interventions that are applicable to primary care,^8^ three content experts (DK, ALH, and MH) examined the interventions. Interventions that involved frequent, repeated intravenous infusions, uncommon equipment in primary care, illicit drugs, experimental chemicals, and drugs unlicensed in the UK were not included in the review (see list in supplement 3). We excluded studies on pregnant women, participants with chronic liver disease, participants with HIV/AIDS, and patients with liver transplant owing to the specific clinical considerations of these populations.

### Data extraction

Data from each eligible study were extracted in duplicate using pre-piloted, standardised Microsoft Excel spreadsheets. When multiple publications from one study existed, we considered and combined all publications to extract information, so that a single result from each study contributed to each synthesis. In the case of missing data, we contacted authors directly, and when no response was received, we attempted to retrieve information from other systematic reviews and meta-analyses. For example, the number of patients in each group in Pelc et al^16^ was informed using the review by Mann et al.^17^

### Risk of bias assessment

A pre-release version of the RoB 2 tool was used to assess the risk of bias in five domains of the randomised trials^18^: bias arising from the randomisation process, deviations from intended interventions, missing outcome data, measurement of the outcome, and selection of the reported result. Domain level judgments led to an overall risk of bias judgment for each study result. Two reviewers (LAM, RGE, GJM, and AM paired with HC) assessed each study independently. Discrepancies between reviewers were resolved by discussion.

### Quality of evidence evaluation

Confidence in the evidence was evaluated using an adapted version of GRADE (grading recommendations assessment, development and evaluation) methodology^19^ through a web based application, Confidence In Network Meta-Analysis (CINeMA).^20^ Content experts (MH and DK), in conjunction with HC, AM, and JPTH, evaluated the evidence in each intervention comparison based on within study bias, reporting bias, indirectness, imprecision, heterogeneity, and incoherence. Supplement 4 shows the criteria for the GRADE assessment using CINeMA. Risk of reporting bias was informed by considering non-statistical factors (empirical knowledge from searches, screening, and communications with expert and study authors) and using funnel plots (if >10 studies in pairwise comparisons and comparison adjusted funnel plots for network meta-analysis).^21^ ^22^

### Data synthesis and analyses

We grouped different dosages of the same interventions into one node for network meta-analyses. For one study,^23^ we grouped disulfiram (1 mg/day) with the placebo (riboflavin) group because the author used the disulfiram group as a control and indicated no reaction between disulfiram and ethanol at this low dose. We coded citalopram and escitalopram as the same node in the network meta-analysis because these two drugs are clinically interchangeable; and the dosage and regimen used in studies that investigated these drugs were aligned with clinical practice. This allowed us to incorporate one study that would otherwise have been excluded.^24^ A variety of control groups were observed among the included studies. We categorised these control groups into placebo or treatment as usual. Placebo groups followed the conventional definition, suggesting a physical pill without the active ingredient or ingredients. Treatment as usual groups consisted of standard, conventional treatments and 12 step facilitation.

The primary outcome measure was dichotomous, ideally extracted as the number of patients who remained abstinent (no alcohol intake) after randomisation, out of the total number of participants randomised. We converted percentages or fractions to whole numbers based on the number of randomised patients, provided an intention-to-treat analysis had been used. If intention-to-treat results were not available, we used reported results for completers. A similar approach was used for dropouts, defined as the number of patients who withdrew from the study at reported time points.

We found that outcomes were reported over a wide range of time points between three and 24 months. We categorised outcomes (in a slight change from the protocol^10^) into short (3-6 months), medium (6-12 months), and long (12-24 months) term outcomes. If a trial reported results at multiple time points, we extracted the result at the longest time point within these periods for the main analysis. To enable all studies to be included, in the main analysis we combined results reported at the nearest time point to 12 months from each study.

We conducted pairwise and network meta-analyses for effectiveness (abstinence) and acceptability (dropouts). We reported estimated odd ratios with 95% confidence intervals comparing each intervention with placebo or with treatment as usual depending on the network structure. Heterogeneity was assessed using the results of the pairwise analyses, and between study variance for the network meta-analyses (τ^2^). To compute absolute probabilities of each outcome under each treatment, we took the median probability on placebo across all trials in the main analysis (rounded to the nearest multiple of 5) and applied the odds ratio for each treatment to this probability.^25^ For evaluations of consistency in the network, we used the design-by-treatment interaction model^26^ (global assessment) and node split^27^ (local assessment) methods. We used the mean of the distribution of ranks for each intervention to present its relative order of preference based on the network meta-analysis.

All analyses were performed in STATA MP15, assuming random effects for intervention effects and fixed effects for study baselines within a frequentist framework.^22^ ^28^ Networks were plotted using Gephi (version 0.9.2).^29^ The dataset used for computing the analyses is available in the *data.bris* repository.

### Additional analyses

Separate network meta-analyses by intervention types (psychosocial interventions, drug, or combined drug interventions) were conducted to check the robustness of results to the possibility that treatment effects were not transitive across different approaches of studies to intervention. We also conducted analyses separately for outcomes measured at short, medium, and long term time points. We had planned sensitivity analyses that excluded studies with overall high risk of bias but did not do these owing to sparsity of data.

We were unable to conduct several preplanned subgroup and meta-regression analyses (length of intervention, optional psychosocial interventions, dosing and schedule of interventions, psychiatric comorbidity, severity of alcohol dependence, and social background) because of inconsistent or poorly reported data on these characteristics across studies. Several study level characteristics were investigated to explore heterogeneity of intervention effects across intervention comparisons: percentage of female populations, mean age, methods of detoxification (medically assisted detoxification or unclear), settings of detoxification (inpatient, outpatient, mixed, or unclear), and continent of study sites (five continents).

### Patient and public involvement

No patients were involved in setting the research question or the outcome measures, nor were they involved in developing plans for design or implementation of the study. No patients were asked to advise on interpretation or writing up of the results. We do plan to disseminate the results of the research to the relevant patient communities.

## Results

**Fig 1:**
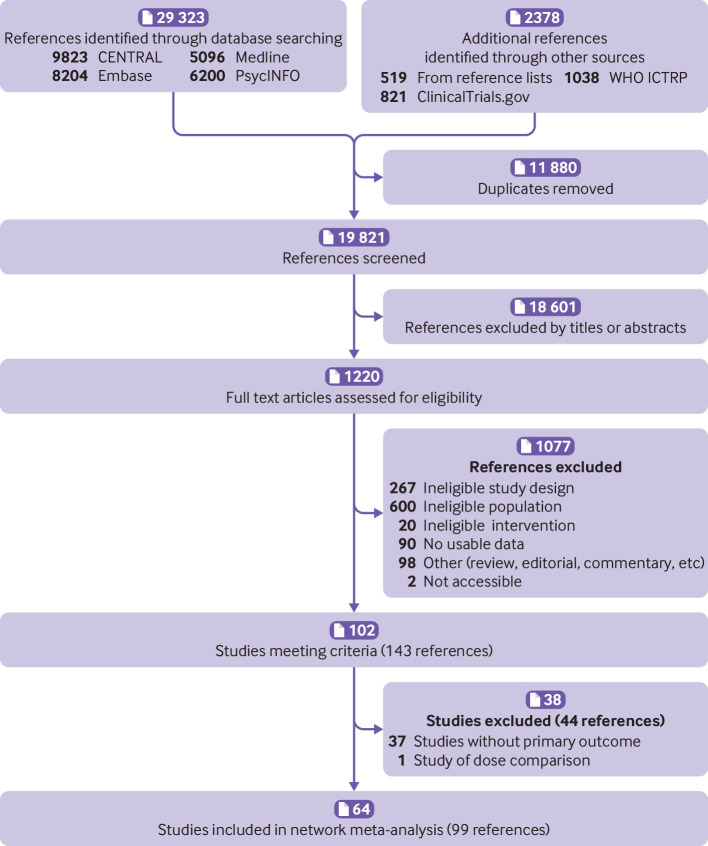
Study selection flowchart

Overall, 29 323 records were identified from electronic databases and 2378 from other sources. After removing 11 880 duplicates, the titles and abstracts of the 19 821 remaining records were screened. Overall, 18 601 records were irrelevant based on the titles and abstracts, thus full texts were sought for the remaining 1220 records. One hundred and two studies (143 references) met the inclusion criteria (fig 1). One study was excluded because the trial compared the effects of oxcarbazepine in two different dosages.^30^ Results for continuous abstinence were available (either from reports or by contacting trial authors) for 64 studies (reported in 99 references), and these were included in the review. Full lists of excluded studies and reasons for exclusion are available in the *data.bris* repository.

### Overview of studies

Supplement 5 shows the characteristics of the 64 included studies. These were published over a period of 35 years (1986-2020). In all but one study, participants started the trial after detoxification; in the remaining study the intervention was started during detoxification.^31^ The methods and settings of detoxification were poorly reported (table 1). Most studies stated only that patients were detoxified, without specifying the details. In studies that did report the methods of medically assisted detoxification, patients were commonly detoxified with benzodiazepines, with a third study reporting inpatient detoxification. Despite variation in inclusion and exclusion criteria, the characteristics of participants were similar across studies: predominantly men, aged around 40, and presenting with mild to moderate mental illness, such as anxiety and depression. Only four studies recruited specific populations: people with depression^32^ ^33^ or sleep disturbance,^34^ elderly people,^35^ and women.^36^ No information was provided on the use of existing drugs or additional drugs in most trials; and if provided, patients were usually not allowed to use or had limited use of additional antidepressants, anticonvulsants, anxiolytics, and hypnotics during the trials.

**Table 1:** General characteristics of studies. Values are numbers (percentages) unless stated otherwise

Characteristics	Abstinence (n=64)	All cause dropouts (n=62)
**Study characteristics**		
Median (range) study sample size	106 (17-774)	115 (17-774)
No of arms:		
2	55 (86)	53 (85)
3	6 (9)	6 (10)
4	3 (5)	3 (5)
Type of controls:		
Placebo	42 (68)	41 (68)
Treatment as usual	9 (14)	9 (15)
No of interventions	43	43
Type of interventions:		
Drug	51 (80)	49 (79)
Psychosocial	6 (9)	6 (10)
Combined	7 (11)	7 (11)
Median (range) follow-up (days)	180 (84-365)	180 (84-365)
Continent:		
North America	12 (19)	12 (19)
Europe	46 (72)	44 (71)
Asia	3 (5)	3 (5)
South America	3 (5)	3 (5)
**Patient characteristics**		
Median (range) age (years); No in group	43.2 (30.6-63.4); n=61	43 (30.6-63.4); n=59
Median (range) women (%); No in group	22 (0-100); n=59	22 (0-100); n=58
Detoxification methods:		
Drug	20 (31)	20 (32)
Unclear	44 (70)	42 (68)
Detoxification settings:		
Inpatient	20 (31)	20 (32)
Outpatient	2 (3)	2 (3)
Mixed	7 (11)	7 (11)
Unclear	35 (55)	33 (53)

Drug interventions were the most studied type of intervention (51/64 studies) (table 1). Six studies investigated seven forms of psychosocial interventions: Addiction‐Comprehensive Health Enhancement Support System,^37^ cognitive behavioural therapy,^38^ ^39^ ^40^ short form cognitive behavioural therapy,^41^ contingency management,^42^ coping skill training,^38^ ^39^ ^40^ home visit,^42^ ^43^ and motivational enhancement therapy.^40^ ^41^ These interventions were applied either on top of an existing treatment programme^37^ ^43^ or alone.^38^ ^39^ ^40^ Two studies specifically investigated combinations of drug and psychosocial interventions: one study compared the effect of combined follow-up by a community nurse and acamprosate with acamprosate alone^44^; another compared the effects of combined treatment with nefazodone and cognitive behavioural therapy.^45^ In most studies, patients received concomitant psychosocial therapy or were encouraged to attend self-help interventions, or both, such as Alcoholics Anonymous and social services. However, details of these concomitant interventions, such as settings, methods, and attendance rates, were poorly reported, therefore further analyses of these factors were not possible.

### Risk of bias within included studies

Supplement 6 shows the results of risk of bias assessments for the outcome abstinence. Overall, 30 and 27 out of 64 studies were judged overall to have “some concerns” or to be at “high risk” of bias, respectively. The main reasons for having some concerns were lack of description in the randomisation process (39/64 studies) and unbalanced missing data between groups (13/64 studies); and the main reason for high risk of bias (20/64 studies) was missing outcome data. Treatment effects of some studies could be contaminated owing to the open label design or the nature of interventions. These contributed to high risk of bias owing to deviations from intended interventions in two trials.

### Effectiveness: maintaining abstinence up to 12 months versus placebo

**Fig 2:**
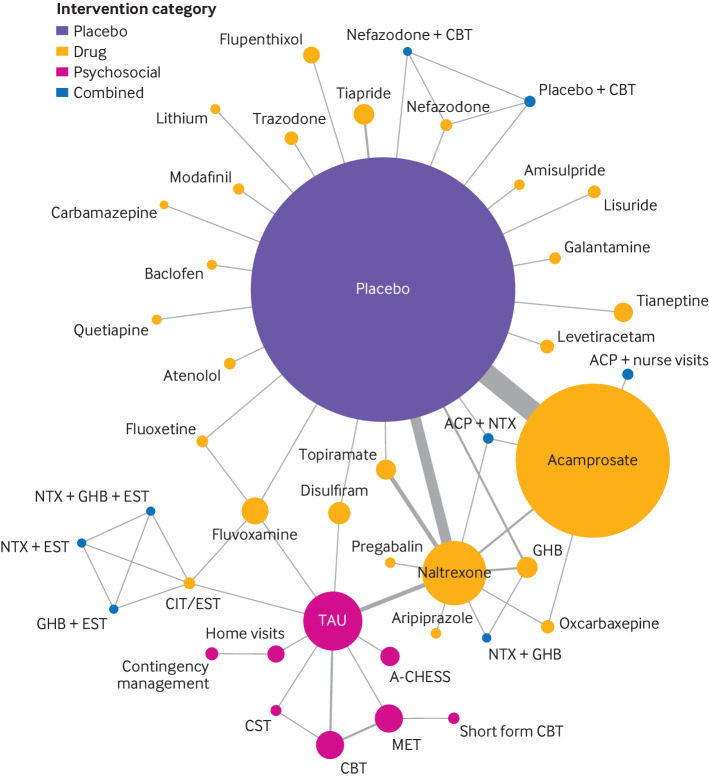
Network plots for alcohol abstinence in relation to treatment for alcohol dependency. Size of circles is proportional to number of randomised patients and width of lines is proportional to number of studies in each direct comparison. A-CHESS=Addiction-Comprehensive Health Enhancement Support System; ACP=acamprosate; CBT=cognitive behavioural therapy; CIT=citalopram; CST=coping skill training; GHB=sodium salt of gamma hydroxybutyric acid (sodium oxybate); MET=motivational enhancement therapy; NTX=naltrexone; TAU=treatment as usual

Sixty four studies (43 interventions) were included in the network meta-analysis of the primary outcome (fig 2). Placebo was the most used control (42/64 studies). Heterogeneity was low or undetectable in most of the separate pairwise comparisons because of the low number of studies involved in each comparison. The estimated between study variance (τ^2^) from the network meta-analysis was 0.084 (see supplement 7). No evidence of inconsistency based on a random effects design-by-treatment interaction model was found (χ^2^=16.19, df=13, P=0.24). Local tests of loop inconsistency did not indicate inconsistency between direct and indirect estimations, except for one comparison between sodium oxybate (also known as sodium salt of gamma hydroxybutyric acid or GHB) and placebo (P=0.042), which is compatible with chance given the large number of comparisons.

Table 2 shows the main results of the analysis, along with assessments of the quality of evidence for each intervention. The median probability of staying abstinent across placebo arms was 25%. Most interventions showed no or insufficient evidence of an effect on maintaining abstinence greater than placebo. Drug interventions had mixed results, with estimated odds ratios ranging from 0.31 (95% confidence interval 0.11 to 0.87) for galantamine (indicating reduced probability of abstinence compared with placebo) to increased odds of abstinence compared with placebo for four interventions: acamprosate (1.86, 1.49 to 2.33), topiramate (1.88, 1.06 to 3.34), sodium oxybate (2.31, 1.22 to 4.36), and quetiapine (6.75, 1.20 to 38.05). Other drugs licensed for treating alcohol dependence, disulfiram (0.93, 0.48 to 1.79) and naltrexone (1.36, 0.97 to 1.91), were not or were weakly associated with improved abstinence. All psychosocial interventions alone were not associated with a greater probability of maintaining abstinence up to 12 months. Four combined interventions were more effective than placebo at maintaining abstinence: acamprosate and nurse visits (4.59, 1.47 to 14.36), acamprosate and naltrexone (3.68, 1.50 to 9.02), sodium oxybate and naltrexone (12.64, 2.77 to 57.78), and naltrexone and sodium oxybate and escitalopram (25.65, 2.13 to 309.46). Treatment as usual (0.52, 0.29 to 0.94), flupenthixol (0.44, 0.20 to 0.95), and galantamine 0.31 (0.11 to 0.87) were associated with reduced odds of relapse compared with placebo. However, confidence in the evidence of the effect of most interventions, apart from acamprosate and tiapride, was low or very low. The reasons for the low to very low confidence were within study bias, imprecision, and heterogeneity, mainly because the evidence for most interventions was generated from single, small studies. 

**Table 2:** Network meta-analysis (NMA) and quality of evidence for abstinence

Intervention (reference: placebo)	No of arms (placebo, n=42)	No of participants (placebo, n=4044)	Odd ratios (95% CI)			Corresponding absolute probability of abstinence (95% CI) (assumed 25% for placebo) (%)	Quality of evidence*
			Direct estimate	Indirect estimate	NMA estimate		
**Psychosocial interventions**							
Treatment as usual	9	800	-	0.52 (0.29 to 0.94)	0.52 (0.29 to 0.94)	15 (9 to 24)	Low†‡§¶
A-CHESS	1	170	-	0.88 (0.35 to 2.21)	0.88 (0.35 to 2.21)	23 (10 to 42)	Very low†‡§¶
CBT	2	306	-	0.53 (0.23 to 1.22)	0.53 (0.23 to 1.22)	15 (7 to 29)	Low†§¶
Short form CBT	1	43	-	0.05 (0.00 to 1.16)	0.05 (0.00 to 1.16)	2 (0 to 28)	Very low†§¶
Contingency management	1	79	-	0.78 (0.17 to 3.61)	0.78 (0.17 to 3.61)	21 (5 to 55)	Low†‡§
Coping skill training	1	40	-	0.35 (0.10 to 1.19)	0.35 (0.10 to 1.19)	10 (3 to 28)	Very low†§¶
Home visit	2	142	-	0.95 (0.32 to 2.85)	0.95 (0.32 to 2.85)	24 (10 to 49)	Low†§¶
MET	2	308	-	0.45 (0.19 to 1.11)	0.45 (0.19 to 1.11)	13 (6 to 27)	Very low†§¶
**Drug interventions**							
Acamprosate	18	2297	1.92 (1.52 to 2.42)	0.74 (0.21 to 2.53)	1.86 (1.49 to 2.33)	38 (33 to 44)	Moderate¶**
Amisulpride	1	37	0.39 (0.09 to 1.64)	-	0.39 (0.09 to 1.64)	12 (3 to 35)	Low†§¶
Aripiprazole	1	29	-	1.49 (0.43 to 5.18)	1.49 (0.43 to 5.18)	33 (12 to 63)	Low†§¶
Atenolol	1	50	0.85 (0.25 to 2.95)	-	0.85 (0.25 to 2.95)	22 (8 to 50)	Very low†‡§¶
Baclofen	1	28	4.63 (1.00 to 21.48)	-	4.63 (1.00 to 21.48)	61 (25 to 88)	Low†§¶
Carbamazepine	1	13	0.55 (0.08 to 3.90)	-	0.55 (0.08 to 3.90)	15 (2 to 57)	Very low†‡§¶
Citalopram/escitalopram	2	45	-	1.03 (0.33 to 3.16)	1.03 (0.33 to 3.16)	25 (10 to 51)	Low†§¶
Disulfiram	2	221	0.97 (0.46 to 2.01)	0.72 (0.13 to 4.05)	0.93 (0.48 to 1.79)	24 (14 to 37)	Low†‡§¶
Fluoxetine	2	50	2.14 (0.48 to 9.52)	4.51 (0.83 to 24.39)	2.97 (0.97 to 9.05)	50 (24 to 75)	Very low†‡§¶
Flupenthixol	1	142	0.44 (0.20 to 0.95)	-	0.44 (0.20 to 0.95)	13 (6 to 24)	Very low†§¶
Fluvoxamine	3	293	0.99 (0.49 to 2.01)	1.14 (0.34 to 3.89)	1.03 (0.57 to 1.88)	26 (16 to 38)	Low†§¶
Galantamine	1	74	0.31 (0.11 to 0.87)	-	0.31 (0.11 to 0.87)	9 (4 to 23)	Low†§¶
GHB	4	201	1.65 (0.85 to 3.24)	7.48 (2.05 to 27.28)	2.31 (1.22 to 4.36)	43 (29 to 59)	Very low†‡¶**
Levetiracetam	1	95	1.03 (0.46 to 2.34)	-	1.03 (0.46 to 2.34)	26 (13 to 44)	Low†§¶
Lisuride	1	57	0.38 (0.13 to 1.12)	-	0.38 (0.13 to 1.12)	11 (4 to 27)	Very low†§¶
Lithium	1	28	1.43 (0.39 to 5.23)	-	1.43 (0.39 to 5.23)	32 (12 to 64)	Low†§¶
Modafinil	1	41	2.48 (0.72 to 8.53)	-	2.48 (0.72 to 8.53)	45 (19 to 74)	Low†§¶
Naltrexone	17	878	1.29 (0.86 to 1.92)	1.59 (0.81 to 3.10)	1.36 (0.97 to 1.91)	31 (24 to 39)	Low†§¶
Nefazodone	1	50	0.57 (0.19 to 1.76)	-	0.57 (0.19 to 1.76)	16 (6 to 37)	Very low†‡§¶
Oxcarbazepine	2	72	-	2.46 (0.91 to 6.61)	2.46 (0.91 to 6.61)	45 (23 to 69)	Very low†§¶
Pregabalin	1	31	-	1.97 (0.58 to 6.74)	1.97 (0.58 to 6.74)	40 (16 to 69)	Low†§¶
Quetiapine	1	29	6.75 (1.20 to 38.05)	-	6.75 (1.20 to 38.05)	69 (29 to 93)	Low†§¶
Tianeptine	1	170	1.22 (0.58 to 2.57)	-	1.22 (0.58 to 2.57)	29 (16 to 46)	Low†§¶
Tiapride	2	187	0.56 (0.30 to 1.05)	-	0.56 (0.30 to 1.05)	16 (9 to 26)	Moderate§¶
Topiramate	3	194	2.26 (0.83 to 6.13)	1.72 (0.84 to 3.52)	1.88 (1.06 to 3.34)	39 (26 to 53)	Very low†‡§¶**
Trazodone	1	88	0.61 (0.20 to 1.84)	-	0.61 (0.20 to 1.84)	17 (6 to 38)	Very low†‡§¶
**Combined interventions**							
Placebo+CBT	1	50	0.83 (0.28 to 2.42)	-	0.83 (0.28 to 2.42)	22 (9 to 45)	Very low†‡§¶
Nefazodone+CBT	1	53	0.77 (0.26 to 2.23)	-	0.77 (0.26 to 2.23)	20 (8 to 43)	Very low†‡§¶
Acamprosate+nurse visit	1	50	-	4.59 (1.47 to 14.36)	4.59 (1.47 to 14.36)	60 (33 to 83)	Very low ^a c d^
Acamprosate+NTX	1	40	5.57 (1.82 to 16.96)	1.63 (0.33 to 7.95)	3.68 (1.50 to 9.02)	55 (33 to 75)	Low†§¶**
GHB+EST	1	12	-	5.13 (0.53 to 49.92)	5.13 (0.53 to 49.92)	63 (15 to 94)	Low†§¶
GHB+NTX	1	18	-	12.64 (2.77 to 57.78)	12.64 (2.77 to 57.78)	81 (48 to 95)	Very low†‡§¶
NTX+EST	1	12	-	2.57 (0.25 to 25.85)	2.57 (0.25 to 25.85)	46 (8 to 90)	Low†§¶
NTX+GHB+EST	1	12	-	25.65 (2.13 to 309.46)	25.65 (2.13 to 309.46)	90 (41 to 99)	Low†§¶

A-CHESS=Addiction‐Comprehensive Health Enhancement Support System; CBT=cognitive behavioural therapy; MET=motivational enhancement therapy; GHB=sodium salt of gamma hydroxybutyric acid (sodium oxybate); EST=escitalopram; NTX=naltrexone.

* See supplement 4 for full details of criteria for downgrading quality of evidence.

† Within study bias.

‡ Indirectness.

§ Imprecision.

¶ Heterogeneity.

** Incoherence.

### Secondary outcome: all cause dropouts up to 12 months versus placebo

**Fig 3:**
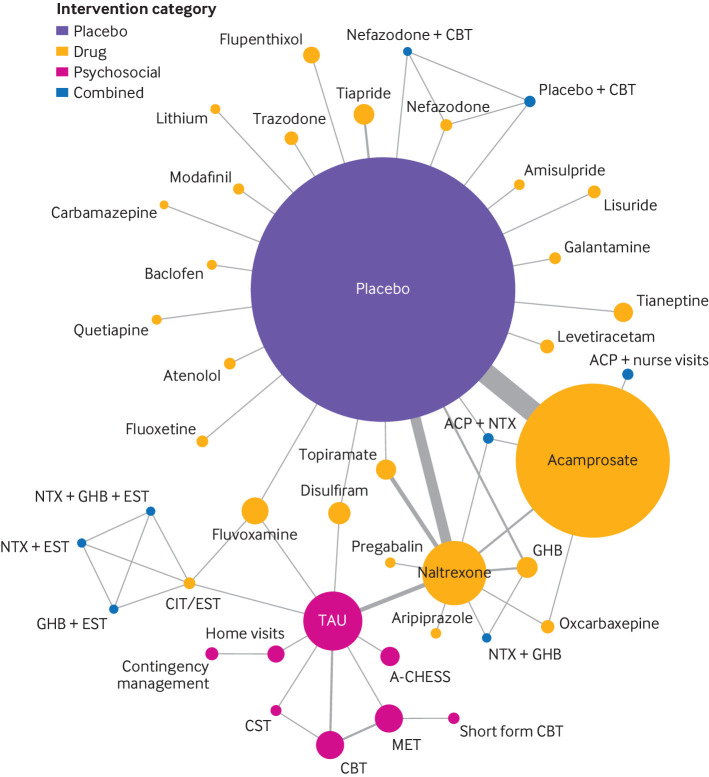
Network plots for all cause dropouts in relation to treatment for alcohol dependency. Size of circles is proportional to number of randomised patients and width of lines is proportional to number of studies in each direct comparison. A-CHESS=Addiction-Comprehensive Health Enhancement Support System; ACP=acamprosate; CBT=cognitive behavioural therapy; CIT=citalopram; CST=coping skill training; GHB=sodium salt of gamma hydroxybutyric acid (sodium oxybate); MET=motivational enhancement therapy; NTX=naltrexone; TAU=treatment as usual

The composition of studies reporting all cause dropouts was similar to that for the primary outcome. Sixty two studies (43 interventions) were included in the network meta-analysis of the secondary outcome (fig 3). Heterogeneity in the comparisons was low or non-detectable owing to low number of studies within the comparisons (see supplement 7). The estimated between study variance (τ^2^) from the network meta-analysis was 0.031. No evidence of inconsistency was found in the random effects design-by-treatment interaction model (χ^2^=12.95, df=12, P=0.37) or when using the node split method.

Table 3 shows the results of the network meta-analysis and confidence in the evidence. The median probability of dropout across placebo arms was 48%, although for interpretational convenience we set it at 50% for computation of corresponding absolute risks for each intervention. Compared with placebo, the relative treatment effects of active interventions on reducing dropouts were similar. Only a few interventions were associated with reduced dropouts compared with placebo: acamprosate (odds ratio 0.73, 95% confidence interval 0.62 to 0.86), naltrexone (0.70, 0.50 to 0.98), topiramate (0.45, 0.24 to 0.83), home visits (0.32, 0.11 to 0.95), short form cognitive behavioural therapy (0.06, 0.01 to 0.33), acamprosate and nurse visits (0.21, 0.07 to 0.57), and acamprosate and naltrexone (0.30, 0.13 to 0.67). Flupenthixol (2.37, 1.27 to 4.40), fluvoxamine (2.15, 1.30 to 3.55), and carbamazepine (12.00, 1.22 to 118.42) were associated with increased odds of dropout compared with placebo. Confidence in the evidence on the effect of interventions was also generally low or very low, with four exceptions as moderate (acamprosate, naltrexone, tiapride, and acamprosate and naltrexone).

**Table 3:** Network meta-analysis and quality of evidence for all cause dropouts

Intervention (reference: placebo)	No of arms (placebo, n=41)	No of participants (placebo, n=4012)	Odd ratio (95% CI)			Corresponding absolute probability of dropout (95% CI) (assumed 50% for placebo) (%)	Quality of evidence
			Direct estimate	Indirect estimate	NMA estimate		
**Psychosocial interventions**							
Treatment as usual	9	800	-	1.14 (0.65 to 1.99)	1.14 (0.65 to 1.99)	53 (39 to 67)	Low†‡§¶
A-CHESS	1	170	-	1.14 (0.50 to 2.60)	1.14 (0.50 to 2.60)	53 (33 to 72)	Very low†‡§¶
CBT	2	306	-	1.16 (0.45 to 3.04)	1.16 (0.45 to 3.04)	54 (31 to 75)	Low†§¶
Short form CBT	1	43	-	0.06 (0.01 to 0.33)	0.06 (0.01 to 0.33)	6 (1 to 25)	Very low†§¶
Contingency management	1	79	-	0.32 (0.02 to 6.55)	0.32 (0.02 to 6.55)	24 (2 to 87)	Low†‡§
Coping skill training	1	40	-	1.98 (0.55 to 7.17)	1.98 (0.55 to 7.17)	66 (35 to 88)	Low†§¶
Home visit	2	142	-	0.32 (0.11 to 0.95)	0.32 (0.11 to 0.95)	24 (10 to 49)	Low†§¶
MET	2	308	-	1.30 (0.46 to 3.64)	1.30 (0.46 to 3.64)	56 (32 to 78)	Low†§¶
**Drug interventions**							
Acamprosate	17	2268	0.71 (0.58 to 0.87)	1.17 (0.31 to 4.34)	0.73 (0.62 to 0.86)	42 (38 to 46)	Moderate§**
Amisulpride	1	37	1.89 (0.66 to 5.43)	-	1.89 (0.66 to 5.43)	65 (40 to 84)	Low†§¶
Aripiprazole	1	29	-	0.67 (0.18 to 2.45)	0.67 (0.18 to 2.45)	40 (15 to 71)	Low†§¶
Atenolol	1	50	1.09 (0.46 to 2.57)	-	1.09 (0.46 to 2.57)	52 (31 to 72)	Low†‡§¶
Baclofen	1	28	0.87 (0.29 to 2.62)	-	0.87 (0.29 to 2.62)	46 (22 to 72)	Low†§¶
Carbamazepine	1	13	12.00 (1.22 to 118.42)	-	12.00 (1.22 to 118.42)	92 (55 to 99)	Very low†‡§¶
Citalopram/escitalopram	2	45	-	3.24 (0.73 to 14.40)	3.24 (0.73 to 14.40)	76 (42 to 94)	Low†§¶
Disulfiram	2	221	0.79 (0.29 to 2.12)	2.34 (0.50 to 10.94)	1.05 (0.49 to 2.28)	51 (33 to 69)	Low†‡§¶
Fluoxetine	1	25	1.07 (0.33 to 3.46)	-	1.07 (0.33 to 3.46)	52 (25 to 78)	Very low†‡§¶
Flupenthixol	1	142	2.37 (1.27 to 4.40)	-	2.37 (1.27 to 4.40)	70 (56 to 81)	Low†¶
Fluvoxamine	2	268	2.07 (1.09 to 3.93)	9.15 (0.40 to 209.33)	2.15 (1.30 to 3.55)	68 (57 to 78)	Low†¶**
Galantamine	1	74	1.15 (0.50 to 2.64)	-	1.15 (0.50 to 2.64)	54 (36 to 71)	Very low†§¶
GHB	4	201	0.70 (0.34 to 1.42)	0.42 (0.11 to 1.57)	0.63 (0.36 to 1.10)	39 (27 to 52)	Low†‡§
Levetiracetam	1	95	0.44 (0.19 to 1.02)	-	0.44 (0.19 to 1.02)	31 (16 to 50)	Very low†§¶
Lisuride	1	57	1.70 (0.57 to 5.10)	-	1.70 (0.57 to 5.10)	63 (36 to 84)	Very low†§¶
Lithium	1	28	1.08 (0.35 to 3.36)	-	1.08 (0.35 to 3.36)	52 (26 to 77)	Very low†§¶
Modafinil	1	41	1.28 (0.49 to 3.30)	-	1.28 (0.49 to 3.30)	56 (33 to 77)	Very low†§¶
Naltrexone	17	878	0.77 (0.49 to 1.20)	0.57 (0.27 to 1.17)	0.70 (0.50 to 0.98)	41 (33 to 50)	Moderate†§
Nefazodone	1	50	1.63 (0.63 to 4.23)	-	1.63 (0.63 to 4.23)	62 (39 to 81)	Very low†‡§¶
Oxcarbazepine	2	72	-	0.54 (0.20 to 1.45)	0.54 (0.20 to 1.45)	35 (17 to 59)	Low†§¶
Pregabalin	1	31	-	0.31 (0.07 to 1.31)	0.31 (0.07 to 1.31)	24 (7 to 57)	Low†§¶
Quetiapine	1	29	0.78 (0.22 to 2.74)	-	0.78 (0.22 to 2.74)	44 (18 to 73)	Low†§¶
Tianeptine	1	170	1.60 (0.92 to 2.80)	-	1.60 (0.92 to 2.80)	62 (48 to 74)	Low†§¶
Tiapride	2	187	0.76 (0.43 to 1.33)	-	0.76 (0.43 to 1.33)	43 (30 to 57)	Moderate§
Topiramate	3	194	0.42 (0.16 to 1.10)	0.47 (0.19 to 1.21)	0.45 (0.24 to 0.83)	31 (19 to 45)	Low†‡§¶
Trazodone	1	88	0.96 (0.41 to 2.22)	-	0.96 (0.41 to 2.22)	49 (29 to 69)	Very low†‡§¶
**Combined interventions**							
Placebo+CBT	1	50	1.00 (0.40 to 2.49)	-	1.00 (0.40 to 2.49)	50 (29 to 71)	Very low†‡§¶
Nefazodone+CBT	1	53	1.09 (0.44 to 2.70)	-	1.09 (0.44 to 2.70)	52 (31 to 73)	Very low†‡§¶
Acamprosate+nurse visit	1	50	-	0.21 (0.07 to 0.57)	0.21 (0.07 to 0.57)	17 (7 to 37)	Low†§¶
Acamprosate+NXT	1	40	0.18 (0.06 to 0.53)	0.81 (0.17 to 3.80)	0.30 (0.13 to 0.67)	23 (12 to 40)	Moderate†¶**
GHB+EST	1	12	-	0.99 (0.03 to 37.75)	0.99 (0.03 to 37.75)	50 (3 to 97)	Very low†§¶
GHB+NTX	1	18	-	0.64 (0.13 to 3.13)	0.64 (0.13 to 3.13)	39 (12 to 76)	Very low†‡§¶
NTX+EST	1	12	-	0.99 (0.03 to 37.75)	0.99 (0.03 to 37.75)	50 (3 to 97)	Very low†§¶
NTX+GHB+EST	1	12	-	0.99 (0.03 to 37.75)	0.99 (0.03 to 37.75)	50 (3 to 97)	Very low†§¶

A-CHESS=Addiction‐Comprehensive Health Enhancement Support System; CBT=cognitive behavioural therapy; MET=motivational enhancement therapy; GHB=sodium salt of gamma hydroxybutyric acid (sodium oxybate); EST=escitalopram; NTX=naltrexone.

* See supplement 4 for full details of criteria for downgrading quality of evidence.

† Within study bias.

‡ Indirectness.

§ Imprecision.

¶ Heterogeneity.

** Incoherence.

### Clustered rank of treatments

**Fig 4:**
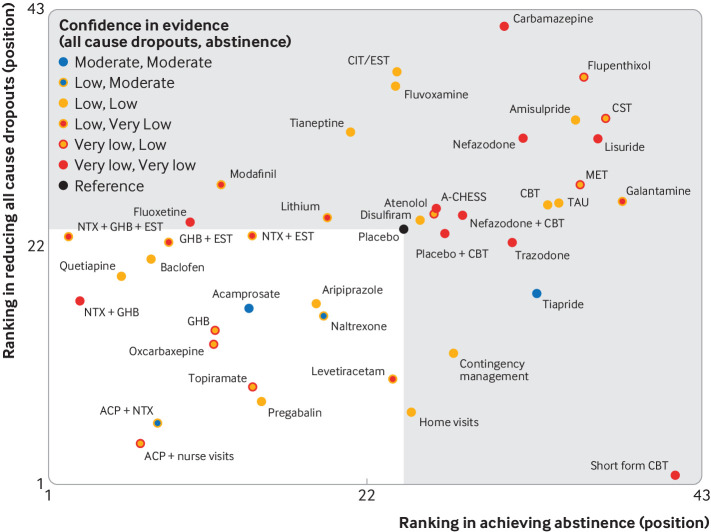
Clustered ranking plot by mean rank values from results of network meta-analyses of abstinence and all cause dropouts. Interventions are coloured according to the confidence of evidence by outline (abstinence) and fill (all cause dropout). The interventions in the white zone were ranked better than placebo based on both outcomes. A-CHESS=Addiction-Comprehensive Health Enhancement Support System; ACP=acamprosate; CBT=cognitive behavioural therapy; CIT=citalopram; EST=escitalopram; CST=coping skill training; GHB=sodium salt of gamma hydroxybutyric acid (sodium oxybate); MET=motivational enhancement therapy; NTX=naltrexone; TAU=treatment as usual

The mean rank of each treatment was plotted to illustrate clustering of interventions according to higher effectiveness (maintaining abstinence) and higher acceptability (reducing dropout), as well as illustrating the corresponding confidence in the evidence (fig 4). Although many interventions cluster in the lower left hand corner of the figure (indicating higher rank on both outcomes than placebo), the low or very low confidence in the evidence limited the credibility of all interventions except for acamprosate.

### Results of additional analysis

Additional network meta-analysis stratified by intervention types did not show significant differences in the relative intervention effects (supplement 8). No evidence was found of heterogeneity being explained by meta-regression on predefined study level characteristics, although this was limited by the number of studies in each intervention and the quality of reporting. Only eight studies (seven interventions) had results for the long term analysis. Acamprosate was the only intervention associated with improved maintenance of abstinence compared with placebo (odds ratio 1.49, 95% confidence interval 0.82 to 2.71), although the evidence is weak.

## Discussion

Acamprosate is the only intervention with enough evidence to conclude that it is better than placebo in supporting detoxified, alcohol dependent patients to maintain abstinence for up to 12 months in primary care settings. Weak evidence suggested that acamprosate might be effective in the longer term. It is uncertain whether the other current licensed drugs, naltrexone and disulfiram, improve abstinence in such patients. Although other interventions, such as sodium oxybate, pregabalin, topiramate, and combinations of other interventions, such as acamprosate and naltrexone, acamprosate and nurse visits, and sodium oxybate and naltrexone, may lead to better outcomes than placebo, the low numbers of studies and patients reduced our confidence in the evidence.

### Strengths and limitations of this review

Our research question and study eligibility criteria were designed to align with current practice to bridge the evidence gap in the care pathway of recently detoxified, alcohol dependent patients in a primary care setting. A main strength of our study is the sensitive search strategies and snowballing technique used to retrieve potentially eligible studies. These were required because the titles, abstracts, and indexes of many studies do not contain keywords or are poorly indexed. We also included all available interventions suitable for primary care to provide an extensive list as a reference for clinical practice.

Our review also has limitations. Firstly, we focused on the maintenance of abstinence. While this is of direct relevance to clinical practice and service planning, it means we excluded studies that investigated the effect of interventions in reducing alcohol consumption in alcohol dependent people who are still drinking (eg, nalmefene^46^ and topiramate^46^) rather than maintaining abstinence. Previous reviews of interventions in abstinent alcohol dependence (ie, post-detoxification) have reported evidence of naltrexone^47^ ^48^ ^49^ in preventing relapse from a lapse, and for acamprosate^48^ ^50^ or disulfiram^51^ in maintaining abstinence in patients with alcohol dependence. These three drugs are recommended by NICE^6^ for clinical effectiveness, and choice is tailored depending on the drinking behaviour and clinical situation of a patient. It remains unclear which method of achieving abstinence is more effective for patients with severe alcohol dependency, detoxification or gradual weaning. A shared decision on the treatment goal should be discussed and made between a patient and a prescriber. Secondly, definitions of detoxification were ambiguous and poorly reported across studies, meaning that we might have included studies when participants were not truly detoxified and missed studies when participants were detoxified but not described as such. Although we tried to contact study authors to confirm whether participants were detoxified in unclear cases, it was often not possible, especially in earlier published studies. Additionally, this (similar to inconsistent reporting in socioeconomic data and characteristics of participants) limited us in the performance of additional analysis in how subsets of particular patients respond to an intervention. Thirdly, the use of all cause dropout as a proxy for acceptability was driven primarily by practical considerations because dropouts often occur in these patients and are well reported across studies. Other outcomes should be taken into account to draw firm conclusions about acceptability of the interventions.^52^ Finally, most included studies were conducted in the United States and Europe, which could have implications for applicability to the UK setting. We excluded studies conducted in hospital and intensive rehabilitation settings to give a comprehensive overview of available interventions that could be considered in primary care settings. The translation of evidence into practice still requires thorough evaluation among patients, practitioners, policymakers, and stakeholders, which could be aided using knowledge translation frameworks.^53^ ^54^

### Comparison with other reviews

Our findings agree with previous systematic reviews supporting the use of acamprosate in detoxified patients with alcohol dependency^50^ ^55^ ^56^ ^57^ and are in line with recommended guidelines.^6^ ^58^ Previous reviews of interventions to maintain abstinence in alcohol dependent patients have mostly focused on a single specific intervention or group of interventions, and synthesised studies conducted in a variety of populations, which makes them less applicable to clinical practice. A recent systematic review and network meta-analysis,^46^ which only included five interventions, exemplifies this problem. In contrast, we included different psychosocial and drug interventions that could be implemented in primary care using network meta-analysis. One study^51^ suggested that disulfiram is effective based on a meta-analysis of different alcohol drinking outcomes and a high heterogeneity of the studies, whereas we found limited evidence to support its effect on abstinence after evaluating studies using rigorous approaches. Of all the drugs studied, disulfiram provides particular challenges in placebo controlled blinded trials because, compared with the other drugs, patients can more easily determine if they are or are not taking disulfiram.

### Conclusions and implications

Most interventions identified in this review were not associated with enough evidence to make recommendations in clinical practice, although many are promising and should be investigated in future trials. The findings also provide directions for potential strategies by including additional interventions to complement the treatment effects, such as naltrexone or home visits. These could contribute to a pragmatic trial design.^59^ However, the mechanism of acamprosate and other interventions on treating alcohol dependence remains unclear. Establishing the properties of drugs and understanding the psychological needs of alcohol dependent patients or the fundamental causes of alcohol dependency could also inform new strategies for future trial designs.

These findings have important implications for clinical practice, as acamprosate was found to be the only intervention with enough high quality evidence for us to conclude that it is better at maintaining alcohol abstinence than placebo. The finding that there is currently little evidence on other interventions, such as disulfiram, for detoxified, alcohol dependent patients in UK primary care settings should lead to the generation of better evidence from high quality pragmatic randomised trials.

**What is already known on this topic**

A considerable need exists for the management of patients with alcohol dependence that cannot be met by specialist services alone but only through expanding treatment in primary care
This is not yet reflected in UK clinical guidance
Current evidence is generally based on single or specific interventions with broad inclusion criteria and mixed outcomes in patients with alcohol dependency

**What this study adds**

Acamprosate was the only intervention with enough evidence of benefit for maintaining alcohol abstinence and acceptability up to 12 months after detoxification in primary care settings
The quality of evidence was mostly low and most of the evidence was derived from single, small trials
More evidence from high quality randomised controlled trials is therefore needed on the best interventions in primary care to inform clinical treatment and enable patient access

## Web extra

Extra material supplied by authors

Supplementary information: additional tables and figures supplied by authors

## Notes

Contributors: SD and HC were responsible for the search results and maintaining the database of references. HC, LAM, RGE, AT, and GJM conducted screening. HC, AT, LAM, RGE, and GJM conducted data extraction. HC, LAM, RGE, GJM, and AM conducted the risk of bias assessment. HC, AM, and JPTH conducted the GRADE assessment. HC conducted analyses and was supervised by JLL and JPTH. MH and DK conceived the review. MH, DK, JPTH, and HC led the design of the review All authors critically reviewed and provided feedback on the manuscript. All authors read and approved the manuscript before its submission. The corresponding author attests that all listed authors meet authorship criteria and that no others meeting the criteria have been omitted. DK and MH act as guarantors.

Ethical approval: Not required.

Data sharing: Dataset and codes used for network meta-analysis can be found in *data.bris* repository (https://data.bris.ac.uk/data/dataset/duuzaux6mg9m2dbe1hnswvz6w). Additional information and data are available from the corresponding author.

The lead authors (DK and MH) affirm that the manuscript is an honest, accurate, and transparent account of the study being reported; that no important aspects of the study have been omitted; and that any discrepancies from the study as planned (and, if relevant, registered) have been explained.

Dissemination to participants and related patients and public communities: Preliminary results of this study were presented to healthcare professionals, researchers in primary care, and patients in South West Society for Academic Primary Care 2020 Bristol conference on 6 March 2020 (session 4C.3 https://sapc.ac.uk/doi/10.37361/sw.2020.1.1). We will disseminate the results widely using traditional media and social media through communication offices of Centre for Academic Primary Care and our National Institute for Health Research infrastructure (School of Primary Care Research and Health Protection Research Unit) in our university and networks with other universities (eg, GW4 Alliance of four major universities in south west England). We will also disseminate the results to trial authors who were contacted during the review and showed interest in our work.

Provenance and peer review: Not commissioned; externally peer reviewed.

## References

[1] NHS Digital. Statistics on Alcohol: England, 2018. 2018. digital.nhs.uk/data-and-information/publications/statistical/statistics-on-alcohol/2018. (Accessed 22 March 2019).

[2] Williams R Aspinall R Bellis M Addressing liver disease in the UK: a blueprint for attaining excellence in health care and reducing premature mortality from lifestyle issues of excess consumption of alcohol, obesity, and viral hepatitis. Lancet 2014;384:1953-97. 10.1016/S0140-6736(14)61838-9. 25433429

[3] Health Social Care Information Centre Statistics on Alcohol. 2016.

[4] Public Health England Adult substance misuse statistics from the National Drug Treatment Monitoring System (NDTMS). Public Health England, 2017.

[5] Dawson DA Grant BF Stinson FS Chou PS Estimating the effect of help-seeking on achieving recovery from alcohol dependence. Addiction 2006;101:824-34. 10.1111/j.1360-0443.2006.01433.x. 16696626

[6] National Collaborating Centre for Mental Health (UK). Alcohol-Use Disorders: Diagnosis, Assessment and Management of Harmful Drinking and Alcohol Dependence. Leicester (UK): British Psychological Society; The British Psychological Society & The Royal College of Psychiatrists. 2011. PMID: 22624177. 22624177

[7] Public Health England. Adult substance misuse treatment statistics 2018 to 2019: report. https://www.gov.uk/government/publications/substance-misuse-treatment-for-adults-statistics-2018-to-2019/adult-substance-misuse-treatment-statistics-2018-to-2019-report. 2019. (Accessed 22 March 2019).

[8] Gillam S Is the declaration of Alma Ata still relevant to primary health care? BMJ 2008;336:536-8. 10.1136/bmj.39469.432118.AD. 18325964 PMC2265356

[9] Miller WR Wilbourne PL Mesa Grande: a methodological analysis of clinical trials of treatments for alcohol use disorders. Addiction 2002;97:265-77. 10.1046/j.1360-0443.2002.00019.x. 11964100

[10] Cheng HYE Elbers RG Higgins JPT Therapeutic interventions for alcohol dependence in non-inpatient settings: a systematic review and network meta-analysis (protocol). Syst Rev 2017;6:77. 10.1186/s13643-017-0462-2. 28399899 PMC5387199

[11] Hutton B Salanti G Caldwell DM The PRISMA extension statement for reporting of systematic reviews incorporating network meta-analyses of health care interventions: checklist and explanations. Ann Intern Med 2015;162:777-84. 10.7326/M14-2385. 26030634

[12] UKATT Research Team Effectiveness of treatment for alcohol problems: findings of the randomised UK alcohol treatment trial (UKATT). BMJ 2005;331:541. 10.1136/bmj.331.7516.541. 16150764 PMC1200586

[13] Adamson SJH Heather N Morton V Raistrick D UKATT Research Team Initial preference for drinking goal in the treatment of alcohol problems: II. Treatment outcomes. Alcohol Alcohol 2010;45:136-42. 10.1093/alcalc/agq005. 20130150

[14] Dawson DAG Goldstein RB Grant BF Rates and correlates of relapse among individuals in remission from DSM-IV alcohol dependence: a 3-year follow-up. Alcohol Clin Exp Res 2007;31:2036-45. 10.1111/j.1530-0277.2007.00536.x. 18034696

[15] Greenhalgh T Peacock R Effectiveness and efficiency of search methods in systematic reviews of complex evidence: audit of primary sources. BMJ 2005;331:1064-5. 10.1136/bmj.38636.593461.68. 16230312 PMC1283190

[16] Pelc I, Le Bon O, Verbanck, P, Lehert, P, Opsome, L. Calcium acetyl homotaurinate for maintaining abstinence in weaned alcoholic patients: a placebo-controlled double-blind multi-centre study. In: Naranjo CA, EdSellers EM, eds. *Novel pharmacological interventions for alcoholism:* *Satellite symposium: Papers and abstracts. *New York; Berlin: Springer 1992:348-52.

[17] Mann K Lehert P Morgan MY The efficacy of acamprosate in the maintenance of abstinence in alcohol-dependent individuals: results of a meta-analysis. Alcohol Clin Exp Res 2004;28:51-63. 10.1097/01.ALC.0000108656.81563.05. 14745302

[18] Sterne JAC Savović J Page MJ RoB 2: a revised tool for assessing risk of bias in randomised trials. BMJ 2019;366:l4898. 10.1136/bmj.l4898. 31462531

[19] Nikolakopoulou A Higgins JPT Papakonstantinou T CINeMA: An approach for assessing confidence in the results of a network meta-analysis. PLoS Med 2020;17:e1003082. 10.1371/journal.pmed.1003082. 32243458 PMC7122720

[20] Papakonstantinou T Nikolakopoulou A Higgins JPT CINeMA: Software for semiautomated assessment of the confidence in the results of network meta-analysis. Campbell Syst Rev 2020;16:e1080 10.1002/cl2.1080. PMC8356302 37131978

[21] Salanti G Del Giovane C Chaimani A Caldwell DM Higgins JPT Evaluating the quality of evidence from a network meta-analysis. PLoS One 2014;9:e99682. 10.1371/journal.pone.0099682. 24992266 PMC4084629

[22] Chaimani A Higgins JPT Mavridis D Spyridonos P Salanti G Graphical tools for network meta-analysis in STATA. PLoS One 2013;8:e76654. 10.1371/journal.pone.0076654 24098547 PMC3789683

[23] Fuller RK Branchey L Brightwell DR Disulfiram treatment of alcoholism. A Veterans Administration cooperative study. JAMA 1986;256:1449-55. 10.1001/jama.1986.03380110055026 3528541

[24] Stella L Addolorato G Rinaldi B An open randomized study of the treatment of escitalopram alone and combined with gamma-hydroxybutyric acid and naltrexone in alcoholic patients. Pharmacol Res 2008;57:312-7. 10.1016/j.phrs.2008.03.001 18434189

[25] Schünemann HJ, Higgins JPT, Vist GE, et al. Chapter 14. Section 14.1.5: Completing ‘Summary of findings’ tables and grading the certainty of the evidence. In: Higgins JPT, Thomas J, Chandler J, et al, eds. Cochrane Handbook for Systematic Reviews of Interventions**version 6.1 (updated September 2020). Cochrane, 2020 www.training.cochrane.org/handbook. (Accessed 25 October 2020).

[26] Higgins JPT Jackson D Barrett JK Lu G Ades AE White IR Consistency and inconsistency in network meta-analysis: concepts and models for multi-arm studies. Res Synth Methods 2012;3:98-110. 10.1002/jrsm.1044 26062084 PMC4433772

[27] Dias S Welton NJ Caldwell DM Ades AE Checking consistency in mixed treatment comparison meta-analysis. Stat Med 2010;29:932-44. 10.1002/sim.3767 20213715

[28] Liu Y Wang W Zhang AB Bai X Zhang S Epley and Semont maneuvers for posterior canal benign paroxysmal positional vertigo: A network meta-analysis. Laryngoscope 2016;126:951-5. 10.1002/lary.25688 26403977

[29] Bastian M, Heymann S, Jacomy M. Gephi: an open source software for exploring and manipulating networks. International AAAI Conference on Weblogs and Social Media. 2009.

[30] Martinotti G Romanelli R Di Nicola M Reina D Mazza M Janiri L Oxcarbazepine at high dosages for the treatment of alcohol dependence [Letter]. Am J Addict 2007;16:247-8. 10.1080/10550490701375558 17612833

[31] Gual A Lehert P Acamprosate during and after acute alcohol withdrawal: a double-blind placebo-controlled study in Spain. Alcohol Alcohol 2001;36:413-8. 10.1093/alcalc/36.5.413 11524307

[32] Cornelius JR Salloum IM Ehler JG Fluoxetine in depressed alcoholics. A double-blind, placebo-controlled trial. Arch Gen Psychiatry 1997;54:700-5. 10.1001/archpsyc.1997.01830200024004 9283504

[33] Cornelius JRS Salloum IM Haskett RF Fluoxetine versus placebo in depressed alcoholics: a 1-year follow-up study. Addict Behav 2000;25:307-10. 10.1016/S0306-4603(99)00065-9 10795957

[34] Friedmann PD Rose JS Swift R Stout RL Millman RP Stein MD Trazodone for sleep disturbance after alcohol detoxification: a double-blind, placebo-controlled trial. Alcohol Clin Exp Res 2008;32:1652-60. 10.1111/j.1530-0277.2008.00742.x 18616688 PMC2567128

[35] Oslin DW Treatment of late-life depression complicated by alcohol dependence. Am J Geriatr Psychiatry 2005;13:491-500. 10.1097/00019442-200506000-00008 15956269 PMC3172720

[36] Ponce G Sánchez-García J Rubio G Rodríguez-Jiménez R Jiménez-Arriero MA Palomo T Eficacia de la naltrexona en el tratamiento de mujeres con trastorno por dependencia del alcohol. Actas Esp Psiquiatr 2005;33:13-8. 15704026

[37] Gustafson DH McTavish FM Chih M-Y A smartphone application to support recovery from alcoholism: a randomized clinical trial. JAMA Psychiatry 2014;71:566-72. 10.1001/jamapsychiatry.2013.4642 24671165 PMC4016167

[38] Burtscheidt W Wölwer W Schwarz R Strauss W Gaebel W Out-patient behaviour therapy in alcoholism: treatment outcome after 2 years. Acta Psychiatr Scand 2002;106:227-32. 10.1034/j.1600-0447.2002.02332.x 12197862

[39] Burtscheidt W Wölwer W Schwarz R Out-patient behaviour therapy in alcoholism: relapse rates after 6 months. Acta Psychiatr Scand 2001;103:24-9. 10.1111/j.1600-0447.2001.00150.x 11202125

[40] Project MATCH Research Group Matching alcoholism treatments to client heterogeneity: treatment main effects and matching effects on drinking during treatment. J Stud Alcohol 1998;59:631-9. 10.15288/jsa.1998.59.631 9811084

[41] Coriale G De Rosa F Battagliese G Motivational enhancement therapy versus cognitive behavioral therapy in a cohort of men and women with alcohol use disorder. Biomedical Reviews 2019;30:125-35 10.14748/bmr.v30.6393.

[42] Jirapramukpitak T Pattanaseri K Chua K-C Takizawa P Home-Based Contingency Management Delivered by Community Health Workers to Improve Alcohol Abstinence: A Randomized Control Trial. Alcohol Alcohol 2020;55:171-8. 10.1093/alcalc/agz106. 31919523

[43] Moraes E de Campos GM Figlie NB Ferraz MB Laranjeira R Home visits in the outpatient treatment of individuals dependent on alcohol: Randomized clinical trial. Addict Disord Their Treat 2010;9:18-31. 10.1097/ADT.0b013e318196d4e5.

[44] Pelc I Hanak C Baert I Effect of community nurse follow-up when treating alcohol dependence with acamprosate. Alcohol Alcohol 2005;40:302-7. 10.1093/alcalc/agh136 15870092

[45] Wetzel H Szegedi A Scheurich A NeVeR Study Group Combination treatment with nefazodone and cognitive-behavioral therapy for relapse prevention in alcohol-dependent men: a randomized controlled study. J Clin Psychiatry 2004;65:1406-13. 10.4088/JCP.v65n1017 15491246

[46] Palpacuer C Duprez R Huneau A Pharmacologically controlled drinking in the treatment of alcohol dependence or alcohol use disorders: a systematic review with direct and network meta-analyses on nalmefene, naltrexone, acamprosate, baclofen and topiramate. Addiction 2018;113:220-37. 10.1111/add.13974. 28940866

[47] Ahmed R Kotapati VP Khan AM Adding Psychotherapy to the Naltrexone Treatment of Alcohol Use Disorder: Meta-analytic Review. Cureus 2018;10:e3107. 10.7759/cureus.3107. 30338182 PMC6175267

[48] Donoghue K Elzerbi C Saunders R Whittington C Pilling S Drummond C The efficacy of acamprosate and naltrexone in the treatment of alcohol dependence, Europe versus the rest of the world: a meta-analysis. Addiction 2015;110:920-30. 10.1111/add.12875. 25664494

[49] Rösner S Hackl-Herrwerth A Leucht S Vecchi S Srisurapanont M Soyka M Opioid antagonists for alcohol dependence. Cochrane Database Syst Rev 2010;(12):CD001867. 10.1002/14651858.CD001867.pub2. 21154349 PMC13590218

[50] Rösner S Hackl-Herrwerth A Leucht S Lehert P Vecchi S Soyka M Acamprosate for alcohol dependence. Cochrane Database Syst Rev 2010;(9):CD004332. 10.1002/14651858.CD004332.pub2. 20824837 PMC12147086

[51] Skinner MD Lahmek P Pham H Aubin HJ Disulfiram efficacy in the treatment of alcohol dependence: a meta-analysis. PLoS One 2014;9:e87366. 10.1371/journal.pone.0087366. 24520330 PMC3919718

[52] Sekhon M Cartwright M Francis JJ Acceptability of healthcare interventions: an overview of reviews and development of a theoretical framework. BMC Health Serv Res 2017;17:88. 10.1186/s12913-017-2031-8. 28126032 PMC5267473

[53] Harrison MB Graham ID van den Hoek J Dogherty EJ Carley ME Angus V Guideline adaptation and implementation planning: a prospective observational study. Implement Sci 2013;8:49. 10.1186/1748-5908-8-49. 23656884 PMC3668213

[54] Guideline International Network. https://g-i-n.net/ 2020. (Accessed on 1 May 2020).

[55] Lejoyeux M Lehert P Alcohol-use disorders and depression: results from individual patient data meta-analysis of the acamprosate-controlled studies. Alcohol Alcohol 2011;46:61-7. 10.1093/alcalc/agq077 21118900

[56] Boothby LA Doering PL Acamprosate for the treatment of alcohol dependence [Review]. Clin Ther 2005;27:695-714. 10.1016/j.clinthera.2005.06.015 16117977

[57] Mann K Lehert P Morgan MY The efficacy of acamprosate in the maintenance of abstinence in alcohol-dependent individuals: results of a meta-analysis. Alcohol Clin Exp Res 2004;28:51-63. 10.1097/01.ALC.0000108656.81563.05 14745302

[58] Lingford-Hughes AR Welch S Peters L Nutt DJ British Association for Psychopharmacology, Expert Reviewers Group BAP updated guidelines: evidence-based guidelines for the pharmacological management of substance abuse, harmful use, addiction and comorbidity: recommendations from BAP. J Psychopharmacol 2012;26:899-952. 10.1177/0269881112444324. 22628390

[59] Timko C Below M Schultz NR Brief D Cucciare MA Patient and program factors that bridge the detoxification-treatment gap: a structured evidence review. J Subst Abuse Treat 2015;52:31-9. 10.1016/j.jsat.2014.11.009 25530425

