# Supplementary material for: Treatment interventions to maintain abstinence from alcohol in primary care: systematic review and network meta-analysis
Source: BMJ. 2020 Nov 25;371:m3934. doi: 10.1136/bmj.m3934 (PMC7687021; doi:10.1136/bmj.m3934)

List of Supplements:

Original Table 2 (page 2)

Original Table 3 (page 3)

Supplement 1. PRISMA NMA Checklist (page 4)

Supplement 2. Search strategies (page 8)

Supplement 3. List of excluded interventions (page 16)

Supplement 4. Criteria of GRADE assessments by CINeMA (page 20)

Supplement 5. Characteristics of the included studies (page 21)

Supplement 6. Results of risk of bias assessment (page 37)

Supplement 7. Network meta-analysis results (page 75)

Supplement 8. Additional analyses (page 83)

TABLE 2. NETWORK META-ANALYSIS AND QUALITY OF EVIDENCE FOR ABSTINENCE

Intervention (reference: placebo)	N of arm	N of Pts	Odd ratios (95% confidence interval)				
	(PLA, n = 42)	(PLA, n = 4044)	Direct estimate	Indirect estimate	NMA forest plot	NMA estimate	Quality of evidence
Psychosocial interventions							
TAU	9	800	-	0.52 (0.29 to 0.94)		0.52 (0.29 to 0.94)	Low [a b c d]
A-CHESS	1	170	-	0.88 (0.35 to 2.21)		0.88 (0.35 to 2.21)	Very low [a b c d]
CBT	2	306	-	0.53 (0.23 to 1.22)		0.53 (0.23 to 1.22)	Low [a c d]
Short-form CBT	1	43	-	0.05 (0.00 to 1.16)		0.05 (0.00 to 1.16)	Very low [a c d]
Contingency management	1	79	-	0.78 (0.17 to 3.61)		0.78 (0.17 to 3.61)	Low [a b c]
CST	1	40	-	0.35 (0.10 to 1.19)		0.35 (0.10 to 1.19)	Very low [a c d]
Home visit	2	142	-	0.95 (0.32 to 2.85)		0.95 (0.32 to 2.85)	Low [a c d]
MET	2	308	-	0.45 (0.19 to 1.11)		0.45 (0.19 to 1.11)	Very low [a c d]
Pharmacological interventions							
Acamprosate	18	2297	1.92 (1.52 to 2.42)	0.74 (0.21 to 2.53)		1.86 (1.49 to 2.33)	Moderate [d e]
Amisulpride	1	37	0.39 (0.09 to 1.64)	-		0.39 (0.09 to 1.64)	Low [a c d]
Aripiprazole	1	29	-	1.49 (0.43 to 5.18)		1.49 (0.43 to 5.18)	Low [a c d]
Atenolol	1	50	0.85 (0.25 to 2.95)	-		0.85 (0.25 to 2.95)	Very low [a b c d]
Baclofen	1	28	4.63 (1.00 to 21.48)	-		4.63 (1.00 to 21.48)	Low [a c d]
Carbamazepine	1	13	0.55 (0.08 to 3.90)	-		0.55 (0.08 to 3.90)	Very low [a b c d]
Citalopram/Escitalopram	2	45	-	1.03 (0.33 to 3.16)		1.03 (0.33 to 3.16)	Low [a c d]
Disulfiram	2	221	0.97 (0.46 to 2.01)	0.72 (0.13 to 4.05)		0.93 (0.48 to 1.79)	Low [a b c d]
Fluoxetine	2	50	2.14 (0.48 to 9.52)	4.51 (0.83 to 24.39)		2.97 (0.97 to 9.05)	Very low [a b c d]
Flupenthixol	1	142	0.44 (0.20 to 0.95)	-		0.44 (0.20 to 0.95)	Very low [a c d]
Fluvoxamine	3	293	0.99 (0.49 to 2.01)	1.14 (0.34 to 3.89)		1.03 (0.57 to 1.88)	Low [a c d]
Galantamine	1	74	0.31 (0.11 to 0.87)	-		0.31 (0.11 to 0.87)	Low [a c d]
GHB (sodium oxybate)	4	201	1.65 (0.85 to 3.24)	7.48 (2.05 to 27.28)		2.31 (1.22 to 4.36)	Very low [a b d e]
Levetiracetam	1	95	1.03 (0.46 to 2.34)	-		1.03 (0.46 to 2.34)	Low [a c d]
Lisuride	1	57	0.38 (0.13 to 1.12)	-		0.38 (0.13 to 1.12)	Very low [a c d]
Lithium	1	28	1.43 (0.39 to 5.23)	-		1.43 (0.39 to 5.23)	Low [a c d]
Modafinil	1	41	2.48 (0.72 to 8.53)	-		2.48 (0.72 to 8.53)	Low [a c d]
Naltrexone	17	878	1.29 (0.86 to 1.92)	1.59 (0.81 to 3.10)		1.36 (0.97 to 1.91)	Low [a c d]
Nefazodone	1	50	0.57 (0.19 to 1.76)	-		0.57 (0.19 to 1.76)	Very low [a b c d]
Oxcarbazepine	2	72	-	2.46 (0.91 to 6.61)		2.46 (0.91 to 6.61)	Very low [a c d]
Pregabalin	1	31	-	1.97 (0.58 to 6.74)		1.97 (0.58 to 6.74)	Low [a c d]
Quetiapine	1	29	6.75 (1.20 to 38.05)	-		6.75 (1.20 to 38.05)	Low [a c d]
Tianeptine	1	170	1.22 (0.58 to 2.57)	-		1.22 (0.58 to 2.57)	Low [a c d]
Tiapride	2	187	0.56 (0.30 to 1.05)	-		0.56 (0.30 to 1.05)	Moderate [c d]
Topiramate	3	194	2.26 (0.83 to 6.13)	1.72 (0.84 to 3.52)		1.88 (1.06 to 3.34)	Very low [a b c d e]
Trazodone	1	88	0.61 (0.20 to 1.84)	-		0.61 (0.20 to 1.84)	Very low [a b c d]
Combined interventions							
Placebo + CBT	1	50	0.83 (0.28 to 2.42)	-		0.83 (0.28 to 2.42)	Very low [a b c d]
Nefazodone + CBT	1	53	0.77 (0.26 to 2.23)	-		0.77 (0.26 to 2.23)	Very low [a b c d]
Acamprosate + Nurse visit	1	50	-	4.59 (1.47 to 14.36)		4.59 (1.47 to 14.36)	Very low [a c d]
Acamprosate + Naltrexone	1	40	5.57 (1.82 to 16.96)	1.63 (0.33 to 7.95)		3.68 (1.50 to 9.02)	Low [a c d e]
GHB + EST	1	12	-	5.13 (0.53 to 49.92)		5.13 (0.53 to 49.92)	Low [a c d]
GHB + NTX	1	18	-	12.64 (2.77 to 57.78)		12.64 (2.77 to 57.78)	Very low [a b c d]
NTX + EST	1	12	-	2.57 (0.25 to 25.85)		2.57 (0.25 to 25.85)	Low [a c d]
NTX + GHB + EST	1	12	-	25.65 (2.13 to 309.46)		25.65 (2.13 to 309.46)	Low [a c d]

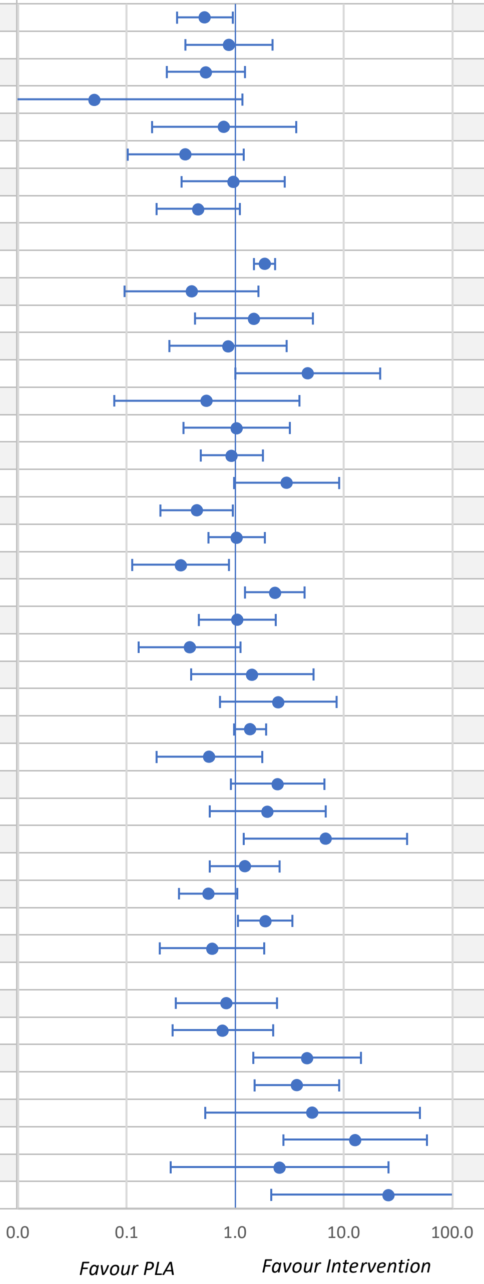

Reasons for downgrading: [a] due to within-study bias; [b] due to indirectness; [c] due to imprecision; [d] due to heterogeneity; [e] due to incoherence; see criteria on Supplement 4.

TABLE 3. NETWORK META-ANALYSIS AND QUALITY OF EVIDENCE FOR ALL-CAUSE DROPOUTS

Intervention (reference: placebo)	N of arm (PLA, n = 41)	N of Pts (PLA, n = 4012)	Odd ratio (95% confidence interval) Direct estimate	Indirect estimate	NMA Forest plot	NMA estimate	Quality of evidence
Psychosocial interventions							
TAU	9	800	-	1.14 (0.65 to 1.99)		1.14 (0.65 to 1.99)	Low [a,b,c,d]
A-CHESS	1	170	-	1.14 (0.50 to 2.60)		1.14 (0.50 to 2.60)	Very low [a,b,c,d]
CBT	2	306	-	1.16 (0.45 to 3.04)		1.16 (0.45 to 3.04)	Low [a,c,d]
Short-form CBT	1	43	-	0.06 (0.01 to 0.33)		0.06 (0.01 to 0.33)	Very low [a,c,d]
Contingency management	1	79	-	0.32 (0.02 to 6.55)		0.32 (0.02 to 6.55)	Low [a,b,c]
CST	1	40	-	1.98 (0.55 to 7.17)		1.98 (0.55 to 7.17)	Low [a,c,d]
Home visit	2	142	-	0.32 (0.11 to 0.95)		0.32 (0.11 to 0.95)	Low [a,c,d]
MET	2	308	-	1.30 (0.46 to 3.64)		1.30 (0.46 to 3.64)	Low [a,c,d]
Pharmacological interventions							
Acamprosate	17	2268	0.71 (0.58 to 0.87)	1.17 (0.31 to 4.34)		0.73 (0.62 to 0.86)	Moderate [c,e]
Amisulpride	1	37	1.89 (0.66 to 5.43)	-		1.89 (0.66 to 5.43)	Low [a,c,d]
Aripiprazole	1	29	-	0.67 (0.18 to 2.45)		0.67 (0.18 to 2.45)	Low [a,c,d]
Atenolol	1	50	1.09 (0.46 to 2.57)	-		1.09 (0.46 to 2.57)	Low [a,b,c,d]
Baclofen	1	28	0.87 (0.29 to 2.62)	-		0.87 (0.29 to 2.62)	Low [a,c,d]
Carbamazepine	1	13	12.00 (1.22 to 118.42)	-		12.00 (1.22 to 118.42)	Very low [a,b,c,d]
Citalopram/Escitalopram	2	45	-	3.24 (0.73 to 14.40)		3.24 (0.73 to 14.40)	Low [a,c,d]
Disulfiram	2	221	0.79 (0.29 to 2.12)	2.34 (0.50 to 10.94)		1.05 (0.49 to 2.28)	Low [a,b,c,d]
Fluoxetine	1	25	1.07 (0.33 to 3.46)	-		1.07 (0.33 to 3.46)	Very low [a,b,c,d]
Flupenthixol	1	142	2.37 (1.27 to 4.40)	-		2.37 (1.27 to 4.40)	Low [a,d]
Fluvoxamine	2	268	2.07 (1.09 to 3.93)	9.15 (0.40 to 209.33)		2.15 (1.30 to 3.55)	Low [a,d,e]
Galantamine	1	74	1.15 (0.50 to 2.64)	-		1.15 (0.50 to 2.64)	Very low [a,c,d]
GHB (sodium oxybate)	4	201	0.70 (0.34 to 1.42)	0.42 (0.11 to 1.57)		0.63 (0.36 to 1.10)	Low [a,b,c]
Levetiracetam	1	95	0.44 (0.19 to 1.02)	-		0.44 (0.19 to 1.02)	Very low [a,c,d]
Lisuride	1	57	1.70 (0.57 to 5.10)	-		1.70 (0.57 to 5.10)	Very low [a,c,d]
Lithium	1	28	1.08 (0.35 to 3.36)	-		1.08 (0.35 to 3.36)	Very low [a,c,d]
Modafinil	1	41	1.28 (0.49 to 3.30)	-		1.28 (0.49 to 3.30)	Very low [a,c,d]
Naltrexone	17	878	0.77 (0.49 to 1.20)	0.57 (0.27 to 1.17)		0.70 (0.50 to 0.98)	Moderate [a,c]
Nefazodone	1	50	1.63 (0.63 to 4.23)	-		1.63 (0.63 to 4.23)	Very low [a,b,c,d]
Oxcarbazepine	2	72	-	0.54 (0.20 to 1.45)		0.54 (0.20 to 1.45)	Low [a,c,d]
Pregabalin	1	31	-	0.31 (0.07 to 1.31)		0.31 (0.07 to 1.31)	Low [a,c,d]
Quetiapine	1	29	0.78 (0.22 to 2.74)	-		0.78 (0.22 to 2.74)	Low [a,c,d]
Tianeptine	1	170	1.60 (0.92 to 2.80)	-		1.60 (0.92 to 2.80)	Low [a,c,d]
Tiapride	2	187	0.76 (0.43 to 1.33)	-		0.76 (0.43 to 1.33)	Moderate [c]
Topiramate	3	194	0.42 (0.16 to 1.10)	0.47 (0.19 to 1.21)		0.45 (0.24 to 0.83)	Low [a,b,c,d]
Trazodone	1	88	0.96 (0.41 to 2.22)	-		0.96 (0.41 to 2.22)	Very low [a,b,c,d]
Combined interventions							
Placebo + CBT	1	50	1.00 (0.40 to 2.49)	-		1.00 (0.40 to 2.49)	Very low [a,b,c,d]
Nefazodone + CBT	1	53	1.09 (0.44 to 2.70)	-		1.09 (0.44 to 2.70)	Very low [a,b,c,d]
Acamprosate + Nurse visit	1	50	-	0.21 (0.07 to 0.57)		0.21 (0.07 to 0.57)	Low [a,c,d]
Acamprosate + Naltrexone	1	40	0.18 (0.06 to 0.53)	0.81 (0.17 to 3.80)		0.30 (0.13 to 0.67)	Moderate [a,d,e]
GHB + EST	1	12	-	0.99 (0.03 to 37.75)		0.99 (0.03 to 37.75)	Very low [a,c,d]
GHB + NTX	1	18	-	0.64 (0.13 to 3.13)		0.64 (0.13 to 3.13)	Very low [a,b,c,d]
NTX + EST	1	12	-	0.99 (0.03 to 37.75)		0.99 (0.03 to 37.75)	Very low [a,c,d]
NTX + GHB + EST	1	12	-	0.99 (0.03 to 37.75)		0.99 (0.03 to 37.75)	Very low [a,c,d]

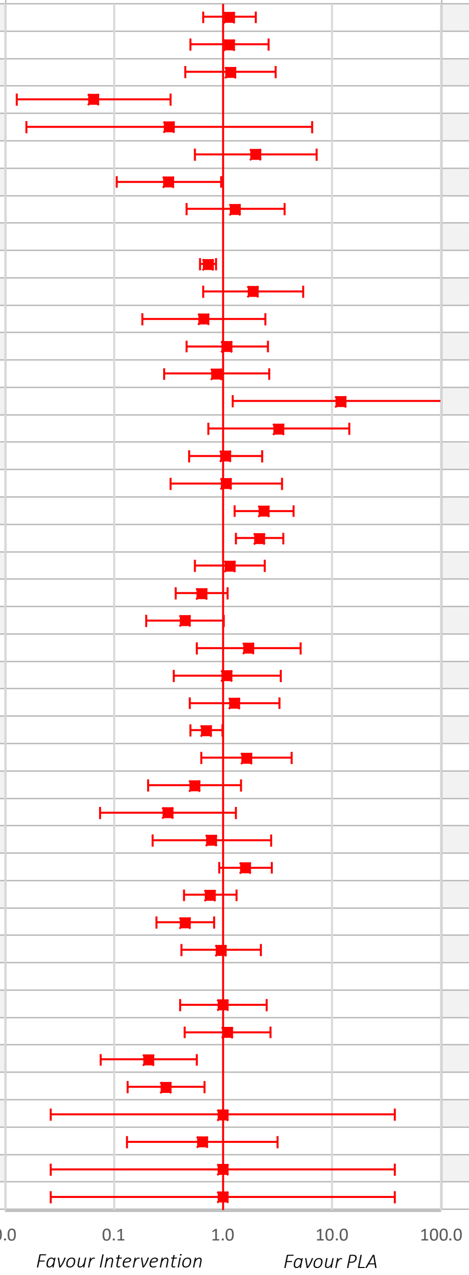

Reasons for downgrading: [a] due to within-study bias; [b] due to indirectness; [c] due to imprecision; [d] due to heterogeneity; [e] due to incoherence; see criteria on Supplement 4.

SUPPLEMENT 1. PRISMA NMA CHECKLIST

Section/Topic	Item #	Checklist Item	Reported on Page #
**TITLE**			
Title	1	Identify the report as a systematic review *incorporating a network meta-analysis (or related form of meta-analysis).*	***1***
**ABSTRACT**			
Structured summary	2	Provide a structured summary including, as applicable:<br>**Background:** main objectives<br>**Methods:** data sources; study eligibility criteria, participants, and interventions; study appraisal; and *synthesis methods, such as network meta-analysis.*<br>**Results:** number of studies and participants identified; summary estimates with corresponding confidence/credible intervals; *treatment rankings may also be discussed. Authors may choose to summarize pairwise comparisons against a chosen treatment included in their analyses for brevity.*<br>**Discussion/Conclusions:** limitations; conclusions and implications of findings.<br>**Other:** primary source of funding; systematic review registration number with registry name.	***2***
**INTRODUCTION**			
Rationale	3	Describe the rationale for the review in the context of what is already known*, including mention of why a network meta-analysis has been conducted.*	***4***
Objectives	4	Provide an explicit statement of questions being addressed, with reference to participants, interventions, comparisons, outcomes, and study design (PICOS).	***4***
**METHODS**			
Protocol and registration	5	Indicate whether a review protocol exists and if and where it can be accessed (e.g., Web address); and, if available, provide registration information, including registration number.	***4***
Eligibility criteria	6	Specify study characteristics (e.g., PICOS, length of follow-up) and report characteristics (e.g., years considered, language, publication status) used as criteria for eligibility, giving rationale. *Clearly describe eligible treatments included in the treatment network, and note whether any have been clustered or merged into the same node (with justification).*	***4-5***

Information sources	7	Describe all information sources (e.g., databases with dates of coverage, contact with study authors to identify additional studies) in the search and date last searched.	***5***
Search	8	Present full electronic search strategy for at least one database, including any limits used, such that it could be repeated.	***5 and Supp 2***
Study selection	9	State the process for selecting studies (i.e., screening, eligibility, included in systematic review, and, if applicable, included in the meta-analysis).	***5***
Data collection process	10	Describe method of data extraction from reports (e.g., piloted forms, independently, in duplicate) and any processes for obtaining and confirming data from investigators.	***6***
Data items	11	List and define all variables for which data were sought (e.g., PICOS, funding sources) and any assumptions and simplifications made.	***6***
**Geometry of the network**	**S1**	Describe methods used to explore the geometry of the treatment network under study and potential biases related to it. This should include how the evidence base has been graphically summarized for presentation, and what characteristics were compiled and used to describe the evidence base to readers.	*7*
Risk of bias within individual studies	12	Describe methods used for assessing risk of bias of individual studies (including specification of whether this was done at the study or outcome level), and how this information is to be used in any data synthesis.	***6***
Summary measures	13	State the principal summary measures (e.g., risk ratio, difference in means). *Also describe the use of additional summary measures assessed, such as treatment rankings and surface under the cumulative ranking curve (SUCRA) values, as well as modified approaches used to present summary findings from meta-analyses.*	*7*
Planned methods of analysis	14	Describe the methods of handling data and combining results of studies for each network meta-analysis. This should include, but not be limited to:<br>• *Handling of multi-arm trials;*<br>• *Selection of variance structure;*<br>• *Selection of prior distributions in Bayesian analyses; and*<br>• *Assessment of model fit.*	*7*
**Assessment of Inconsistency**	**S2**	Describe the statistical methods used to evaluate the agreement of direct and indirect evidence in the treatment network(s) studied. Describe efforts taken to address its presence when found.	*7*
Risk of bias across studies	15	Specify any assessment of risk of bias that may affect the cumulative evidence (e.g., publication bias, selective reporting within studies).	***6***
Additional analyses	16	Describe methods of additional analyses if done, indicating which were pre-specified. This may include, but not be limited to, the following:<br>• Sensitivity or subgroup analyses;<br>• Meta-regression analyses;<br>• *Alternative formulations of the treatment network; and*	***8***

- *Use of alternative prior distributions for Bayesian analyses (if applicable).*

RESULTS†

Study selection	17	Give numbers of studies screened, assessed for eligibility, and included in the review, with reasons for exclusions at each stage, ideally with a flow diagram.	***8 and Fig 1***
**Presentation of network structure**	**S3**	Provide a network graph of the included studies to enable visualization of the geometry of the treatment network.	***Fig 2***
**Summary of network geometry**	**S4**	Provide a brief overview of characteristics of the treatment network. This may include commentary on the abundance of trials and randomized patients for the different interventions and pairwise comparisons in the network, gaps of evidence in the treatment network, and potential biases reflected by the network structure.	***Supp 5***
Study characteristics	18	For each study, present characteristics for which data were extracted (e.g., study size, PICOS, follow-up period) and provide the citations.	***Tab 1 and Supp 5***
Risk of bias within studies	19	Present data on risk of bias of each study and, if available, any outcome level assessment.	***Supp 6***
Results of individual studies	20	For all outcomes considered (benefits or harms), present, for each study: 1) simple summary data for each intervention group, and 2) effect estimates and confidence intervals. *Modified approaches may be needed to deal with information from larger networks.*	***10-11***
Synthesis of results	21	Present results of each meta-analysis done, including confidence/credible intervals. *In larger networks, authors may focus on comparisons versus a particular comparator (e.g. placebo or standard care), with full findings presented in an appendix. League tables and forest plots may be considered to summarize pairwise comparisons.* If additional summary measures were explored (such as treatment rankings), these should also be presented.	***Tables 2 and 3***
**Exploration for inconsistency**	**S5**	Describe results from investigations of inconsistency. This may include such information as measures of model fit to compare consistency and inconsistency models, *P* values from statistical tests, or summary of inconsistency estimates from different parts of the treatment network.	***Tables 2 & 3 and Supp 7***
Risk of bias across studies	22	Present results of any assessment of risk of bias across studies for the evidence base being studied.	***10-11***
Results of additional analyses	23	Give results of additional analyses, if done (e.g., sensitivity or subgroup analyses, meta-regression analyses*, alternative network geometries studied, alternative choice of prior distributions for Bayesian analyses,* and so forth).	***11***

**DISCUSSION**			
Summary of evidence	24	Summarize the main findings, including the strength of evidence for each main outcome; consider their relevance to key groups (e.g., healthcare providers, users, and policy-makers).	***11-12***
Limitations	25	Discuss limitations at study and outcome level (e.g., risk of bias), and at review level (e.g., incomplete retrieval of identified research, reporting bias). *Comment on the validity of the assumptions, such as transitivity and consistency. Comment on any concerns regarding network geometry (e.g., avoidance of certain comparisons).*	***12***
Conclusions	26	Provide a general interpretation of the results in the context of other evidence, and implications for future research.	***13***
**FUNDING**			
Funding	27	Describe sources of funding for the systematic review and other support (e.g., supply of data); role of funders for the systematic review. This should also include information regarding whether funding has been received from manufacturers of treatments in the network and/or whether some of the authors are content experts with professional conflicts of interest that could affect use of treatments in the network.	***17-18***

PICOS = population, intervention, comparators, outcomes, study design.
* Text in italics indicateS wording specific to reporting of network meta-analyses that has been added to guidance from the PRISMA statement.
† Authors may wish to plan for use of appendices to present all relevant information in full detail for items in this section.

SUPPLEMENT 2. SEARCH STRATGIES

Central Database of Controlled Trials (CENTRAL)

#1 *:ti,ab,kw in Trials (Word variations have been searched)
#2 SR-ADDICTN or HS-ADDICTN
#3 #1 and #2
#4 alcohol*
#5 #3 and #4
#6 MeSH descriptor: [Alcoholism] this term only
#7 MeSH descriptor: [Alcohol-Related Disorders] this term only
#8 MeSH descriptor: [Alcohol Abstinence] this term only
#9 MeSH descriptor: [Alcoholic Intoxication] this term only
#10 #6 or #7 or #8 or #9
#11 (alcohol* near/3 (abuse* or addict* or dependen* or disorder* or abstinen*)):ti,ab,kw (Word variations have been searched)
#12 (problem* near/3 (drink* or alcohol* use*)):ti,ab,kw (Word variations have been searched)
#13 (problem* next alcohol*):ti,ab,kw (Word variations have been searched)
#14 #11 or #12 or #13
#15 #10 or #14 or #5

Ovid MEDLINE Databases [Epub Ahead of Print, In-Process & Other Non-Indexed Citations, Ovid MEDLINE(R) Daily and Ovid MEDLINE(R) 1946 to Present]

1. alcohol related disorders/
2. alcoholism/
3. alcohol abstinence/
4. alcoholic intoxication/
5. (alcohol* adj3 (abuse* or addict* or dependen* or disorder* or abstinen*)).ti,ab,kf.
6. alcoholism.ti,kf.
7. (problem* adj2 (drink* or alcohol* use*)).ti,ab,kf.
8. or/1-7
9. exp Narcotic Antagonists/
10. ((Opiate or opioid) and (antagonist* or inhibitor*)).ti,ab,kf,rn.
11. Naltrexone/ or Naloxone/
12. (nalmefene or Revia or Vivitrol or naltrexon or naloxone).ti,ab,kf,rn.
13. exp Dopamine antagonists/
14. exp Antipsychotic Agents/
15. (Dopamine antagonists or (antidopaminergic and (agent* or drug* or intervention* or treatment* or pharmacotherap*))).ti,ab,kf.
16. exp Phenothiazines/
17. (Olanzapine or Zyprexa or asenapine or quetiapine or Seroquel or risperidone or Risperidal or Risperdal or ziprazidone or ziprasidone or aripiprazole or Abilify or Thorazine or Aminazine or haldol or Largactil or Chlordelazine or Chlorpromazine or Contomin or Fenactil or Propaphenin or Chlorazine or Thioridazine* or Thiozine or Tiapride or Rideril or Sonapax or Meleril or Melleril or Melleryl or Mellaril or Melleretten or Melzine or Aldazine or Zuclopenthixol or alpha Clopenthixol or Cisordinol or Flufenazin* or Fluphenazine or Lyogen or Prolixin or ecopipam or Geodon or Seroquel or Haloperidol or quinolinone or Sch39166*).mp.
18. exp Anticonvulsants/
19. ((antiepileptic* or anti epileptic* or antiseizure* or anti seizure* or anticonvulsant* or anti convulsant* or anticonvulsive or anti convulsive*) and (agent* or drug* or intervention* or treatment* or pharmacotherap*)).ti,ab,kf.

20. (ACTH or (carbamazepine or Tegretol) or clorazepate or clobazam or clonazepam or chlordiazepoxide or divalproex or sodium divalproex or sodium valproate or (valproate or Depakote) or ethosuximide or ethosuccimide or ethotoin or felbamate or fosphenytoin or (gabapentin or Neurontin) or lignocaine or lamotrigine or Levetiracetam or lidocaine or hydantoins or levetiracetam or mephobarbital or methsuximide or oxcarbazepine or paraldehyde or phenacemide or phenytoin or pregabalin or primidone or succinimide or tiagabine or (topiramate or Topamax) or (valproate or Depacon) or vigabatrin or zonisamide).mp.
21. exp Valproic Acid/
22. exp "Serotonin and Noradrenaline Reuptake Inhibitors"/
23. exp Serotonin Uptake Inhibitors/
24. exp Antidepressive Agents/
25. exp Neurotransmitter Uptake Inhibitors/
26. (antidepress* or anti depress* or MAOI* or monoamine oxidase inhibit* or ((serotonin or serotonergic or norepinephrine or noradrenaline or nor epinephrine or nor adrenaline or neurotransmitt* or dopamine*) and (uptake or reuptake or reuptake)) or noradrenerg* or antiadrenergic or anti adrenergic or SSRI* or SNRI* or TCA* or tricyclic* or tetracyclic* or heterocyclic* or psychotropic*).mp.
27. (Agomelatine or Alaproclate or Amoxapine or Amineptine or Amitriptylin* or Amitriptylinoxide or Atomoxetine or Befloxatone or Benactyzine or Binospirone or Brofaromine or (Buproprion or Amfebutamone) or Butriptyline or Caroxazone or Cianopramine or Cilobamine or Cimoxatone or Citalopram or (Chlorimipramin* or Clomipramin* or Chlomipramin* or Clomipramine) or Clonidine or Clorgyline or Clovoxamine or (CX157 or Tyrima) or Demexiptiline or Deprenyl or (Desipramine* or Pertofrane) or Desvenlafaxine or Dibenzepin or Diclofensine or Dimetacrin* or Dosulepin or Dothiepin or Doxepin or Duloxetine or Desvenlafaxine or DVS233 or Escitalopram or Etoperidone or Femoxetine or Fluotracen or Fluoxetine or Fluvoxamine or (Hyperforin or Hypericum or St John*) or Imipramin* or Iprindole or Iproniazid* or Ipsapirone or Isocarboxazid* or Levomilnacipran or Lofepramine* or (Lu AA21004 or Vortioxetine) or Lu AA24530 or (LY2216684 or Edivoxetine) or Maprotiline or Melitracen or Metapramine or Mianserin or Milnacipran or Minaprine or Mirtazapine or Moclobemide or Nefazodone or Nialamide or Nitroxazepine or Nomifensine or Norfenfluramine or Nortriptylin* or Noxiptilin* or Opipramol or Oxaflozane or Paroxetine or Prazosin or Promazine or Phenelzine or Pheniprazine or Pipofezine or Pirlindole or Pivagabine or Pizotyline or Propizepine or Protriptylin* or Quinupramine or Reboxetine or Rolipram or Scopolamine or Selegiline or Sertraline or Setiptiline or Teciptiline or Thozalinone or Tianeptin* or Toloxatone or Tranylcypromin* or Trazodone or Trimipramine or Venlafaxine or Viloxazine or Vilazodone or Viqualine or Vortioxetine or Zalospirone).mp.
28. exp Alcohol Deterrents/
29. (Metadoxine or Tetrahydrocannabinol or Zofran or Pioglitazone or Aprepitant or Mecamylamine or Dutasteride or Ghrelin or Ivermectin or Isoflavone or Kudzu or Disulfiram or Metronidazole or Acamprosate or Propranolol or Doxazosin or Ketamine or Psilocybin or Agomelatine or Ondansetron or Varenicline or PUFAs or omega* or Oxytocin or Memantine or Citicoline or Diphenhydramine or Methylphenidate or Pexacerfont or Exenatide or Carisbamate or Perampanel or Flumazenil or Progesterone).mp.
30. exp Benzodiazepines/
31. exp benzodiazepinones/
32. (Benzodiazepin* or Adinazolam or Alprazolam or Bentazepam or Bretazenil or Bromazepam or Brotizolam or Camazepam or Chlordiazepoxide or Cinolazepam or Clobazam or Clonazepam or Clorazepate or Clotiazepam or Cloxazolam or Delorazepam or Devazepide or Diazepam or Estazolam or Etizolam or Fludiazepam or Flunitrazepam or Flumazenil or Flurazepam or Flutoprazepam or Halazepam or Haloxazolam or Ketazolam or Loflazepate or Loprazolam or Lorazepam or Lormetazepam or Medazepam or Metaclazepam or Mexazolam or Midazolam or Nimetazepam or Nitrazepam or Nordazepam or Oxazepam or Oxazolam or Phenazepam or Pinazepam or Prazepam or Premazepam or Propazepam or Quazepam or Ripazepam or Serazepine or Temazepam or Tetrazepam or Tofisopam or Triazolam or Zolazepam or Zaleplon or Zolpidem or Zopiclone).mp.
33. exp gamma-Aminobutyric Acid/
34. GABA agonist*.ti,ab,kf.

35. exp GABA agonists/
36. exp GABA Uptake Inhibitors/
37. (Baclofen or GHB or gamma Hydroxybutyric acid or gamma aminobutyric acid or sodium oxybate).mp.
38. exp Glutamatergic Agents/
39. (amantadin* or atomoxetin* or dcycloserin* or dextromethorphan or GLYX 13 or "MK 0657" or (ketamin* or Ketalar or Ketaject or Ketanest) or (lanicemin* or AZD6765) or memantin* or quinolin* or rellidep or riluzol* or (tramadol* or ETS6103 or viotra) or ampa or cerc 301 or d serin* or glun2b or glutamate or glutamin* or glutamatergic or glutathione* or glycin* or mglu* or N acetyl cysteine* or N methyl D aspartate or nmda or nrx 1074 or kainite or nr2b or sarcosin* or NAC).mp.
40. Calcium Channel Blockers/
41. (calcium adj3 (antagonist* or blocker* or inhibit*)).mp.
42. (amlodipine or amrinone or azelnidipine or bencyclan* or bepridil or AT877 or cilnidipine or cinnarizine or conotoxin* or daropidine or diltiazem or efonidipine or felodipine or fendiline or flunarizine or gallopamil or isradipine or lacidopine or lidoflazine or mibefradil or nicardipine or nifedipine or nimodipine or nisoldipine or nitrendipine or perhexiline or prenylamine or verapamil or magnesium sulph*).mp.
43. (therapy or drug therapy or rehabilitation).fs.
44. exp Drug Therapy/
45. or/9-44
46. randomized controlled trial.pt.
47. (randomi#ed or randomi#ation).ab,ti,kf.
48. RCT.ab.
49. (random* adj3 (administ* or allocat* or assign* or class* or control* or determine* or divide* or distribut* or expose* or fashion* or number* or place* or recruit* or subsitut* or treat*)).ab,kf.
50. placebo.ab,ti,kf.
51. trial.ab,ti,kf.
52. ((singl* or doubl* or tripl* or trebl*) adj3 (blind* or mask* or dumm*)).mp.
53. clinical trial, phase ii/ or clinical trial, phase iii/ or clinical trial, phase iv/ or randomized controlled trial/ or pragmatic clinical trial/
54. ((waitlist* or wait* list* or treatment as usual or TAU) adj3 (control or group*)).ab.
55. (((standard or routine or usual) adj2 (care or treatment or medication or therapy)) and (control* or group*)).ab.
56. or/46-55
57. psychotherap$.ti,ab,kf.
58. (psychotherap$ or psychoeducat* or psycho educat*).ti,ab,kf.
59. (behav* adj2 (activation or therap* or treat* or intervention or modification or train*)).ti,ab,kf.

60. (CBT or (cognitive adj2 (therap* or treat* or intervention or modification or train*))).ti,ab,kf.
61. (motivational adj2 (enhancement or interview or support or skills)).ti,ab,kf.
62. mindfulness.ti,ab,kf.
63. (famil* adj2 therap*).ti,ab,kf.
64. ((couple* or spouse* or partner* or marital or marriage or conjoint or interpersonal) adj2 (therap* or counsel* or treat* or intervention*)).ti,ab,kf.
65. (psycholog* adj2 (therap* or treat* or intervention or modification or train*)).ti,ab,kf.
66. exp Psychotherapy/
67. exp Self help Groups/
68. ((self adj2 help) and group*).ti,ab,kf.
69. (twelve adj2 step).ti,ab,kf.
70. exp Rehabilitation/
71. (group adj2 (activit* or discussion* or therap* or treat* or intervention* or support or train*)).ti,ab,kf.
72. problem solving.mp.
73. (psychosoci* or psycho soci* or social support).ti,ab,kf.
74. (volunteering or activity scheduling).ti,ab,kf.

75. (community adj2 (activit* or discussion* or therap* or treat* or intervention* or support or train*)).ti,ab,kf.
76. (contingency management or incentive* or reward or rewards or voucher* or money or monetary).ti,ab,kf.
77. ((alcohol* or addict*) adj2 (therap* or treat* or intervention or management or modification or support or train*)).ti,ab,kf.
78. or/57-77
79. 8 and (45 or 78) and 56
80. (random$ adj sampl$ adj7 ("cross section$" or questionnaire$1 or survey$ or database$1)).ti,ab. not (comparative study/ or controlled study/ or randomi?ed controlled.ti,ab. or randomly assigned.ti,ab.)
81. Cross-sectional study/ not (randomized controlled trial/ or controlled clinical study/ or controlled study/ or randomi?ed controlled.ti,ab. or control group$1.ti,ab.)
82. (((case adj control$) and random$) not randomi?ed controlled).ti,ab.
83. (Systematic review not (trial or study)).ti.
84. (nonrandom$ not random$).ti,ab.
85. "Random field$".ti,ab.
86. (random cluster adj3 sampl$).ti,ab.
87. (review.ab. and review.pt.) not trial.ti.
88. "we searched".ab. and (review.ti. or review.pt.)
89. (databases adj4 searched).ab.
90. (rat or rats or mouse or mice or swine or porcine or murine or sheep or lambs or pigs or piglets or rabbit or rabbits or cat or cats or dog or dogs or cattle or bovine or monkey or monkeys or trout or marmoset$1).ti. and animal experiment/
91. Animal experiment/ not (human experiment/ or human/)
92. exp animals/ not humans.sh.
93. or/80-92
94. 79 not 93

Ovid Embase (1974 to present)

1. alcohol abuse/ or "alcohol use disorder"/
2. alcoholism/
3. alcohol abstinence/
4. alcohol withdrawal/
5. detoxification/ and alcohol*.ti.
6. (alcohol* adj3 (abuse* or addict* or dependen* or disorder* or abstinen*)).ti,ab,kw.
7. alcoholism.ti,kw.
8. (problem* adj2 (drink* or alcohol* use*)).ti,ab,kw.
9. or/1-8
10. exp Narcotic Antagonist/
11. ((Opiate or opioid) and (antagonist* or inhibitor*)).ti,ab,kw,rn.
12. Naltrexone/ or Naloxone/
13. (nalmefene or Revia or Vivitrol or naltrexon or naloxone).ti,ab,kw,rn.
14. exp Dopamine Receptor Blocking Agent/ or exp Dopamine Receptor Affecting Agent/ or exp Dopamine Uptake Inhibitor/
15. exp Neuroleptic Agent/
16. (Dopamine antagonist* or (antidopaminergic and (agent* or drug* or intervention* or treatment* or pharmacotherap*))).ti,ab,kw.
17. exp Phenothiazine/ or (methotrimeprazine or levomepromazine).mp.
18. (Olanzapine or Zyprexa or asenapine or quetiapine or Seroquel or risperidone or Risperidal or Risperdal or ziprazidone or ziprasidone or aripiprazole or Abilify or Thorazine or Aminazine or haldol or Largactil or Chlordelazine or Chlorpromazine or Contomin or Fenactil or Propaphenin or Chlorazine or Thioridazine* or Thiozine or Tiapride or Rideril or Sonapax or Meleril or Melleril or

Melleryl or Mellaril or Melleretten or Melzine or Aldazine or Zuclopenthixol or alpha Clopenthixol or Cisordinol or Flufenazin* or Fluphenazine or Lyogen or Prolixin or ecopipam or Geodon or Seroquel or Haloperidol or quinolinone or Sch39166* or amisulpride).mp.
19. exp Anticonvulsive Agent/
20. ((antiepileptic* or anti epileptic* or antiseizure* or anti seizure* or anticonvulsant* or anti convulsant* or anticonvulsive or anti convulsive*) and (agent* or drug* or intervention* or treatment* or pharmacotherap*)).ti,ab,kw.
21. (ACTH or (carbamazepine or Tegretol) or clorazepate or clobazam or clonazepam or chlordiazepoxide or divalproex or sodium divalproex or sodium valproate or (valproate or Depakote) or ethosuximide or ethosuccimide or ethotoin or felbamate or fosphenytoin or (gabapentin or Neurontin) or lignocaine or lamotrigine or Levetiracetam or lidocaine or hydantoins or levetiracetam or mephobarbital or methsuximide or oxcarbazepine or paraldehyde or phenacemide or phenytoin or pregabalin or primidone or succinimide or tiagabine or (topiramate or Topamax) or (valproate or Depacon) or vigabatrin or zonisamide).mp.
22. exp Serotonin Receptor Affecting Agent/ or exp Serotonin Noradrenalin Reuptake Inhibitor/ or exp Triple Reuptake inhibitor/
23. exp Adrenergic Receptor Affecting Agent/
24. exp Antidepressant Agent/
25. exp Neurotransmitter Uptake Inhibitors/
26. (antidepress* or anti depress* or MAOI* or monoamine oxidase inhibit* or ((serotonin or serotonergic or norepinephrine or noradrenaline or nor epinephrine or nor adrenaline or neurotransmitt* or dopamine*) and (uptake or reuptake or reuptake)) or noradrenerg* or antiadrenergic or anti adrenergic or SSRI* or SNRI* or TCA* or tricyclic* or tetracyclic* or heterocyclic* or psychotropic*).mp.
27. (Agomelatine or Alaproclate or Amoxapine or Amineptine or Amitriptylin* or Amitriptylinoxide or Atomoxetine or Befloxatone or Benactyzine or Binospirone or Brofaromine or (Buproprion or Amfebutamone) or Butriptyline or Caroxazone or Cianopramine or Cilobamine or Cimoxatone or Citalopram or (Chlorimipramin* or Clomipramin* or Chlomipramin* or Clomipramine) or Clonidine or Clorgyline or Clovoxamine or (CX157 or Tyrima) or Demexiptiline or Deprenyl or (Desipramine* or Pertofrane) or Desvenlafaxine or Dibenzepin or Diclofensine or Dimetacrin* or Dosulepin or Dothiepin or Doxepin or Duloxetine or Desvenlafaxine or DVS233 or Escitalopram or Etoperidone or Femoxetine or Fluotracen or Fluoxetine or Fluvoxamine or (Hyperforin or Hypericum or St John*) or Imipramin* or Iprindole or Iproniazid* or Ipsapirone or Isocarboxazid* or Levomilnacipran or Lofepramine* or (Lu AA21004 or Vortioxetine) or Lu AA24530 or (LY2216684 or Edivoxetine) or Maprotiline or Melitracen or Metapramine or Mianserin or Milnacipran or Minaprine or Mirtazapine or Moclobemide or Nefazodone or Nialamide or Nitroxazepine or Nomifensine or Norfenfluramine or Nortriptylin* or Noxiptilin* or Opipramol or Oxaflozane or Paroxetine or Prazosin or Promazine or Phenelzine or Pheniprazine or Pipofezine or Pirlindole or Pivagabine or Pizotyline or Propizepine or Protriptylin* or Quinupramine or Reboxetine or Rolipram or Scopolamine or Selegiline or Sertraline or Setiptiline or Teciptiline or Thozalinone or Tianeptin* or Toloxatone or Tranylcypromin* or Trazodone or Trimipramine or Venlafaxine or Viloxazine or Vilazodone or Viqualine or Vortioxetine or Zalospirone).mp.
28. exp "drugs used in the treatment of addiction"/
29. (Metadoxine or Tetrahydrocannabinol or Zofran or Pioglitazone or Aprepitant or Mecamylamine or Dutasteride or Ghrelin or Ivermectin or Isoflavone or Kudzu or Disulfiram or Metronidazole or Acamprosate or Propranolol or Doxazosin or Ketamine or Psilocybin or Agomelatine or Ondansetron or Varenicline or PUFAs or omega* or Oxytocin or Memantine or Citicoline or Diphenhydramine or Methylphenidate or Pexacerfont or Exenatide or Carisbamate or Perampanel or Flumazenil or Progesterone).mp.
30. exp Anxiolytic Agent/
31. exp Benzodiazepine/
32. exp Benzodiazepine Derivative/
33. exp Sedative Agent/ or exp Hypnotic Sedative Agent/
34. (Benzodiazepin* or Adinazolam or Alprazolam or Bentazepam or Bretazenil or Bromazepam or Brotizolam or Camazepam or Chlordiazepoxide or Cinolazepam or Clobazam or Clonazepam or

Clorazepate or Clotiazepam or Cloxazolam or Delorazepam or Devazepide or Diazepam or Estazolam or Etizolam or Fludiazepam or Flunitrazepam or Flumazenil or Flurazepam or Flutoprazepam or Halazepam or Haloxazolam or Ketazolam or Loflazepate or Loprazolam or Lorazepam or Lormetazepam or Medazepam or Metaclazepam or Mexazolam or Midazolam or Nimetazepam or Nitrazepam or Nordazepam or Oxazepam or Oxazolam or Phenazepam or Pinazepam or Prazepam or Premazepam or Propazepam or Quazepam or Ripazepam or Serazepine or Temazepam or Tetrazepam or Tofisopam or Triazolam or Zolazepam or Zaleplon or Zolpidem or Zopiclone or Meprobamate).mp.
35. exp 4 aminobutyric acid receptor stimulating agent/
36. GABA agonist*.ti,ab,kw.
37. (Baclofen or GHB or gamma Hydroxybutyric acid or gamma aminobutyric acid or sodium oxybate).mp.
38. (amantadin* or atomoxetin* or dcycloserin* or dextromethorphan or GLYX 13 or "MK 0657" or (ketamin* or Ketalar or Ketaject or Ketanest) or (lanicemin* or AZD6765) or memantin* or quinolin* or rellidep or riluzol* or (tramadol* or ETS6103 or viotra) or ampa or cerc 301 or d serin* or glun2b or glutamate or glutamin* or glutamatergic or glutathione* or glycin* or mglu* or N acetyl cysteine* or N methyl D aspartate or nmda or nrx 1074 or Org 25935 or kainite or nr2b or sarcosin* or NAC).mp.
39. exp Calcium Channel Blockers/
40. (calcium adj3 (antagonist* or blocker* or inhibit*)).mp.
41. (amlodipine or amrinone or azelnidipine or bencyclan* or bepridil or AT877 or caroverine cilnidipine or cinnarizine or conotoxin* or daropidine or diltiazem or efonidipine or felodipine or fendiline or flunarizine or gallopamil or isradipine or lacidopine or lidoflazine or mibefradil or nicardipine or nifedipine or nimodipine or nisoldipine or nitrendipine or perhexiline or prenylamine or verapamil or magnesium sulph* or magnesium sulf*).mp.
42. *Drug Therapy/
43. Psychopharmacology/ or Psychopharmacotherapy/ or Psychotropic Agent/
44. or/10-43
45. randomized controlled trial/
46. (randomi#ed or randomi#ation).ab,ti,kw.
47. RCT.ab.
48. (random* adj3 (administ* or allocat* or assign* or class* or control* or determine* or divide* or distribut* or expose* or fashion* or number* or place* or recruit* or subsitut* or treat*)).ab,kw.
49. placebo.ab,ti,kw.
50. trial.ab,ti,kw.
51. ((singl* or doubl* or tripl* or trebl*) adj3 (blind* or mask* or dumm*)).mp.
52. phase 1 clinical trial/ or phase 2 clinical trial/ or phase 3 clinical trial/ or phase 4 clinical trial/
53. or/45-52
54. *Therapy/
55. exp Psychiatric Treatment/
56. exp Counseling/
57. (psychotherap$ or psychoeducat* or psycho educat*).ti,ab,kw.
58. (behav* adj2 (activation or therap* or treat* or intervention or modification or train*)).ti,ab,kw.
59. (CBT or (cognitive adj2 (therap* or treat* or intervention or modification or train*))).ti,ab,kw.
60. (motivational adj2 (enhancement or interview or support or skills)).ti,ab,kw.
61. mindfulness.ti,ab,kw.
62. (famil* adj2 therap*).ti,ab,kw.
63. ((couple* or spouse* or partner* or marital or marriage or conjoint or interpersonal) adj2 (therap* or counsel* or treat* or intervention*)).ti,ab,kw.
64. (psycholog* adj2 (therap* or treat* or intervention or modification or train*)).ti,ab,kw.
65. exp Self Help/
66. ((self adj2 help) and group*).ti,ab,kw.
67. (twelve adj2 step).ti,ab,kw.
68. exp Rehabilitation/

69. (group adj2 (activit* or discussion* or therap* or treat* or intervention* or support or train*)).ti,ab,kw.
70. problem solving.mp.
71. exp Social Care/ or Psychosocial Care/
72. (psychosoci* or psycho soci* or social support).ti,ab,kw.
73. (volunteering or activity scheduling).ti,ab,kw.
74. (community adj2 (activit* or discussion* or therap* or treat* or intervention* or support or train*)).ti,ab,kw.
75. (contingency management or incentive* or reward or rewards or voucher* or money or monetary).ti,ab,kw.
76. ((alcohol* or addict*) adj2 (therap* or treat* or intervention or management or modification or support or train*)).ti,ab,kw.
77. or/54-76
78. (random$ adj sampl$ adj7 ("cross section$" or questionnaire$1 or survey$ or database$1)).ti,ab. not (comparative study/ or controlled study/ or randomi?ed controlled.ti,ab. or randomly assigned.ti,ab.)
79. Cross-sectional study/ not (randomized controlled trial/ or controlled clinical study/ or controlled study/ or randomi?ed controlled.ti,ab. or control group$1.ti,ab.)
80. (((case adj control$) and random$) not randomi?ed controlled).ti,ab.
81. (Systematic review not (trial or study)).ti.
82. (nonrandom$ not random$).ti,ab.
83. "Random field$".ti,ab.
84. (random cluster adj3 sampl$).ti,ab.
85. (review.ab. and review.pt.) not trial.ti.
86. "we searched".ab. and (review.ti. or review.pt.)
87. "update review".ab.
88. (databases adj4 searched).ab.
89. (rat or rats or mouse or mice or swine or porcine or murine or sheep or lambs or pigs or piglets or rabbit or rabbits or cat or cats or dog or dogs or cattle or bovine or monkey or monkeys or trout or marmoset$1).ti. and animal experiment/
90. Animal experiment/ not (human experiment/ or human/)
91. or/78-90
92. 9 and (44 or 77) and 53
93. 92 not 91

Ovid PsycINFO

1. alcohol abuse/
2. Alcoholism/
3. exp Alcohol Intoxication/
4. alcohol withdrawal/
5. alcohol*.ti,id. and ("substance abuse and addiction measures"/ or detoxification/)
6. (alcohol* adj3 (abuse* or addict* or dependen* or disorder* or abstinen*)).ti,ab,id.
7. (problem* adj2 (drink* or alcohol* use*)).ti,ab,id.
8. or/1-7
9. (treatment-as-usual or (treatment* adj2 usual) or (standard adj2 care) or (standard adj2 treatment) or (routine adj2 care) or (usual adj2 medication*) or (usual adj2 care) or TAU).ti,ab,id.
10. (waitlist* or wait-list* or waiting-list* or wait* list* or (waiting adj (condition or control)) or WLC).ti,ab,id.
11. (((delay* adj3 (start or treatment*)) or no intervention or no treatment* or no-treatment or non treatment* or nontreatment* or non-treatment or minim* treatment* or untreated group* or untreated control* or without any treatment) and (control* or group*)).ti,ab,id.
12. ((no intervention* or non intervention* or non-intervention* or without any intervention*) and (control* or group*)).ti,ab,id.

13. (receiv* nothing or "did not receive" or standard control or control group).ti,ab,id.
14. (("no therap*" or "no psychotherap**" or "non therap*" or nontherap* or nonpsychotherap* or "non psychotherap*" or "minim* therap*" or "minim* psychotherap*" or "no contact" or pseudotherap* or "pseudo therap*" or "pseudo psychotherap*" or "therap* as usual" or "usual therap*") and (control* or group*)).ti,ab,id.
15. (reference group or observation group or control group).ti,ab,id.
16. ((convention* treatment or conventional therap* or standard treatment* or standard therap*) and (control* or group*)).ti,ab,id.
17. treatment effectiveness evaluation.sh.
18. mental health program evaluation.sh.
19. placebo.sh.
20. randomi#ed.ti,ab.
21. ((singl* or doubl* or trebl* or tripl*) adj3 (blind* or mask* or dummy)).mp.
22. (random* adj3 (administ* or class* or control* or determine* or divide* or distribut* or expose* or fashion or number* or place* or recruit* or subsitut* or treat*)).ab.
23. trial.ti,ab.
24. or/9-23
25. 8 and 24

ClinicalTrials.gov

Advanced search: Interventional studies AND
1. Condition= alcoholism n=40
2. Condition= (addiction AND alcohol) n=11
3. Condition= “alcohol dependence” OR “alcohol dependency” OR “alcohol use disorder” OR “alcohol use disorders” OR “alcohol related disorder” OR “alcohol related disorders” n=107
4. alcohol:ti and keywords: (abstinence OR abstinent OR abstain) n=21
5. alcohol:ti and keywords: (detox OR detoxification OR detoxified) n=5

WHO International Clinical Trials Registry Platform (ICTRP)

alcohol use disorder OR chronic alcoholic intoxication OR alcohol dependence OR alcohol dependency OR alcohol use disorders

Supplement 3. List of excluded interventions

ID	AUTHOR/YEAR	INTERVENTION	REASONS
**11778**	Ashrafioun 2009[1]	1. Usual care<br>2. Usual care + motivational interview<br>3. Usual care + twelve step facilitation message	Intervention started while hospitalisation.
**520**	Bejczy 2014[2]	1. glycine transporter-1 Org 25935<br>2. Placebo	Not marketed in the world
**14425**	Blake 1967[3]	1. Electrical aversion therapy<br>2. Relaxation aversion	Aversion therapy
**17369**	Buchholz 2020[4]	1. Intervention group: MATE-interview, followed by level of care (LOC)-recommendation with multidisciplinary team<br>2. Control group: MATE-interview without LOC-recommendation (normal follow-up)	Interventions were conducted during the inpatient settings
**14707**	Cannon 1981[5]	1. Emetic aversion conditioning group<br>2. Shock aversion conditioning group<br>3. control	Aversion therapy
**14495**	Driessen 2001[6]	1. 3-week in-patient motivational treatment programme	Inpatient setting.
**14380**	Fleiger 1973[7]	1. Covert sensitization – convert or imagined stimuli for both the conditioned stimulus and the unconditioned stimulus<br>2. Control	Aversion therapy. Inpatient setting.
**3497**	Klauss 2014[8]	1. Transcranial direct current stimulation (tDCS)<br>2. Sham-tDCS group	Medical device
**14452**	Madill 1966[9]	1. Aversion therapy by succinylcholine-induced paralysis (alcohol or bottle was given during paralysis)<br>2. Pseudo-conditioning group (with succinylcholine given but without alcohol or bottle given during paralysis)<br>3. Placebo	Aversion therapy by succinylcholine-induced paralysis
**4328, 4322**	Martinotti 2010[10 11]	1. Acetyl-L-Carnitine (ALC) at a dosage of 3 g/day by slow IV infusion (500 ml of solution infused in 3–4 h) for 10 days and then 3 g three times a day orally for the remainder of the study<br>2. ALC 1 g/day by slow IV infusion for 10 days and then 3 g three times a day orally for the remainder of the study<br>3. Placebo	Require infusion and frequent follow-ups

**14460**	O'Connell 1988[12]	1. Relapse Prevention (rehabilitation programme)<br>2. Social skill training (rehabilitation programme)<br>3. Cognitive reframing (rehabilitation programme)<br>4. Meditation training (rehabilitation programme)<br>5. Control	Not intervention of interests. Inpatient setting.
**14464**	Regester 1971[13]	1. Aversion therapy by giving electric shock	Aversion therapy
**6430**	Soyka 2008[14]	1. Cannabinoid receptor 1 blocker rimonabant (SR 141716)<br>2. Placebo	Not marketed in the world
**14785**	Steffen 1975[15]	1. Feedback-assisted Relaxation training, which took place in the Rutgers Alcohol Behavior Research Laboratory, was accomplished by the Bio-Electric Information Feedback System (see Steffen, Nathan, & Taylor, 1974, for a further description of setting and apparatus)<br>2. Attention placebo	Laboratory setting using electromyographically induced relaxation
**14472**	Vogler 1975[16]	1. Aversion therapy with electrical shock<br>2. Control	Aversion therapy with electrical shock
**14474**	West 1979[17]	1. Rehabilitation program<br>2. Waiting list	Rehabilitation program
**14476**	WHO 1992[18]	1. Control<br>2. Simple advice<br>3. Brief counselling	Brief intervention for hazardous alcohol use.
**7227, 7226**	Wiesbeck 1999[19 20]	1. Ritanserin<br>2. Placebo	Ritanserin is not marketed for clinical use

References

1. Ashrafioun LB, R. D.; Frydrych, L. M.; Homish, G. G.; Foschio, E. M.; Bashaw, H. L. A randomized trial of two behavioral interventions to improve outcomes following inpatient detoxfication for alcohol dependence [Conference]. *Alcoholism, clinical and experimental research* 2009;Conference: Texas Research Society On Alcoholism - 19th Annual Scientific Meeting. San Antonio, TX United States. Conference Start: 20090220. Conference End: 20090220. Conference Publication:(var.pagings):47A. doi: http://dx.doi.org/10.1111/j.1530-0277.2009.00957.x
2. Bejczy AN, K. R.; Szegedi, A.; Schoemaker, J.; Ruwe, F.; Soderpalm, B. Efficacy and safety of the glycine transporter-1 inhibitor org 25935 for the prevention of relapse in alcohol-dependent patients: a randomized, double-blind, placebo-controlled trial. *Alcoholism, clinical and experimental research* 2014;38(9):2427-35.

3. Blake BG. A FOLLOW-UP OF ALCOHOLICS TREATED BY BEHAVIOUR THERAPY. *Behaviour Research and Therapy* 1967;5(2):89-94. doi: 10.1016/0005-7967(67)90002-2
4. Buchholz AD, J.; Rosahl, A.; Hempleman, J.; Konig, H. H.; Konnopka, A.; Kraus, L.; Kriston, L.; Piontek, D.; Reimer, J.; Rohrig, J.; Scherbaum, N.; Silkens, A.; Berner, M. Patient-Centered Placement Matching of Alcohol-Dependent Patients Based on a Standardized Intake Assessment: Primary Outcomes of an Exploratory Randomized Controlled Trial. *European Addiction Research* 2020:1-13. doi: 10.1159/000505913 [published Online First: 2020/02/20]
5. Cannon DSB, T. B. Emetic and electric shock alcohol aversion therapy: assessment of conditioning. *Journal of consulting and clinical psychology* 1981;49(1):20-33.
6. Driessen MM, S.; Hill, A.; Wetterling, T.; Lange, W.; Junghanns, K. The course of anxiety, depression and drinking behaviours after completed detoxification in alcoholics with and without comorbid anxiety and depressive disorders. *Alcohol and Alcoholism* 2001;36(3):249-55. doi: 10.1093/alcalc/36.3.249
7. Fleiger DLZ, H. W. Covert sensitization treatment with alcoholics. *Canadian Counsellor* 1973;7(4):269-77.
8. Klauss JPP, L. C.; Silva Merlo, B. L.; Almeida Correia Santos, G.; Fregni, F.; Nitsche, M. A.; Miyuki Nakamura-Palacios, E. A randomized controlled trial of targeted prefrontal cortex modulation with tDCS in patients with alcohol dependence. *International journal of neuropsychopharmacology* 2014;17(11):1793-803.
9. Madill MFC, D.; Laverty, S. G.; Sanderson, R. E.; Vandewater, S. L. AVERSION TREATMENT OF ALCOHOLICS BY SUCCINYLCHOLINE-INDUCED APNEIC PARALYSIS - ANALYSIS OF EARLY CHANGES IN DRINKING BEHAVIOR. *Quarterly Journal of Studies on Alcohol* 1966;27(3):483-509.
10. Martinotti GR, D.; Nicola, M.; Andreoli, S.; Tedeschi, D.; Ortolani, I.; Pozzi, G.; Iannoni, E.; D'Iddio, S.; Janiri, L. Acetyl-L-carnitine for alcohol craving and relapse prevention in anhedonic alcoholics: a randomized, double-blind, placebo-controlled pilot trial. *Alcohol and alcoholism (Oxford, Oxfordshire)* 2010;45(5):449-55.
11. Martinotti GA, S.; Reina, D.; Nicola, M.; Ortolani, I.; Tedeschi, D.; Fanella, F.; Pozzi, G.; Iannoni, E.; D'Iddio, S.; Prof, L. J. Acetyl-l-Carnitine in the treatment of anhedonia, melancholic and negative symptoms in alcohol dependent subjects [Conference]. *Progress in neuro-psychopharmacology & biological psychiatry* 2011;35(4):953-58.
12. O'Connell JM. Effectiveness of an alcohol relapse prevention program [doctoral dissertation, Fordham University, New York]. *Dissertation Abstracts International* 1988;48(9-A):2245.
13. Regester DC. Changes in autonomic responsivity and drinking behavior of alcoholics as a function of aversion therapy. *Dissertation Abstracts International* 1971;32((2-B)):1225.
14. Soyka MK, G.; Schmidt, P.; Lesch, O. M.; Leweke, M.; Fehr, C.; Gann, H.; Mann, K. F. Cannabinoid receptor 1 blocker rimonabant (SR 141716) for treatment of alcohol dependence: results from a placebo-controlled, double-blind trial. *Journal of clinical psychopharmacology* 2008;28(3):317-24.
15. Steffen JJ. Electromyographically induced relaxation in the treatment of chronic alcohol abuse. *Journal of consulting and clinical psychology* 1975;43(2):275.
16. Vogler REF, R.; Kraemer, S.; Brengelmann, J. C. Electrical aversive conditioning of alcoholics: One year follow-up. *Journal of Behavior Therapy and Experimental Psychiatry* 1975;6:171-73.

17. West PT. Three modes of training alcoholics in interpersonal communications skills: A comparative study [doctoral dissertation, University of Western Ontario]. 1979
18. World Health Organisation. Project on identification and management of alcohol-related problems. Report on phase II: A randomized clinical trial of brief interventions in primary health care. *Program on Substance Abuse* 1992
19. Wiesbeck GAW, H. G.; Chick, J.; Naranjo, C. A.; Boening, J.; Beckmann, H. Ritanserin in relapse prevention in abstinent alcoholics: results from a placebo-controlled double-blind international multicenter trial. Ritanserin in Alcoholism Work Group. *Alcoholism, clinical and experimental research* 1999;23(2):230-35.
20. Wiesbeck GAW, H. G.; Chick, J.; Boening, J. The effects of ritanserin on mood, sleep, vigilance, clinical impression, and social functioning in alcohol-dependent individuals. Ritanserin in Alcoholism Work Group. *Alcohol and alcoholism (Oxford, Oxfordshire)* 2000;35(4):384-89.

SUPPLEMENT 4. CRITERIA OF GRADE ASSESSMENT BY CINEMA

Judgement	Criteria	Instruction for downgrading
Within-study bias	Within-study bias was evaluated by majority of risk of bias assessment results within each comparison (refer to S6).<br><br>We increased the concern one level for comparisons with single study only.	• Major concerns: downgrade the evidence one level<br>• Some concerns: downgrade the evidence one level with 2 or more some concerns in other judgements
Reporting bias	Reporting bias was evaluated by non-statistical consideration of likelihood of non-publication of evidence.	We selected "suspected" among all comparisons but did not downgrade the confidence by this judgement.
Indirectness	As outcome (continuous abstinence) has consistent, clear definition, indirectness was only evaluated by majority of populations within each comparison.<br><br>Populations among studies were assessed by distributions of age, gender and comorbidities (refer to S4)	• Major concerns: downgrade the evidence one level<br>• Some concerns: downgrade the evidence one level with 2 or more some concerns in other judgements
Imprecision	Imprecision was focused on width of confidence interval (CI) based on a clinically important odds ratio of 1.2 for abstinence and 0.8 for dropouts.<br><br>We increased the concern one level if the width of CI is between 4 times and 10 times of lower limit. The concern level was increase two levels if the width of CI is above 10 times of lower limit.	• Major concerns: downgrade the evidence one level<br>• Some concerns: downgrade the evidence one level with 2 or more some concerns in other judgements
Heterogeneity	Heterogeneity was evaluated according to the CINeMA documentation by variability of effects in relation to the clinically important size of effect and between-study variance for the network meta-analysis.<br><br>We increased the concern one level if there is no information regarding between-study heterogeneity for each direct comparison or $I^2 >$ 60% in the direct comparison.	• Major concerns: downgrade the evidence one level<br>• Some concerns: downgrade the evidence one level with 2 or more some concerns in other judgements
Incoherence	Incoherence was evaluated by the design-by-treatment intervention model globally and side-splitting approach locally.	• Major concerns: downgrade the evidence one level<br>• Some concerns: downgrade the evidence one level with 2 or more some concerns in other judgements
Quality of the evidence (GRADE):<br><br>**High quality:** Further research is very unlikely to change our confidence in the estimate of effect.<br>**Moderate quality:** Further research is likely to have an important impact on our confidence in the estimate of effect and may change the estimate.<br>**Low quality**: Further research is very likely to have an important impact on our confidence in the estimate of effect and is likely to change the estimate.<br>**Very low quality:** We are very uncertain about the estimate.		

SUPPLEMENT 5. CHARACTERISTICS OF THE INCLUDED STUDIES

Trial; Country<br><br>Funding;<br>Reference;<br>Enrolment date;<br>Registry	Design<br><br>Recruitment<br><br>N of patients; Mean age (year); %Female	Alcohol dependence diagnostic criteria<br><br>Detoxification method	Intervention groups<br><br>Treatment duration/follow-up timepoints	Supportive treatment (all groups)	Severity of alcohol dependence; Baseline consumption; Previous treatment	Comorbidities; Substance use	Socioeconomic status	Main results: Abstinence (n/N)<br><br>Dropouts (n/N)
**Angelone 1998** [1]<br>Italy	RCT, single-blind<br><br>Alcohol Related Disorders Unit<br><br>N = 73; 48.8 (SD 10.1); 32%	DSM-IV<br><br>In-patient detoxification via chlordesmethyldiazepam IV and supplied with glutathione, 5-adenosylmethionine, thiamine, and electrolytes (Mean: 10days)	1. No pharmacological treatment (TAU) (N = 23)<br>2. Fluvoxamine: 150 mg/day Oral (N = 25)<br>3. Citalopram: 20 mg/day Oral (N = 33)<br><br>16 weeks	Cognitive-behavioural group therapy, daily for 8 weeks after detoxification, then weekly	MAST: 33.0 (SD 9.6)<br><br>History of withdrawal: 50.7%<br>Duration of AD (month): 129.1 (SD 95.0)	NR	NR	TAU: 7/23<br>FLX: 14/25<br>CIT: 17/33<br><br>TAU: 0/23<br>FLX: 3/25<br>CIT: 5/33
**BACLAD** [2]<br>Germany<br><br>Supported by German Research Foundation	RCT, double-blind<br><br>The outpatient unit of UniversitätsmedizinBerlin<br><br>N = 56; 46.5 (SD 7.0); 30.3%	DSM-IV-TR&ICD-10<br><br>detoxification	1. Placebo (N = 28)<br>2. Baclofen: 15 mg/day initially, increased to 270 mg/day, and then tapering down (N = 28)<br><br>24 weeks/28 weeks	Medical Management as described by Pettinati et al 2004), which focuses on psychoeducation and enhancement of motivation and adherence.	Years of hazardous alcohol consumption: 12.7 (SD 8.8)<br><br>Alcohol consumption (g/day) before inclusion: 198.9 (SD 93.9)<br><br>ADS: 16.2 (5.6)<br><br>N of previous detoxifications:<br>One: 32.1%<br>2-5: 50.0%<br>More than 5: 17.9%	Smoker: 62.5%	Married: 28.6% (PLA: 11% vs GHB: 5%)<br>Employed: 57.1%<br>Education (above secondary): 84.6%<br><br>Family history of alcoholism: 60.7%	PLA: 3/28<br>BAC: 10/28<br><br>BAC: 12/28<br>PLA: 13/28
**Baltieri 2003** [3,4]<br>Brazil<br><br>Dec 2001 to Feb 2002	RCT, double-blind<br><br>Clinical Hospital of the University of São Paulo<br><br>N = 75; 44.2 (SD 8.3); 0%	ICD-10<br><br>1-week detoxification	1. Placebo (N = 35)<br>2. Acamprosate: 1998 mg/day Oral (N = 40)<br><br>12 weeks/24 weeks	Usual procedures of GREA (behavioural orientation, clinical assessment and incentive to the participation in the group of AA)	Alcohol intake (g/day): 360.0 (SD 150.0)	NR	NR	PLA: 7/35<br>ACP: 17/40<br><br>PLA: 7/35<br>ACP: 10/40
**Baltieri 2008** [5,6]<br>Brazil<br><br>2005 to 2007	RCT, double-blind<br><br>Clinical Hospital of the University of São Paulo<br><br>N = 155; 44.3 (SD 8.4); 0%	ICD-10<br><br>1-week outpatient detoxification via lorazepam and Vitamin B1	1. Placebo (N = 54)<br>2. Naltrexone: 50 mg/day Oral (N = 49)<br>3. Topiramate: 25 mg/day initially, increased to 300mg/day Oral (N = 52)<br><br>12 weeks	Relapse prevention counselling<br><br>AA (encouraged)	SADD: 29.0 (SD 8.5)<br>OCDS: 49.8 (SD 13.2)<br><br>Quantity of alcohol used (g/day): 301 (SD 174)<br>Time since alcohol-related problems occurred (year): 9.7 (SD 10.0)	Cigarettes per day: 16.6 (SD 12.2)<br><br>HAM-D: 10.3 (SD 6.9)	Married: 51%<br>Non-White: 29%<br>High school graduate and above: 47.8%<br><br>Family history of alcoholism: 81.3%	PLA: 15/54<br>NTX: 14/49<br>TPM: 24/52<br><br>PLA: 31/54<br>NTX: 20/49<br>TPM: 19/52
**Barrias 1997** [7]<br>Portugal	RCT, double-blind<br><br>9 Centres<br><br>N = 302; 40.4; 7.9%	DSM-III<br><br>Detoxification and at least 5-day abstinence	1. Placebo (N = 152)<br>2. Acamprosate: BW≥ 60 kgs: 1998 mg/day; BW < 60 kgs: 1332 mg/day Oral (N = 150)<br><br>12 months/18 months	NR	MAST: 32.0<br>Impulse (craving): 65.6<br><br>Quantity of alcohol:<br>< 5 drinks/day: 4%<br>6-10 drinks/day: 31.1%<br>> 10 drinks/day: 64.9%<br><br>Frequency:<br>1-2 d/week: 2%<br>3-6 d/week: 9.6%<br>Daily: 88.4%	Depression (HDS): 19.4	NR	PLA: 31/152<br>ACP: 52/150<br><br>PLA: 69/152<br>ACP: 64/150
**Bender 2007** [8]<br>Germany<br><br>Supported by Sanofi-Aventis (Berlin, Germany)	RCT, double-blind<br><br>Multicentre<br><br>N = 299; 42.0 (SD 8.6); 26.8%	ICD-10<br><br>Detoxification and abstinence for at least 7 days	1. Placebo (N = 150)<br>2. Tiapride: up to 600 mg/day depending on post-withdrawal symptoms in the first month, then reduced to 300 mg/day for the rest 5 months Oral (N = 149)<br><br>24 weeks	Usual psychosocial alcohol treatment programme depending on the centre	N of previous alcohol-specific treatments: 3 (SD 6.5)<br><br>Duration of regular alcohol consumption (year): 17.5 (SD 9.5)<br>Amount of daily alcohol consumption during last drinking period (mL/day): 258.5 (SD 163.7)	NR	Permanent relationship (married): 75%<br>Employed: 54.5%<br>High school graduate and above*: 83%<br><br>*Sum of completion of apprenticeship, vocational school and university	PLA: 54/150<br>TPD: 37/149<br><br>PLA: 35/150<br>TPD: 31/149

**Besson 1998** [9]<br>Switzerland<br><br>Supported in part by state funds and by a grant from Lipha, Inc.	Randomised, double-blind<br><br>Three psychiatric clinics<br><br>N = 110; 42.5; 20%	DSM-III (chronic or episodic alcohol dependence)<br><br>Acute withdrawal treatment and a minimum of 5 days of abstinence	1. Placebo (N = 55)<br>2. Acamprosate: 1332mg-1998 mg/day, adjusted by weight. BW ≥ 60kg, 1998 mg/day; BW < 60 kg, 1332 mg/day. Oral (N = 55)<br><br>Twenty-two patients (40%) in the placebo group and 24 patients (43.6%) in the acamprosate group received concomitant disulfiram.<br><br>360 days/720 days	Short sessions (15 to 20 min) of psychosocial assessment and support approximately twice a month.<br><br>Social service (when necessary)	MAST: 31.6<br>VAS: 39.9<br><br>Duration of illness (year): 15.0<br><br>Previous use of disulfiram: 50%<br>Previous detoxification: 61%<br>Previous AA: 22%<br>Previous psychotherapy: 50%	Thioridazine (anxiolytic): 30%<br>HAM-A: 28.7<br><br>Dibenzepin (antidepressants): 11.8%<br>HAM-D: 42.2	Family history: 53.6%	PLA: 3/55<br>ACP: 14/55<br><br>PLA: 36/55<br>ACP: 36/55
**Burtscheidt 2001** [10-12]<br>Germany<br><br>Supported by the German Federal Ministry of Education and Research	RCT, open-label<br><br>Department of Psychiatry at the Heinrich-Heine-University Duesseldorf<br><br>N = 120; 42.4 (SD 7.4); 30%	DSM III-R/ICD 10<br><br>In-patient detoxification treatment	1. Standard therapy (TAU): facilitating the contact to self-help groups and extramural treatment facilities, organizing weekly meetings of former patients and offering counselling and crisis intervention on demand over 26 sessions and 6 months (N = 40)<br>2. CBT: weekly behavioural group therapy (max of six patients) lasted 100 minutes over 26 sessions and 6 months as described by Beck et al. (N =40)<br>3. CST: weekly behavioural group therapy (max of six patients) lasted 100 minutes over 26 sessions and 6 months modified from Monti et al. (N = 40)<br><br>6 months/12, 18, 24 and 30 months	NR	Age of regular alcohol consumption (year): 26.3 (SD 8)<br>Previous inpatient detoxification in the last 5 years: 46.7%	NR	Married: 36%<br>High school graduate and above: 45%<br>Employed: 38%	TAU: 11/40<br>CBT: 11/40<br>CST: 8/40<br><br>TAU: 4/40<br>CBT: 6/40<br>CST: 8/40
**Caputo 2003** [13]<br>Italy<br><br>Supported by internal funds	RCT, open-label<br><br>NR<br><br>N = 35; 48.8 (SD 9.1); 51.4%	DSM-IV<br><br>Detoxification	1. GHB: 50 mg/kg TID Oral (N = 18)<br>2. Naltrexone: 50 mg/day Oral (N = 17)<br><br>3 months	Weekly counselling sessions<br><br>AA and social services	Duration of alcohol addiction (year): 14.6 (SD 9.4)<br>Alcohol craving scale: 9.0 (SD 2.2)<br><br>Degree of alcohol dependence according to DSM-IV criteria (%):<br>Mild (2-3 items of criteria): 11.5%<br>Moderate (4-5 items of criteria): 28.6%<br>Severe (6 or more items of criteria): 60.0%	NR	Married: 51.5%<br>High school graduate and above: 37.2%<br>Employed: 65.7%	GHB: 12/18<br>NTX: 6/17<br><br>GHB: 4/18<br>NTX: 4/17
**Caputo 2007** [14]<br>Italy<br><br>Supported by internal funds	RCT, open-label<br><br>Multicentre in Italy<br><br>N = 55; 48.0 (SD 10.5); 0%	DSM-IV-TR<br><br>Detoxification	1. GHB: 50 mg/kg TID Oral (N = 20)<br>2. Naltrexone: 50 mg/day Oral (N = 17)<br>3. Combined: GHB 50mg/kg TID + NTX 50 mg/day Oral (N = 18)<br><br>3 months	AA and social services	Duration of alcohol addiction (year): 15.5 (SD 10.7)<br><br>Degree of alcohol dependence according to DSM-IV-TR criteria (%):<br>Mild (2-3 items of criteria): 2.0%<br>Moderate (4-5 items of criteria): 17.2%<br>Severe (6 or more items of criteria): 79.8%	NR	Married: 34.1%<br>High school graduate and above: 38.4%<br>Employed: 49.9%	GHB: 8/20<br>NTX: 1/17<br>GHB+NTX: 13/18<br><br>GHB: 2/20<br>NTX: 4/17<br>GHB+NTX: 3/18
**Chick 2000** [15]<br>UK<br><br>1991-1993<br>Funded by Lipha Pharmaceuticals	RCT, open-label<br><br>20 centres across the UK<br><br>N = 581; 43.3; 16.5%	DSM-III<br><br>In-or out-patient detoxification by chlordizepoxide or no drugs (defined as at least 5 days of abstinence)	1. Placebo (N = 292)<br>2. Acamprosate: 1998 mg/day Oral (N = 289)<br><br>24 weeks/28 weeks	Usual psychosocial out-patient treatment programme	MAST: 37.5<br>SADQ: 33.5<br>CAGE (a score of 4): 75%<br>Craving at baseline: 23.0 mm<br><br>Alcohol consumption (units/week): 178.1	NR	Unmarried: 44.0%<br>Unemployed: 48.5%	PLA: 32/292<br>ACP: 35/289<br><br>PLA: 189/292<br>ACP: 189/289
**Chick 2004** [16-19]<br>United Kingdom, Eire, Austria and Switzerland<br><br>Funded by Solvay–Duphar	RCT, double-blind<br><br>Multicentre across Europe<br><br>N = 521; 42.0 (SD 9.8); 25.1%	DSM-III-R<br><br>Detoxification and abstinent for 10–30 days	1. Placebo (N = 249*)<br>2. Fluvoxamine: 100-300 mg/day Oral (N = 243*)<br><br>1 year<br><br>*ITT sample used in the trial	Outpatient psychosocial, varied between centres<br><br>AA (advised)	SADQ: 32.1 (SD 13.6)<br>Age at start of regular drinking (year): 21.7 (SD 7.9)<br>Age at start of problem drinking (year): 31.4 (SD 10.3)<br>Typical week's recent heavy drinking (unit): 178.0 (SD 117.5)<br>N of days drank in typical week: 6.2 (SD 1.5)<br><br>DSM-III-R (severity of dependence, %):<br>Mild: 5%<br>Moderate: 30.5%<br>Severe: 65.0%	NR	NR	PLA: 72/249<br>FLX: 70/243<br><br>PLA: 133/249<br>FLX: 171/243

**Cornelius 1997** [20-22]<br>USA<br><br>Supported by NIAAA and MHCRC	RCT, double-blind<br><br>The Western Psychiatric Institute and Clinic of the University of Pittsburgh<br><br>N = 51; 34.8 (SD 10.2); 49.0%	DSM-III-R<br><br>2-3 days of detoxification with minor tranquilizers	1. Placebo (N = 26)<br>2. Fluoxetine: 20 mg/day. Max 40 mg/day Oral (N = 25)<br><br>12 weeks/1 year	Weekly supportive psychotherapy sessions and weekly meetings with an psychiatrist<br><br>AA	N of DSM-III-R criteria, AD: 5.7 (1.7)<br>N of DSM-III-R criteria, Major depression: 6.8 (1.1)<br><br>N of days drinking in past 90 days: 49.8 (SD 29.1)<br>N of days drinking to drunkenness in past 90 days: 36.0 (SD 27.1)	Marijuana use: 22<br><br>HAM-D-24 before detox: 33.1<br>HAM-D-24 after detox: 18.6 (SD 8.1)<br>BDI before detox: 27.3 (SD 12.5)<br>BDI after detox: 15.9 (SD 11.3)	Married: 11.7%<br>Non-white: 52.9%<br>Employed: 31.4%	PLA: 4/26<br>FLT: 7/25<br><br>PLA: 10/26<br>FLT: 10/25
**Coriale 2019** [23]<br>Italy<br><br>Supported by the Italian Health Ministry-National Fund to fighting drugs, 4116 (ex1686)	RCT<br><br>"Latium Region Alcohol Referral Center"<br>of Policlinico Umberto I, Sapienza University Hospital<br><br>N = 90; 47.1 (SD 9.8); 30%	DSM-V<br><br>6-10 days detoxification with diazepam	1. Short-form CBT: 5-session intervention, each session lasted 60mins (N = 43)<br>2. MET: 3-session of client-centred intervention, each session lasted 60 mins (N = 47)<br><br>3 months/365 days	Medical follow up every month; psychological follow up at third, sixth and twelfth months<br><br>Patients received Naltrexone (31.4%), Nalmefene (21.2%) and Acamprosate (47.43%) for alcohol treatment	Drinks per day: 13.7 (10.3)<br><br>Age of onset: 29.9 (11.3)<br>Year of consumption: 15.6 (10.1)	Smoking (cigs/day): 17.8<br><br>MMSE: 16.0 (SD 1.36)<br>SCL-90 (Depression): 0.64 (SD 0.44)<br>SCL-90 (Anxiety): 0.53 (SD 0.38)<br>SCL-90 (GSI): 0.52 (SD 0.29)	Educational level (1 Low 4 Top): 2.73 (0.25)	sCBT: 0/43<br>MET: 4/47<br><br>sCBT: 15/43<br>MET: 43/47
**Croissant 2006** [24]<br>Germany	RCT, open-label<br><br>NR<br><br>N = 30; 45.7 (SD 7.3); 26.7%	ICD-10 and DSM-IV<br><br>In- or out-patient detoxification and 1-week abstinence	1. Acamprosate: 1998 mg/day Oral (N = 15)<br>2. Oxcarbazepine: 150 mg/day initially, increased to 1200 mg/day Oral (N = 15)<br><br>3 months/6 months	NR	N of ICD 10 criteria: 4.7 (SD 1.5)<br>DSM-IV criteria: 5.6 (SD 1.8)<br>Duration of AD: 11.6 (8.4)<br>ADS: 15.2 (SD 8.0)<br>Ethanol consumption (drinks/day): 12.2 (SD 10.2)<br>Drinks per drinking day: 17.7 (SD 13.7)<br>N of alcoholism inpatient treatments: 3.1 (SD 2.5)<br>History of anticraving medication: 1.5 (SD 10.4)<br>OCDS-G: 16.2 (SD 7.2)	Nicotine dependence (%): 63.3%<br>FTND: 3.9 (SD 3.4)<br>STAI: 44.9 (SD 11.5)<br>BDI: 11.2 (7.4)	Married: 50%<br>High school graduate and above: 53.3% (≥ 10 years)<br>Employed: 50%	ACP: 2/15<br>OCB: 4/15<br><br>ACP: 10/15<br>OCB: 10/15
**De Fuente 1989** [25]<br>Mexico<br><br>Supported in part by CONACYT-Mexico	RCT, double-blind<br><br>Alcoholism and Drug Dependence Unit, Instituto Mexicano de Psiquiatria, Mexico<br><br>N = 53; 44 (SD 12); 26.4%	National Council on Alcoholism major diagnostic criteria<br><br>In-paitent 4-week detoxification by chlordiazepoxide and psychosocial therapy	1. Placebo (N = 25)<br>2. Lithium: 0.6-1.2 mEq/L (N = 28)<br><br>6 months	NR	NR	Probable depression: 47%	NR	PLA: 7/25<br>LIT: 10/28<br><br>PLA: 12/25<br>LIT: 14/28
**Favre 1997** [26 27]<br>France<br><br>Aug 1990 to Jun 1994	RCT, double-blind<br><br>Multicentre<br><br>N = 342; 42.1 (SD 7.6); 14.5%	DSM III-R<br>In-or out-patient detoxification	1. Placebo (N = 172)<br>2. Tianeptine: 12.5 mg TID Oral (N = 170)<br><br>9 months	NR	Duration of the dependence (year): 6.5 (5.8)<br><br>Severity of dependence (DSM III-R):<br>Mild: 5.5%<br>Moderate: 43.5%<br>Severe: 51%<br><br>Short-MAST: 8.5 (2.3)<br>Previous alcohol withdrawals: 2.2 (1.9)	MADRS: 7.8 (SD 5.2)<br>HSCL: 30.2 (SD 23.8)	NR	PLA: 42/172<br>TIP: 48/170<br><br>PLA: 94/172<br>TIP: 112/170
**Florez 2008** [28]<br>Spain<br><br>Jan 2005 to Feb 2006	RCT, open-label<br><br>Outpatient alcohol clinic<br><br>N = 102; 46.8 (SD 8.6); 14.7%<br>6 months	ICD-10<br><br>Detoxification via clorazepate (<14 days)	1. Naltrexone: 50 mg/day (N = 51)<br>2. Topiramate: 50 mg/day, increased to 200 mg/day. Max 400 mg/day (N = 51)<br><br>6 months	45 to 60-minute Individualized psychological therapy based on the Relapse Prevention Model (Carroll, 1996; Irvin et al., 1999; Jaffe et al., 1996)	OCDS total: 17.2 (SD 7.2)<br><br>Alcohol intake (> 700 g/week): 73.5%	Fagerstrom: 3.54 (SD 3.65)<br>Personality disorders: 27.5%	Married: 69.6%<br>Employed: 24.5%<br>High school graduate and above: 17.6%<br><br>Family history of alcoholism: 51.0%	NTX: 23/51<br>TPM: 24/51<br><br>NTX: 6/51<br>TPM: 4/51
**Florez 2011** [29]<br>Spain	RCT, open-label<br><br>Outpatient addiction treatment clinic<br><br>N = 182; 47.8 (SD 9.2); 14.8%	ICD-10<br><br>Detoxification by clorazepate	1. Naltrexone: 50 mg/day Oral (N = 91)<br>2. Topiramate: 50mg/day initially, increased to 200 mg/day Oral (N = 91)<br><br>6 months	BRENDA weekly	OCDS total: 18.1 (SD 7.6)<br>Alcohol intake (> 700 g/week): 74.2%	Fagerstrom: 3.59 (SD 3.69)<br>Personality disorders: 23.1%	Married: 62.1%<br>Employed: 46.7%<br>Elementary school only: 87.9%<br><br>Family history of alcoholism: 72.0%	NTX: 38/91<br>TPM: 43/91<br><br>NTX: 11/91<br>TPM: 6/91

**Friedmann 2008** [30]<br>USA<br><br>Jun 2002 to Jan 2006<br>Supported by NIAAA	RCT, double-blind<br><br>Two centres in the USA<br><br>N = 173; 41 (SD 7.2); 8.7%	DSM-IV<br><br>In-patient 5-day detoxification with chlordiazepoxide	1. Placebo (N = 85)<br>2. Trazodone: 50 mg before bedtime. Max 150 mg. (N = 88)<br><br>12 weeks	NR	Drinks per drinking day in past 3 months: 21.9 (SD 12.7)<br>Mean proportion of days abstinent in past 3 months: 0.21 (0.27)<br>Mean proportion of heavy drinking days: 75.0%	All experienced sleep disturbance during previous periods of abstinence or a global score of 5 or greater on the Pittsburgh Sleep Quality Index (PSQI)<br><br>Sleep quality: 11.9 (3.5)<br>% Depressed: 30.1%	Homeless: 20.8%<br>Unemployed: 34.4%<br>Caucasian: 86.2%<br>12+ years of school: 74.1%	PLA: 12/85<br>TZD: 8/88<br><br>PLA: 16/85<br>TZD: 16/88
**Fuller 1986** [31 32]<br>USA<br><br>Jul 1979 to Jul 1983	RCT<br><br>Nine Veterans Administration medical centres<br><br>N = 605; 41.7 (SD 10.3); 0%	National Council on Alcoholism<br><br>In-patient detoxification	1. Placebo (riboflavin 50mg) (N = 199)<br>2. Disulfiram: 1 mg (N = 204)<br>3. Disulfiram: 250 mg (N = 202)<br><br>1 year<br><br>Group 1 and 2 results were combined for meta-analysis	Counselling or psychotherapy once a week for 6 months and then biweekly for the next 6 months	Duration of ethanol abuse (year): 11.7 (SD 9.9)<br>Days drank in month prior to study: 20.4 (SD 9.9)	NR	Non-white: 46%<br>Married: 70.0%<br>Employed: 53.7%<br>High school graduate and above: 74.4%	PLA: 78/403<br>DSF: 38/202<br><br>PLA: 20/403<br>DSF: 8/202
**GATE 2** [33-35]<br>Austria, Germany, Italy and Poland	RCT, double-blind<br><br>Multicentre<br><br><br>N = 314; NR; NR	DSM-IV<br><br>Detoxification	1. Placebo (N = 160)<br>2. GHB: 3.06 g/day for BW < 65kg and 3.5g/day for BW > 65 kg (N = 154)<br><br>6 months/12 months	NR	NR	NR	NR	PLA: 48/160<br>GHB: 63/154<br><br>PLA: 129/160<br>GHB: 114/154
**Geerlings 1997** [36]<br>Beligum, Luxembourg, Netherlands<br><br>Funded by Lipha Belgium	RCT, double-blind<br><br>22 treatment centres<br><br>N = 262; 41.0 (SD 8.7); 24.1%	DSM-III<br><br>Detoxification	1. Placebo (N = 134)<br>2. Acamprosate: 1332-1998 mg/day, depending on weight. BW ≥ 60 kg: 1998 mg/day; BW < 60 kg: 1332 mg/day Oral (N = 128)<br><br>6 months/12 months	Out-patient psychosocial intervention	Duration of alcohol problems (year): 11.1 (SD 8.0)<br>N of previous weaning cures: 2.5 (SD 4.2)<br><br>Daily consumption:<br><5 std drinks/day: 3.5%<br>5-10 std drinks/day: 23.0%<br>> 10 std drinks/day: 73.4%<br><br>Frequency:<br><2 times/week: 6.5%<br>2-6 times/week: 28.0%<br>Daily: 65.6%	NR	NR	PLA: 7/134<br>ACP: 14/128<br><br><br>PLA: 111/134<br>ACP: 98/128
**Gottlieb 1994** [37]<br>USA<br><br>Supported in part by Stuart Pharmaceuticals/ICI Pharma	RCT, double-blind<br><br>Acute Care and Evaluation Unit of St. Mary's Hospital<br><br>N = 100; 19 (SD 13.4); NR	SADQ<br><br>Supervised alcohol withdrawal	1. Placebo (N = 50)<br>2. Atenolol: 0-100 mg/day, depending on heart rate Oral (N = 50)<br><br>1 year	Customary behavioural relapse prevention therapy	Craving for alcohol: 28.5<br>SADQ (0-60) (median): (P: 25; A: 27)	NR	High school graduate: 64%	PLA: 8/50<br>ATL: 7/50<br><br>PLA: 28/50<br>ATL: 29/50
**Gual 2001** [38]<br>Spain<br><br>Funded by Merck Lipha, Spain	RCT, double-blind<br><br>11 centres<br><br>N = 296; 41.0 (SD 9.2); 20.4%	DSM-III-R<br><br>In- or out-patient detoxification by tetrabamate or chlomethiazole during the first 14-day period	1. Placebo (N = 147*)<br>2. Acamprosate: 1998mg/day Oral (N = 141*)<br><br>180 days<br><br>*ITT sample used in the trial	NR	DSM-III-R total index: 7.77 (SD 1.3)<br>MAST: 27.8 (SD 8.5)<br>Dependence duration (year): 12.8 (SD 7.9)<br><br>Frequency of alcohol consumption:<br>2 times: 2.1%<br>> 2 times: 13.9%<br>Daily: 84%<br><br>Alcohol quantity (day per drinking day):<br>< 5: 3.8%<br>5-10: 29.9%<br>>10: 66.3%	Antabuse (disulfiram prescription): 52.4%	NR	PLA: 26/147<br>ACP: 35/141<br><br>PLA: 57/147<br>ACP: 45/141
**Gual 2002** [39]<br>Spain	RCT<br><br>Four Spanish hospitals<br><br>N = 81; 39.6 (SD 8.5); 14.9%	DSM-III-R<br><br>Detoxification	1. Placebo (N = 43)<br>2. Tiapride: 100mg every 8 hours Oral (N= 38)<br><br>180 days	NR	Age of beginning drinking (< 15 years): 67.9%	NR	Married: 77.3%<br><br>Education (primary): 47%<br><br>Family history of alcoholism: 49.7%	PLA: 19/43<br>TPD: 11/38<br><br>PLA: 13/43<br>TPD: 7/38

**Gustafson 2014** [40 41]<br>USA<br><br>Feb 2010 to Jun 2012<br>NCT101003119	RCT, open-label<br><br>Three residential programs<br><br>N = 349; 38.4 (SD 10.4); 39.2%	DSM-IV<br><br>Detoxified from residential programs	1. Control (TAU) (N = 179)<br>2. Addiction-Comprehensive Health Enhancement Support System (A-CHESS; smartphone-based application) (N = 170)<br><br>12 months	Varied depending on the centre, containing CBT, counselling, motivational interviewing and psychoeducation...etc.<br><br>AA	NR	Use or abuse drugs besides alcohol:62.5%<br>Have other mental health problems/issues: 47.0%<br>Continues to be affected by history of emotional or physical trauma: 53.3%	White: 80.2%<br>Unemployed: 78.5%<br>High school graduate and above: 79.9%	TAU: 63/179<br>A-CHESS: 81/170<br><br>TAU: 40/179<br>A-CHESS: 38/170
**Huang 2002** [42]<br><br>China<br><br>Dec 1995 to Dec 1999	RCT, single-blind<br><br>Guangzhou Psychiatric Hospital<br><br>N = 45; 45 (SD 8); 0%	DSM-IV and CCMD-2-R<br><br>Inpatient detoxification	1. Placebo (N = 23)<br>2. Naltrexone: 30 mg/day Oral (N = 20)<br><br>12 weeks	NR	Year of drinking heavily (year): 18.3 (SD 5.5)<br>N of inpatient detoxification: 2.2 (SD 1.7)<br>Amount of drinking (g/day): 319.8 (SD 82.3)<br><br>Craving: 2.92 (SD 0.88)	NR	NR	PLA: 6/22<br>NTX: 16/23<br><br>PLA: 2/22<br>NTX: 1/23
**Huang 2005** [43]<br>Taiwan	RCT, double-blind<br><br>A psychiatric hospital<br><br>N = 40; 40.5 (SD 8.0); 0%	DSM-III-R<br><br>In-patient 2-week detoxification	1. Placebo (N = 20)<br>2. Naltrexone: 50 mg/day (N = 20)<br><br>14 weeks	Weekly 30-minute individual supportive psychotherapy sessions	Age of habitual drinking (year): 26.9 (6.8)<br>Baseline alcohol craving score (VAS): 6.3 (2.5)	NR	Married: 70%<br>≥ 9 years of education: 40%<br>SES I-III: 12.5%<br>SES IV-V: 87.5%	PLA: 13/20<br>NTX: 11/20<br><br>PLA: 7/20<br>NTX: 9/20
**Janiri 1997** [44]<br>Italy	RCT, open<br><br>NR<br><br>N = 50; 43.7 (SD 11.6); 20%	DSM-IV<br><br>Detoxification	1. Fluvoxamine: 100 mg/day (N = 25)<br>2. Fluoxetine: 20 mg/day (N = 25)<br><br>90 days	NR	NR	NR	NR	FLT: 9/25<br>FLX: 3/25
**Jirapramukpitak 2020** [45]<br>Thailand<br><br>Jul 2015 to Apr 2016<br>TCTR20160215004<br><br>Supported by Thai Health Promotion Foundation (58-07-007)	RCT<br><br>A university hospital<br><br>N = 161; 50.1 (SD 11.5); 24.8%	DSM-IV<br><br>Detoxified with benzodiazepaines, folic acid, vitamin B (1, 6 and 12) along with brief advice and psychoeducation for 2-4 weeks	1. Home-visit: 40 visits during the 12-week (N = 80)<br>2. Contingency management Low (CM-L): in addition to the Home-visit, 30 baht every time when patients had a negative for alcohol (N = 42)*<br>3. CM High (CM-H) High: in addition to the Home-visit, 60 baht every time when patients had a negative for alcohol (N = 37)*<br><br>12 weeks<br><br>*Groups 2 & 3 were combined in the NMA	NR	NR	Smoking: 60.3%<br><br>Combined psychiatric illness: 3.7%	Education (Primary school or lower): 61.5%	HOV: 12/80<br>CM: 10/79<br><br>HOV: 1/80<br>CM: 1/79
**Joos 2013** [46]<br>**Belgium**<br><br>**NTR1736**<br>**Oct 2009 to Jul 2011**	RCT, double-blind<br><br>Two addiction treatment centers<br><br>N = 83; 41.8 (SD 9.4); 14.5%	DSM-IV<br><br>Detoxification	1. Placebo (N = 42)<br>2. Modafinil: 100 mg/day initially, increased to 300 mg/day (N = 41)<br><br>10 weeks/8.5 months	Behaviourally orientated treatment program within a residential and/or a day care setting.	Age of onset of heavy drinking (year): 28.9 (SD 11.0)<br><br>Years of heavy drinking: 10.7 (SD 7.8)<br>%heavy drinking days in 30 days before admission: 52.4% (SD 36.3)<br>%days abstinent in 30 days before admission: 43.7% (SD 36.9)	Non-smokers, %: 13.2<br><br>Cannabis: 18.4%; Other substance/poly: 13.6%	Married: 25.3%	PLA: 6/42<br>MDF: 12/41<br><br>PLA: 15/42<br>MDF: 17/41
**Kampman 2007** [47 48]<br>**USA**<br><br>**Funded by AstraZeneca Pharmaceuticals**	RCT, double-blind<br><br>NR<br><br>N = 61; 47 (SD 8.8); 33%	DSM-IV<br><br>Detoxification (unknown)	1. Placebo (N = 32)<br>2. Quetiapine: 50 mg/day, increased to 400 mg/day (N = 29)<br><br>12 weeks	Weekly BRENDA (20-30 minutes per session)	%drinking days in the 90 days prior to detoxification: 77%<br>%heavy drinking days in the 90 days prior to detoxification: 72%<br>Drinks per drinking day: 15.5 (SD 10.3)<br>ASI: 0.633 (SD 0.196)	HAM-D: 7.0 (SD 6.2)<br>HAM-A: 5.0 (SD 5.0)	White: 54%<br>Married: 31.2%<br>Employed: 90.1%<br>Education (year): 13.8 (SD 2.6)	PLA: 2/32<br>QTP: 9/29<br><br>PLA: 8/32<br>QTP: 6/29
**Kiefer 2003** [49-53]<br>**Germany**<br><br>**Nov 1998 to Nov 2000**<br>**Supported by Unviersity of Hamburg, DuPont (medication), and Merck (medication)**	RCT, double-blind<br><br>Two Hospitals<br><br>N = 160; 46.2 (SD 9.3); 26.3%	DSM-IV (at least 5 criteria)<br><br>In-patient detoxification	1. Placebo (N = 40)<br>2. Acamprosate: 1998 mg, divided as TID Oral (N = 40)<br>3. Naltrexone: 50 mg/day Oral (N = 40)<br>4. Combined: ACP 1998 mg/day + NTX 50 mg/day (N = 40)<br><br>Patients started with the intake of medication 5 +/- 1 days before discharge from inpatient treatment.<br><br>12 weeks	Weekly group therapy (coping skills and relapse prevention)	Years since first alcohol-related problems occurred: 10.14 (SD 8.4)<br>Years since first signs of withdrawal occurred: 7.42 (SD 8.09)<br>N of inpatient detoxification: 2.69 (SD 4.03)<br>Alcohol intake (g/day): 254.9 (SD 129.4)<br><br>OCDS: 17.6 (SD 12.0)<br>VAS: 21.2 (SD 27.3)	No. of cigarettes per day: 22.7 (15.4)<br><br>SCL-90 (N = 143): 61.3 (51.8)<br>Somatic distress (N =143): 7.5 (6.7)<br>Depression (N=143): 12.7 (8.8)<br>Anxiety (N=143): 7.3 (6.4)	Married: 28%<br>Partnership: 51%<br>High school: 22%<br>Unemployed: 39%<br><br>Family history of alcoholism: 45%	PLA: 10/40<br>ACP: 17/40<br>NTX: 22/40<br>ACP+NTX: 26/40<br><br>PLA: 30/40<br>ACP: 23/40<br>NTX: 18/40<br>ACP+NTX: 14/40

**Ladewig 1993 [54]**<br>**Switzerland**	RCT, double-blind<br><br>Three centres in Switzerland<br><br>N = 62; 46.8 (SD 10.2); NR	DSM-III<br><br>Five-day detoxification	1. Placebo (N = 32)<br>2. Acamprosate: 1332-1998 mg/day depending on weight. BW < 60kg: 1332 mg/day; BW ≥ 60kg: 1998 mg/day (N = 29)<br><br>180 days/360 days	NR	MAST: 38.0 (SD 39.1)	NR	NR	ACP: 8/29<br>PLA: 4/32
**Landabaso 1999 [55]**<br>**Spain**	RCT<br><br>NR<br><br>N = 30; 30.6 (SD 6.2); 26.7%	DSM-IV<br><br>Detoxification (unknown)	1. TAU (N = 15)<br>2. Naltrexone: 25 mg/day Oral (N = 15)<br><br>6 months/18 months	Usual treatment (supportive psychotherapy) with an aversion agent	NR	NR	Married: 53.4%<br>Employed: 76.7%	TAU: 3/15<br>NTX: 11/15<br><br>TAU: 7/15<br>NTX: 2/15
**Mann 2006 [56]**<br>**Germany**<br><br>**1997 to 2001**<br>**Supported by J. Moormann, M.D. (HF-Arzneimittel, Werne, Germany)**	RCT, double-blind<br><br>Seven German psychiatric hospitals<br><br>N = 151; 43.4 (SD 8.7); 30.2%	ICD-10 and DSM-IV<br><br>In-patient or out-patient detoxification and abstinent for 3–25 days	1. Placebo (N = 75)<br>2. Galantamine: 25 mg Transdermal (N = 74)<br><br>12 weeks/24 weeks	Low-intensity psychosocial standard therapy	OCDS-G: 10.6 (SD 6.9)<br>Age at onset of regular alcohol consumption (year): 25.0 (SD 8.6)<br>Age at onset of alcohol dependence (year): 34.9 (SD 8.3)	Smokers n (%): 76.5%	NR	PLA: 23/75<br>GAL: 9/74<br><br>PLA: 42/75<br>GAL: 44/74
**Marra 2002 [57]**<br>**France**<br><br>**Funded by Sanofi-Synthelabo**	RCT, double-blind<br><br>Two hospitals of Assistance Publique-Hôpitaux de Paris<br><br>N = 72; 45.2 (SD 7.6); 31.0%	DSM-IV<br><br>Inpatient 10-18 days detoxification	1. Placebo (N = 34*)<br>2. Amisulpride: 50 mg/d (N = 37*)<br><br>6 months<br><br>*One patient excluded in the ITT sample but uncertain in which group	Individual counselling	OCDS-O (0-24): 9.8 (4.0)<br>OCDS-C (0-32): 13.0 (3.1)<br>Number of previous participation in inpatient detoxification programmes: 1.3 (SD 2.0)<br>N of days of abstinence 6 months before detoxification: 26.1 (SD 30.3)<br>Age of onset of alcohol dependence: 35.4 (SD 8.9)<br><br>Alcohol consumption (g/week): 1295.2 (SD 663.6)	Antidepressant use: 12.7%<br>Generalized anxiety disorder: 14.1%	Employed: 59.2%<br>Living alone: 35.2%<br>Education (>7 years): 70.4%	PLA: 8/34<br>AMS: 4/37<br><br>PLA: 20/34<br>AMS: 27/37
**Martinotti 2007 [58]**<br>**Italy**<br><br>**Sep 2005 to Aug 2006**	RCT, open-label<br><br>Day-Hospital of Psychiatry and Drug Dependence of the University General Hospital 'A. Gemelli'<br><br>N = 84; 46.3 (SD 11.9); 19%	DSM-IV<br><br>3-5 days detoxification by benzodiazepines	1. Naltrexone: 10 mg/day for one week, then increased to 50 mg/day (N = 27)<br>2. Oxcarbazepine (High-dose): 600 mg/day for one week, then increased to 1500-1800 mg/day (N = 29)<br>3. Oxcarbazepine (Low-dose): 300 mg/day for one week, then increased to 600-900 mg/day (N = 28)<br><br>90 days<br><br>Group 2 and 3 were combined for the meta-analysis	Supportive self-help group (2 days/week)	Duration of alcohol misuse (year): 16.1 (SD 7.9)<br>OCDS: 20.0 (SD 12.1)<br>VAS: 3.5 (3.6)	Multiple substance abuse: 32.1%<br>Dual diagnosis (axis I): 41.7%<br>SCL-90-R (GSI): 1.1 (SD 0.7)	Married: 32.1%<br>High school and above: 57.1%	NTX: 11/27<br>OCB: 29/57<br><br>NTX: 6/27<br>OCB: 9/57
**Martinotti 2009 [59]**<br>**Italy**<br><br>**Aug 2006 to Nov 2006**	RCT, double-blind<br><br>Day-Hospital of Psychiatry and Drug Dependence of the University General Hospital 'A. Gemelli'<br><br>N = 57; 40.3 (SD 11.8); 20%	DSM-IV<br><br>5-10 days detoxification by benzodiazepines	1. Naltrexone: 10 mg/day for one week, then increased to 50 mg/day Oral (N = 28)<br>2. Aripiprazole: 5 mg/day for one week, then increased to 5-15 mg/day Oral (N = 29)<br><br>16 weeks	Supportive self-help group (2 days/week)	Daily drinks: 8.5 (SD 3.5)<br>Years of addiction (year): 14.8 (SD 6.7)	Axis I diagnosis: 49.1%<br>Axis II diagnosis: 29.8%<br>Cannabis abuse: 15.8%<br>Cocaine abuse: 10.5%<br>BZD abuse: 1.8%<br>MDMA abuse: 1.8%<br>Tobacco smoking: 61.4%	NR	NTX: 11/28<br>ARI: 12/29<br><br>NTX: 7/28<br>ARI: 7/29
**Martinotti 2010 [60,61]**<br>**Italy**	RCT, double-blind<br><br>Day-Hospital of Psychiatry and Drug Dependence of the University General Hospital<br><br>N = 59; 40.2 (SD 11.8); 20%	DSM-IV<br><br>5-10 days detoxification by diazepam	1. Naltrexone: 10 mg/day for one week, then increased to 50 mg/day (N = 28)<br>2. Pregabalin: 50 mg/day for one week, then increased to 150-450 mg/day (N = 31)<br><br>16 weeks	Supportive self-help group (2 days/week)	Mean daily drinks: 8.5 (SD 3.5)*<br>Years of addiction: 14.8 (SD 6.7)*<br><br>*N = 71 from recruitment stage	Axis I diagnoses: 34.0%<br>Axis II diagnoses: 34%<br>Cannabis abuse: 13.4%<br>Cocaine abuse: 8.5%<br>BZD abuse: 1.7%<br>Tobacco smoking: 59.3%	NR	NTX: 11/28<br>PGB: 15/31<br><br>NTX: 7/28<br>PGB: 4/31

**MATCH project[62-66]**<br>**USA**<br><br>**Supported by NIAAA**	RCT<br><br>Only participants recruited from five aftercare sites were included in this review<br><br>N = 774; 41.9 (SD 11.1); 20%	DSM-III-R<br><br>At least 7 days of inpatient or intense day hospital treatment	1. TSF (TAU): 12 sessions over 12 weeks as described by Nowinski et al. Aimed to help individual become an active participant in AA meetings (N = 247)<br>2. CBT: 12 sessions over 12 weeks as described by Kadden et al. (N = 266)<br>3. MET: 4 sessions over 12 weeks with the last two sessions conducted at Weeks 6 and 12 as described by Miller et al. (N = 261)<br><br>12 weeks/9 and 15 months	NR	N of DSM-III-R criteria: 6.8 (SD 1.9)<br>Problem drinking (year): 14.8 (SD 10.0)<br>% any alcohol treatment: 61.8%<br><br>Average drinking per drinking day: 20.5 (SD 12.1)	Lifetime Axis diagnosis: 59.1%<br><br>Current illicit drug use: 31.9%	Married: 32%<br>Six months' continuous employment: 48%<br><br>[Ethnicity]<br>Non-white: 20%	TAU: 72/247<br>CBT: 79/266<br>MET: 69/261<br><br>TAU: 11/247<br>CBT: 10/266<br>MET: 12/261
**Moncini 2000 [67 68]**<br>**Italy**	RCT, double-blind<br><br>Toxicological Unit of the Department of Pharmacology, Florence University<br><br>N = 17; 46.4; 23.5%	DSM-IV<br><br>30-day in-patient detoxification	1. Placebo (N = 8)<br>2. GHB: 50 mg/kg/day (N = 9)<br><br>6 months	NR	NR	NR	NR	PLA: 4/8<br>GHB: 6/9<br><br>PLA: 2/8<br>GHB: 2/9
**Moraes 2010 [69 70]**<br>**Brazil**<br><br>**2004 to 2005**<br>**Supported by the Sao Paulo State Research Foundation-FAPESP**	RCT, open-label<br><br>Alcohol and Drugs Research Unit (UNIAD), an outpatient clinic of the Department of Psychiatry, Universidade Federal de Sao Paulo (UNIFESP), Brazil<br><br>N = 120; 43 (range 21-59); 10%	DSM-IV<br><br>Detoxification	1. Conventional treatment (CT; TAU): 20 psychotherapy group sessions in 10-week (N= 58)<br>2. Home visit (CT + Home visit): 4 visits by a psychologist and a social worker with strategies of the motivational interview (N = 62)	NR	Severe AD: 85%<br><br>Consumption in the month:<br>Mild (< 4 shots/d): 3 days<br>Moderate (5-9 shots/day): 4.7days<br>Heavy (≥10 shots/d): 4.1 days	Anxiety or depression (SRQ-20): 25.8%<br>Cognitive impairment (scale FAB): 63.3%	Married: 41.7%<br>White: 76.7%<br>Elementary education and above: 67.5%<br>Independent workers: 34.2%	TAU: 25/58<br>HOV: 36/62<br><br>TAU: 22/58<br>HOV: 9/62
**Mueller 1997 [71]**<br>**USA**<br><br>**Mar to Aug 1993**<br>**Medications were provided by Ciba-Geig**	RCT, double-blind<br><br>Butler Hospital, USA<br><br>N = 29; 38.7 (SD 8.5); 37.9%	DSM-III-R<br><br>Detoxification by chlordiazepoxide	1. Placebo (N = 16)<br>2. Carbamazepine: 100 mg TID in the first day, then 200 mg TID (N = 13)<br><br>12 months	NR	Age drinking became a problem (year): 24.1 (SD 9.5)<br>SADD: 25.5 (SD 10.2)<br><br>%Drinking days: 76.8 (SD 25.4)<br>Drinks per drinking day: 16.1 (SD 8.9)	Beck Depression Inventory score: 17.3 (SD 9.9)<br>Global Assessment of Function: 52.8 (SD 5.2)<br>California Personality Inventory Socialization: 25.2 (SD 4.0)<br><br>[Speilberger Anxiety]<br>State: 52.3 (SD 11.8)<br>Trait: 51.9 (SD 12.9)	Married: 51.7%<br>Level of education (yr): 13.0 (SD 2.7)<br>%Caucasian: 89.7%	PLA: 4/16<br>CBZ: 2/13<br><br>PLA: 8/16<br>CBZ: 12/13
**Oslin 2005 [72]**<br>**USA**<br><br>**Supported by NIMH, Pfizer Pharmaceuticals (medication) and DuPont Pharmaceuticals (medication)**	RCT, single-blind<br>Philadelphia VA Medical Centre<br><br>N = 74; 63.4 (SD 6.3); 20.3%	DSM-IV<br><br>Detoxification from alcohol (a minimum of 3 consecutive days of abstinence)	1. Placebo (N = 37)<br>2. Naltrexone: 50 mg/day Oral (N = 37)<br><br>Both groups received sertraline 50 mg/day for one week, then increased to 100 mg/day<br>12 weeks	Compliance-enhancement therapy (BRENDA)	Years of alcohol use: 39.6 (SD 10.8)<br>Years of drinking-to-intoxication: 17.3 (SD 9.9)<br>%days drinking: 79.0% (SD 26.9)<br>Drinks per drinking day: 8.4 (SD 5.4)<br>%heavy drinking day 90 days before detoxification: 67.5% (SD 33.3)<br>Previous alcohol dependence treatment: 48.6%<br>ASI Alcohol Score: 0.66 (SD 0.17)	HAM-D: 21.8 (SD 5.6)<br>PCS (SF-36): 45.0 (SD 9.4)<br>MCS (SF-36): 35.7 (SD 10.8)<br>Primary depression (%): 67.2%<br>Independent major depression: 31.1%	Married: 44%<br>Caucasian: 66.3%	PLA: 20/37<br>NTX: 16/37<br><br>PLA: 4/37<br>NTX: 7/37
**Paille 1995 [73]**<br>**France**<br><br>**Apr 1989 to Nov 1992**	RCT, double-blind<br><br>31 specialist alcohol centres<br><br>N = 538; 43.2 (SD 8.6); 20%	DSM-III-R<br><br>In- or out-patient detoxification and 7-28 days of abstinence	1. Placebo (N = 177)<br>2. Acamprosate: 1.3 g/day Oral (N = 188)<br>3. Acamprosate: 2 g/day Oral (N = 173)<br><br>Group 2 and 3 combined for the meta-analysis<br><br>12 months/18 months	Supportive psychotherapy	Duration of consumption (year): 9.5 (SD 7.2)<br><br>[Number of previous detoxifications]:<br>None; 50.3% (270/537)<br>More than one: 49.7% (267/537)	Covi anxiety scale score before withdrawal: 5.6 (SD 2.7)<br>Raskin depression scale score before withdrawal: 4.4 (SD 2.9)	Living alone: 13.9%<br>Employed: 47.0%	PLA: 20/177<br>ACP: 67/361<br><br>PLA: 115/177<br>ACP: 186/361

**Pelc 1992 [74]**<br>**NR**	RCT, double-blind<br><br>Multicentre<br><br>N = 102; NR; NR	DSM-III-R<br><br>Detoxification	1. Placebo (N = 47)<br>2. Calcium acetyl homotaurinaæ (acamprosate) 1998 mg Oral (N = 55)<br><br>180 days	NR	NR	NR	NR	PLA: 2/47<br>ACP: 14/55<br><br>PLA: 38/47<br>ACP: 31/55
**Pelc 1996 [75]**<br>**Belgium and France**<br><br>**Funded by Lipha Belgium**	RCT, double-blind<br><br>11 centres<br><br>N = 188; NR; NR	DSM-III-R<br><br>14-day in-patient detoxification programme	1. Placebo (N = 62)<br>2. Acamprosate: 1332 mg/day Oral (N = 63)<br>3. Acamprosate: 1998 mg/day Oral (N = 63)<br><br>Group 2 and 3 were combined for meta-analysis<br><br>90 days	Supportive counselling and social support when needed	NR	NR	NR	PLA: 16/62<br>ACP: 60/126<br><br>PLA: 30/62<br>ACP: 39/126
**Pelc 2005 [76]**<br>**Beligum**<br><br>**Apr 1997 to Mar 1998**<br>**Funded by Merck**	RCT, open-label<br><br>An addiction clinic in the Psychiatry Department of the Brugmann University Hospital<br><br>N = 100; 43.3 (SD 8.0); 22%	DSM-IV<br><br>3-week acute detoxification programme; 1-week abstinence	1. Acamprosate + Standard care: ACP 1332-1998 mg/day, adjusted by weight. BW≥ 60 kgs: 1998 mg/day; BW < 60 kgs: 1332 mg/day Oral (N = 50)<br>2. Acamprosate + Nurse follow-up: ACP regimen + telephone (weekly) and home visit by community nurses (N = 50)<br><br>6 months	NR	DSM IV score: 6.2 (0.9)<br>N of drinks/day: 19.1 (SD 11.1)<br><br>Years of alcohol dependence: 14.1 (9.7)<br>N of previous withdrawals: 0.4 (0.6)	Regular smoker: 82%	Married: 18%<br>Above secondary: 68%<br><br>Family history: 63%	ACP: 8/50<br>ACP+NUS: 16/50<br><br>ACP: 42/50<br>ACP+NUS: 30/50
**Poldrugo 1997 [77]**<br>**Italy**<br><br>**Nov 1989 to Jun 1992**	RCT, double-blind<br><br>Multicentre, five alcoholism treatment units in the North-eastern region of Italy<br><br>N = 246; 43.9 (SD 9.7); 27.2%	DSM-III<br><br>Inpatient detoxification	1. Placebo (N = 124)<br>2. Acamprosate: 1332-1998 mg, adjusted by weight. BW≥ 60 kgs: 1998 mg/day; BW < 60 kgs: 1332 mg/day Oral (N = 122)<br><br>6 months/12 months	Psychological support, including group sessions, family therapy, education on alcoholism, community meetings and physical and recreational activities. ("Club of Treated Alcoholics")	MAST total: 27.6 (SD 10.4)<br>Psychotherapy use: 28.9%<br>Previous participation to exalcoholics: 27.2%<br>Previous disulfiram use: 20.3%<br><br>Quantity on a drinking day:<br>< 5 drinks: 5.3%<br>5-10 drinks: 19.5%<br>>10 drinks: 75.2%<br><br>Frequency on a drinking week:<br><3 days: 1.2%<br>3-6 days: 13%<br>Daily: 85.8%	Disulfiram at inclusion (yes): 20.7%<br>Other drugs at inclusion (yes): 13.4%<br>HAM-DI: 23.3 (3.3)<br>HAM-A: 3.8 (5.7)	Family history of alcoholism: 50.8%	PLA: 37/124<br>ACP: 53/122<br><br>PLA: 83/124<br>ACP: 62/122
**Ponce 2005 [78]**<br>**Spain**	RCT, single-blind<br><br>Addictive behavior unit of the hospital October 12 Madrid<br><br>N = 100; 36.8 (SD 10.1); 100%	DSM-IV<br><br>Inpatient detoxification	1. No Naltrexone (TAU) (N = 50)<br>2. Naltrexone: 50 mg/day Oral (N = 50)<br><br>12 weeks	Meeting with psychiatrist every 7 days in the first month and then after 15 days.	Age of first contacting alcohol (year): 16.0 (SD 6.2)<br>Age of habit alcohol consumption (year): 23.3 (SD 11.0)<br>Age of consumption being abuse (year): 29.3 (SD 13.2)<br>Age of alcohol dependence (year): 35.0 (SD 15.3)<br>Consumption diary: 79.4%	NR	Family history of psychiatric disorders: 32.8%<br>Family history of alcoholism: 63.6%	TAU: 21/50<br>NTX: 38/50<br><br>TAU: 19/50<br>NTX: 8/50
**PREDICT [79-82]**<br>**Germany**<br><br>**Nov 2002 to Sep 2006**<br>**NCT00317031**	RCT, double-blind<br><br>Multicentre in Germany<br><br>N = 426; 45.3 (8.7); 23%	DSM-IV<br><br>Inpatient detoxification	1. Placebo (N = 85)<br>2. Acamprosate: 1998 mg/day Oral (N = 172)<br>3. Naltrexone: 50 mg/day Oral (N = 169)<br><br>12 weeks/12 months	Medical management (Pettinati et al 2004)	N of DSM-IV symptoms: 6.1 (SD 1.1)<br>ADS: 15.0 (SD 6.7)<br>OCDS: 13.6 (SD 6.1)	NR	Married: 39%<br>Employed: 48%	PLA: 41/86<br>ACP: 76/172<br>NTX: 73/169<br><br>PLA: 8/86<br>ACP: 22/172<br>NTX: 18/169
**Richter 2012 [83]**<br>**Germany**<br><br>**NCT00758277**<br>**Jan 2005 to Jul 2009**<br>**Funded partially by UCBPharma**	RCT, double-blind<br><br>Multiple centre (10 hospitals)<br><br>N = 201; 47.7 (SD 9.5); 28.4%	DSM-IV or ICD-10<br>inpatient detoxification	1. Placebo (N = 106)<br>2. Levetiracetam: 1000mg/day in the first week, then increased to 2000 mg/day, then tapered down to 500 mg/day last week Oral (N = 95)<br><br>16 weeks	NR	Mean duration of alcohol consumption in years: 17.0 (10.6)	Smokers: 70.6%	NR	PLA: 36/106<br>LEV: 33/95<br><br>PLA: 26/106<br>LEV: 12/95

**Rubio 2005** [84]<br>**Spain**	RCT, open-label<br><br>Addictive Behaviour Unit of the 'Doce de Octubre' hospital (Madrid).<br><br>N = 336; 41.6 (SD 8.6); 0%	DSM-IV<br><br>5–10 days detoxification by diazepam and abstinence (mean of 14.5 days (SD 7.2))	1. Non-naltrexone (TAU) (N = 168)<br>2. Naltrexone: 50 mg/day (N = 168)<br><br>Patients with depression or anxiety disorder, sertraline (100-200 mg/day) was added.<br><br>3 months	Supportive group therapy weekly	Alcohol consumption (g/occasion): 219.33<br>Amount of alcohol per day: 218.5 (SD 57.9)<br>Heavy drinking days per 28days: 25.1 (SD 9.2)<br>Age of onset of habitual consumption (year): 16.5<br>Beginning of alcohol problems (year): 22.8	Other substance use disorders (excluded nicotine): 21.7%<br>Use of disulfiram: 24.7%<br>Use of sertraline: 24.4%<br>Antecedents of depressive/anxiety disorders: 15.5%<br>FHA+: 61.9%<br>Family history of other psychiatric disorders: 23.2%	NR	TAU: 95/168<br>NTX: 111/168<br><br>TAU: 58/168<br>NTX: 47/168
**Sass 1996** [85-87]<br>**Germany**<br><br>**Funded by the Lipha Company**	RCT, double-blind<br><br>12 centres; all centres were psychiatric outpatient clinics; most of these clinics had specialized alcohol treatment facilities.<br><br>N = 272; 41.2 (SD 8.5); 22.4%	DSM-III-R and Munich Alcoholism Test<br>Patients had to be completely abstinent from any alcohol consumption for a minimum of 14 days and a maximum of 28 days and free of withdrawal symptoms before they could be admitted into the study. This period corresponded with the inpatient detoxification therapy period that included pharmacotherapy (mainly clomethiazole or benzodiazepines).	1. Placebo (N = 136)<br>2. Acamprosate: 1332-1998 mg/day, divided as TID Oral (N = 136)<br><br>48 weeks/48 weeks	Counselling or psychotherapy	N of DSM-III-R symptoms: 7.9 (SD 1.2)<br>Craving (VAS): 86.8 (SD 48.8)<br>Duration of alcoholism (year): 10.4 (6.2)<br><br>N of previous detoxifying treatment:<br>None: 30.1%<br>1-2: 43.8%<br>3-4: 13.6%<br>≥ 5: 12.5%	NR	Married: 46.5%<br>Living alone: 38%<br>Unemployed: 26.5%	PLA: 34/136<br>ACP: 61/136<br><br>PLA: 81/136<br>ACP: 57/136
**Schmidt 2002** [88 89]<br>**Germany**<br><br>**Supported by Deutsche Forschungsgemeinsch aft**	RCT, double-blind<br><br>Department of Psychiatry of the Free University of Berlin<br><br>N = 136; 45.3 (SD 8.1); 34.5%	ICD-10<br><br>Hospital detoxification	1. Placebo (N = 63*)<br>2. Lisuride: 1.0 mg/day (low-dose) and 1.8 mg/day (high-dose) with assignment ratio of 2:1 (N = 57*)<br><br>6 months/12 months<br><br>*ITT sample numbers used in the trial	Individual counselling and group therapy (one to two times every week)	N of fulfilled ICD-10 criteria of alcohol dependence before detoxification: 6.4 (1.4)<br><br>Age of onset of alcoholism (year): 34.2 (SD 9.5)<br>N of patients with previous detoxification: 66%	NR	Family history of alcoholism:<br>Living alone: 54.2%<br>Basic school level only:45.8%<br>Unemployed: 26.7%	PLA: 19/63<br>LUD: 8/57<br><br>PLA: 7/63<br>LUD: 10/57
**Stella 2008** [90]<br>**Italy**	RCT, open-label<br><br>NR<br><br>N = 47*; 41.8 (SD 12.0); 29.8%<br><br>*N = 48 enrolled	DSM-IV<br>Detoxification; metadoxine (900mg/day iv, divided into 3 administrations for 5 days) and diazepam (30–45 mg/day iv)	1. Escitalopram: 20 mg/day Oral (N = 12*)<br>2. Escitalopram 20mg/day + NTX 50 mg/day Oral (N = 12)<br>3. Escitalopram 20 mg/day + GHB 75 mg/kg/day, divided into five doses Oral (N = 12)<br>4. Escitalopram 20 mg/day + GHB 75 mg/kg/day + NTX 50 mg/day Oral (N = 12)<br><br>6 months<br><br>*one dropped out after detoxification	Counselling and supportive behavioural therapy	Duration of alcohol dependence (year): 12.4 (5.8)	NR	Married: 59.6%<br>Employed: 74.3%<br>Secondary school and above: 29.8%	EST: 2/12<br>EST+NTX: 4/12<br>EST+GHB: 6/12<br>EST+GHB+ NTX: 10/12<br><br>EST: 1/12<br>EST+NTX: 0/12<br>EST+GHB: 0/12<br>EST+GHB+ NTX: 0/12
**Tempesta 2000** [91-93]<br>**Italy**<br><br>**Funded by Lipha s.a., France**	RCT, double-blind<br><br>18 out-patient centres in Italy<br><br>N = 330; 45.9 (SD 11.2); 17.3%	DSM-III-R<br><br>Alcohol weaning therapy	1. Placebo (N = 166)<br>2. Acamprosate: 1998 mg/day divided as TID Oral (N = 164)<br><br>6 months/9 months	Individual behaviour-oriented supportive counselling (1–2 sessions/week, 1 h per session)<br>AA	Drinking history (year): 10.7 (SD 9.0)<br>MAST score: 22.7 (SD 10.6)<br>Previous treatment for alcoholism: 10%<br><br>Alcohol amount<br>≤ 5 drinks/day: 4.5%<br>5-10 drinks/day: 42.4%<br>> 10 drinks/day: 53%<br><br>Alcohol frequency<br>≤ 2days/week: 1.8%<br>3-6 days/week: 13%<br>Daily: 85.2%	NR	Married: 68.2%	PLA: 48/166<br>ACP: 62/164<br><br>PLA: 44/166<br>ACP: 40/164

**Ulrichsen 2010 [94]**<br>**Denmark**<br><br>**Supported by Trygfonden, Aase og Ejnar Danielsens Fond and The A.P. Møller Foundation**	RCT, open-label<br><br>Psychiatric Center Gentofte<br><br>N = 39; 52.0 (SD 10.1); 30.8%	ICD-10<br>Detoxification by phenobarbital 200 mg (hourly) or diazepam 20 mg if not tolerated	1. Control (TAU) (N = 20)<br>2. Disulfiram: 800 mg twice a week Oral (N = 19)<br><br>6 months	Cognitive behavioural therapy (CBT) programme	Age of first alcohol intake (year): 15.2 (SD 1.9)<br>Age of realizing to have an alcohol problem (years): 37.0 (SD 9.1)<br>Age of debut of withdrawal symptoms (years): 43.2 (SD 10.8)<br><br>Previous treatment for alcoholism:<br>GP: 56.4%<br>Minnesota treatment centre: 33.3%<br>AA meetings: 46.2%<br>Disulfiram: 76.9%<br>Acamprosate: 10.2%<br>Naltrexone: 10.2%<br>Never been treated: 15.4%	Previous treatment for depression: 43.6%<br>Current antidepressive drugs: 35.9%<br>Current benzodiazepines: 7.7%<br>Current nicotine intake: 67.7%<br>Ever tried to take illegal drugs: 69.2%<br>Ever had abused illegal drugs: 15.4%<br><br>Affective disorders: 17.9%<br>Anxiety disorders: 28.2%<br>Personality disorders: 2.6%<br>Hyperkinetic disorders: 2.6%	Married: 38.5%<br>Employed: 43.6%<br>High school and above: 82.1%	TAU: 4/20<br>DSF: 5/19<br><br>TAU: 10/20<br>DSF: 12/19
**Volpicelli 1997 [95]**<br>**USA**<br><br>**Supported by NIAAA**	RCT, single-blind<br><br>University of Pennsylvania/Veterans Affairs Treatment Research Centre<br><br>N = 97; 38.4 (SD 8.7); 22.7%	DSM-III-R<br><br>Detoxification for alcohol withdrawal	1. Placebo (N = 49)<br>2. Naltrexone: 50 mg/day Oral (N = 48)<br><br>12 weeks	Counselling consisted of individual psychotherapy modified after Gorski and Miller's relapse prevention program	Years of regular drinking: 15.4 (SD 9.1)<br>Baseline drinking days: 14.1 (SD 8.9)	NR	Non-white: 62.6%<br>Employed: 67.7%<br>Married: 44.5%	PLA: 17/49<br>NTX: 21/48<br><br><br>PLA: 13/49<br>NTX: 13/48
**Wetzel 2004 [96]**<br>**Germany**<br><br>**Supported by a grant from Bundesministerium für Bildung und Forschung (BMBF) and Bristol-Myers Squibb.**	RCT, double-blind<br><br>3 university sites in Germany (Departments of Psychiatry at the Universities of Mainz, Rostock, and Homburg/Saar)<br><br>N = 242; 42.8 (8.4); 0%	DSM-IV and ICD-10<br><br>In-patient detoxification	1. Nefazodone + CBT: 200 mg/day initially, increased to 600 mg/day (N = 53)<br>2. Nefazodone + Group counselling (GC): 200 mg/day initially, increased to 600 mg/day (N = 50)<br>3. Placebo + CBT (N = 50)<br>4. Placebo + GC (N = 47)<br><br>CBT: 24 group therapy sessions, with 6 sessions within the first 2 weeks, followed by 10 sessions during week 3 and week 4 and weekly sessions thereafter until week 12.<br>GC: 24 sessions of a nonspecific group intervention to facilitate insight, self-help potentials, and support. The theoretical background was nondirective and client-oriented, with the therapist acting as moderator of the group discussion.<br><br>12 weeks/12 months	NR	N of DSM-IV criteria: 6.1 (SD 0.9)<br><br>Drinking days in previous 90 day (%): 70.8 (31.1)<br>N of drinks per drinking day in previous 90 days: 14.7 (SD 8.9)<br><br>Age when started getting intoxicated regularly (year): 19.0 (SD 5.8)<br>Age when first had difficulty stopping before intoxication (year): 26.3 (SD 9.9)	Smoker: 82.3%<br><br>Lifetime DSM-IV diagnosis (%):<br>Major depression: 18.9%<br>Social phobia: 8.1%<br>Generalized anxiety disorder: 0.5%<br>Substance use disorder: 4.9%<br>Antisocial personality disorder: 7.7%	Married: 58.9%<br>Education (year): 9.8 (SD 1.5)<br><br>History of paternal alcoholism: 31.0%<br>History of alcoholism in first-degree relatives: 48.5%	PLA: 13/47<br>PLA+CBT: 12/50<br>NZD: 9/50<br>NZD+CBT: 12/53<br><br>PLA: 31/47<br>PLA+CBT: 33/50<br>NZD: 38/50<br>NZD+CBT: 36/53
**Whitworth 1996 [97]**<br>**Austria**<br><br>**1989 to 1993**<br>**Funded by Groupe LIPHA**	RCT, double-blind<br><br>multicentre, Hospital<br><br>N = 455; 42.0 (SD 8.5); 21.2%	DSM-III<br><br>Alcohol-withdrawal treatment and minimal 5-day abstinence	1. Placebo (N = 224*)<br>2. Acamprosate: 1332-1998 mg, adjusted by weight. BW >60 kgs: 1998 mg/day; BW ≤ 60 kgs: 1332 mg/day (N = 224*)<br><br>360 days/720 days<br><br>*ITT sample numbers used in the trial	NR	MAST: 32.6 (SD 8.7)<br>Daily alcohol consumption (g)<br>≤59: 6%<br>60-120: 31.3%<br>≥121: 62.7%	NR	NR	PLA: 16/224 ACP: 41/224<br><br>PLA: 139/224<br>ACP: 129/224
**Wiesbeck 2001 [98,99]**<br>**Germany and Austria**<br><br>**Jun 1994 to Mar 1998**<br>**Funded by the Bayer**	RCT, double-blind<br><br>multi-centre<br><br>N = 281; 41.7 (SD 7.8); 27.4%	DSM-II-R and Munich Alcoholism Test (MALT)<br><br>Detoxification and 14-42 days of abstinence	1. Placebo (N = 139)<br>2. Flupenthixol: 10 mg every two weeks IM (N = 142)<br><br>6 months/12 months	Supportive psychotherapy self-help support groups (AA)	DSM-III-R criteria for dependence: 8.1 (SD 1.0)<br>Munich Alcoholism Test (MALT): 33.5 (SD 5.8)<br>Goettinger Dependence Scale, GABS (German SADQ): 58.0 (SD 17.8)<br>VAS: 13.7 (SD 21.8)<br><br>Alcohol intake before detoxification: 260.0 (SD 152)	Social functioning (SFQ): 14.8 (SD 3.9)	NR	PLA: 58/139<br>FLP: 34/142<br><br>PLA: 81/139<br>FLP: 109/142

Abbreviations: AA: Alcoholics Anonymous, ACP: Acamprosate, AD: Alcohol Dependence, ADS: Alcohol Dependence Scale, AMS: Amisulpride, ARI: Aripiprazole, ATL: Atenolol, BAC: Baclofen, BZD: Benzodiazepines, CBZ: Carbamazepine, CIT: Citalopram, CST: Cognitive Stimulation Therapy, DSF: Disulfiram, EST: Escitalopram, FLP: Flupenthixol, FLT: Fluoxetine, FLX: Fluvoixamine, GAL: Galantamine, GHB: GHB (sodium oxybate), GSI: General Symptom Index, HAM-A: Hamilton Anxiety Rating Scale, HAM-D: Hamilton Depression Rating Scale, HDS: Hamilton Depression Scale, HOV: Home visit, IM: Intramuscular, LEV: Levetiracetam, LIT: Lithium, LUD: Lisuride, MAST: Michigan Alcoholism Screening Test, MCS: Mental component scores, MDF: Modafinil, MDMA: Methylenedioxymethamphetamine , MET: Motivational Enhancement Therapy, NTX: Naltrexone , NZD: Nefazodone, OCB: Oxcarbazepine, OCDS: Obsessive Compulsive Drinking Scale, OCDS-G: Obsessive Compulsive Drinking Scale German version, PCS: Physical component scores, PGB: Pregablin, QTP: Quetiapine, SADD: Short Alcohol Dependence Data, SADQ: Alcohol Dependence Data Questionnaire, sCBT: Short-form CBT, SCL-90-R : Symptom Checklist-90-Revised, SES: Socioeconomic status, SF-36: Short Form (36) Health Surve, TAU: Treat as usual, TID: Three time a day, TIP: Tianeptine, TPD: Tiapride, TPM: Topiramate, TZD: Trazodone , VAS: Visual analogue scale.

References

1. Angelone SMB, L.; Bella, D.; Catalano, M. Effects of fluvoxamine and citalopram in maintaining abstinence in a sample of Italian detoxified alcoholics. *Clinical Trial; Comparative Study; Journal Article; Randomized Controlled Trial* 1998;33(2):151-56.
2. Muller CAG, O.; Pelz, P.; Higl, V.; Kruger, J.; Stickel, A.; Beck, A.; Wernecke, K. D.; Hellweg, R.; Heinz, A. High-dose baclofen for the treatment of alcohol dependence (BACLAD study): A randomized, placebo-controlled trial. *European neuropsychopharmacology* 2015;25(8):1167-77.
3. Baltieri DAA, A. G. Acamprosate in alcohol dependence: a randomized controlled efficacy study in a standard clinical setting. *Clinical Trial; Comparative Study; Journal Article; Multicenter Study; Randomized Controlled Trial* 2004;65(1):136-39.
4. Baltieri DAA, A. G. Efficacy of acamprosate in the treatment of alcohol-dependent outpatients. *Clinical Trial; Journal Article; Randomized Controlled Trial; Research Support, Non-US Gov't* 2003;25(3):156-59.
5. Baltieri DAD, F. R.; Ribeiro, P. L.; Andrade, A. G. Comparing topiramate with naltrexone in the treatment of alcohol dependence. *Addiction (Abingdon, England)* 2008;103(12):2035-44.
6. Baltieri DAD, F. R.; Ribeiro, P. L.; Andrade, A. G. Effects of topiramate or naltrexone on tobacco use among male alcohol-dependent outpatients. *Clinical Trial; Comparative Study; Journal Article; Multicenter Study; Randomized Controlled Trial; Research Support, Non-US Gov't* 2009;105(1-2):33-41.
7. Barrias JC, S.; Ferreira, L.; Fonte, A.; Potgieter, A.; Teixeira de Sousa, E. Acamprosate: multicenter Portuguese efficacy and tolerance evaluation study [Portuguese]. *Psiquiatria Clinica* 1997;18:149-60.
8. Bender SS, N.; Soyka, M.; Ruther, E.; Mann, K.; Gastpar, M. The efficacy of the dopamine D2/D3 antagonist tiapride in maintaining abstinence: a randomized, double-blind, placebo-controlled trial in 299 alcohol-dependent patients. *Clinical Trial; Journal Article; Multicenter Study; Randomized Controlled Trial; Research Support, Non-US Gov't* 2007;10(5):653-60.
9. Besson JA, F.; Kasas, A.; Lehert, P.; Potgieter, A. Combined efficacy of acamprosate and disulfiram in the treatment of alcoholism: a controlled study. *Clinical Trial; Comparative Study; Journal Article; Multicenter Study; Randomized Controlled Trial; Research Support, Non-US Gov't* 1998;22(3):573-79.
10. Burtscheidt WW, W.; Schwarz, R.; Strauss, W.; Gaebel, W. Out-patient behaviour therapy in alcoholism: treatment outcome after 2 years. *Clinical Trial; Journal Article; Randomized Controlled Trial; Research Support, Non-US Gov't* 2002;106(3):227-32.
11. Burtscheidt WW, W.; Schwarz, R.; Strauss, W.; Lo?=ll, A.; Lo?=thcke, H.; Redner, C.; Gaebel, W. Out-patient behaviour therapy in alcoholism: relapse rates after 6 months. *Clinical Trial; Journal Article; Randomized Controlled Trial; Research Support, Non-US Gov't* 2001;103(1):24-29.
12. Wolwer WB, W.; Redner, C.; Schwarz, R.; Gaebel, W. Out-patient behaviour therapy in alcoholism: impact of personality disorders and cognitive impairments. *Clinical Trial; Journal Article; Randomized Controlled Trial; Research Support, Non-US Gov't* 2001;103(1):30-37.
13. Caputo FA, G.; Lorenzini, F.; Domenicali, M.; Greco, G.; Rea,; Gasbarrini, G.; Stefanini, G. F.; Bernardi, M. Gamma-hydroxybutyric acid versus naltrexone in maintaining alcohol abstinence: an open randomized comparative study. *Clinical Trial; Comparative Study; Journal Article; Multicenter Study; Randomized Controlled Trial; Research Support, Non-US Gov't* 2003;70(1):85-91.
14. Caputo FA, G.; Stoppo, M.; Francini, S.; Vignoli, T.; Lorenzini, F.; Re, A.; Comaschi, C.; Andreone, P.; Trevisani, F.; Bernardi, M. Comparing and combining gamma-hydroxybutyric acid (GHB) and naltrexone in maintaining abstinence from alcohol: an open randomised comparative study. *Journal: Article* 2007;17(12):781-89.
15. Chick JH, H.; Morgan, M. Y.; Ritson, B. United Kingdom Multicentre Acamprosate Study (UKMAS): a 6-month prospective study of acamprosate versus placebo in preventing relapse after withdrawal from alcohol. *Clinical Trial; Comparative Study; Journal Article; Randomized Controlled Trial* 2000;35(2):176-87.
16. Chick JA, H.; Hornik, K. Efficacy of fluvoxamine in preventing relapse in alcohol dependence: a one-year, double-blind, placebo-controlled multicentre study with analysis by typology. *Clinical Trial; Comparative Study; Journal Article; Multicenter Study; Randomized Controlled Trial; Research Support, Non-US Gov't* 2004;74(1):61-70.

17. Meszaros KL, E.; Hornik, K.; Fureder, T.; Willinger, U.; Fischer, G.; Schonbeck, G.; Aschauer, H. N. The Tridimensional Personality Questionnaire as a predictor of relapse in detoxified alcohol dependents. The European Fluvoxamine in Alcoholism Study Group. *Clinical Trial; Comparative Study; Journal Article; Multicenter Study; Randomized Controlled Trial; Research Support, Non-US Gov't* 1999;23(3):483-86.
18. Meszaros KW, U.; Fischer, G.; Scho?=nbeck, G.; Aschauer, H. N. The tridimensional personality model: influencing variables in a sample of detoxified alcohol dependents. European Fluvoxamine in Alcoholism Study Group. *Clinical Trial; Journal Article; Multicenter Study; Randomized Controlled Trial; Research Support, Non-US Gov't* 1996;37(2):109-14.
19. Willinger UL, E.; Hornik, K.; Fischer, G.; Scho?=nbeck, G.; Aschauer, H. N.; Meszaros, K. Anxiety as a predictor of relapse in detoxified alcohol-dependent patients. *Clinical Trial; Comparative Study; Journal Article; Randomized Controlled Trial* 2002;37(6):609-12.
20. Cornelius JRS, I. M.; Ehler, J. G.; Jarrett, P. J.; Cornelius, M. D.; Perel, J. M.; Thase, M. E.; Black, A. Fluoxetine in depressed alcoholics. A double-blind, placebo-controlled trial. *Clinical Trial; Comparative Study; Journal Article; Randomized Controlled Trial; Research Support, Non-US Gov't* 1997;54(8):700-05.
21. Cornelius JRS, I. M.; Haskett, R. F.; Daley, D. C.; Cornelius, M. D.; Thase, M. E.; Perel, J. M. Fluoxetine versus placebo in depressed alcoholics: a 1-year follow-up study. *Clinical Trial; Journal Article; Randomized Controlled Trial; Research Support, US Gov't, PHS* 2000;25(2):307-10.
22. Cornelius JRS, I. M.; Haskett, R. F.; Ehler, J. G.; Jarrett, P. J.; Thase, M. E.; Perel, J. M. Fluoxetine versus placebo for the marijuana use of depressed alcoholics. *Clinical Trial; Journal Article; Randomized Controlled Trial; Research Support, US Gov't, PHS* 1999;24(1):111-14.
23. Coriale GDR, F.; Battagliese, G.; Gencarelli, S.; Fiore, M.; Ferraguti, G.; Vitali, M.; Rotondo, C.; Messina, M. P.; Attilia, M. L.; Ceccanti, M. Motivational enhancement therapy versus cognitive behavioral therapy in a cohort of men and women with alcohol use disorder. *Biomedical Reviews* 2019;30:125-35.
24. Croissant BD, A.; Klein, O.; Zambrano, S.; Nakovics, H.; Heinz, A.; Mann, K. A pilot study of oxcarbazepine versus acamprosate in alcohol-dependent patients *Clinical Trial; Comparative Study; Journal Article; Multicenter Study; Randomized Controlled Trial; Research Support, Non-US Gov't* 2006;30(4):630-35.
25. Fuente JRM, R. M.; Niven, R. G.; Ilstrup, D. M. A controlled study of lithium carbonate in the treatment of alcoholism. *Clinical Trial; Controlled Clinical Trial; Journal Article; Research Support, Non-US Gov't* 1989;64(2):177-80.
26. Favre JDG-S, C.; Delalleau, B.; Loo, H. Tianeptine and alcohol dependence. *Journal: Article* 1997;7 Suppl 3:S347-51.
27. Favre JDL, H.; Marey, C.; Delalleau, B. Long-term efficacy of tianeptine on alcoholic relapses in non-depressed alcoholic patients. Preliminary results. *European psychiatry* 1993;8(SUPPL. 2):125S-29S.
28. Florez GG-P, P.; Alvarez, S.; Saiz, P. A.; Nogueiras, L.; Bobes, J. Using topiramate or naltrexone for the treatment of alcohol-dependent patients. *Clinical Trial; Comparative Study; Journal Article; Multicenter Study; Randomized Controlled Trial; Research Support, Non-US Gov't* 2008;32(7):1251-59.
29. Florez GSP, A.; Garcia-Portilla, Paz; Alvarez, Sandra; Nogueiras, Luis; Bobes, Julio. Topiramate for the treatment of alcohol dependence: Comparison with naltrexone. *European addiction research* 2010;17(1):29-36. doi: http://dx.doi.org/10.1159/000320471
30. Friedmann PDR, J. S.; Swift, R.; Stout, R. L.; Millman, R. P.; Stein, M. D. Trazodone for sleep disturbance after alcohol detoxification: a double-blind, placebo-controlled trial. *Clinical Trial; Comparative Study; Journal Article; Multicenter Study; Randomized Controlled Trial; Research Support, Non-US Gov't* 2008;32(9):1652-60.
31. Fuller RKB, L.; Brightwell, D. R.; Derman, R. M.; Emrick, C. D.; Iber, F. L.; James, K. E.; Lacoursiere, R. B.; Lee, K. K.; Lowenstam, I. Disulfiram treatment of alcoholism. A Veterans Administration cooperative study. *Clinical Trial; Journal Article; Randomized Controlled Trial; Research Support, US Gov't, Non-PHS* 1986;256(11):1449-55.
32. Branchey LD, W.; Lee, K. K.; Fuller, R. K. Psychiatric complications of disulfiram treatment. *Clinical Trial; Controlled Clinical Trial; Journal Article; Research Support, US Gov't, Non-PHS* 1987;144(10):1310-12.
33. Cacciaglia RL, O. M.; Vivet, P. Gate 2 study: Sodium oxybate in the maintenance of alcohol abstinence and prevention of alcohol relapse [Conference]. *Alcohol and alcoholism* 2013;48:i47-i48.
34. Caputo FS, K.; Walter, H.; Ceccanti, M.; Djurkowski, M.; Filipecka, E.; Florkowski, A.; Gerra, G.; Holzbach, R.; Horodnicki, J.; Platz, W.; Spazzapan, B.; Zblowska, H.; Bernardi, M.; Cacciaglia, R.; Vivet, P.; Lesch, O. M.; Addolorato, G. Sodium oxybate in the prevention of alcohol relapses in alcohol dependent patients (gate 2 study) [Conference]. *Alcohol and alcoholism* 2013;Conference: 14th Congress of the European Society for Biomedical Research on Alcoholism, ESBRA 2013. Warsaw Poland. Conference Start: 20130908. Conference End: 20130911. Conference Publication:(var.pagings):i34. doi: http://dx.doi.org/10.1093/alcalc/agt101
35. Skala KC, F.; Mirijello, A.; Vassallo, G.; Antonelli, M.; Ferrulli, A.; Walter, H.; Lesch, O.; Addolorato, G. Sodium oxybate in the treatment of alcohol dependence: From the alcohol withdrawal syndrome to the alcohol relapse prevention. *Expert opinion on pharmacotherapy* 2014;15(2):245-57. doi: http://dx.doi.org/10.1517/14656566.2014.863278

36. Geerlings PJA, C.; d, B. W. Acamprosate and prevention of relapse in alcoholics. Results of a randomized, placebo-controlled, double-blind study in out-patient alcoholics in the Netherlands, Belgium and Luxembourg. *European addiction research* 1997;3(3):129-37.
37. Gottlieb LDH, R. I.; Kraus, M. L.; Segal, S. R.; Viscoli, C. M. Randomized controlled trial in alcohol relapse prevention: role of atenolol, alcohol craving, and treatment adherence. *Clinical Trial; Journal Article; Randomized Controlled Trial; Research Support, Non-US Gov't* 1994;11(3):253-58.
38. Gual AL, P. Acamprosate during and after acute alcohol withdrawal: a double-blind placebo-controlled study in Spain. *Clinical Trial; Comparative Study; Journal Article; Randomized Controlled Trial* 2001;36(5):413-18.
39. Gual AM, M.; Ortega, Ll. Efficacy of tiapride in the maintenance of abstinence in weaned alcoholics. Results of a double blind trial against placebo. [Spanish]. *Adicciones* 2002;14(3):321-26.
40. Gustafson DHM, F. M.; Chih, M. Y.; Atwood, A. K.; Johnson, R. A.; Boyle, M. G.; Levy, M. S.; Driscoll, H.; Chisholm, S. M.; Dillenburg, L.; Isham, A.; Shah, D. A smartphone application to support recovery from alcoholism: a randomized clinical trial. *Journal Article; Multicenter Study; Randomized Controlled Trial; Research Support, NIH, Extramural* 2014;71(5):566-72.
41. Chih MY. Exploring the use patterns of a mobile health application for alcohol addiction before the initial lapse after detoxification [Conference]. *Journal Article; Randomized Controlled Trial* 2014;2014:385-94.
42. Huang XH, X.; Peng, H.; Mai, G. Placebo-controlled trial of naltrexone in outpatient treatment of alcohol dependence [Mandarin]. *Chinese mental health journal* 2002;16(5):302-03.
43. Huang MCC, C. H.; Yu, J. M.; Chen, C. C. A double-blind, placebo-controlled study of naltrexone in the treatment of alcohol dependence in Taiwan. *Addiction biology* 2005;10(3):289-92.
44. Janiri LH, A.; Lombardi, U.; Rago, R.; Mannelli, P.; Tempesta, E. SSRIs in alcoholism: Fluvoxamine vs fluoxetine in alcoholic outpatients [Conference]. *Sixth world congress of biological psychiatry, nice, france June 22-27, 1997* 1997;42(1):35S.
45. Jirapramukpitak TP, K.; Chua, K. C.; Takizawa, P. Home-Based Contingency Management Delivered by Community Health Workers to Improve Alcohol Abstinence: A Randomized Control Trial. *Alcohol Alcohol* 2020;9:09. doi: 10.1093/alcalc/agz106 [published Online First: 2020/01/11]
46. Joos LG, A. E.; Schmaal, L.; Fransen, E.; Brink, W.; Sabbe, B. G.; Dom, G. Effect of modafinil on impulsivity and relapse in alcohol dependent patients: a randomized, placebo-controlled trial. *Journal: Article* 2013;23(8):948-55.
47. Kampman KMP, H. M.; Lynch, K. G.; Whittingham, T.; Macfadden, W.; Dackis, C.; Tirado, C.; Oslin, D. W.; Sparkman, T.; O'Brien, C. P. A double-blind, placebo-controlled pilot trial of quetiapine for the treatment of Type A and Type B alcoholism. *Journal Article; Multicenter Study; Randomized Controlled Trial* 2007;27(4):344-51.
48. Kampman KMP, H. M.; Macfadden, W.; Lynch, K. G.; Whittingham, T. A pilot trial of quetiapine for the treatment of alcohol dependence [Conference]. *Proceedings of the 68th annual scientific meeting of the college on problems of drug dependence; 2006 june 17-22; scottsdale, arizona, USA* 2006
49. Kiefer FJ, H.; Tarnaske, T.; Helwig, H.; Briken, P.; Holzbach, R.; Ko?=mpf, P.; Stracke, R.; Baehr, M.; Naber, D.; Wiedemann, K. Comparing and combining naltrexone and acamprosate in relapse prevention of alcoholism: a double-blind, placebo-controlled study. *Clinical Trial; Comparative Study; Journal Article; Randomized Controlled Trial; Research Support, Non-US Gov't* 2003;60(1):92-99.
50. Kiefer FH, H.; Tarnaske, T.; Otte, C.; Jahn, H.; Wiedemann, K. Pharmacological relapse prevention of alcoholism: clinical predictors of outcome. *European addiction research* 2005;11(2):83-91.
51. Kiefer FAFO, C.; Wolf, K.; Jahn, H.; Wiedemann, K. Long-term effects of pharmacotherapy on relapse prevention in alcohol dependence. *Acta neuropsychiatrica* 2004;16(5):233-38.
52. Kiefer FJ, H.; Holzbach, R.; Briken, P.; Stracke, R.; Wiedemann, K. The NALCAM-study: Efficacy, tolerability, outcome. [German]. *Sucht* 2003;49(6):342-51.
53. Kiefer FJ, H.; Briken, P.; Ko?=mpf, P.; Holzbach, R.; Naber, D.; Wiedemann, K. Naltrexone versus acamprosate in the relapse prevention of alcoholism: A randomized placebo controlled trial [Conference]. *Journal: Article* 2002;12 Suppl 3:S391.
54. Ladewig DK, T.; Leher, P.; Fendl, A. [Acamprosate--a stabilizing factor in long-term withdrawal of alcoholic patients] [German]. *Clinical Trial; English Abstract; Journal Article; Randomized Controlled Trial* 1993;50(3):182-88.
55. Landabaso MAI, I.; Sanz, J.; Calle, R.; Ruiz De Apodaka, J.; Jimenez-Lerma, J. M.; Gutierrez-Fraile, M. Naltrexone in the treatment of alcoholism. Two-year follow up results. *European journal of psychiatry* 1999;13(2):97-105.

56. Mann KA, K.; Diehl, A.; Ebert, D.; Mundle, G.; Nakovics, H.; Reker, T.; Richter, G.; Schmidt, L. G.; Driessen, M.; Rettig, K.; Opitz, K.; Croissant, B. Galantamine: a cholinergic patch in the treatment of alcoholism: a randomized, placebo-controlled trial. *Journal Article; Multicenter Study; Randomized Controlled Trial* 2006;184(1):115-21.
57. Marra DW, D.; Berlin, I.; Hispard, E.; Notides, C.; Tilikete, S.; Payan, C.; Lo?=pine, J. P.; Dally, S.; Aubin, H. J. Amisulpride does not prevent relapse in primary alcohol dependence: results of a pilot randomized, placebo-controlled trial. *Clinical Trial; Comparative Study; Journal Article; Multicenter Study; Randomized Controlled Trial; Research Support, Non-US Gov't* 2002;26(10):1545-52.
58. Martinotti GN, M.; Romanelli, R.; Andreoli, S.; Pozzi, G.; Moroni, N.; Janiri, L. High and low dosage oxcarbazepine versus naltrexone for the prevention of relapse in alcohol-dependent patients. *Clinical Trial; Comparative Study; Journal Article; Randomized Controlled Trial* 2007;22(3):149-56.
59. Martinotti GN, M.; Giannantonio, M.; Janiri, L. Aripiprazole in the treatment of patients with alcohol dependence: a double-blind, comparison trial vs. naltrexone. *Comparative Study; Journal Article; Randomized Controlled Trial* 2009;23(2):123-29.
60. Martinotti GN, M.; Tedeschi, D.; Andreoli, S.; Reina, D.; Pomponi, M.; Mazza, M.; Romanelli, R.; Moroni, N.; Filippis, R.; Giannantonio, M.; Pozzi, G.; Bria, P.; Janiri, L. Pregabalin versus naltrexone in alcohol dependence: a randomised, double-blind, comparison trial. *Journal of psychopharmacology (Oxford, England)* 2010;24(9):1367-74.
61. Martinotti GDN, M.; Tedeschi, D.; Guglielmo, R.; Janiri, L. Pregabalin versus naltrexone in alcohol dependence: Results from a multicenter, rondomized, double-blind, comparison trial [Conference]. *Journal: Article* 2008;Conference: 21st ECNP Congress. Barcelona Spain. Conference Start: 20080830. Conference End: 20080903. Conference Publication:(var.pagings):S523-S24.
62. Matching Alcoholism Treatments to Client Heterogeneity: Project MATCH posttreatment drinking outcomes. *Clinical Trial; Comparative Study; Journal Article; Multicenter Study; Randomized Controlled Trial* 1997;58(1):7-29.
63. Matching alcoholism treatments to client heterogeneity: treatment main effects and matching effects on drinking during treatment. Project MATCH Research Group. *Clinical Trial; Comparative Study; Journal Article; Multicenter Study; Randomized Controlled Trial* 1998;59(6):631-39.
64. Project MATCH (Matching Alcoholism Treatment to Client Heterogeneity): rationale and methods for a multisite clinical trial matching patients to alcoholism treatment. *Clinical Trial; Comparative Study; Journal Article; Multicenter Study; Randomized Controlled Trial; Research Support, Non-US Gov't* 1993;17(6):1130-45.
65. Allen JA, R. F.; Babor, T. F.; Carbonari, J.; Carroll, K. M.; Connors, G. J.; Cooney, N. L.; Del, B. F. K.; DiClemente, C. C.; Donovan, D.; Kadden, R. M.; Litt, M.; Longabaugh, R.; Mattson, M.; Miller, W. R.; Randall, C. L.; Rounsaville, B. J.; Rychtarik, R. G.; Stout, R. L.; Tonigan, J. S.; Wirtz, P. W.; Zweben, A. Project MATCH secondary a priori hypotheses. Project MATCH Research Group. *Comparative Study; Journal Article; Randomized Controlled Trial; Research Support, Non-US Gov't* 1997;92(12):1671-98.
66. Comments on project MATCH: Matching alcohol treatments to client heterogeneity. *Addiction (Abingdon, England)* 1999;94(1):31-34. doi: http://dx.doi.org/10.1080/09652149934152
67. Moncini MM, E.; Gambassi, F.; Mannaioni, P. F. Gamma-hydroxybutyric acid and alcohol-related syndromes. *Alcohol (Fayetteville, NY)* 2000;20(3):285-91.
68. Bello MGG, F.; Mugnai, L.; Masini, E.; Mannaioni, P. F. Gamma-hydroxybutyric acid induced suppression and prevention of alcohol withdrawal syndrome and relief of craving in alcohol dependent patients. *Alcologia* 1995;7(2):111-18.
69. Moraes EC, G. M.; Figlie, N. B.; Ferraz, M. B.; Laranjeira, R. Home visits in the outpatient treatment of individuals dependent on alcohol: Randomized clinical trial. *Addictive disorders & their treatment* 2010;9(1):18-31.
70. Moraes EC, G. M.; Figlie, N. B.; Laranjeira, R.; Ferraz, M. B. Cost-effectiveness of home visits in the outpatient treatment of patients with alcohol dependence. *European addiction research* 2010;16(2):69-77.
71. Mueller TIS, R. L.; Rudden, S.; Brown, R. A.; Gordon, A.; Solomon, D. A.; Recupero, P. R. A double-blind, placebo-controlled pilot study of carbamazepine for the treatment of alcohol dependence. *Clinical Trial; Comparative Study; Journal Article; Multicenter Study; Randomized Controlled Trial; Research Support, Non-US Gov't* 1997;21(1):86-92.
72. Oslin DW. Treatment of late-life depression complicated by alcohol dependence. *Journal Article; Randomized Controlled Trial; Research Support, NIH, Extramural; Research Support, US Gov't, Non-PHS; Research Support, US Gov't, PHS* 2005;13(6):491-500.
73. Paille FMG, J. D.; Perkins, A. C.; Royer, R. J.; Steru, L.; Parot, P. Double-blind randomized multicentre trial of acamprosate in maintaining abstinence from alcohol. *Clinical Trial; Comparative Study; Journal Article; Randomized Controlled Trial* 1995;30(2):239-47.

74. Pelc IB, O.; Verbanck, P.; Lehert, P.; Opsome, L. Calcium acetyl homotaurinate for maintaining abstinence in weaned alcoholic patients: a placebo-controlled double-blind multi-centre study. *Novel pharmacological interventions for alcoholism* 1992:348-52.
75. Pelc IV, P.; Bon, O.; Gavrilovic, M.; Lion, K.; Lehert, P. Efficacy and safety of acamprosate in the treatment of detoxified alcohol-dependent patients. A 90-day placebo-controlled dose-finding study. *Clinical Trial; Journal Article; Multicenter Study; Randomized Controlled Trial; Research Support, Non-US Gov't* 1997;171:73-77.
76. Pelc IH, C.; Baert, I.; Houtain, C.; Lehert, P.; Landron, F.; Verbanck, P. Effect of community nurse follow-up when treating alcohol dependence with acamprosate. *Clinical Trial; Comparative Study; Journal Article; Randomized Controlled Trial* 2005;40(4):302-07.
77. Poldrugo F. Acamprosate treatment in a long-term community-based alcohol rehabilitation programme. *Comparative Study; Journal Article; Randomized Controlled Trial; Research Support, Non-US Gov't* 1997;92(11):1537-46.
78. Ponce GS-G, J.; Rubio, G.; Rodriguez-Jimenez, R.; Jimenez-Arriero, M. A.; Palomo, T. [Efficacy of naltrexone in the treatment of alcohol dependence disorder in women] [Spanish]. *Clinical Trial; English Abstract; Journal Article; Randomized Controlled Trial* 2005;33(1):13-18.
79. Mann KL, Tagrid; Hoffmann, Sabine; Reinhard, Iris; Hermann, Derik; Batra, Anil; Berner, Michael; Wodarz, Norbert; Heinz, Andreas; Smolka, Michael N.; Zimmermann, Ulrich S.; Wellek, Stefan; Kiefer, Falk; Anton, Raymond F.; Team, Predict Study. Results of a double-blind, placebo-controlled pharmacotherapy trial in alcoholism conducted in Germany and comparison with the US COMBINE study. *Addiction Biology* 2013;18(6):937-46. doi: 10.1111/adb.12012
80. NCT00317031. Individually Adapted Therapy of Alcoholism. https://ClinicalTrialsgov/show/NCT00317031 2002
81. Mann KK, F.; Smolka, M.; Gann, H.; Wellek, S.; Heinz, A. Searching for responders to acamprosate and naltrexone in alcoholism treatment: rationale and design of the PREDICT study. *Clinical Trial; Comparative Study; Journal Article; Multicenter Study; Randomized Controlled Trial; Research Support, Non-US Gov't* 2009;33(4):674-83.
82. Mann KVd-K, S.; Reinhard, I.; Lemo?=nager, T.; Fauth-Bo?=hler, M.; Hermann, D.; Hoffmann, S.; Zimmermann, U. S.; Kiefer, F.; Heinz, A.; Smolka, M. N. Predicting naltrexone response in alcohol-dependent patients: the contribution of functional magnetic resonance imaging. *Clinical Trial; Comparative Study; Journal Article; Multicenter Study; Randomized Controlled Trial; Research Support, Non-US Gov't* 2014;38(11):2754-62.
83. Richter CE, S.; Bschor, T.; Bonnet, U.; Haasen, C.; Preuss, U. W.; Heinz, A.; Fo?=rg, A.; Volkmar, K.; Glauner, T.; Schaefer, M. Efficacy and safety of levetiracetam for the prevention of alcohol relapse in recently detoxified alcohol-dependent patients: a randomized trial. *Journal Article; Multicenter Study; Randomized Controlled Trial* 2012;32(4):558-62.
84. Rubio GP, G.; Rodriguez-Jimenez, R.; Jimenez-Arriero, M. A.; Hoenicka, J.; Palomo, T. Clinical predictors of response to naltrexone in alcoholic patients: who benefits most from treatment with naltrexone? *Clinical Trial; Comparative Study; Journal Article; Randomized Controlled Trial* 2005;40(3):227-33.
85. Sass HS, M.; Mann, K.; Zieglgo?=nsberger, W. Relapse prevention by acamprosate. Results from a placebo-controlled study on alcohol dependence. *Clinical Trial; Comparative Study; Journal Article; Randomized Controlled Trial; Research Support, Non-US Gov't* 1996;53(8):673-80.
86. Soyka MS, H. Acamprosate: a new pharmacotherapeutic approach to relapse prevention in alcoholism--preliminary data. *Clinical Trial; Journal Article; Multicenter Study; Randomized Controlled Trial* 1994;2:531-36.
87. Sass HS, M.; Mann, K.; W, Zieglgänsberger. Relapse prevention by acamprosate. Results from a placebo-controlled study on alcohol dependence [erratum appears in Arch Gen Psychiatry 1996;53(12):1097]. *Clinical Trial; Comparative Study; Journal Article; Randomized Controlled Trial; Research Support, Non-US Gov't* 1996;53(8):673-80.
88. Schmidt LGK, S.; Smolka, M.; Schmidt, K.; Rommelspacher, H. Lisuride, a dopamine D2 receptor agonist, and anticraving drug expectancy as modifiers of relapse in alcohol dependence. *Clinical Trial; Journal Article; Randomized Controlled Trial; Research Support, Non-US Gov't* 2002;26(2):209-17.
89. Schmidt LGD, P.; Kuhn, S.; Rommelspacher, H. Relapse prevention in alcoholics with an anticraving drug treatment: first results of the Berlin Study. *Clinical Trial; Journal Article; Randomized Controlled Trial* 1994;27 Suppl 1:21-23.
90. Stella LA, G.; Rinaldi, B.; Capuano, A.; Berrino, L.; Rossi, F.; Maione, S. An open randomized study of the treatment of escitalopram alone and combined with gamma-hydroxybutyric acid and naltrexone in alcoholic patients. *Journal Article; Randomized Controlled Trial* 2008;57(4):312-17.
91. Tempesta EJ, L.; Bignamini, A.; Chabac, S.; Potgieter, A. Acamprosate and relapse prevention in the treatment of alcohol dependence: a placebo-controlled study. *Clinical Trial; Comparative Study; Journal Article; Randomized Controlled Trial* 2000;35(2):202-09.
92. Tempesta EJ, L.; Bignamini, A.; Chabac, S.; Potgieter, A. Acamprosate in alcohol dependence: a placebo-controlled study in a comprehensive post- detoxification program [Conference]. *9th congress of the association of european psychiatrists Copenhagen, denmark 20-24th september 1998* 1998

93. Tempesta EJ, L.; Bignamini, A.; Mannelli, P. The efficacy and safety of acamproste on the maintenance of abstinence in weaned alcoholics [Conference]. *NIDA research monograph* 1996;162:329.
94. Ulrichsen JN, M. K.; Ulrichsen, M. Disulfiram in severe alcoholism--an open controlled study. *Journal Article; Randomized Controlled Trial; Research Support, Non-US Gov't* 2010;64(6):356-62.
95. Volpicelli JRR, K. C.; Rhines, J. S.; Volpicelli, L. A.; Alterman, A. I.; O'Brien, C. P. Naltrexone and alcohol dependence. Role of subject compliance. *Clinical Trial; Comparative Study; Journal Article; Randomized Controlled Trial; Research Support, Non-US Gov't* 1997;54(8):737-42.
96. Wetzel HS, A.; Scheurich, A.; Lo?=rch, B.; Singer, P.; Schlo?=fke, D.; Sittinger, H.; Wobrock, T.; Mo?=ller, M. J.; Anghelescu, I.; Hautzinger, M. Combination treatment with nefazodone and cognitive-behavioral therapy for relapse prevention in alcohol-dependent men: a randomized controlled study. *Clinical Trial; Journal Article; Randomized Controlled Trial; Research Support, Non-US Gov't* 2004;65(10):1406-13.
97. Whitworth ABF, F.; Lesch, O. M.; Nimmerrichter, A.; Oberbauer, H.; Platz, T.; Potgieter, A.; Walter, H.; Fleischhacker, W. W. Comparison of acamprosate and placebo in long-term treatment of alcohol dependence. *Clinical Trial; Comparative Study; Journal Article; Multicenter Study; Randomized Controlled Trial; Research Support, Non-US Gov't* 1996;347(9013):1438-42.
98. Wiesbeck GAW, H. G.; Lesch, O. M.; Glaser, T.; Toennes, P. J.; Boening, J. Flupenthixol decanoate and relapse prevention in alcoholics: results from a placebo-controlled study. *Clinical Trial; Comparative Study; Journal Article; Randomized Controlled Trial* 2001;36(4):329-34.
99. Wiesbeck GAW, H. G.; Wodarz, N.; Lesch, O. M.; Glaser, T.; Boening, J. Gender-related differences in pharmacological relapse prevention with flupenthixol decanoate in detoxified alcoholics. *Clinical Trial; Comparative Study; Journal Article; Multicenter Study; Randomized Controlled Trial; Research Support, Non-US Gov't* 2003;6(4):259-62.

SUPPLEMENT 6. RESULTS OF RISK OF BIAS ASSESSMENT

Trial	Outcome	R	De	Mi	Me	S	O
Angelone 1998	Abstinence (16 weeks)	!	!	+	+	+	!
BACLAD study	Abstinence (24 weeks)	+	+	+	+	+	+
Baltieri 2004	Abstinence (24 weeks)	!	+	+	+	+	!
Baltieri 2008	Abstinence (12 weeks)	+	+	–	+	+	–
Barrias 1997	Abstinence (360 days)	!	+	+	+	+	!
Bender 2007	Abstinence (24 weeks)	+	+	+	+	+	+
Besson 1998	Abstinence (360 days)	!	+	+	+	+	!
Burtscheidt 2002	Abstinence (12 months)	!	!	+	!	+	!
Caputo 2003	Abstinence (3 months)	!	+	+	+	+	!
Caputo 2007	Abstinence (3 months)	!	!	+	+	+	!
Chick 2000	Abstinence (6 months)	+	+	+	+	+	+
Chick 2004	Abstinence (52 weeks)	+	+	–	+	+	–
Cornelius 1997	Abstinence (12 weeks)	!	+	+	+	+	!
Coriale 2019	Abstinence (365 days)	!	+	–	!	!	–
Croissant 2006	Abstinence (24 weeks)	!	!	!	+	+	!
Favre 1997	Abstinence (9 months)	!	+	!	+	+	!
Florez 2008	Abstinence (6 months)	!	–	+	+	+	–
Florez 2010	Abstinence (6 months)	!	!	+	–	+	–
Friedmann 2008	Abstinence (6 months)	!	+	+	+	+	!
Fuente 1989	Abstinence (6 months)	!	+	+	+	+	!
Fuller 1986	Abstinence (12 months)	+	+	!	+	+	!
GATE 2 study	Abstinence (12 months)	!	+	–	+	!	–
Geerlings 1997	Abstinence (1 year)	+	+	!	+	+	!
Gottlieb 1994	Abstinence (1 year)	!	+	–	+	+	–
Gual 2001	Abstinence (180 days)	!	+	+	+	+	!
Gual 2002	Abstinence (180 days)	!	+	+	+	+	!
Gustafson 2014	Abstinence (12 months)	+	!	+	–	+	–
Huang 2002	Abstinence (3 months)	!	+	+	+	+	!
Huang 2005	Abstinence (14 weeks)	!	+	+	+	+	!
Janiri 1997	Abstinence (90 days)	!	!	!	–	!	–
Jirapramukpitak 2020	Abstinence (12 weeks)	!	+	+	+	+	!
Joos 2013	Abstinence (8.5 months)	+	+	–	+	!	–

Trial	Outcome	R	De	Mi	Me	S	O
Kampman 2007	Abstinence (12 weeks)	!	+	+	+	+	!
Kiefer 2003	Abstinence (12 weeks)	+	+	+	+	+	+
Ladewig 1993	Abstinence (12 months)	!	+	+	+	+	!
Landabaso 1999	Abstinence (12 months)	!	!	+	–	!	–
Mann 2006	Abstinence (24 weeks)	!	+	!	+	+	!
Marra 2002	Abstinence (12 months)	!	+	–	+	+	–
Martinotti 2007	Abstinence (90 days)	+	!	–	+	+	–
Martinotti 2009	Abstinence (16 weeks)	+	+	!	+	+	!
Martinotti 2010	Abstinence (16 weeks)	+	+	!	+	+	!
MATCH project	Abstinence (9 months)	+	!	+	+	+	!
Moncini 2000	Abstinence (6 months)	+	+	!	+	+	!
Moraes 2010	Abstinence (12 weeks)	+	!	–	–	+	–
Mueller 1997	Abstinence (12 months)	!	+	–	+	+	–
Oslin 2005	Abstinence (3 months)	!	+	+	–	+	–
Paille 1995	Abstinence (360 days)	!	+	–	+	+	–
Pelc 1992	Abstinence (180 days)	!	+	–	+	!	–
Pelc 1997	Abstinence (90 days)	!	+	–	+	+	–
Pelc 2005	Abstinence (26 weeks)	+	–	–	–	+	–
Poldrugo 1997	Abstinence (12 months)	+	+	–	+	+	–
Ponce 2005	Abstinence (12 weeks)	!	!	–	+	+	–
PREDICT study	Abstinence (90 days)	+	+	+	+	+	+
Richter 2012	Abstinence (16 weeks)	+	+	–	+	+	–
Rubio 2005	Abstinence (12 weeks)	!	!	!	+	+	!
Sass 1996	Abstinence (48 weeks)	+	+	–	+	+	–
Schmidt 2002	Abstinence (12 months)	!	+	–	+	+	–
Stella 2008	Abstinence (6 months)	!	!	+	–	+	–
Tempesta 2000	Abstinence (270 days)	+	+	+	+	+	+
Ulrichsen 2010	Abstinence (6 months)	+	!	!	–	+	–
Volpicelli 1997	Abstinence (12 weeks)	!	+	!	+	+	!
Wetzel 2004	Abstinence (52 weeks)	+	+	!	+	+	!
Whitworth 1996	Abstinence (360 days)	+	+	+	+	+	+
Wiesbeck 2001	Abstinence (12 months)	!	+	!	+	+	!

R Bias arising from the randomization process
De Bias due to deviations from intended interventions
Mi Bias due to missing outcome data
Me Bias in measurement of the outcome
S Bias in selection of the reported result
O Overall risk of bias

+ Low risk
! Some concerns
– High risk

**Reference**	MATCH project	**Aim**	assignment to intervention (the 'intention-to-treat' effect)	**Source**	Journal article(s) with results of the trial; Trial protocol; Grant database summary (e.g. NIH RePORTER, Research Councils UK Gateway to Research)
**Outcome**	Abstinence (9 months)	**Results**	79/266 (CBT) vs 69/261 (MET) vs 72/247 (TSF)		

Domain	Signalling question	Response	Comments
**Bias arising from the randomization process**	1.1 Was the allocation sequence random?	Y	"The randomization process is centrally controlled by the (Yale) CC."
	1.2 Was the allocation sequence concealed until participants were recruited and assigned to interventions?	Y	"To ensure consistent delivery of treatments across sites, training, supervision, and certification of therapists are centralized at the Yale CC."
	1.3 Were there baseline imbalances that suggest a problem with the randomization process?	PN	"This procedure was successful, in that there were no significant differences across treatments on the matching variables assessed at baseline"
	**Risk of bias judgement**	**Low**	This study employed adequate randomisation methods in the trial and represents low risk of bias in the randomisation process.
**Bias due to deviations from intended interventions**	2.1 Were participants aware of their assigned intervention during the trial?	Y	Interventions were different and MET had only 4 sessions so it was impossible to blind participants.
	2.2 Were carers and trial personnel aware of participants' assigned intervention during the trial?	PY	
	2.3 If Y/PY/NI to 2.1 or 2.2: Were there deviations from the intended intervention beyond what would be expected in usual practice?	NI	
	2.4 If Y/PY to 2.3: Were these deviations from intended intervention unbalanced between groups and likely to have affected the outcome?	NA	
	2.5 If N/PN/NI to 2.4: Were these deviations likely to have affected the outcome?	N	
	2.6 If Y/PY/NI to 2.5: Was there potential for a substantial impact (on the estimated effect of intervention) of analysing participants in the wrong group?	NA	
	**Risk of bias judgement**	**Some concerns**	It was impossible to blind participants in this trial due to nature of interventions employed, which potentially induce deviations from intended interventions. On the other hand, the authors applied ITT analyses. Together, these contributed to "some concerns" in this domain.
**Bias due to missing outcome data**	3.1 Were outcome data available for all, or nearly all, participants randomized?	Y	Whole data were supplied by the committee.
	3.2 If N/PN/NI to 3.1: Are the proportions of missing outcome data and reasons for missing outcome data similar across intervention groups?	NA	
	3.3 If N/PN/NI to 3.1: Is there evidence that results were robust to the presence of missing outcome data?	NA	
	**Risk of bias judgement**	**Low**	The MATCH project commeittee provided the data and nearly all participants were followed during 9 months period in the main analysis.
**Bias in measurement of the outcome**	4.1 Were outcome assessors aware of the intervention received by study participants?	PY	It was impossible to blind outcome assessors.
	4.2 If Y/PY/NI to 4.1: Was the assessment of the outcome likely to be influenced by knowledge of intervention received?	PN	"Laboratory tests are used to screen subjects for exclusion criteria (e.g., unreported drug use), monitor changes in alcohol consumption..."
	**Risk of bias judgement**	**Low**	Although it was impossible to blind outcome assesser(patients), the outcome (abstinence) was confirmed by laboratory tests, which put low risk of bias in this domain.
**Bias in selection of the reported result**	Are the reported outcome data likely to have been selected, on the basis of the results, from…		
	5.1. ... multiple outcome measurements (e.g. scales, definitions, time points) within the outcome domain?	N	All time points were reported
	5.2 ... multiple analyses of the data?	N	
	**Risk of bias judgement**	**Low**	The reviewer re-analysed total abstinence, aligning to the common definition. This was not included in the protocol so we rated "Low" in selection of the reported results.
**Overall bias**	**Risk of bias judgement**	**Some concerns**	Some concerns in deviations from the intended interventions contributed to "some concerns" in overall bias.

**Reference**	Angelone 1998	**Aim**	assignment to intervention (the 'intention-to-treat' effect)	**Source**	Journal article(s) with results of the trial
**Outcome**	Abstinence (16 weeks)	**Results**	17/33 (Citalopram 20 mg) vs 14/25 (Fluvoixamine 150 mg) vs 7/23 (No pharmacological treatment)		

Domain	Signalling question	Response	Comments
**Bias arising from the randomization process**	1.1 Was the allocation sequence random?	NI	Only stated "random"
	1.2 Was the allocation sequence concealed until participants were recruited and assigned to interventions?	NI	
	1.3 Were there baseline imbalances that suggest a problem with the randomization process?	PN	A significant difference among groups, 23 vs 25 vs 33 but it was done deliberately, "The citalopram group was deliberately made larger a priori to achieve more experience with this drug, which is relatively new in Italy". No significant difference among characteristics of the study sample besides age and M:F ratio in the citalopram group.
	**Risk of bias judgement**	**Some concerns**	No details were given regarding randomisation process. Although there were some difference between aga and M:F ratio among trials, it might due to small number of participants in this trial as by chance. Together these contributed to "some concerns" in this domain.
**Bias due to deviations from intended interventions**	2.1 Were participants aware of their assigned intervention during the trial?	NI	Did not address blinding procedures except psychiatrists.
	2.2 Were carers and trial personnel aware of participants' assigned intervention during the trial?	PN	"Psychiatric assessment was made by a trained psychiatrist (blind to the medication) every 2 weeks starting from the fourth week."
	2.3 If Y/PY/NI to 2.1 or 2.2: Were there deviations from the intended intervention beyond what would be expected in usual practice?		One of the groups did not receive interventions, which might lead to deviations.
	2.4 If Y/PY to 2.3: Were these deviations from intended intervention unbalanced between groups and likely to have affected the outcome?	NA	
	2.5 If N/PN/NI to 2.4: Were these deviations likely to have affected the outcome?	N	
	2.6 If Y/PY/NI to 2.5: Was there potential for a substantial impact (on the estimated effect of intervention) of analysing participants in the wrong group?	NA	
	**Risk of bias judgement**	**Some concerns**	There was no complete information regarding blinding and one group did not receive any interventions (treated as usual), which might l to deviation. On the other hand, the authors applied ITT analyses. Together, these contributed to "some concerns" in this domain.
**Bias due to missing outcome data**	3.1 Were outcome data available for all, or nearly all, participants randomized?	N	Some drop-outs were seen owing to side effects or moved were not included n=3 of 25 in fluvoxamine group; n=5 of 33 in citalopram group.
	3.2 If N/PN/NI to 3.1: Are the proportions of missing outcome data and reasons for missing outcome data similar across intervention groups?	PN	0%; 12%; 15% respectively - not clear if reasons for missing outcome data are similar or not.
	3.3 If N/PN/NI to 3.1: Is there evidence that results were robust to the presence of missing outcome data?	PY	The authors did not perform sensitivity analyses but both on-treatment and ITT analyses led same results.
	**Risk of bias judgement**	**Low**	Although there were some missing data, results still stood in consideration of missing data. "Low" risk of bias in this domain was rated.
**Bias in measurement of the outcome**	4.1 Were outcome assessors aware of the intervention received by study participants?	PN	Did not address blinding of participants in the trial and the outcome was self-assessed and confirmed by relatives.<br>"The presence of relatives, or other key individuals for the patient, was required at each assessment, to confirm the patient's report and to obtain additional information about their alcohol intake."
	4.2 If Y/PY/NI to 4.1: Was the assessment of the outcome likely to be influenced by knowledge of intervention received?	NA	
	**Risk of bias judgement**	**Low**	Abstinence was confirmed by relatives or key individuals, which strengthend the reliability of results.

Domain	Signalling question	Response	Comments
Bias in selection of the reported result	Are the reported outcome data likely to have been selected, on the basis of the results, from…		
	5.1. ... multiple outcome measurements (e.g. scales, definitions, time points) within the outcome domain?	PN	
	5.2 ... multiple analyses of the data?	PN	
	**Risk of bias judgement**	**Low**	No protocol or statisitical analysis was found but nature of outcome, abstinence, presents low risk of selected reported results and the authors described the method section clearly. Thus, we rated "Low" risk of bias in this domain.
**Overall bias**	**Risk of bias judgement**	**Some concerns**	Lack of detailed randomisation process and potential deviations from the intended interventions due to difference among interventions, together, these contributed to "some concerns" in overall bias for this trial.

**Reference**	Baltieri 2004	**Aim**	assignment to intervention (the 'intention-to-treat' effect)	**Source**	Journal article(s) with results of the trial
**Outcome**	Abstinence (24 weeks)	**Results**	17/40 (Acamprosate 1998 mg) vs 7/35 (Placebo)		

Domain	Signalling question	Response	Comments
**Bias arising from the randomization process**	1.1 Was the allocation sequence random?	NI	Only stated "random"
	1.2 Was the allocation sequence concealed until participants were recruited and assigned to interventions?	NI	
	1.3 Were there baseline imbalances that suggest a problem with the randomization process?	PN	There were 35 and 40 patients in placebo and acamprosate groups; The average daily alcohol intake was slight higher in acamprosate group than placebo group (370.1 (164.91) vs 348.5 (132.46)) but not statistically significant.
	**Risk of bias judgement**	**Some concerns**	No details were given regarding randomisation process and no significant difference between groups suggesting "Some concerns" in this domain.
**Bias due to deviations from intended interventions**	2.1 Were participants aware of their assigned intervention during the trial?	PN	"double-blind"
	2.2 Were carers and trial personnel aware of participants' assigned intervention during the trial?	PN	
	2.3 If Y/PY/NI to 2.1 or 2.2: Were there deviations from the intended intervention beyond what would be expected in usual practice?	NA	
	2.4 If Y/PY to 2.3: Were these deviations from intended intervention unbalanced between groups and likely to have affected the outcome?	NA	
	2.5 If N/PN/NI to 2.4: Were these deviations likely to have affected the outcome?	N	
	2.6 If Y/PY/NI to 2.5: Was there potential for a substantial impact (on the estimated effect of intervention) of analysing participants in the wrong group?	NA	
	**Risk of bias judgement**	**Low**	Double-blind design and ITT analysis employed in this trial put low risk of bias in deviation from intended interventions.
**Bias due to missing outcome data**	3.1 Were outcome data available for all, or nearly all, participants randomized?	N	"Only 58 (77%) of patients remained for the length of the study."
	3.2 If N/PN/NI to 3.1: Are the proportions of missing outcome data and reasons for missing outcome data similar across intervention groups?	Y	"Ten patients who were receiving acamprosate and seven patients who were receiving placebo dropped out." "The reasons for dropping out were unwillingness to continue the treatment (two patients of the acamprosate group and two of the placebo group); "protocol violation," which was defined as the use of other psychopharmacological drugs during the study (one patient of the acamprosate group and one of the placebo group); and unavailability for follow-up (seven patients of the acamprosate group and four of the placebo group)."
	3.3 If N/PN/NI to 3.1: Is there evidence that results were robust to the presence of missing outcome data?	PN	Conservative "Patients who missed a visit or withdrew from the study were deemed to be nonabstinent at the time those data were not available" and no sensitivity analysis.
	**Risk of bias judgement**	**Low**	Although there were some missing data, results still stood in consideration of balanced missing data. "Low" risk of bias in this domain was rated.
**Bias in measurement of the outcome**	4.1 Were outcome assessors aware of the intervention received by study participants?	PN	"double-blind" "Major variables recorded at each visit included clinical examination results, patients' self-reported quantity and frequency of alcohol consumption and drug side effects. The patients' declaration of drinking behavior was verified by the results of γ-glutamyltransferase (GGT) levels in every case and by interviewing a family member if possible."
	4.2 If Y/PY/NI to 4.1: Was the assessment of the outcome likely to be influenced by knowledge of intervention received?	NA	
	**Risk of bias judgement**	**Low**	Double-blind design and self-reporting outcome (confirmed by relatives and biochemistry results) put this domain as low risk of bias.
**Bias in selection of the reported result**	Are the reported outcome data likely to have been selected, on the basis of the results, from…		
	5.1. ... multiple outcome measurements (e.g. scales, definitions, time points) within the outcome domain?	PN	"The patients' declaration of drinking behavior was verified by the results of γ-glutamyltransferase (GGT) levels in every case and by interviewing a family member if possible."
	5.2 ... multiple analyses of the data?	PN	
	**Risk of bias judgement**	**Low**	No protocol or statisitical analysis was found but nature of outcome, abstinence, presents low risk of selected reported results and the authors described the method section clearly. Thus, we rated "Low" risk of bias in this domain.
**Overall bias**	**Risk of bias judgement**	**Some concerns**	Lack of detailed randomisation process contributed to "some concerns" in overall bias for this trial.

**Reference**	Baltieri 2008	**Aim**	assignment to intervention (the 'intention-to-treat' effect)	**Source**	Journal article(s) with results of the trial
**Outcome**	Abstinence (12 weeks)	**Results**	14/49 (Naltrexone) vs 15/54 (Placebo) vs 24/52 (Topiramate)		

Domain	Signalling question	Response	Comments
**Bias arising from the randomization process**	1.1 Was the allocation sequence random?	Y	"patients were assigned randomly to one of the three medication conditions through a random number list" "Medication was dispensed under double-blind conditions. Only two pharmacists from the pharmacy sector at the Psychiatric Institute of the Clinical Hospital of the University of São Paulo knew which medication corresponded to the specific code. The packages containing the capsules were distributed to patients by two blinded research assistants, who also assessed patient outcomes throughout the study."
	1.2 Was the allocation sequence concealed until participants were recruited and assigned to interventions?	PY	
	1.3 Were there baseline imbalances that suggest a problem with the randomization process?	PN	"As shown in Table 2, therewere no significant differences among the groups at baseline on any socio-demographic, drug use, hepatic function or psychometric variables measured."
	**Risk of bias judgement**	**Low**	This study employed adequate randomisation methods in the trial and represents low risk of bias in the randomisation process.
**Bias due to deviations from intended interventions**	2.1 Were participants aware of their assigned intervention during the trial?	PN	"double-blind"; "Validity of the double-blind procedure was verified by obtaining a prediction from each patient and staff member as to whether a given individual had received active or placebo medication during the study." "Overall, researchers were able to differentiate active treatment (naltrexone or topiramate) correctly from placebo treatment in 33.6% of cases. Among subjects, 27% were able to differentiate active treatment (naltrexone or topiramate) correctly from placebo treatment."
	2.2 Were carers and trial personnel aware of participants' assigned intervention during the trial?	PN	
	2.3 If Y/PY/NI to 2.1 or 2.2: Were there deviations from the intended intervention beyond what would be expected in usual practice?	NA	
	2.4 If Y/PY to 2.3: Were these deviations from intended intervention unbalanced between groups and likely to have affected the outcome?	NA	
	2.5 If N/PN/NI to 2.4: Were these deviations likely to have affected the outcome?	N	

	2.6 If Y/PY/NI to 2.5: Was there potential for a substantial impact (on the estimated effect of intervention) of analysing participants in the wrong group?	NA	
	**Risk of bias judgement**	**Low**	Double-blind design and ITT analysis employed in this trial put low risk of bias in deviation from intended interventions.
**Bias due to missing outcome data**	3.1 Were outcome data available for all, or nearly all, participants randomized?	N	70/155 patients dropped out.
	3.2 If N/PN/NI to 3.1: Are the proportions of missing outcome data and reasons for missing outcome data similar across intervention groups?	N	More drop-outs (57.4%) in placebo group than other groups (40.8% and 36.4%).<br>"Differences between conditions in overall dropout rates approached significance (X2 = 5.10, P < 0.07) and were statistically significant within the lost-to-follow-up category (X = 7.723, P < 0.02) with a significant difference between topiramate and placebo in post-hoc analysis.
	3.3 If N/PN/NI to 3.1: Is there evidence that results were robust to the presence of missing outcome data?	PN	Conservative "Patients who missed a visit or withdrew from the study were deemed to be non-abstinent at the time of missed visits." and no sensitivity analysis
	**Risk of bias judgement**	**High**	High porportion of missing data and imbalanced missing data without sensitivity analysis put "high" risk of bias in this domain.
**Bias in measurement of the outcome**	4.1 Were outcome assessors aware of the intervention received by study participants?	PN	"two blinded research assistants, who also assessed patient outcomes throughout the study."
	4.2 If Y/PY/NI to 4.1: Was the assessment of the outcome likely to be influenced by knowledge of intervention received?	NA	
	**Risk of bias judgement**	**Low**	Double-blind design in the outcome assessors put low risk of bias in this trial.
**Bias in selection of the reported result**	Are the reported outcome data likely to have been selected, on the basis of the results, from…		
	5.1. ... multiple outcome measurements (e.g. scales, definitions, time points) within the outcome domain?	PN	"Alcohol consumption during the treatment was determined using a dailymonitoring card and compliance was evaluated by self-report, capsules count of the returned medication package and the dailymonitoring card."
	5.2 ... multiple analyses of the data?	PN	
	**Risk of bias judgement**	**Low**	No protocol or statisitical analysis was found but nature of outcome, abstinence, presents low risk of selected reported results and the authors described the method section clearly. Thus, we rated "Low" risk of bias in this domain.
**Overall bias**	**Risk of bias judgement**	**High**	High porportion of missing data and imbalanced missing data without sensitivity analysis put "high" risk of bias.

**Reference**	Bender 2007	**Aim**	assignment to intervention (the 'intention-to-treat' effect)	**Source**	Journal article(s) with results of the trial
**Outcome**	Abstinence (24 weeks)	**Results**	54/150 (Placebo) vs 37/149 (Tiapride 300 mg)		

Domain	Signalling question	Response	Comments
**Bias arising from the randomization process**	1.1 Was the allocation sequence random?	Y	"...patients were randomly assigned to one of the two treatment groups according to a predefined random code."<br>"Eligible patients were chronologically randomized by assigning them the lowest unassigned treatment number available at the study centre."
	1.2 Was the allocation sequence concealed until participants were recruited and assigned to interventions?	PY	"...Study medication was administered in tablets indistinguishable in colour, size, form, smell, taste, consistency, and packaging"
	1.3 Were there baseline imbalances that suggest a problem with the randomization process?	N	"Tiapride (n = 149) vs Placebo (n = 150)
	**Risk of bias judgement**	**Low**	This study employed adequate randomisation methods in the trial and represents low risk of bias in the randomisation process.
**Bias due to deviations from intended interventions**	2.1 Were participants aware of their assigned intervention during the trial?	N	"This multi-centre, randomized, double-blind, placebocontrolled, parallel-group study was conducted at 11 centres in Germany (six psychiatric university hospitals, three non-academic psychiatric hospitals, one day-clinic and one private practice)."
	2.2 Were carers and trial personnel aware of participants' assigned intervention during the trial?	PN	"Study medication was administered in tablets indistinguishable in colour, size, form, smell, taste, consistency, and packaging."
	2.3 If Y/PY/NI to 2.1 or 2.2: Were there deviations from the intended intervention beyond what would be expected in usual practice?	NA	
	2.4 If Y/PY to 2.3: Were these deviations from intended intervention unbalanced between groups and likely to have affected the outcome?	NA	
	2.5 If N/PN/NI to 2.4: Were these deviations likely to have affected the outcome?	N	
	2.6 If Y/PY/NI to 2.5: Was there potential for a substantial impact (on the estimated effect of intervention) of analysing participants in the wrong group?	NA	
	**Risk of bias judgement**	**Low**	Double-blind design and ITT analysis employed in this trial put low risk of bias in deviation from intended interventions.
**Bias due to missing outcome data**	3.1 Were outcome data available for all, or nearly all, participants randomized?	N	"Of the 299 patients participating in the study, 31 patients (21%) in the tiapride group and 35 patients (23%) in the placebo group discontinued the treatment prematurely."
	3.2 If N/PN/NI to 3.1: Are the proportions of missing outcome data and reasons for missing outcome data similar across intervention groups?	PY	They did not give reasons for mssing outcome data but the reasons should be expected to be similar across intervention groups.<br>"The number of dropouts due to adverse events or intercurrent illnesses was comparable in both groups (tiapride, n=10; placebo, n=9)."
	3.3 If N/PN/NI to 3.1: Is there evidence that results were robust to the presence of missing outcome data?	PY	"In the worst-case analysis, all dropouts for unknown reasons (lost to follow-up: tiapride, n=12; placebo, n=24) were considered as relapse. In this analysis, the relapse rate was 62% in the tiapride group (93 patients) and 57% in the placebo group (85 patients)."<br>"The difference in relapse rates in the worst-case analysis was not statistically significant (x2 test, p=0.31)."
	**Risk of bias judgement**	**Low**	Although there were some missing data, results still stood in consideration of balanced missing data after sensitivity analysis. "Low" risk of bias in this domain was rated.
**Bias in measurement of the outcome**	4.1 Were outcome assessors aware of the intervention received by study participants?	PN	"...double-blind study" might suggest that outcome assessors were not aware of the intervention.
	4.2 If Y/PY/NI to 4.1: Was the assessment of the outcome likely to be influenced by knowledge of intervention received?	NA	
	**Risk of bias judgement**	**Low**	Double-blind design that outcome assessers (patients) were not influenced by the knowledge of intervention received puts this domain as low risk of bias.
**Bias in selection of the reported result**	Are the reported outcome data likely to have been selected, on the basis of the results, from…		
	5.1. ... multiple outcome measurements (e.g. scales, definitions, time points) within the outcome domain?	PN	
	5.2 ... multiple analyses of the data?	PN	
	**Risk of bias judgement**	**Low**	No protocol or statisitical analysis was found but nature of outcome, abstinence, presents low risk of selected reported results and the authors described the method section clearly. Thus, we rated "Low" risk of bias in this domain.
**Overall bias**	**Risk of bias judgement**	**Low**	Overall low risk of bias

**Reference**	Besson 1998	**Aim**	assignment to intervention (the 'intention-to-treat' effect)	**Source**	Journal article(s) with results of the trial

Outcome	Abstinence (360 days)	Results	14/55 (Acamprosate 1998 mg) vs 3/55 (Placebo)

Domain	Signalling question	Response	Comments
Bias arising from the randomization process	1.1 Was the allocation sequence random?	PY	"randomized, parallel, double-blind, placebo-controlled study" "Balanced randomization"
	1.2 Was the allocation sequence concealed until participants were recruited and assigned to interventions?	NI	
	1.3 Were there baseline imbalances that suggest a problem with the randomization process?	N	No significant difference between groups was found.
	**Risk of bias judgement**	**Some concerns**	No details were given regarding randomisation process, contributed to "some concerns" in this domain.
Bias due to deviations from intended interventions	2.1 Were participants aware of their assigned intervention during the trial?	PN	"double-blind"
	2.2 Were carers and trial personnel aware of participants' assigned intervention during the trial?	PN	
	2.3 If Y/PY/NI to 2.1 or 2.2: Were there deviations from the intended intervention beyond what would be expected in usual practice?	NA	
	2.4 If Y/PY to 2.3: Were these deviations from intended intervention unbalanced between groups and likely to have affected the outcome?	NA	
	2.5 If N/PN/NI to 2.4: Were these deviations likely to have affected the outcome?	N	
	2.6 If Y/PY/NI to 2.5: Was there potential for a substantial impact (on the estimated effect of intervention) of analysing participants in the wrong group?	NA	
	**Risk of bias judgement**	**Low**	Double-blind design and ITT analysis employed in this trial put "Low" risk of bias in deviation from intended interventions.
Bias due to missing outcome data	3.1 Were outcome data available for all, or nearly all, participants randomized?	PN	36/55 participants in each group were nonattendant at 1y follow-up.<br>In the analysis, non-attenders were assumed to have relapsed.
	3.2 If N/PN/NI to 3.1: Are the proportions of missing outcome data and reasons for missing outcome data similar across intervention groups?	PY	Proportions of missing data identical in the two groups. Reasons for missing data approximately balanced between groups, although slightly more patients in the placebo group were nonattendant due to relapse requiring rehospitalization, and slightly more patients were nonattendent due to concurrent illness in the acamprosate group.
	3.3 If N/PN/NI to 3.1: Is there evidence that results were robust to the presence of missing outcome data?	PN	
	**Risk of bias judgement**	**Low**	Although there were some missing data, results still stood in consideration of missing data. "Low" risk of bias in this domain was rated.
Bias in measurement of the outcome	4.1 Were outcome assessors aware of the intervention received by study participants?	PN	"double-blind"
	4.2 If Y/PY/NI to 4.1: Was the assessment of the outcome likely to be influenced by knowledge of intervention received?	NA	
	**Risk of bias judgement**	**Low**	Double-blind design and self-reporting put this domain as low risk of bias.
Bias in selection of the reported result	Are the reported outcome data likely to have been selected, on the basis of the results, from…		
	5.1. ... multiple outcome measurements (e.g. scales, definitions, time points) within the outcome domain?	PN	
	5.2 ... multiple analyses of the data?	PN	
	**Risk of bias judgement**	**Low**	No protocol was found but nature of outcome, abstinence, presents low risk of selected reported results and the authors described the method section clearly. Thus, we rated "Low" risk of bias in this domain.
**Overall bias**	**Risk of bias judgement**	**Some concerns**	Lack of detailed methods for the randomisation process contributed to "some concerns" in overall bias for this trial.

Reference	Burtscheidt 2002	Aim	assignment to intervention (the 'intention-to-treat' effect)	Source	Journal article(s) with results of the trial
Outcome	Abstinence (12 months)	Results	11/40 (CBT) vs 8/40 (CST) vs 11/40 (Standard therapy)		

Domain	Signalling question	Response	Comments
Bias arising from the randomization process	1.1 Was the allocation sequence random?	NI	"Preferences of the patients for any of the three treatment approaches did not emerge; there were no significant differences in age, sex, severity and duration of illness, and sociodemographic data in terms of education, employment, and familial status between the three therapy groups."
	1.2 Was the allocation sequence concealed until participants were recruited and assigned to interventions?	NI	
	1.3 Were there baseline imbalances that suggest a problem with the randomization process?	PN	there were no significant differences in age, sex, severity and duration of illness, and sociodemographic data in terms of education, employment, and familial status between the three therapy groups.
	**Risk of bias judgement**	**Some concerns**	No details were given regarding randomisation process, contributed to "some concerns" in this domain.
Bias due to deviations from intended interventions	2.1 Were participants aware of their assigned intervention during the trial?	PY	No blinding was used in this trial and interventions varied across groups.
	2.2 Were carers and trial personnel aware of participants' assigned intervention during the trial?	PY	
	2.3 If Y/PY/NI to 2.1 or 2.2: Were there deviations from the intended intervention beyond what would be expected in usual practice?	NI	No details on deviations from expected practice
	2.4 If Y/PY to 2.3: Were these deviations from intended intervention unbalanced between groups and likely to have affected the outcome?	NA	
	2.5 If N/PN/NI to 2.4: Were these deviations likely to have affected the outcome?	N	No evidence that patients recieved a treatment other than the one they were assigned to.
	2.6 If Y/PY/NI to 2.5: Was there potential for a substantial impact (on the estimated effect of intervention) of analysing participants in the wrong group?	NA	
	**Risk of bias judgement**	**Some concerns**	The open label design in this trial might prompt deviations from the intended interventions, contributing to "some concerns" in this domain.
Bias due to missing outcome data	3.1 Were outcome data available for all, or nearly all, participants randomized?	PN	There were some missing data due to loss to follow-up (~15%).
	3.2 If N/PN/NI to 3.1: Are the proportions of missing outcome data and reasons for missing outcome data similar across intervention groups?	PY	"There were no significant differences between the three treatment groups in this respect." = missing data rate.<br><br>No information provided on whether reasons for missing data were comparable between intervention groups
	3.3 If N/PN/NI to 3.1: Is there evidence that results were robust to the presence of missing outcome data?	NI	Unclear how missing data were handled. No sensitivity analysis
	**Risk of bias judgement**	**Low**	Although there were some missing data, results still stood in consideration of balanced missing data. "Low" risk of bias in this domain was rated.
Bias in measurement of the outcome	4.1 Were outcome assessors aware of the intervention received by study participants?	PY	Self report and reports from family/friends, who are aware of the treatment received.
	4.2 If Y/PY/NI to 4.1: Was the assessment of the outcome likely to be influenced by knowledge of intervention received?	PY	Outcomes are subjective and could have been influenced by awareness of the treatment
	**Risk of bias judgement**	**Some concerns**	Although this is an open study, the outcome (abstinence) was confirmed by family member and saliva test, which put "some concerns" risk of bias in this domain.
Bias in selection of the reported	Are the reported outcome data likely to have been selected, on the basis of the results, from…		
	5.1. ... multiple outcome measurements (e.g. scales, definitions, time points) within the outcome domain?	PN	

of the reported result	5.2 ... multiple analyses of the data?	PN	
	**Risk of bias judgement**	**Low**	No protocol was found but nature of outcome, abstinence, presents low risk of selected reported results and the authors described the method section clearly. Thus, we rated "Low" risk of bias in this domain.
**Overall bias**	**Risk of bias judgement**	**Some concerns**	Lack of detailed methods for the randomisation process and blinding of participants and personnel, together, these contributed to "some concerns" in overall bias for this trial.

**Reference**	Caputo 2003	**Aim**	assignment to intervention (the 'intention-to-treat' effect)	**Source**	Journal article(s) with results of the trial
**Outcome**	Abstinence (3 months)	**Results**	12/18 (GHB 50 mg/kg) vs 6/17 (Naltrexone 50 mg)		

Domain	Signalling question	Response	Comments
**Bias arising from the randomization process**	1.1 Was the allocation sequence random?	NI	"...receive randomly during the treatment period, as well as on the possibility of dropping out of the study at any time"
	1.2 Was the allocation sequence concealed until participants were recruited and assigned to interventions?	NI	
	1.3 Were there baseline imbalances that suggest a problem with the randomization process?	N	"At the time of admission to the study (Table 1), the two groups did not differ in terms of demographic data, education, employment, marital status, duration of alcohol addiction, time of abstinence, alcohol craving scale and alcohol dependence degree."
	**Risk of bias judgement**	**Some concerns**	No details were given regarding randomisation process, contributed to "some concerns" in this domain.
**Bias due to deviations from intended interventions**	2.1 Were participants aware of their assigned intervention during the trial?	Y	"Patients were aware of the drug they would receive and were abstinent at the time of admission to the study."
	2.2 Were carers and trial personnel aware of participants' assigned intervention during the trial?	NI	
	2.3 If Y/PY/NI to 2.1 or 2.2: Were there deviations from the intended intervention beyond what would be expected in usual practice?	PN	As both medications were used for treating alcohol dependence.
	2.4 If Y/PY to 2.3: Were these deviations from intended intervention unbalanced between groups and likely to have affected the outcome?	NA	
	2.5 If N/PN/NI to 2.4: Were these deviations likely to have affected the outcome?	PN	
	2.6 If Y/PY/NI to 2.5: Was there potential for a substantial impact (on the estimated effect of intervention) of analysing participants in the wrong group?	NA	
	**Risk of bias judgement**	**Low**	Although this is an open trial, both interventions were used clinically for treating alcohol dependence. This expects minimal deviations from the intended intervention beyond usual practice. Therefore, we rate "Low" risk of bias in this domain.
**Bias due to missing outcome data**	3.1 Were outcome data available for all, or nearly all, participants randomized?	N	"Eight (22.9%; seven males) patients dropped out: four patients developed severe side-effects [one (2.8%) in the GHB group and three (8.5%) in the NTX group]..."
	3.2 If N/PN/NI to 3.1: Are the proportions of missing outcome data and reasons for missing outcome data similar across intervention groups?	PY	% comparable across arms, and reasons seem broadly similar.
	3.3 If N/PN/NI to 3.1: Is there evidence that results were robust to the presence of missing outcome data?	PY	No sensitivity analysis but results still stood by considering the missing data.
	**Risk of bias judgement**	**Low**	Although there were some missing data, missing data presented equally in both groups and results stood the same in consideration of missing data. "Low" risk of bias in this domain was rated.
**Bias in measurement of the outcome**	4.1 Were outcome assessors aware of the intervention received by study participants?	Y	Open study
	4.2 If Y/PY/NI to 4.1: Was the assessment of the outcome likely to be influenced by knowledge of intervention received?	PN	"...the interview of a family member and the determination of blood alcohol concentrations and alcohol in the saliva (Quantitative Ethanol Determination; Enzymatics Inc., Horsham, UK) at the end of every week of treatment..."
	**Risk of bias judgement**	**Low**	Although this is an open study, the outcome (abstinence) was confirmed by family member and saliva test, which put low risk of bias in this domain.
**Bias in selection of the reported result**	Are the reported outcome data likely to have been selected, on the basis of the results, from…		
	5.1. ... multiple outcome measurements (e.g. scales, definitions, time points) within the outcome domain?	PN	
	5.2 ... multiple analyses of the data?	PN	
	**Risk of bias judgement**	**Low**	No protocol or statisitical analysis was found but nature of outcome, abstinence, presents low risk of selected reported results and the authors described the method section clearly. Thus, we rated "Low" risk of bias in this domain.
**Overall bias**	**Risk of bias judgement**	**Some concerns**	Lack of detailed randomisation process contributed to "some concerns" in overall bias for this trial.

**Reference**	Caputo 2007	**Aim**	assignment to intervention (the 'intention-to-treat' effect)	**Source**	Journal article(s) with results of the trial
**Outcome**	Abstinence (3 months)	**Results**	6/17 (Naltrexone 50 mg) vs 13/18 (GHB 50 mg/kg + NTX 50 mg) vs 12/18 (GHB 50 mg/kg)		

Domain	Signalling question	Response	Comments
**Bias arising from the randomization process**	1.1 Was the allocation sequence random?	NI	"...patients were randomly allocated to three groups."
	1.2 Was the allocation sequence concealed until participants were recruited and assigned to interventions?	NI	
	1.3 Were there baseline imbalances that suggest a problem with the randomization process?	PN	20 vs 18 vs 17.
	**Risk of bias judgement**	**Some concerns**	No details were given regarding randomisation process, contributed to "some concerns" in this domain.
**Bias due to deviations from intended interventions**	2.1 Were participants aware of their assigned intervention during the trial?	Y	"an open trial"
	2.2 Were carers and trial personnel aware of participants' assigned intervention during the trial?	PY	"After providing their informed consent, being aware of the aim of the study, dosing rate and possible side-effects of the drugs they were going to receive, as well as the possibility of dropping out of the..."
	2.3 If Y/PY/NI to 2.1 or 2.2: Were there deviations from the intended intervention beyond what would be expected in usual practice?	NI	No details on deviations from expected practice
	2.4 If Y/PY to 2.3: Were these deviations from intended intervention unbalanced between groups and likely to have affected the outcome?	NA	
	2.5 If N/PN/NI to 2.4: Were these deviations likely to have affected the outcome?	PN	
	2.6 If Y/PY/NI to 2.5: Was there potential for a substantial impact (on the estimated effect of intervention) of analysing participants in the wrong group?	NA	
	**Risk of bias judgement**	**Some concerns**	The open label design in this trial might prompt deviations from the intended interventions, contributing to "some concerns" in this domain.
**Bias due to missing outcome data**	3.1 Were outcome data available for all, or nearly all, participants randomized?	PN	There were around 10-25% of missing data in each group. (2/20 vs 3/18 vs 4/17)
	3.2 If N/PN/NI to 3.1: Are the proportions of missing outcome data and reasons for missing outcome data similar across intervention groups?	PY	"The incidence of each side-effect did not significantly differ between groups. . . .In addition to the patients who abandoned the study because of the occurrence of side-effects . . . the distribution of drop-outs in the three groups did not differ significantly."
	3.3 If N/PN/NI to 3.1: Is there evidence that results were robust to the presence of missing outcome data?	PY	No mention of how missing data were handled but results stood in consideration of missing data.

	**Risk of bias judgement**	**Low**	Although there were some missing data, results still stood in consideration of missing data. "Low" risk of bias in this domain was rated.
**Bias in measurement of the outcome**	4.1 Were outcome assessors aware of the intervention received by study participants?	PY	"...open trial..."
	4.2 If Y/PY/NI to 4.1: Was the assessment of the outcome likely to be influenced by knowledge of intervention received?	PN	"These parameters were assessed on the basis of participant self-evaluation, the interview of a family member and the determination of alcohol concentrations in blood and saliva" - robust outcome
	**Risk of bias judgement**	**Low**	Although this is an open study, the outcome (abstinence) was confirmed by family member and blood/saliva test, which put low risk of bias in this domain.
**Bias in selection of the reported result**	Are the reported outcome data likely to have been selected, on the basis of the results, from…		
	5.1. ... multiple outcome measurements (e.g. scales, definitions, time points) within the outcome domain?	PN	
	5.2 ... multiple analyses of the data?	PN	
	**Risk of bias judgement**	**Low**	No protocol or statisitical analysis was found but nature of outcome, abstinence, presents low risk of selected reported results and the authors described the method section clearly. Thus, we rated "Low" risk of bias in this domain.
**Overall bias**	**Risk of bias judgement**	**Some concerns**	Lack of detailed randomisation process, deviations from the intended interventions, together, these contributed to "some concerns" in overall bias for this trial.

**Reference**	Chick 2004	**Aim**	assignment to intervention (the 'intention-to-treat' effect)	**Source**	Journal article(s) with results of the trial
**Outcome**	Abstinence (52 weeks)	**Results**	70/243 (Fluvoxamine 300mg) vs 72/249 (Placebo)		

**Domain**	**Signalling question**	**Response**	**Comments**
**Bias arising from the randomization process**	1.1 Was the allocation sequence random?	Y	"Randomisation after meeting inclusion and exclusion criteria was within centres, in blocks of eight, four patients per block to each treatment. At randomisation, patients were given the next sequential number at that centre, and received the trial supplies for that patient number."<br><br>"The randomisation code was provided by the department of statistics and data management at Solvay–Duphar B.V."
	1.2 Was the allocation sequence concealed until participants were recruited and assigned to interventions?	Y	
	1.3 Were there baseline imbalances that suggest a problem with the randomization process?	N	"At entry, there were no differences that reached statistical significance in the characteristics measured of patients allocated to the two treatment groups."
	**Risk of bias judgement**	**Low**	This study employed adequate randomisation methods and represented low risk of bias in the randomisation process.
**Bias due to deviations from intended interventions**	2.1 Were participants aware of their assigned intervention during the trial?	PN	"Fluvoxamine (50 mg) and placebo were supplied in indistinguishable yellow enteric-coated tablets, in numbered containers for dispensing, according to a randomisation schedule held centrally and by the clinic pharmacist"
	2.2 Were carers and trial personnel aware of participants' assigned intervention during the trial?	PN	
	2.3 If Y/PY/NI to 2.1 or 2.2: Were there deviations from the intended intervention beyond what would be expected in usual practice?	NA	
	2.4 If Y/PY to 2.3: Were these deviations from intended intervention unbalanced between groups and likely to have affected the outcome?	NA	
	2.5 If N/PN/NI to 2.4: Were these deviations likely to have affected the outcome?	N	
	2.6 If Y/PY/NI to 2.5: Was there potential for a substantial impact (on the estimated effect of intervention) of analysing participants in the wrong group?	NA	
	**Risk of bias judgement**	**Low**	Double-blind design and ITT analysis employed in this trial put low risk of bias in deviation from intended interventions.
**Bias due to missing outcome data**	3.1 Were outcome data available for all, or nearly all, participants randomized?	N	By week 52, only 75/243 of Flu group and 117/249 of Pla group remained.
	3.2 If N/PN/NI to 3.1: Are the proportions of missing outcome data and reasons for missing outcome data similar across intervention groups?	PN	More participants in fluvoxamine group withdrawn.<br><br>See Table 2 - propotions are similar but reasons different between treatement arms.
	3.3 If N/PN/NI to 3.1: Is there evidence that results were robust to the presence of missing outcome data?	PN	No sensitivity analysis but used a conservative approach - "...in which all drop-outs were to be regarded as treatment failures."
	**Risk of bias judgement**	**High**	High porportion of missing data and imbalanced missing data without sensitivity analysis put "high" risk of bias in this domain.
**Bias in measurement of the outcome**	4.1 Were outcome assessors aware of the intervention received by study participants?	PN	"Patients were assessed, usually by the same rater at each occasion, after detoxification on the day of randomisation, and after 2, 4, 6, 8, 12, 16, 24, 32, 40 and 52 weeks of treatment." Robust double blind protocol means assessor would not know which treatment was received."
	4.2 If Y/PY/NI to 4.1: Was the assessment of the outcome likely to be influenced by knowledge of intervention received?	NA	
	**Risk of bias judgement**	**Low**	Double-blind design put this domain as low risk of bias.
**Bias in selection of the reported result**	Are the reported outcome data likely to have been selected, on the basis of the results, from…		
	5.1. ... multiple outcome measurements (e.g. scales, definitions, time points) within the outcome domain?	PN	
	5.2 ... multiple analyses of the data?	PN	
	**Risk of bias judgement**	**Low**	No protocol or statisitical analysis was found but nature of outcome, abstinence, presents low risk of selected reported results and the authors described the method section clearly. Thus, we rated "Low" risk of bias in this domain.
**Overall bias**	**Risk of bias judgement**	**High**	High porportion of missing data and imbalanced missing data without sensitivity analysis put "high" risk of bias.

**Reference**	Chick 2000	**Aim**	assignment to intervention (the 'intention-to-treat' effect)	**Source**	Journal article(s) with results of the trial
**Outcome**	Abstinence (6 months)	**Results**	35/289 (Acamprosate 1998 mg) vs 32/292 (Placebo)		

**Domain**	**Signalling question**	**Response**	**Comments**
**Bias arising from the randomization process**	1.1 Was the allocation sequence random?	Y	"Patients were then reassessed and, using randomization in blocks of eight, allocated..."<br><br>"...identically presented placebo..."
	1.2 Was the allocation sequence concealed until participants were recruited and assigned to interventions?	PY	
	1.3 Were there baseline imbalances that suggest a problem with the randomization process?	N	Not significant difference between groups (Table 1)
	**Risk of bias judgement**	**Low**	Although there was no information regarding allocation concealment, this study employed adequate randomisation methods and identical placebo in the trial, which represents low risk of bias in the randomisation process.
**Bias due to**	2.1 Were participants aware of their assigned intervention during the trial?	PY	No blinding procedures were used.
	2.2 Were carers and trial personnel aware of participants' assigned intervention during the trial?	PY	
	2.3 If Y/PY/NI to 2.1 or 2.2: Were there deviations from the intended intervention beyond what would be expected in usual practice?	PN	"It was intended that the medication would be used as an adjunct, not an alternative, to the clinic's usual psychosocial out-patient treatment programme."

deviations from intended interventions	2.4 If Y/PY to 2.3: Were these deviations from intended intervention unbalanced between groups and likely to have affected the outcome?	NA	
	2.5 If N/PN/NI to 2.4: Were these deviations likely to have affected the outcome?	N	
	2.6 If Y/PY/NI to 2.5: Was there potential for a substantial impact (on the estimated effect of intervention) of analysing participants in the wrong group?	NA	
	**Risk of bias judgement**	**Low**	Although no blinding procedures were employed, this trial used identical placebo and aimed to be an adjunct, not an alternative, to the clinic's usual practice, we rated this domain as "Low" risk of bias.
**Bias due to missing outcome data**	3.1 Were outcome data available for all, or nearly all, participants randomized?	N	"Only 203 patients completed the study [A: 100 (35%), P: 103 (35%)]."
	3.2 If N/PN/NI to 3.1: Are the proportions of missing outcome data and reasons for missing outcome data similar across intervention groups?	PY	"There were no statistically significant differences in attendance between the treatment groups at any time point in the study."<br>data appear similar per arm
	3.3 If N/PN/NI to 3.1: Is there evidence that results were robust to the presence of missing outcome data?	PN	"It was assumed that all patients who terminated treatment before the end of the study, including those experiencing adverse events, were treatment failures."
	**Risk of bias judgement**	**Low**	High proportion of missing data but balanced missing data, suggesting "Low" risk of bias in this domain.
**Bias in measurement of the outcome**	4.1 Were outcome assessors aware of the intervention received by study participants?	PN	Although there is no blinding, this trial employed identical placebo and uses of an alcolmeter.
	4.2 If Y/PY/NI to 4.1: Was the assessment of the outcome likely to be influenced by knowledge of intervention received?	NA	
	**Risk of bias judgement**	**Low**	Although this study did not emphasise blinding, the outcome (abstinence) was confirmed by biochemistry test, which put low risk of bias in this domain.
**Bias in selection of the reported result**	Are the reported outcome data likely to have been selected, on the basis of the results, from…		
	5.1. ... multiple outcome measurements (e.g. scales, definitions, time points) within the outcome domain?	PN	
	5.2 ... multiple analyses of the data?	PN	
	**Risk of bias judgement**	**Low**	No protocol or statisitical analysis was found but nature of outcome, abstinence, presents low risk of selected reported results and the authors described the method section clearly. Thus, we rated "Low" risk of bias in this domain.
**Overall bias**	**Risk of bias judgement**	**Low**	Low risk of bias overall

**Reference**	Cornelius 1997	**Aim**	assignment to intervention (the 'intention-to-treat' effect)	**Source**	Journal article(s) with results of the trial
**Outcome**	Abstinence (12 weeks)	**Results**	7/25 (Fluoxetine 20 mg) vs 4/26 (Placebo)		

Domain	Signalling question	Response	Comments
**Bias arising from the randomization process**	1.1 Was the allocation sequence random?	NI	"Patient randomization was stratified for sex and race...."
	1.2 Was the allocation sequence concealed until participants were recruited and assigned to interventions?	NI	
	1.3 Were there baseline imbalances that suggest a problem with the randomization process?	N	"No significant differences were seen between treatment groups for sex, race, age, or marital or employment status."
	**Risk of bias judgement**	**Some concerns**	No details were given regarding randomisation process, contributed to "some concerns" in this domain.
**Bias due to deviations from intended interventions**	2.1 Were participants aware of their assigned intervention during the trial?	N	Double-blind
	2.2 Were carers and trial personnel aware of participants' assigned intervention during the trial?	N	
	2.3 If Y/PY/NI to 2.1 or 2.2: Were there deviations from the intended intervention beyond what would be expected in usual practice?	NA	
	2.4 If Y/PY to 2.3: Were these deviations from intended intervention unbalanced between groups and likely to have affected the outcome?	NA	
	2.5 If N/PN/NI to 2.4: Were these deviations likely to have affected the outcome?	PN	
	2.6 If Y/PY/NI to 2.5: Was there potential for a substantial impact (on the estimated effect of intervention) of analysing participants in the wrong group?	NA	
	**Risk of bias judgement**	**Low**	Double-blind design and ITT analysis employed in this trial put "Low" risk of bias in deviation from intended interventions.
**Bias due to missing outcome data**	3.1 Were outcome data available for all, or nearly all, participants randomized?	Y	"Forty-six of these patients (90% of those randomized) completed the pharmacotherapy study;<br>the other 5 patients (10%) dropped out before the end<br>of the trial."
	3.2 If N/PN/NI to 3.1: Are the proportions of missing outcome data and reasons for missing outcome data similar across intervention groups?	NA	
	3.3 If N/PN/NI to 3.1: Is there evidence that results were robust to the presence of missing outcome data?	NA	
	**Risk of bias judgement**	**Low**	Almost all outcome data were available, thus this domain was rated "Low" risk of bias.
**Bias in measurement of the outcome**	4.1 Were outcome assessors aware of the intervention received by study participants?	PN	The one year "evaluations of current symptoms were conducted by an interviewer who had been kept blind to the original assessment of protocol medication and to any subsequent medication use."
	4.2 If Y/PY/NI to 4.1: Was the assessment of the outcome likely to be influenced by knowledge of intervention received?	NA	
	**Risk of bias judgement**	**Low**	Double-blind design put this domain as low risk of bias.
**Bias in selection of the reported result**	Are the reported outcome data likely to have been selected, on the basis of the results, from…		
	5.1. ... multiple outcome measurements (e.g. scales, definitions, time points) within the outcome domain?	NI	
	5.2 ... multiple analyses of the data?	NI	
	**Risk of bias judgement**	**Low**	No protocol was found but nature of outcome, abstinence, presents low risk of selected reported results and the authors described the method section clearly. Thus, we rated "Low" risk of bias in this domain.
**Overall bias**	**Risk of bias judgement**	**Some concerns**	Lack of detailed methods for the randomisation process contributed to "some concerns" in overall bias for this trial.

**Reference**	Croissant 2006	**Aim**	assignment to intervention (the 'intention-to-treat' effect)	**Source**	Journal article(s) with results of the trial
**Outcome**	Abstinence (24 weeks)	**Results**	2/15 (Acamprosate 1998 mg) vs 4/15 (Oxcarbazepine 1200 mg)		

Domain	Signalling question	Response	Comments
	1.1 Was the allocation sequence random?	NI	only stated "random [illegible]

**Bias arising from the randomization process**	1.2 Was the allocation sequence concealed until participants were recruited and assigned to interventions?	NI	only stated "random"
	1.3 Were there baseline imbalances that suggest a problem with the randomization process?	N	No significant imbalance between groups.
	**Risk of bias judgement**	**Some concerns**	No details were given regarding randomisation process, contributed to "some concerns" in this domain.
**Bias due to deviations from intended interventions**	2.1 Were participants aware of their assigned intervention during the trial?	Y	"...we conducted an open-label, ..."
	2.2 Were carers and trial personnel aware of participants' assigned intervention during the trial?	Y	
	2.3 If Y/PY/NI to 2.1 or 2.2: Were there deviations from the intended intervention beyond what would be expected in usual practice?	NI	
	2.4 If Y/PY to 2.3: Were these deviations from intended intervention unbalanced between groups and likely to have affected the outcome?	NA	
	2.5 If N/PN/NI to 2.4: Were these deviations likely to have affected the outcome?	N	
	2.6 If Y/PY/NI to 2.5: Was there potential for a substantial impact (on the estimated effect of intervention) of analysing participants in the wrong group?	NA	
	**Risk of bias judgement**	**Some concerns**	The open label design in this trial might prompt deviations from the intended interventions, contributing to "some concerns" in this domain.
**Bias due to missing outcome data**	3.1 Were outcome data available for all, or nearly all, participants randomized?	PN	Only 10/30 completed the study (24 weeks) and 4 lost without any information supplied.
	3.2 If N/PN/NI to 3.1: Are the proportions of missing outcome data and reasons for missing outcome data similar across intervention groups?	NI	
	3.3 If N/PN/NI to 3.1: Is there evidence that results were robust to the presence of missing outcome data?	NI	
	**Risk of bias judgement**	**Some concerns**	High porportion of missing data and no detailed reasons for missing data put "some concerns" in this domain.
**Bias in measurement of the outcome**	4.1 Were outcome assessors aware of the intervention received by study participants?	PY	Drinking data collection was performed by a trained research assistant who was unblinded to treatment assignment, but not involved in the patient treatment.'
	4.2 If Y/PY/NI to 4.1: Was the assessment of the outcome likely to be influenced by knowledge of intervention received?	PN	
	**Risk of bias judgement**	**Low**	Although this is an open study, the outcome (abstinence) is not influenced by knowledge of intervention received.
**Bias in selection of the reported result**	Are the reported outcome data likely to have been selected, on the basis of the results, from…		
	5.1. ... multiple outcome measurements (e.g. scales, definitions, time points) within the outcome domain?	PN	
	5.2 ... multiple analyses of the data?	PN	
	**Risk of bias judgement**	**Low**	No protocol was found but nature of outcome, abstinence, presents low risk of selected reported results and the authors described the method section clearly. Thus, we rated "Low" risk of bias in this domain.
**Overall bias**	**Risk of bias judgement**	**Some concerns**	Lack of detailed randomisation process, potential deviations from the intended interventions due to difference among interventions and missing data, together, these contributed to "some concerns" in overall bias for this trial.

**Reference**	Favre 1997	**Aim**	assignment to intervention (the 'intention-to-treat' effect)	**Source**	Journal article(s) with results of the trial
**Outcome**	Abstinence (9 months)	**Results**	42/172 (Placebo) vs 48/170 (Tianeptine 37.5 mg)		

Domain	Signalling question	Response	Comments
**Bias arising from the randomization process**	1.1 Was the allocation sequence random?	NI	only stated "random"
	1.2 Was the allocation sequence concealed until participants were recruited and assigned to interventions?	NI	
	1.3 Were there baseline imbalances that suggest a problem with the randomization process?	PN	A difference in previous alcohol withdrawals was noted but might due to chance
	**Risk of bias judgement**	**Some concerns**	No details were given regarding randomisation process, contributed to "some concerns" in this domain.
**Bias due to deviations from intended interventions**	2.1 Were participants aware of their assigned intervention during the trial?	PN	"double-blind"
	2.2 Were carers and trial personnel aware of participants' assigned intervention during the trial?	PN	
	2.3 If Y/PY/NI to 2.1 or 2.2: Were there deviations from the intended intervention beyond what would be expected in usual practice?	NA	
	2.4 If Y/PY to 2.3: Were these deviations from intended intervention unbalanced between groups and likely to have affected the outcome?	NA	
	2.5 If N/PN/NI to 2.4: Were these deviations likely to have affected the outcome?	N	
	2.6 If Y/PY/NI to 2.5: Was there potential for a substantial impact (on the estimated effect of intervention) of analysing participants in the wrong group?	NA	
	**Risk of bias judgement**	**Low**	Double-blind design and ITT analysis employed in this trial put low risk of bias in deviation from intended interventions.
**Bias due to missing outcome data**	3.1 Were outcome data available for all, or nearly all, participants randomized?	N	High rate of drop-outs - 60.2% patients permaturely discontinued
	3.2 If N/PN/NI to 3.1: Are the proportions of missing outcome data and reasons for missing outcome data similar across intervention groups?	PN	"the premature terminations were more frequent in the tianeptine group (65.9% vs. 54.7%; P=0.04). "<br><br>No reasons of missing data were given.
	3.3 If N/PN/NI to 3.1: Is there evidence that results were robust to the presence of missing outcome data?	PY	They presented results by per protocol and ITT analyses, which led to the same results.
	**Risk of bias judgement**	**Some concerns**	High porportion of missing data and imbalanced missing data without proper sensitivity analyses put "some concerns" risk of bias in this domain.
**Bias in measurement of the outcome**	4.1 Were outcome assessors aware of the intervention received by study participants?	PN	Self-reported and checked by biological results: Abstinence was determined if the patient said he had no more than one drink since the last visit, the GGT level and the mean corpuscular volume were normal, or had not increased since the last examination, and the blood alcohol was lower than 0.10 g/ l.
	4.2 If Y/PY/NI to 4.1: Was the assessment of the outcome likely to be influenced by knowledge of intervention received?	NA	
	**Risk of bias judgement**	**Low**	Double-blind design and self-reporting outcome (confirmed by biochemistry results) put this domain as low risk of bias.
**Bias in selection of the reported result**	Are the reported outcome data likely to have been selected, on the basis of the results, from…		
	5.1. ... multiple outcome measurements (e.g. scales, definitions, time points) within the outcome domain?	PN	
	5.2 ... multiple analyses of the data?	PN	
	**Risk of bias judgement**	**Low**	No protocol was found but nature of outcome, abstinence, presents low risk of selected reported results and the authors described the method section clearly. Thus, we rated "Low" risk of bias in this domain.
**Overall bias**	**Risk of bias judgement**	**Some concerns**	High proportion of missing data and lack of detailed randomisation process contributed to "Some concerns" risk of bias in overall bias.

Reference	Florez 2008	Aim	assignment to intervention (the 'intention-to-treat' effect)	Source	Journal article(s) with results of the trial
Outcome	Abstinence (6 months)	Results	23/51 (Naltrexone 50 mg) vs 24/51 (Topiramate)		

Domain	Signalling question	Response	Comments
Bias arising from the randomization process	1.1 Was the allocation sequence random?	NI	Only stated "random"
	1.2 Was the allocation sequence concealed until participants were recruited and assigned to interventions?	NI	
	1.3 Were there baseline imbalances that suggest a problem with the randomization process?	N	51 vs 51
	**Risk of bias judgement**	**Some concerns**	No details were given regarding randomisation process, contributed to "some concerns" in this domain.
Bias due to deviations from intended interventions	2.1 Were participants aware of their assigned intervention during the trial?	Y	"open-label"
	2.2 Were carers and trial personnel aware of participants' assigned intervention during the trial?	Y	
	2.3 If Y/PY/NI to 2.1 or 2.2: Were there deviations from the intended intervention beyond what would be expected in usual practice?	PY	Naltrexone is used for alcohol dependence treatment but no topiramate. Also, more patients taking disulfiram and drop-out in naltrexone group.<br><br>"mean number of psychotherapy sessions (8.61 for naltrexone; 9.20 for topiramate); patients taking disulfiram (6 patients, 11.76%, for naltrexone; 3 patients, 5.88% for topiramate); drop-outs (6 patients, 11.76%, for naltrexone; 4 patients, 7.84% for topiramate)."
	2.4 If Y/PY to 2.3: Were these deviations from intended intervention unbalanced between groups and likely to have affected the outcome?	PY	As they are related to outcome.
	2.5 If N/PN/NI to 2.4: Were these deviations likely to have affected the outcome?	N	
	2.6 If Y/PY/NI to 2.5: Was there potential for a substantial impact (on the estimated effect of intervention) of analysing participants in the wrong group?	NA	
	**Risk of bias judgement**	**High**	Open-label design and potential deviation evidence from the number of patients taking disulfiram, suggesting high risk of bias in this domain.
Bias due to missing outcome data	3.1 Were outcome data available for all, or nearly all, participants randomized?	PY	Almost all outcome data were available (6/51 patients, 11.76%, for naltrexone; 4/51 patients, 7.84% for topiramate).
	3.2 If N/PN/NI to 3.1: Are the proportions of missing outcome data and reasons for missing outcome data similar across intervention groups?	NA	
	3.3 If N/PN/NI to 3.1: Is there evidence that results were robust to the presence of missing outcome data?	NA	
	**Risk of bias judgement**	**Low**	Almost all outcome data were available, thus this domain was rated "Low" risk of bias.
Bias in measurement of the outcome	4.1 Were outcome assessors aware of the intervention received by study participants?	Y	"Open-label" and self-reported outcome
	4.2 If Y/PY/NI to 4.1: Was the assessment of the outcome likely to be influenced by knowledge of intervention received?	PN	Abstinence was assessed by the participants and significant one with a clear definition.<br>"Alcohol intake was assessed at each treatment session. Both the patient and the significant other were interviewed and the highest intake level reported was used."
	**Risk of bias judgement**	**Low**	Although this is an open study, the outcome (abstinence) was confirmed by family member, which put low risk of bias in this domain.
Bias in selection of the reported result	Are the reported outcome data likely to have been selected, on the basis of the results, from…		
	5.1. ... multiple outcome measurements (e.g. scales, definitions, time points) within the outcome domain?	N	
	5.2 ... multiple analyses of the data?	N	
	**Risk of bias judgement**	**Low**	No protocol was found but nature of outcome, abstinence, presents low risk of selected reported results and the authors described the method section clearly. Thus, we rated "Low" risk of bias in this domain.
Overall bias	**Risk of bias judgement**	**High**	High risk of bias in deviations from the intended interventions contributed to "High" risk of bias in overall bias.

Reference	Friedmann 2008	Aim	assignment to intervention (the 'intention-to-treat' effect)	Source	Journal article(s) with results of the trial
Outcome	Abstinence (6 months)	Results	12/85 (Placebo) vs 8/88 (Trazodone)		

Domain	Signalling question	Response	Comments
Bias arising from the randomization process	1.1 Was the allocation sequence random?	Y	"Urn randomization software allocated subjects..."
	1.2 Was the allocation sequence concealed until participants were recruited and assigned to interventions?	NI	
	1.3 Were there baseline imbalances that suggest a problem with the randomization process?	PN	"The trazodone (N = 88) and placebo groups (N = 85) did not differ at baseline on any measured characteristic (Table 1)."
	**Risk of bias judgement**	**Some concerns**	No details were given regarding randomisation process, contributed to "some concerns" in this domain.
Bias due to deviations from intended interventions	2.1 Were participants aware of their assigned intervention during the trial?	PN	"double-blind" "identifical placebo"
	2.2 Were carers and trial personnel aware of participants' assigned intervention during the trial?	PN	
	2.3 If Y/PY/NI to 2.1 or 2.2: Were there deviations from the intended intervention beyond what would be expected in usual practice?	NA	
	2.4 If Y/PY to 2.3: Were these deviations from intended intervention unbalanced between groups and likely to have affected the outcome?	NA	
	2.5 If N/PN/NI to 2.4: Were these deviations likely to have affected the outcome?	N	
	2.6 If Y/PY/NI to 2.5: Was there potential for a substantial impact (on the estimated effect of intervention) of analysing participants in the wrong group?	NA	
	**Risk of bias judgement**	**Low**	Double-blind design and ITT analysis employed in this trial put low risk of bias in deviation from intended interventions.
Bias due to missing outcome data	3.1 Were outcome data available for all, or nearly all, participants randomized?	N	Missing data: 16/88 t vs 16/85 p, which is around 18%.
	3.2 If N/PN/NI to 3.1: Are the proportions of missing outcome data and reasons for missing outcome data similar across intervention groups?	Y	From Fig 1 consort chart.
	3.3 If N/PN/NI to 3.1: Is there evidence that results were robust to the presence of missing outcome data?	PY	"Mixed linear regression analyses with full information maximum likelihood (FIML) estimation compared the other drinking outcome trajectories by treatment condition across baseline, 1-, 3-, and 6-month intervals." "For the measure of complete abstinence, those who did not complete all follow-ups were assumed to have resumed drinking, and were therefore coded as not having achieved complete abstinence by the end of the study."
	**Risk of bias judgement**	**Low**	Although there were some missing data, the authors used mixed linear regression analyses and conservative approach to analysis their results, resulting "Low" risk of bias in this domain.

Bias in measurement of the outcome	4.1 Were outcome assessors aware of the intervention received by study participants?	PN	"double-blind" & Self-reported outcome
	4.2 If Y/PY/NI to 4.1: Was the assessment of the outcome likely to be influenced by knowledge of intervention received?	NA	
	**Risk of bias judgement**	**Low**	Double-blind design and self-reporting outcome put this domain as low risk of bias.
Bias in selection of the reported result	Are the reported outcome data likely to have been selected, on the basis of the results, from…		
	5.1. ... multiple outcome measurements (e.g. scales, definitions, time points) within the outcome domain?	PN	
	5.2 ... multiple analyses of the data?	PN	
	**Risk of bias judgement**	**Low**	No protocol was found but nature of outcome, abstinence, presents low risk of selected reported results and the authors described the method section clearly. Thus, we rated "Low" risk of bias in this domain.
**Overall bias**	**Risk of bias judgement**	**Some concerns**	Lack of detailed method for the randomisation process contributed to "some concerns" in overall bias for this trial.

**Reference**	Fuente 1989	**Aim**	assignment to intervention (the 'intention-to-treat' effect)	**Source**	Journal article(s) with results of the trial
**Outcome**	Abstinence (6 months)	**Results**	10/28 (Lithium) vs 7/25 (Placebo)		

Domain	Signalling question	Response	Comments
Bias arising from the randomization process	1.1 Was the allocation sequence random?	NI	Only stated "random"
	1.2 Was the allocation sequence concealed until participants were recruited and assigned to interventions?	NI	
	1.3 Were there baseline imbalances that suggest a problem with the randomization process?	NI	Did not provide basic characteristics of participants by groups
	**Risk of bias judgement**	**Some concerns**	No details were given regarding randomisation process and characteristics of participants by group, contributed to "some concerns" in this domain.
Bias due to deviations from intended interventions	2.1 Were participants aware of their assigned intervention during the trial?	PN	"The patient and the primary investigator remained blind to the lithium or placebo assignment."
	2.2 Were carers and trial personnel aware of participants' assigned intervention during the trial?	PN	
	2.3 If Y/PY/NI to 2.1 or 2.2: Were there deviations from the intended intervention beyond what would be expected in usual practice?	NA	
	2.4 If Y/PY to 2.3: Were these deviations from intended intervention unbalanced between groups and likely to have affected the outcome?	NA	
	2.5 If N/PN/NI to 2.4: Were these deviations likely to have affected the outcome?	N	
	2.6 If Y/PY/NI to 2.5: Was there potential for a substantial impact (on the estimated effect of intervention) of analysing participants in the wrong group?	NA	
	**Risk of bias judgement**	**Low**	Double-blind design and ITT analysis employed in this trial put low risk of bias in deviation from intended interventions.
Bias due to missing outcome data	3.1 Were outcome data available for all, or nearly all, participants randomized?	PY	Only half of participants completed the trial but the authors verified those patients dropped out.
	3.2 If N/PN/NI to 3.1: Are the proportions of missing outcome data and reasons for missing outcome data similar across intervention groups?	NA	
	3.3 If N/PN/NI to 3.1: Is there evidence that results were robust to the presence of missing outcome data?	NA	
	**Risk of bias judgement**	**Low**	Outcome data were available since the authors tried to verified patient's drinking conditions, contributing to "Low" risk of bias in this domain
Bias in measurement of the outcome	4.1 Were outcome assessors aware of the intervention received by study participants?	PN	"double-blind" & self-report
	4.2 If Y/PY/NI to 4.1: Was the assessment of the outcome likely to be influenced by knowledge of intervention received?	NA	
	**Risk of bias judgement**	**Low**	Patients, as the outcome assessor, were blinded, thus this domain was rated as low risk of bias.
Bias in selection of the reported result	Are the reported outcome data likely to have been selected, on the basis of the results, from…		
	5.1. ... multiple outcome measurements (e.g. scales, definitions, time points) within the outcome domain?	PN	
	5.2 ... multiple analyses of the data?	PN	
	**Risk of bias judgement**	**Low**	No protocol was found but nature of outcome, abstinence, presents low risk of selected reported results and the authors described the method section clearly. Thus, we rated "Low" risk of bias in this domain.
**Overall bias**	**Risk of bias judgement**	**Some concerns**	Lack of detailed methods for the randomisation process contributed to "some concerns" in overall bias for this trial.

**Reference**	Fuller 1986	**Aim**	assignment to intervention (the 'intention-to-treat' effect)	**Source**	Journal article(s) with results of the trial
**Outcome**	Abstinence (12 months)	**Results**	78/403 (Combined Disulfiram 1 mg + Placebo) vs 38/202 (Disulfiram 250 mg)		

Domain	Signalling question	Response	Comments
Bias arising from the randomization process	1.1 Was the allocation sequence random?	PY	"Treatment assignment was done by opening sequentially numbered envelopes based on a randomization list."
	1.2 Was the allocation sequence concealed until participants were recruited and assigned to interventions?	Y	
	1.3 Were there baseline imbalances that suggest a problem with the randomization process?	PN	
	**Risk of bias judgement**	**Low**	This study employed adequate randomisation methods in the trial and represents low risk of bias in the randomisation process.
Bias due to deviations from intended interventions	2.1 Were participants aware of their assigned intervention during the trial?	PN	"double-blind" in the experimental arms but not control (riboflavin); all treatment personnel and research assistants were blinded.
	2.2 Were carers and trial personnel aware of participants' assigned intervention during the trial?	N	
	2.3 If Y/PY/NI to 2.1 or 2.2: Were there deviations from the intended intervention beyond what would be expected in usual practice?	NA	
	2.4 If Y/PY to 2.3: Were these deviations from intended intervention unbalanced between groups and likely to have affected the outcome?	NA	
	2.5 If N/PN/NI to 2.4: Were these deviations likely to have affected the outcome?	N	

	2.6 If Y/PY/NI to 2.5: Was there potential for a substantial impact (on the estimated effect of intervention) of analysing participants in the wrong group?	NA	
	**Risk of bias judgement**	**Low**	Double-blind design and ITT analysis employed in this trial put low risk of bias in deviation from intended interventions.
**Bias due to missing outcome data**	3.1 Were outcome data available for all, or nearly all, participants randomized?	PN	There were around 10% of patients in each group with insufficient data to evaluate abstinence (Figure 1)
	3.2 If N/PN/NI to 3.1: Are the proportions of missing outcome data and reasons for missing outcome data similar across intervention groups?	NI	The proportion of drop-outs seems to be similar between groups but no details were given.
	3.3 If N/PN/NI to 3.1: Is there evidence that results were robust to the presence of missing outcome data?	PN	Conservative analysis by assumming dropped out patients as "failed"
	**Risk of bias judgement**	**Some concerns**	Although there were some missing data, the proportion of drop-outs seemed to be similar but no details were given. The authors did not run sensitivity analyses but they assummed drop-outs as failed. "Some concerns" risk of bias in this domain was rated.
**Bias in measurement of the outcome**	4.1 Were outcome assessors aware of the intervention received by study participants?	N	Abstinence (drinking) was reported by relative's/friend's interviews or from the urine or blood specimens.
	4.2 If Y/PY/NI to 4.1: Was the assessment of the outcome likely to be influenced by knowledge of intervention received?	NA	
	**Risk of bias judgement**	**Low**	Double-blind design and self-reporting outcome (confirmed by relatives and biochemistry results) put this domain as low risk of bias.
**Bias in selection of the reported result**	Are the reported outcome data likely to have been selected, on the basis of the results, from…		
	5.1. ... multiple outcome measurements (e.g. scales, definitions, time points) within the outcome domain?	PN	
	5.2 ... multiple analyses of the data?	PN	
	**Risk of bias judgement**	**Low**	No protocol was found but nature of outcome, abstinence, presents low risk of selected reported results and the authors described the method section clearly. Thus, we rated "Low" risk of bias in this domain.
**Overall bias**	**Risk of bias judgement**	**Some concerns**	Some concerns overall due to the way of authors handling with missing data.

**Reference**	Geerlings 1997	**Aim**	assignment to intervention (the 'intention-to-treat' effect)	**Source**	Journal article(s) with results of the trial
**Outcome**	Abstinence (1 year)	**Results**	14/128 (Acamprosate 1998 mg) vs 7/134 (Placebo)		

**Domain**	**Signalling question**	**Response**	**Comments**
**Bias arising from the randomization process**	1.1 Was the allocation sequence random?	Y	"...were randomly assigned (balanced randomisation in groups of 4+4) to the study treatment..."<br>"...it appeared that many subjects only came for the medication and/or the results of blood test rsuling in minimal contact with the clinic..."<br>"...double-blind"
	1.2 Was the allocation sequence concealed until participants were recruited and assigned to interventions?	PY	
	1.3 Were there baseline imbalances that suggest a problem with the randomization process?	N	Participants in placebo group seemed to be able to stay alcohol-free longer than participants in acamporsate group, which might be resulted from chance
	**Risk of bias judgement**	**Low**	This study employed adequate randomisation methods in the trial and represents low risk of bias in the randomisation process.
**Bias due to deviations from intended interventions**	2.1 Were participants aware of their assigned intervention during the trial?	PN	"double-blind"
	2.2 Were carers and trial personnel aware of participants' assigned intervention during the trial?	PN	
	2.3 If Y/PY/NI to 2.1 or 2.2: Were there deviations from the intended intervention beyond what would be expected in usual practice?	NA	
	2.4 If Y/PY to 2.3: Were these deviations from intended intervention unbalanced between groups and likely to have affected the outcome?	NA	
	2.5 If N/PN/NI to 2.4: Were these deviations likely to have affected the outcome?	N	
	2.6 If Y/PY/NI to 2.5: Was there potential for a substantial impact (on the estimated effect of intervention) of analysing participants in the wrong group?	NA	
	**Risk of bias judgement**	**Low**	Double-blind design and ITT analysis employed in this trial put low risk of bias in deviation from intended interventions.
**Bias due to missing outcome data**	3.1 Were outcome data available for all, or nearly all, participants randomized?	N	More than 64% of participant left prematurely.
	3.2 If N/PN/NI to 3.1: Are the proportions of missing outcome data and reasons for missing outcome data similar across intervention groups?	PN	"30 of the 128 from the acamprosate group (23%) and 23 of the 134 from the placebo group (17%) completed treatment"<br>No details on reasons for missing outcomes for this period.
	3.3 If N/PN/NI to 3.1: Is there evidence that results were robust to the presence of missing outcome data?	PN	Drop-outs were assumed as relapsed.
	**Risk of bias judgement**	**Some concerns**	High porportion of missing data without sensitivity analysis put "some concerns" risk of bias in this domain.
**Bias in measurement of the outcome**	4.1 Were outcome assessors aware of the intervention received by study participants?	PN	"double-blind"
	4.2 If Y/PY/NI to 4.1: Was the assessment of the outcome likely to be influenced by knowledge of intervention received?	NA	
	**Risk of bias judgement**	**Low**	Double-blind design and self-reporting outcome put this domain as low risk of bias.
**Bias in selection of the reported result**	Are the reported outcome data likely to have been selected, on the basis of the results, from…		
	5.1. ... multiple outcome measurements (e.g. scales, definitions, time points) within the outcome domain?	PN	
	5.2 ... multiple analyses of the data?	PN	
	**Risk of bias judgement**	**Low**	No protocol was found but nature of outcome, abstinence, presents low risk of selected reported results and the authors described the method section clearly. Thus, we rated "Low" risk of bias in this domain.
**Overall bias**	**Risk of bias judgement**	**Some concerns**	Some concerns overall due to high proportion of missing outcomes without sensitivity analysis

**Reference**	Gottlieb 1994	**Aim**	assignment to intervention (the 'intention-to-treat' effect)	**Source**	Journal article(s) with results of the trial
**Outcome**	Abstinence (1 year)	**Results**	7/50 (Atenolol 100mg) vs 8/50 (Placebo)		

**Domain**	**Signalling question**	**Response**	**Comments**
**Bias arising from the randomization**	1.1 Was the allocation sequence random?	Y	"Randomization with a block size of 8 was carried out under the direction of one of us (LDG)."<br>"Neither the clinical personnel (physicians and counselors) nor the patients were aware of the subjects' treatment assignment."
	1.2 Was the allocation sequence concealed until participants were recruited and assigned to interventions?	NI	

the randomization process	1.3 Were there baseline imbalances that suggest a problem with the randomization process?	PN	Craving for alcohol higher in the atenolol group, but no other differences so probably due to chance.
	**Risk of bias judgement**	**Some concerns**	No details were given regarding randomisation process, contributed to "some concerns" in this domain.
**Bias due to deviations from intended interventions**	2.1 Were participants aware of their assigned intervention during the trial?	N	"double-blind"
	2.2 Were carers and trial personnel aware of participants' assigned intervention during the trial?	N	
	2.3 If Y/PY/NI to 2.1 or 2.2: Were there deviations from the intended intervention beyond what would be expected in usual practice?	NA	
	2.4 If Y/PY to 2.3: Were these deviations from intended intervention unbalanced between groups and likely to have affected the outcome?	NA	
	2.5 If N/PN/NI to 2.4: Were these deviations likely to have affected the outcome?	N	Doesn't specifically state ITT but no patients were analysed in a group different to the one to which they were assigned. "Patients who did not return for follow-up and could not be contacted were presumed to have returned to drinking and were counted as treatment failures."
	2.6 If Y/PY/NI to 2.5: Was there potential for a substantial impact (on the estimated effect of intervention) of analysing participants in the wrong group?	NA	
	**Risk of bias judgement**	**Low**	Double-blind design and ITT analysis employed in this trial put "Low" risk of bias in deviation from intended interventions.
**Bias due to missing outcome data**	3.1 Were outcome data available for all, or nearly all, participants randomized?	N	The authors reported drinking status in all participants
	3.2 If N/PN/NI to 3.1: Are the proportions of missing outcome data and reasons for missing outcome data similar across intervention groups?	PN	Numbers who dropped out, presumed drinking slightly higher in the placebo group. Numbers who withdrew whilst not drinking: higher in the atenolol group (17 vs. 13).
	3.3 If N/PN/NI to 3.1: Is there evidence that results were robust to the presence of missing outcome data?	PN	May have led to a conservate estimate of the effect of atenolol.
	**Risk of bias judgement**	**High**	High porportion of missing data and imbalanced missing data without sensitivity analysis put "high" risk of bias in this domain.
**Bias in measurement of the outcome**	4.1 Were outcome assessors aware of the intervention received by study participants?	N	"double-blind"
	4.2 If Y/PY/NI to 4.1: Was the assessment of the outcome likely to be influenced by knowledge of intervention received?	NA	
	**Risk of bias judgement**	**Low**	Double-blind design and self-reporting outcome put this domain as low risk of bias.
**Bias in selection of the reported result**	Are the reported outcome data likely to have been selected, on the basis of the results, from…		
	5.1. ... multiple outcome measurements (e.g. scales, definitions, time points) within the outcome domain?	N	
	5.2 ... multiple analyses of the data?	N	
	**Risk of bias judgement**	**Low**	No protocol was found but nature of outcome, abstinence, presents low risk of selected reported results and the authors described the method section clearly. Thus, we rated "Low" risk of bias in this domain.
**Overall bias**	**Risk of bias judgement**	**High**	High risk of bias in missing data and some concerns in randomisation process contributed to "High" risk of bias in overall bias.

**Reference**	Gual 2001	**Aim**	assignment to intervention (the 'intention-to-treat' effect)	**Source**	Journal article(s) with results of the trial
**Outcome**	Abstinence (180 days)	**Results**	35/141 (Acamprosate 1998 mg) vs 26/147 (Placebo)		

Domain	Signalling question	Response	Comments
**Bias arising from the randomization process**	1.1 Was the allocation sequence random?	NI	Only stated "random", "double-blind"
	1.2 Was the allocation sequence concealed until participants were recruited and assigned to interventions?	NI	
	1.3 Were there baseline imbalances that suggest a problem with the randomization process?	N	
	**Risk of bias judgement**	**Some concerns**	No details were given regarding randomisation process, contributed to "some concerns" in this domain.
**Bias due to deviations from intended interventions**	2.1 Were participants aware of their assigned intervention during the trial?	PN	"double-blind"
	2.2 Were carers and trial personnel aware of participants' assigned intervention during the trial?	PN	
	2.3 If Y/PY/NI to 2.1 or 2.2: Were there deviations from the intended intervention beyond what would be expected in usual practice?	NA	
	2.4 If Y/PY to 2.3: Were these deviations from intended intervention unbalanced between groups and likely to have affected the outcome?	NA	
	2.5 If N/PN/NI to 2.4: Were these deviations likely to have affected the outcome?	N	No, mITT (at least one dose) employed. No swapping of patients between groups.
	2.6 If Y/PY/NI to 2.5: Was there potential for a substantial impact (on the estimated effect of intervention) of analysing participants in the wrong group?	NA	
	**Risk of bias judgement**	**Low**	Double-blind design and mITT analysis employed in this trial put low risk of bias in deviation from intended interventions.
**Bias due to missing outcome data**	3.1 Were outcome data available for all, or nearly all, participants randomized?	N	More participants lost to follow-up and refused to comtinue in the placebo group. And more participants dropped before started the trial.
	3.2 If N/PN/NI to 3.1: Are the proportions of missing outcome data and reasons for missing outcome data similar across intervention groups?	PN	Appears that % and reasons balanced between groups, with sole exception of "No data after baseline"
	3.3 If N/PN/NI to 3.1: Is there evidence that results were robust to the presence of missing outcome data?	PY	The authors presented both ITT and pre-protocol analysis results, which led same conclusion.
	**Risk of bias judgement**	**Low**	Although there were some missing data, results still stood in consideration of missing data. "Low" risk of bias in this domain was rated.
**Bias in measurement of the outcome**	4.1 Were outcome assessors aware of the intervention received by study participants?	PN	"double-blind"
	4.2 If Y/PY/NI to 4.1: Was the assessment of the outcome likely to be influenced by knowledge of intervention received?	NA	
	**Risk of bias judgement**	**Low**	Double-blind design and self-reporting outcome put this domain as low risk of bias.
**Bias in selection of the reported result**	Are the reported outcome data likely to have been selected, on the basis of the results, from…		
	5.1. ... multiple outcome measurements (e.g. scales, definitions, time points) within the outcome domain?	PN	
	5.2 ... multiple analyses of the data?	PN	
	**Risk of bias judgement**	**Low**	No protocol was found but nature of outcome, abstinence, presents low risk of selected reported results and the authors described the method section clearly. Thus, we rated "Low" risk of bias in this domain.
**Overall bias**	**Risk of bias judgement**	**Some concerns**	Lack of detailed methods for the randomisation process these contributed to "some concerns" in overall bias for this trial.

Reference	Gual 2002	Aim	assignment to intervention (the 'intention-to-treat' effect)	Source	Journal article(s) with results of the trial
Outcome	Abstinence (180 days)	Results	19/43 (Placebo) vs 11/38 (Tiapride 300mg)		

Domain	Signalling question	Response	Comments
Bias arising from the randomization process	1.1 Was the allocation sequence random?	NI	only stated "random", "double-blind"
	1.2 Was the allocation sequence concealed until participants were recruited and assigned to interventions?	NI	
	1.3 Were there baseline imbalances that suggest a problem with the randomization process?	PN	Table 2 doesn't show any difference.
	**Risk of bias judgement**	**Some concerns**	No details were given regarding randomisation process, contributed to "some concerns" in this domain.
Bias due to deviations from intended interventions	2.1 Were participants aware of their assigned intervention during the trial?	PN	"double-blind"
	2.2 Were carers and trial personnel aware of participants' assigned intervention during the trial?	PN	
	2.3 If Y/PY/NI to 2.1 or 2.2: Were there deviations from the intended intervention beyond what would be expected in usual practice?	NA	
	2.4 If Y/PY to 2.3: Were these deviations from intended intervention unbalanced between groups and likely to have affected the outcome?	NA	
	2.5 If N/PN/NI to 2.4: Were these deviations likely to have affected the outcome?	N	
	2.6 If Y/PY/NI to 2.5: Was there potential for a substantial impact (on the estimated effect of intervention) of analysing participants in the wrong group?	NA	
	**Risk of bias judgement**	**Low**	Double-blind design and ITT analysis employed in this trial put low risk of bias in deviation from intended interventions.
Bias due to missing outcome data	3.1 Were outcome data available for all, or nearly all, participants randomized?	N	The attrition rate was higher in the placebo group (Table 4).
	3.2 If N/PN/NI to 3.1: Are the proportions of missing outcome data and reasons for missing outcome data similar across intervention groups?	PN	Imbalanced reasons between groups
	3.3 If N/PN/NI to 3.1: Is there evidence that results were robust to the presence of missing outcome data?	PY	The authors performed analyses included and excluded patients dropped out, leading to the same conclusion.
	**Risk of bias judgement**	**Low**	Although there were some missing data, results still stood in consideration of missing data. "Low" risk of bias in this domain was rated.
Bias in measurement of the outcome	4.1 Were outcome assessors aware of the intervention received by study participants?	PN	"double-blind"
	4.2 If Y/PY/NI to 4.1: Was the assessment of the outcome likely to be influenced by knowledge of intervention received?	NA	
	**Risk of bias judgement**	**Low**	Double-blind design puts this domain as low risk of bias.
Bias in selection of the reported result	Are the reported outcome data likely to have been selected, on the basis of the results, from…		
	5.1. ... multiple outcome measurements (e.g. scales, definitions, time points) within the outcome domain?	NI	
	5.2 ... multiple analyses of the data?	NI	
	**Risk of bias judgement**	**Low**	No protocol was found but nature of outcome, abstinence, presents low risk of selected reported results and the authors described the method section clearly. Thus, we rated "Low" risk of bias in this domain.
Overall bias	**Risk of bias judgement**	**Some concerns**	Lack of detailed methods for the randomisation process contributed to "some concerns" in overall bias for this trial.

Reference	Gustafson 2014	Aim	assignment to intervention (the 'intention-to-treat' effect)	Source	Journal article(s) with results of the trial
Outcome	Abstinence (12 months)	Results	81/170 (A-CHESS) vs 63/179 (Control)		

Domain	Signalling question	Response	Comments
Bias arising from the randomization process	1.1 Was the allocation sequence random?	Y	"patients were randomized to the control group or A-CHESS in a 1:1 ratio using a computer-generated random allocation sequencewith blocks of 8 .Randomizationwasimplemented usingsequentiallynumbered containers."
	1.2 Was the allocation sequence concealed until participants were recruited and assigned to interventions?	Y	
	1.3 Were there baseline imbalances that suggest a problem with the randomization process?	PN	
	**Risk of bias judgement**	**Low**	This study employed adequate randomisation methods in the trial and represents low risk of bias in the randomisation process.
Bias due to deviations from intended interventions	2.1 Were participants aware of their assigned intervention during the trial?	Y	It was impossible to blind participants due to nature of interventions.
	2.2 Were carers and trial personnel aware of participants' assigned intervention during the trial?	Y	
	2.3 If Y/PY/NI to 2.1 or 2.2: Were there deviations from the intended intervention beyond what would be expected in usual practice?	NI	
	2.4 If Y/PY to 2.3: Were these deviations from intended intervention unbalanced between groups and likely to have affected the outcome?	NA	
	2.5 If N/PN/NI to 2.4: Were these deviations likely to have affected the outcome?	N	ITT analysis
	2.6 If Y/PY/NI to 2.5: Was there potential for a substantial impact (on the estimated effect of intervention) of analysing participants in the wrong group?	NA	
	**Risk of bias judgement**	**Some concerns**	It was impossible to blind participants in this trial, which might prompt deviations from the intended interventions, contributing to "some concerns" in this domain.
Bias due to missing outcome data	3.1 Were outcome data available for all, or nearly all, participants randomized?	PN	There were around 15% of lost to follow-up
	3.2 If N/PN/NI to 3.1: Are the proportions of missing outcome data and reasons for missing outcome data similar across intervention groups?	NI	No reasons were given for missing data but the proportions were similar.
	3.3 If N/PN/NI to 3.1: Is there evidence that results were robust to the presence of missing outcome data?	PY	Yes, used mixed effect models
	**Risk of bias judgement**	**Low**	Although there were some missing data, results still stood in consideration of missing data and the authors analysed results using mixed effect models. "Low" risk of bias in this domain was rated.
Bias in measurement of the outcome	4.1 Were outcome assessors aware of the intervention received by study participants?	Y	Outcome assessors were participants.
	4.2 If Y/PY/NI to 4.1: Was the assessment of the outcome likely to be influenced by knowledge of intervention received?	PY	This is possible and self-reported outcome
	**Risk of bias judgement**	**High**	Lack of blinding to outcome assessors (participants themselve) and self-reporting outcomes, which put "High" risk of bias in this domain.
	Are the reported outcome data likely to have been selected, on the basis of the results, from…		

Bias in selection of the reported result	5.1. ... multiple outcome measurements (e.g. scales, definitions, time points) within the outcome domain?	N	
	5.2 ... multiple analyses of the data?	N	
	**Risk of bias judgement**	**Low**	No protocol was found but nature of outcome, abstinence, presents low risk of selected reported results and the authors described method section clearly. Thus, we rated "Low" risk of bias in this domain.
**Overall bias**	**Risk of bias judgement**	**High**	The nature of interventions made it was impossible to blind participants, contributing deviations from the intended interventions and outcome measurements. Overally, these result "High" risk of bias overall.

**Reference**	Huang 2005	**Aim**	assignment to intervention (the 'intention-to-treat' effect)	**Source**	Journal article(s) with results of the trial
**Outcome**	Abstinence (14 weeks)	**Results**	11/20 (Naltrexone 50mg) vs 13/20 (Placebo)		

Domain	Signalling question	Response	Comments
**Bias arising from the randomization process**	1.1 Was the allocation sequence random?	NI	Only stated "random", "double-blind"
	1.2 Was the allocation sequence concealed until participants were recruited and assigned to interventions?	NI	
	1.3 Were there baseline imbalances that suggest a problem with the randomization process?	N	"As shown in Table 1, there were no significant differences in the distribution of demographic characteristics between the naltrexone and placebo-treated groups."
	**Risk of bias judgement**	**Some concerns**	No details were given regarding randomisation process, contributed to "some concerns" in this domain.
**Bias due to deviations from intended interventions**	2.1 Were participants aware of their assigned intervention during the trial?	PN	"double-blind"
	2.2 Were carers and trial personnel aware of participants' assigned intervention during the trial?	PN	
	2.3 If Y/PY/NI to 2.1 or 2.2: Were there deviations from the intended intervention beyond what would be expected in usual practice?	NA	
	2.4 If Y/PY to 2.3: Were these deviations from intended intervention unbalanced between groups and likely to have affected the outcome?	NA	
	2.5 If N/PN/NI to 2.4: Were these deviations likely to have affected the outcome?	N	Appears to be ITT analysis, though not explicitly stated. No evidence of switches
	2.6 If Y/PY/NI to 2.5: Was there potential for a substantial impact (on the estimated effect of intervention) of analysing participants in the wrong group?	NA	
	**Risk of bias judgement**	**Low**	Double-blind design and ITT analysis employed in this trial put low risk of bias in deviation from intended interventions.
**Bias due to missing outcome data**	3.1 Were outcome data available for all, or nearly all, participants randomized?	N	"nine (45%) did not complete the study, whereas in the placebo-treated group seven (35%) of 20 subjects failed to complete the study."
	3.2 If N/PN/NI to 3.1: Are the proportions of missing outcome data and reasons for missing outcome data similar across intervention groups?	PY	"Four of the nine non-completers in the naltrexone-treated group and three of the seven noncompleters in the placebo-treated group dropped out (p = 0.671) because of alcohol relapse, as defined in this study. However, the rest of the non-completers in both groups were reluctant to continue the study."
	3.3 If N/PN/NI to 3.1: Is there evidence that results were robust to the presence of missing outcome data?	NI	No evidence of sensitivty analysis
	**Risk of bias judgement**	**Low**	Although there were some missing data, results still stood in consideration of missing data, which was balanced in both groups. "Low" risk of bias in this domain was rated.
**Bias in measurement of the outcome**	4.1 Were outcome assessors aware of the intervention received by study participants?	PN	"double-blind"
	4.2 If Y/PY/NI to 4.1: Was the assessment of the outcome likely to be influenced by knowledge of intervention received?	NA	
	**Risk of bias judgement**	**Low**	Double-blind design and self-reporting outcome (confirmed by biochemistry results) put this domain as low risk of bias.
**Bias in selection of the reported result**	Are the reported outcome data likely to have been selected, on the basis of the results, from…		
	5.1. ... multiple outcome measurements (e.g. scales, definitions, time points) within the outcome domain?	PN	
	5.2 ... multiple analyses of the data?	PN	
	**Risk of bias judgement**	**Low**	No protocol was found but nature of outcome, abstinence, presents low risk of selected reported results and the authors described the method section clearly. Thus, we rated "Low" risk of bias in this domain.
**Overall bias**	**Risk of bias judgement**	**Some concerns**	Lack of detailed methods for the randomisation process contributed to "some concerns" in overall bias for this trial.

**Reference**	Huang 2002	**Aim**	assignment to intervention (the 'intention-to-treat' effect)	**Source**	Journal article(s) with results of the trial
**Outcome**	Abstinence (3 months)	**Results**	16/23 (Naltrexone 30mg) vs 6/22 (Placebo)		

Domain	Signalling question	Response	Comments
**Bias arising from the randomization process**	1.1 Was the allocation sequence random?	Y	Block randomisation, no information on allocation concealment
	1.2 Was the allocation sequence concealed until participants were recruited and assigned to interventions?	NI	
	1.3 Were there baseline imbalances that suggest a problem with the randomization process?	N	No significant difference in outcomes measured at baseline, including alcohol consumption and craving.
	**Risk of bias judgement**	**Some concerns**	No details were given regarding randomisation process, contributed to "some concerns" in this domain.
**Bias due to deviations from intended interventions**	2.1 Were participants aware of their assigned intervention during the trial?	N	"single-blind"
	2.2 Were carers and trial personnel aware of participants' assigned intervention during the trial?	PY	
	2.3 If Y/PY/NI to 2.1 or 2.2: Were there deviations from the intended intervention beyond what would be expected in usual practice?	PN	
	2.4 If Y/PY to 2.3: Were these deviations from intended intervention unbalanced between groups and likely to have affected the outcome?	NA	
	2.5 If N/PN/NI to 2.4: Were these deviations likely to have affected the outcome?	N	
	2.6 If Y/PY/NI to 2.5: Was there potential for a substantial impact (on the estimated effect of intervention) of analysing participants in the wrong group?	NA	
	**Risk of bias judgement**	**Low**	
	3.1 Were outcome data available for all, or nearly all, participants randomized?	Y	Although a large proportion of the study group, only one patient dropped out in the naltrexone grouo and two in the placebo group. If these drops outs were all abstinent it would not change the result that natrexone is superior.

Bias due to missing outcome data	3.2 If N/PN/NI to 3.1: Are the proportions of missing outcome data and reasons for missing outcome data similar across intervention groups?	NA	
	3.3 If N/PN/NI to 3.1: Is there evidence that results were robust to the presence of missing outcome data?	NA	
	**Risk of bias judgement**	**Low**	
Bias in measurement of the outcome	4.1 Were outcome assessors aware of the intervention received by study participants?	PN	Self-reporting and "single-blind"
	4.2 If Y/PY/NI to 4.1: Was the assessment of the outcome likely to be influenced by knowledge of intervention received?	NA	
	**Risk of bias judgement**	**Low**	Single-blind design and self-reporting outcome (confirmed by relatives and biochemistry results) put this domain as low risk of bias.
Bias in selection of the reported result	Are the reported outcome data likely to have been selected, on the basis of the results, from…		
	5.1. ... multiple outcome measurements (e.g. scales, definitions, time points) within the outcome domain?	PN	
	5.2 ... multiple analyses of the data?	PN	
	**Risk of bias judgement**	**Low**	No protocol was found but nature of outcome, abstinence, presents low risk of selected reported results and the authors described the method section clearly. Thus, we rated "Low" risk of bias in this domain.
**Overall bias**	**Risk of bias judgement**	**Some concerns**	Lack of detailed methods for the randomisation process contributed to "some concerns" in overall bias for this trial.

**Reference**	Janiri 1997	**Aim**	assignment to intervention (the 'intention-to-treat' effect)	**Source**	Conference abstract(s) about the trial
**Outcome**	Abstinence (90 days)	**Results**	9/25 (Fluoxetine 20mg) vs 3/25 (Fluvoxamine 100mg)		

Domain	Signalling question	Response	Comments
Bias arising from the randomization process	1.1 Was the allocation sequence random?	PY	Only stated "random"
	1.2 Was the allocation sequence concealed until participants were recruited and assigned to interventions?	NI	
	1.3 Were there baseline imbalances that suggest a problem with the randomization process?	NI	
	**Risk of bias judgement**	**Some concerns**	Conference abstract with limited information
Bias due to deviations from intended interventions	2.1 Were participants aware of their assigned intervention during the trial?	Y	Open study
	2.2 Were carers and trial personnel aware of participants' assigned intervention during the trial?	NI	
	2.3 If Y/PY/NI to 2.1 or 2.2: Were there deviations from the intended intervention beyond what would be expected in usual practice?	NI	
	2.4 If Y/PY to 2.3: Were these deviations from intended intervention unbalanced between groups and likely to have affected the outcome?	NA	
	2.5 If N/PN/NI to 2.4: Were these deviations likely to have affected the outcome?	N	
	2.6 If Y/PY/NI to 2.5: Was there potential for a substantial impact (on the estimated effect of intervention) of analysing participants in the wrong group?	NA	
	**Risk of bias judgement**	**Some concerns**	The open label design in this trial might prompt deviations from the intended interventions, contributing to "some concerns" in this domain.
Bias due to missing outcome data	3.1 Were outcome data available for all, or nearly all, participants randomized?	N	More than half participants dropped out
	3.2 If N/PN/NI to 3.1: Are the proportions of missing outcome data and reasons for missing outcome data similar across intervention groups?	NI	Don't know drop-out rates in each group.
	3.3 If N/PN/NI to 3.1: Is there evidence that results were robust to the presence of missing outcome data?	PN	No reasons were given
	**Risk of bias judgement**	**Some concerns**	High porportion of missing data and no detailed reasons for missing data put "some concerns" in this domain.
Bias in measurement of the outcome	4.1 Were outcome assessors aware of the intervention received by study participants?	PY	Open study, self-reported outcomes
	4.2 If Y/PY/NI to 4.1: Was the assessment of the outcome likely to be influenced by knowledge of intervention received?	PY	
	**Risk of bias judgement**	**High**	Open study and self-reported outcome put this domain as "High" risk of bias
Bias in selection of the reported result	Are the reported outcome data likely to have been selected, on the basis of the results, from…		
	5.1. ... multiple outcome measurements (e.g. scales, definitions, time points) within the outcome domain?	NI	
	5.2 ... multiple analyses of the data?	NI	
	**Risk of bias judgement**	**Some concerns**	No protocol was found and insufficient information available. Thus, we rated "Some concerns" risk of bias in this domain.
**Overall bias**	**Risk of bias judgement**	**High**	Lack of information as a conference abstract put this trial as "high" risk of bias

**Reference**	Joos 2013	**Aim**	assignment to intervention (the 'intention-to-treat' effect)	**Source**	Journal article(s) with results of the trial
**Outcome**	Abstinence (8.5 months)	**Results**	12/41 (Modafinil 300 mg) vs 6/42 (Placebo)		

Domain	Signalling question	Response	Comments
Bias arising from the randomization process	1.1 Was the allocation sequence random?	Y	"A stratified, permuted block randomization was used with gender as the only stratum and blocks contained random sizes of 2, 4 or 6 allocations for males, and 2 or 4 allocations for females. Personnel, not associated with the wards involved in the study, generated the allocation sequence by using 'Random Allocation Software' (Saghaei, 2004) and assigned the patients to one of the 2 treatment groups. Only these persons and the involved pharmacists were aware of the medication assignment."
	1.2 Was the allocation sequence concealed until participants were recruited and assigned to interventions?	Y	
	1.3 Were there baseline imbalances that suggest a problem with the randomization process?	N	No significant difference between groups.
	**Risk of bias judgement**	**Low**	This study employed adequate randomisation methods in the trial and represents low risk of bias in the randomisation process.
	2.1 Were participants aware of their assigned intervention during the trial?	N	"Group allocation was blind for both the participants and the researchers or care providers, who enrolled, treated, or assessed the patients."
	2.2 Were carers and trial personnel aware of participants' assigned intervention during the trial?	N	

Bias due to deviations from intended interventions	2.3 If Y/PY/NI to 2.1 or 2.2: Were there deviations from the intended intervention beyond what would be expected in usual practice?	NA	
	2.4 If Y/PY to 2.3: Were these deviations from intended intervention unbalanced between groups and likely to have affected the outcome?	NA	
	2.5 If N/PN/NI to 2.4: Were these deviations likely to have affected the outcome?	PN	
	2.6 If Y/PY/NI to 2.5: Was there potential for a substantial impact (on the estimated effect of intervention) of analysing participants in the wrong group?	NA	
	**Risk of bias judgement**	**Low**	Double-blind design and ITT analysis employed in this trial put low risk of bias in deviation from intended interventions.
Bias due to missing outcome data	3.1 Were outcome data available for all, or nearly all, participants randomized?	N	High proportion of drop-out: 17/41 and 14/42, including 5 and 3 declined to participate
	3.2 If N/PN/NI to 3.1: Are the proportions of missing outcome data and reasons for missing outcome data similar across intervention groups?	N	Overall, drop-out occurred equally within the modafinil... ...and the placebo group...' Contrary to numbers in figure 2
	3.3 If N/PN/NI to 3.1: Is there evidence that results were robust to the presence of missing outcome data?	NI	Did not mention how to handle missing data.
	**Risk of bias judgement**	**High**	No information how to handle missing data and contradictory in reported drop-outs in Figure 2 and contexts put this domain as "High" risk of bias.
Bias in measurement of the outcome	4.1 Were outcome assessors aware of the intervention received by study participants?	N	double-blind
	4.2 If Y/PY/NI to 4.1: Was the assessment of the outcome likely to be influenced by knowledge of intervention received?	NA	
	**Risk of bias judgement**	**Low**	Double-blind design puts this domain as low risk of bias.
Bias in selection of the reported result	Are the reported outcome data likely to have been selected, on the basis of the results, from…		
	5.1. ... multiple outcome measurements (e.g. scales, definitions, time points) within the outcome domain?	PY	
	5.2 ... multiple analyses of the data?	N	
	**Risk of bias judgement**	**Some concerns**	Absence rate was not pre-specified in the methods section and not all results reported (follow-up 1), leading "some concerns" risk of bias in this domain.
**Overall bias**	**Risk of bias judgement**	**High**	High risk of bias overall due to imcompleted outcome reporting (missing follow-up time point 1)

**Reference**	Kampman 2007	**Aim**	assignment to intervention (the 'intention-to-treat' effect)	**Source**	Journal article(s) with results of the trial
**Outcome**	Abstinence (12 weeks)	**Results**	2/32 (Placebo) vs 9/29 (Quetiapine 400 mg)		

**Domain**	**Signalling question**	**Response**	**Comments**
Bias arising from the randomization process	1.1 Was the allocation sequence random?	NI	Only stated "random"
	1.2 Was the allocation sequence concealed until participants were recruited and assigned to interventions?	NI	
	1.3 Were there baseline imbalances that suggest a problem with the randomization process?	PN	29 q vs 32 p (combined); some evidence of imbalances among participant characteristics, which might be by chance
	**Risk of bias judgement**	**Some concerns**	No details were given regarding randomisation process, contributed to "some concerns" in this domain.
Bias due to deviations from intended interventions	2.1 Were participants aware of their assigned intervention during the trial?	PN	"double-blind"
	2.2 Were carers and trial personnel aware of participants' assigned intervention during the trial?	PN	
	2.3 If Y/PY/NI to 2.1 or 2.2: Were there deviations from the intended intervention beyond what would be expected in usual practice?	NA	
	2.4 If Y/PY to 2.3: Were these deviations from intended intervention unbalanced between groups and likely to have affected the outcome?	NA	
	2.5 If N/PN/NI to 2.4: Were these deviations likely to have affected the outcome?	N	No evidence that patients recieved a treatment other than the one they were assigned to.
	2.6 If Y/PY/NI to 2.5: Was there potential for a substantial impact (on the estimated effect of intervention) of analysing participants in the wrong group?	NA	
	**Risk of bias judgement**	**Low**	Double-blind design employed in this trial put low risk of bias in deviation from intended interventions.
Bias due to missing outcome data	3.1 Were outcome data available for all, or nearly all, participants randomized?	PN	There were 6/29 q and 8/32 p missing data in both groups and reasons were not given.
	3.2 If N/PN/NI to 3.1: Are the proportions of missing outcome data and reasons for missing outcome data similar across intervention groups?	PY	"there were no significant differences between medication and placebo groups in treatment completion (23/29, 77% for the quetiapine group; and 24/32, 75% for the placebo group)"
	3.3 If N/PN/NI to 3.1: Is there evidence that results were robust to the presence of missing outcome data?	NI	No information on how missing data handled, and no sensitivity analysis completed
	**Risk of bias judgement**	**Low**	Although there were some missing data, results still stood in consideration of missing data. "Low" risk of bias in this domain was rated.
Bias in measurement of the outcome	4.1 Were outcome assessors aware of the intervention received by study participants?	PN	"double-blind"; self-report
	4.2 If Y/PY/NI to 4.1: Was the assessment of the outcome likely to be influenced by knowledge of intervention received?	NA	
	**Risk of bias judgement**	**Low**	Double-blind design and self-reporting outcome (abstinence) put this domain as low risk of bias.
Bias in selection of the reported result	Are the reported outcome data likely to have been selected, on the basis of the results, from…		
	5.1. ... multiple outcome measurements (e.g. scales, definitions, time points) within the outcome domain?	PN	
	5.2 ... multiple analyses of the data?	PN	
	**Risk of bias judgement**	**Low**	No protocol was found but nature of outcome, abstinence, presents low risk of selected reported results and the authors described the method section clearly. Thus, we rated "Low" risk of bias in this domain.
**Overall bias**	**Risk of bias judgement**	**Some concerns**	Lack of detailed methods for the randomisation process contributed to "some concerns" in overall bias for this trial.

**Reference**	Kiefer 2003	**Aim**	assignment to intervention (the 'intention-to-treat' effect)	**Source**	Journal article(s) with results of the trial
**Outcome**	Abstinence (12 weeks)	**Results**	26/40 (ACP 1998mg + NTX 50 mg) vs 17/40 (Acamprosate 1998 mg) vs 22/40 (Naltrexone 50 mg) vs 10/40 (Placebo)		

**Domain**	**Signalling question**	**Response**	**Comments**

**Bias arising from the randomization process**	1.1 Was the allocation sequence random?	Y	"Allocation codes were provided in sealed envelopes for each patient at the pharmacy of the University Hospital of Hamburg, where formulation and blinding was conducted. The randomization was organized by a computer-generated list (M.B.)."
	1.2 Was the allocation sequence concealed until participants were recruited and assigned to interventions?	Y	
	1.3 Were there baseline imbalances that suggest a problem with the randomization process?	N	Each group has a size of 40 and no significant difference among characteristics of participants.
	**Risk of bias judgement**	**Low**	This study employed adequate randomisation methods in the trial and represents low risk of bias in the randomisation process.
**Bias due to deviations from intended interventions**	2.1 Were participants aware of their assigned intervention during the trial?	N	"Double-blind"<br>"Medication was given in a double-dummy design."
	2.2 Were carers and trial personnel aware of participants' assigned intervention during the trial?	PN	
	2.3 If Y/PY/NI to 2.1 or 2.2: Were there deviations from the intended intervention beyond what would be expected in usual practice?	NA	
	2.4 If Y/PY to 2.3: Were these deviations from intended intervention unbalanced between groups and likely to have affected the outcome?	NA	
	2.5 If N/PN/NI to 2.4: Were these deviations likely to have affected the outcome?	N	ITT analysis
	2.6 If Y/PY/NI to 2.5: Was there potential for a substantial impact (on the estimated effect of intervention) of analysing participants in the wrong group?	NA	
	**Risk of bias judgement**	**Low**	Double-blind design and ITT analysis employed in this trial put low risk of bias in deviation from intended interventions.
**Bias due to missing outcome data**	3.1 Were outcome data available for all, or nearly all, participants randomized?	N	10% dropped out; large % relapsed (fig 1 e.g. 12/40 for naltrexone, 17/40 for acamprosate) and so their assessments were discontinued.
	3.2 If N/PN/NI to 3.1: Are the proportions of missing outcome data and reasons for missing outcome data similar across intervention groups?	PN	NX: 12/40 relapse = 30% ; acam 17/40 relapse 43%; N+A 9/40 relapsed 23% placebo 75% relapse<br>adverse effects similar across tx groups but not placebo arm.
	3.3 If N/PN/NI to 3.1: Is there evidence that results were robust to the presence of missing outcome data?	PY	The authors used "multivariate analyses of covariance (MANCIVAs) with the time to the various events and the clumulative abstinence as dependent variables..."
	**Risk of bias judgement**	**Low**	Although some missing data in this trial, the authors applied multivariate analyses of covariance and survival analyses on the results. So we rated "Low" risk of bias.
**Bias in measurement of the outcome**	4.1 Were outcome assessors aware of the intervention received by study participants?	N	Self-report and confirmed by laboratory results.<br>"At each assessment, the patient was classified by the therapist as abstinent or relapsed according to his or her self-report."<br>"Drinking diary, laboratory measures, and interviews of collaterals were compared for consistency and were used to justify abstinence, lapses, and relapses (D.N., K.W.)."
	4.2 If Y/PY/NI to 4.1: Was the assessment of the outcome likely to be influenced by knowledge of intervention received?	NA	
	**Risk of bias judgement**	**Low**	Double-blind design and self-reporting outcome (confirmed by relatives and biochemistry results) put this domain as low risk of bias.
**Bias in selection of the reported result**	Are the reported outcome data likely to have been selected, on the basis of the results, from…		
	5.1. ... multiple outcome measurements (e.g. scales, definitions, time points) within the outcome domain?	PN	
	5.2 ... multiple analyses of the data?	PN	
	**Risk of bias judgement**	**Low**	No protocol was found but nature of outcome, abstinence, presents low risk of selected reported results and the authors described the method section clearly. Thus, we rated "Low" risk of bias in this domain.
**Overall bias**	**Risk of bias judgement**	**Low**	Low risk of bias in all domains resulting low risk of bias overall.

**Reference**	Ladewig 1993	**Aim**	assignment to intervention (the 'intention-to-treat' effect)	**Source**	Journal article(s) with results of the trial
**Outcome**	Abstinence (12 months)	**Results**	8/29 (Acamprosate) vs 4/32 (Placebo)		

Domain	Signalling question	Response	Comments
**Bias arising from the randomization process**	1.1 Was the allocation sequence random?	NI	Only stated "random"
	1.2 Was the allocation sequence concealed until participants were recruited and assigned to interventions?	NI	
	1.3 Were there baseline imbalances that suggest a problem with the randomization process?	NI	No baseline characteristics of participants was given by groups.
	**Risk of bias judgement**	**Some concerns**	No details were given regarding randomisation process, contributed to "some concerns" in this domain.
**Bias due to deviations from intended interventions**	2.1 Were participants aware of their assigned intervention during the trial?	PN	"double-blind"
	2.2 Were carers and trial personnel aware of participants' assigned intervention during the trial?	PN	
	2.3 If Y/PY/NI to 2.1 or 2.2: Were there deviations from the intended intervention beyond what would be expected in usual practice?	NA	
	2.4 If Y/PY to 2.3: Were these deviations from intended intervention unbalanced between groups and likely to have affected the outcome?	NA	
	2.5 If N/PN/NI to 2.4: Were these deviations likely to have affected the outcome?	N	Analysis was intention to treat.
	2.6 If Y/PY/NI to 2.5: Was there potential for a substantial impact (on the estimated effect of intervention) of analysing participants in the wrong group?	NA	
	**Risk of bias judgement**	**Low**	Double-blind design and ITT analysis employed in this trial put low risk of bias in deviation from intended interventions.
**Bias due to missing outcome data**	3.1 Were outcome data available for all, or nearly all, participants randomized?	PY	The authors followed all participants and reported their outcomes (not by group)
	3.2 If N/PN/NI to 3.1: Are the proportions of missing outcome data and reasons for missing outcome data similar across intervention groups?	NA	
	3.3 If N/PN/NI to 3.1: Is there evidence that results were robust to the presence of missing outcome data?	NA	
	**Risk of bias judgement**	**Low**	Although there were some missing data, the authors followed all participants and reported their abstinence status during the trial. Therefore, we rated "Low" risk of bias in this domain.
**Bias in measurement of the outcome**	4.1 Were outcome assessors aware of the intervention received by study participants?	N	"Double-blind"
	4.2 If Y/PY/NI to 4.1: Was the assessment of the outcome likely to be influenced by knowledge of intervention received?	NA	
	**Risk of bias judgement**	**Low**	Double-blind design and self-reporting outcome put this domain as low risk of bias.
**Bias in selection of the reported result**	Are the reported outcome data likely to have been selected, on the basis of the results, from…		
	5.1. ... multiple outcome measurements (e.g. scales, definitions, time points) within the outcome domain?	NI	
	5.2 ... multiple analyses of the data?	NI	
	**Risk of bias judgement**	**Low**	No protocol was found but nature of outcome, abstinence, presents low risk of selected reported results and the authors described the method section clearly. Thus, we rated "Low" risk of bias in this domain.

Overall bias	Risk of bias judgement	Some concerns	Lack of detailed methods for the randomisation process contributed to "some concerns" in overall bias for this trial.

Reference	Landabaso 1999	Aim	assignment to intervention (the 'intention-to-treat' effect)	Source	Journal article(s) with results of the trial
Outcome	Abstinence (12 months)	Results	3/15 (TAU) vs 11/15 (Naltrexone 25 mg)		

Domain	Signalling question	Response	Comments
Bias arising from the randomization process	1.1 Was the allocation sequence random?	NI	Only stated "random"
	1.2 Was the allocation sequence concealed until participants were recruited and assigned to interventions?	NI	
	1.3 Were there baseline imbalances that suggest a problem with the randomization process?	PN	Same size between two groups and no significant difference in characteristic between groups.
	**Risk of bias judgement**	**Some concerns**	No details were given regarding randomisation process, contributed to "some concerns" in this domain.
Bias due to deviations from intended interventions	2.1 Were participants aware of their assigned intervention during the trial?	NI	Did not address blinding
	2.2 Were carers and trial personnel aware of participants' assigned intervention during the trial?	NI	
	2.3 If Y/PY/NI to 2.1 or 2.2: Were there deviations from the intended intervention beyond what would be expected in usual practice?	NI	No information on deviations from intended interventions.
	2.4 If Y/PY to 2.3: Were these deviations from intended intervention unbalanced between groups and likely to have affected the outcome?	NA	
	2.5 If N/PN/NI to 2.4: Were these deviations likely to have affected the outcome?	PN	No evidence of patients being analysed in the incorrect group.
	2.6 If Y/PY/NI to 2.5: Was there potential for a substantial impact (on the estimated effect of intervention) of analysing participants in the wrong group?	NA	
	**Risk of bias judgement**	**Some concerns**	There was no complete information regarding blinding, contributing to "some concerns" in this domain.
Bias due to missing outcome data	3.1 Were outcome data available for all, or nearly all, participants randomized?	PY	Very small number of missing data (Table 2) and contexts
	3.2 If N/PN/NI to 3.1: Are the proportions of missing outcome data and reasons for missing outcome data similar across intervention groups?	NA	
	3.3 If N/PN/NI to 3.1: Is there evidence that results were robust to the presence of missing outcome data?	NA	
	**Risk of bias judgement**	**Low**	Almost all outcome data were available, thus this domain was rated "Low" risk of bias.
Bias in measurement of the outcome	4.1 Were outcome assessors aware of the intervention received by study participants?	NI	Didn't address the method of assessing abstinence but it seemed to be self-report.
	4.2 If Y/PY/NI to 4.1: Was the assessment of the outcome likely to be influenced by knowledge of intervention received?	PY	Potential for assessor bias in prompting of patients to accurately or not recall the number of drinks taken
	**Risk of bias judgement**	**High**	No texts mentioned blinding outcome assessor (patient themselves) and potential bias, contributing to "High" risk of bias.
Bias in selection of the reported result	Are the reported outcome data likely to have been selected, on the basis of the results, from…		
	5.1. ... multiple outcome measurements (e.g. scales, definitions, time points) within the outcome domain?	NI	No detailed of evluation.
	5.2 ... multiple analyses of the data?	NI	
	**Risk of bias judgement**	**Some concerns**	No protocol was found and the authors did not describe the method for abstinence clearly. Thus, we rated "Some concerns" risk of bias in this domain.
Overall bias	**Risk of bias judgement**	**High**	High risk of bias due to unclear methods in blinding and methods for assessing outcomes.

Reference	Mann 2006	Aim	assignment to intervention (the 'intention-to-treat' effect)	Source	Journal article(s) with results of the trial
Outcome	Abstinence (24 weeks)	Results	9/74 (Galantamine 25 mg) vs 23/75 (Placebo)		

Domain	Signalling question	Response	Comments
Bias arising from the randomization process	1.1 Was the allocation sequence random?	NI	Only stated "randomized"
	1.2 Was the allocation sequence concealed until participants were recruited and assigned to interventions?	NI	
	1.3 Were there baseline imbalances that suggest a problem with the randomization process?	PN	"With regard to sociodemographic and prestudy data, both GAL and placebo groups were homogenous at baseline (Table 1)."
	**Risk of bias judgement**	**Some concerns**	No details were given regarding randomisation process, contributed to "some concerns" in this domain.
Bias due to deviations from intended interventions	2.1 Were participants aware of their assigned intervention during the trial?	PN	double-blind
	2.2 Were carers and trial personnel aware of participants' assigned intervention during the trial?	PN	
	2.3 If Y/PY/NI to 2.1 or 2.2: Were there deviations from the intended intervention beyond what would be expected in usual practice?	NA	
	2.4 If Y/PY to 2.3: Were these deviations from intended intervention unbalanced between groups and likely to have affected the outcome?	NA	
	2.5 If N/PN/NI to 2.4: Were these deviations likely to have affected the outcome?	N	No mention of patients not recieving their assigned intervention.
	2.6 If Y/PY/NI to 2.5: Was there potential for a substantial impact (on the estimated effect of intervention) of analysing participants in the wrong group?	NA	
	**Risk of bias judgement**	**Low**	Double-blind design and ITT analysis employed in this trial put low risk of bias in deviation from intended intervention
Bias due to missing outcome data	3.1 Were outcome data available for all, or nearly all, participants randomized?	N	Only 64/149 patients completed the study.
	3.2 If N/PN/NI to 3.1: Are the proportions of missing outcome data and reasons for missing outcome data similar across intervention groups?	NI	The authors did not publish the details of drop-outs.
	3.3 If N/PN/NI to 3.1: Is there evidence that results were robust to the presence of missing outcome data?	PN	"If appropriate, we calculated last-observation-carried-forward (LOCF) analyses. A two-tailed P value less than 0.05 was considered to be significant.Missing data were not replaced."<br>"Survival analyses carried out for each trial center yielded no between-center differences."
	**Risk of bias judgement**	**Some concerns**	High proportion of drop-outs and lack of details of drop-outs put this domain as "some concerns".

**Bias in measurement of the outcome**	4.1 Were outcome assessors aware of the intervention received by study participants?	N	By patient's diaries and double-blind
	4.2 If Y/PY/NI to 4.1: Was the assessment of the outcome likely to be influenced by knowledge of intervention received?	NA	
	**Risk of bias judgement**	**Low**	Double-blind design and self-reporting outcome (confirmed by relatives and biochemistry results) put this domain as low risk of bias
**Bias in selection of the reported result**	Are the reported outcome data likely to have been selected, on the basis of the results, from…		
	5.1. ... multiple outcome measurements (e.g. scales, definitions, time points) within the outcome domain?	PN	
	5.2 ... multiple analyses of the data?	PN	
	**Risk of bias judgement**	**Low**	No protocol was found but nature of outcome, abstinence, presents low risk of selected reported results and the authors described the method section clearly. Thus, we rated "Low" risk of bias in this domain.
**Overall bias**	**Risk of bias judgement**	**Some concerns**	Lack of detailed methods for the randomization process and high proportion of missing data without details, together, these contributed to "some concerns" in overall bias for this trial.

**Reference**	Marra 2002	**Aim**	assignment to intervention (the 'intention-to-treat' effect)	**Source**	Journal article(s) with results of the trial
**Outcome**	Abstinence (12 months)	**Results**	4/37 (Amisulpride 50 mg) vs 8/34 (Placebo)		

**Domain**	**Signalling question**	**Response**	**Comments**
**Bias arising from the randomization process**	1.1 Was the allocation sequence random?	NI	only stated "random"
	1.2 Was the allocation sequence concealed until participants were recruited and assigned to interventions?	NI	
	1.3 Were there baseline imbalances that suggest a problem with the randomization process?	PN	Except VAS results, there were not significant difference between groups.
	**Risk of bias judgement**	**Some concerns**	No details were given regarding randomisation process, contributed to "some concerns" in this domain.
**Bias due to deviations from intended interventions**	2.1 Were participants aware of their assigned intervention during the trial?	PN	Double-blind
	2.2 Were carers and trial personnel aware of participants' assigned intervention during the trial?	PN	
	2.3 If Y/PY/NI to 2.1 or 2.2: Were there deviations from the intended intervention beyond what would be expected in usual practice?	NA	
	2.4 If Y/PY to 2.3: Were these deviations from intended intervention unbalanced between groups and likely to have affected the outcome?	NA	
	2.5 If N/PN/NI to 2.4: Were these deviations likely to have affected the outcome?	N	"Data of the intention-to-treat population are presented. The intention-to-treat population included all patients." Noe evidence that patient were analysed in the wrong group
	2.6 If Y/PY/NI to 2.5: Was there potential for a substantial impact (on the estimated effect of intervention) of analysing participants in the wrong group?	NA	
	**Risk of bias judgement**	**Low**	Double-blind design and ITT analysis employed in this trial put low risk of bias in deviation from intended interventions.
**Bias due to missing outcome data**	3.1 Were outcome data available for all, or nearly all, participants randomized?	N	There were high proportion of drop-outs (50% in placebo and 62.2% in AMI group)
	3.2 If N/PN/NI to 3.1: Are the proportions of missing outcome data and reasons for missing outcome data similar across intervention groups?	PN	Numbers comparable but some difference in reasons between treatment arms. E.g number of drop-outs due to not severe adverse events
	3.3 If N/PN/NI to 3.1: Is there evidence that results were robust to the presence of missing outcome data?	PN	No sensitivity analysis presented.<br>"Those who were drinking when they dropped out were considered to have been drinking from the time of dropout until the end of the 6-month treatment period."
	**Risk of bias judgement**	**High**	High porportion of missing data and imbalanced missing data without sensitivity analysis put "high" risk of bias in this domain.
**Bias in measurement of the outcome**	4.1 Were outcome assessors aware of the intervention received by study participants?	N	self-reported; double-blind
	4.2 If Y/PY/NI to 4.1: Was the assessment of the outcome likely to be influenced by knowledge of intervention received?	NA	
	**Risk of bias judgement**	**Low**	Double-blind design and self-reporting put this domain as low risk of bias.
**Bias in selection of the reported result**	Are the reported outcome data likely to have been selected, on the basis of the results, from…		
	5.1. ... multiple outcome measurements (e.g. scales, definitions, time points) within the outcome domain?	N	
	5.2 ... multiple analyses of the data?	N	
	**Risk of bias judgement**	**Low**	No protocol was found but nature of outcome, abstinence, presents low risk of selected reported results and the authors described the method section clearly. Thus, we rated "Low" risk of bias in this domain.
**Overall bias**	**Risk of bias judgement**	**High**	Lack of detailed methods for the randomisation process and missing data, together, these contributed to "High" in overall bias for this trial.

**Reference**	Martinotti 2009	**Aim**	assignment to intervention (the 'intention-to-treat' effect)	**Source**	Journal article(s) with results of the trial
**Outcome**	Abstinence (16 weeks)	**Results**	12/29 (Aripiprazole 15 mg) vs 11/28 (Naltrexone 50 mg)		

**Domain**	**Signalling question**	**Response**	**Comments**
**Bias arising from the randomization process**	1.1 Was the allocation sequence random?	Y	"Random assignment was achieved in a non-centre-specific manner with an interactive voice-response central randomisation service."
	1.2 Was the allocation sequence concealed until participants were recruited and assigned to interventions?	PY	
	1.3 Were there baseline imbalances that suggest a problem with the randomization process?	PN	No detailed characteristic of participants but the authors stated that there were no significant differences betweene the baseline characteristics of patients.
	**Risk of bias judgement**	**Low**	This study employed adequate randomisation methods in the trial and represents "Low" risk of bias in the randomisation process.
**Bias due to deviations from intended interventions**	2.1 Were participants aware of their assigned intervention during the trial?	N	double-blind; identical placebo
	2.2 Were carers and trial personnel aware of participants' assigned intervention during the trial?	PN	
	2.3 If Y/PY/NI to 2.1 or 2.2: Were there deviations from the intended intervention beyond what would be expected in usual practice?	NA	
	2.4 If Y/PY to 2.3: Were these deviations from intended intervention unbalanced between groups and likely to have affected the outcome?	NA	

interventions	2.5 If N/PN/NI to 2.4: Were these deviations likely to have affected the outcome?	N	"Primary and secondary efficacy analyses were performed on the intent-to-treat population, which included all randomly assigned patients who took at least one dose of study medication."
	2.6 If Y/PY/NI to 2.5: Was there potential for a substantial impact (on the estimated effect of intervention) of analysing participants in the wrong group?	NA	
	**Risk of bias judgement**	**Low**	Double-blind design and ITT analysis employed in this trial put "Low" risk of bias in deviation from intended interventions.
**Bias due to missing outcome data**	3.1 Were outcome data available for all, or nearly all, participants randomized?	PN	Completion: 22/29 in ARI group and 21/28 in NAL group
	3.2 If N/PN/NI to 3.1: Are the proportions of missing outcome data and reasons for missing outcome data similar across intervention groups?	PN	There were higher proportion of discontinuation due to adverse events in NAL group (17.8%), compared with (6.9%) the ARI group.
	3.3 If N/PN/NI to 3.1: Is there evidence that results were robust to the presence of missing outcome data?	PN	
	**Risk of bias judgement**	**Some concerns**	High porportion of missing data and no detailed reasons for missing data put "some concerns" in this domain.
**Bias in measurement of the outcome**	4.1 Were outcome assessors aware of the intervention received by study participants?	N	double-blind; self-evluation and family member interview
	4.2 If Y/PY/NI to 4.1: Was the assessment of the outcome likely to be influenced by knowledge of intervention received?	NA	
	**Risk of bias judgement**	**Low**	Double-blind design and self-reporting outcome (confirmed by relatives) put this domain as low risk of bias.
**Bias in selection of the reported result**	Are the reported outcome data likely to have been selected, on the basis of the results, from…		
	5.1. ... multiple outcome measurements (e.g. scales, definitions, time points) within the outcome domain?	PN	
	5.2 ... multiple analyses of the data?	PN	
	**Risk of bias judgement**	**Low**	No protocol was found but nature of outcome, abstinence, presents low risk of selected reported results and the authors described the method section clearly. Thus, we rated "Low" risk of bias in this domain.
**Overall bias**	**Risk of bias judgement**	**Some concerns**	Some concerns overall due to missing data in this trial.

**Reference**	Martinotti 2007	**Aim**	assignment to intervention (the 'intention-to-treat' effect)	**Source**	Journal article(s) with results of the trial
**Outcome**	Abstinence (90 days)	**Results**	11/27 (Naltrexone 50 mg) vs 29/57 (Combined: Oxcarbazepine High+Low doses)		

**Domain**	**Signalling question**	**Response**	**Comments**
**Bias arising from the randomization process**	1.1 Was the allocation sequence random?	Y	"Random assignment was achieved in a non-centre-specific manner with an interactive voice-response central randomisation service."
	1.2 Was the allocation sequence concealed until participants were recruited and assigned to interventions?	PY	
	1.3 Were there baseline imbalances that suggest a problem with the randomization process?	PN	There was no significant difference between groups except OCDS between groups, which might result from chance.
	**Risk of bias judgement**	**Low**	This study employed adequate randomisation methods in the trial and represents low risk of bias in the randomisation process.
**Bias due to deviations from intended interventions**	2.1 Were participants aware of their assigned intervention during the trial?	Y	open-trial
	2.2 Were carers and trial personnel aware of participants' assigned intervention during the trial?	Y	
	2.3 If Y/PY/NI to 2.1 or 2.2: Were there deviations from the intended intervention beyond what would be expected in usual practice?	NI	
	2.4 If Y/PY to 2.3: Were these deviations from intended intervention unbalanced between groups and likely to have affected the outcome?	NA	
	2.5 If N/PN/NI to 2.4: Were these deviations likely to have affected the outcome?	N	No evidence of switching. The authors seemed to use ITT analysis.
	2.6 If Y/PY/NI to 2.5: Was there potential for a substantial impact (on the estimated effect of intervention) of analysing participants in the wrong group?	NA	
	**Risk of bias judgement**	**Some concerns**	The open label design in this trial might prompt deviations from the intended interventions, contributing to "some concerns" in this domain.
**Bias due to missing outcome data**	3.1 Were outcome data available for all, or nearly all, participants randomized?	N	"...patients who completed the study... 93.1% ...77.7%... ...75%..."
	3.2 If N/PN/NI to 3.1: Are the proportions of missing outcome data and reasons for missing outcome data similar across intervention groups?	N	There were higher proportion of droup-outs in low OXC and NAL groups.
	3.3 If N/PN/NI to 3.1: Is there evidence that results were robust to the presence of missing outcome data?	N	No sensitivity analysis and the authors did not mention the methods for dealing drop-outs.
	**Risk of bias judgement**	**High**	High porportion of missing data and imbalanced missing data without sensitivity analysis put "high" risk of bias in this domain.
**Bias in measurement of the outcome**	4.1 Were outcome assessors aware of the intervention received by study participants?	Y	Self-evluation; family interview
	4.2 If Y/PY/NI to 4.1: Was the assessment of the outcome likely to be influenced by knowledge of intervention received?	PN	Abstinence was confirmed by family members
	**Risk of bias judgement**	**Low**	Although this is an open study, the outcome (abstinence) was confirmed by a family member, which put low risk of bias in this domain.
**Bias in selection of the reported result**	Are the reported outcome data likely to have been selected, on the basis of the results, from…		
	5.1. ... multiple outcome measurements (e.g. scales, definitions, time points) within the outcome domain?	PN	
	5.2 ... multiple analyses of the data?	PN	
	**Risk of bias judgement**	**Low**	No protocol was found but nature of outcome, abstinence, presents low risk of selected reported results and the authors described the method section clearly. Thus, we rated "Low" risk of bias in this domain.
**Overall bias**	**Risk of bias judgement**	**High**	Open-label design and missing data put this trial "High" risk of bias overall

**Reference**	Martinotti 2010	**Aim**	assignment to intervention (the 'intention-to-treat' effect)	**Source**	Journal article(s) with results of the trial
**Outcome**	Abstinence (16 weeks)	**Results**	11/28 (Naltrexone 50 mg) vs 15/31 (Pregablin 450 mg)		

**Domain**	**Signalling question**	**Response**	**Comments**
**Bias arising from the randomization**	1.1 Was the allocation sequence random?	Y	"...randomisation was performed using a common computer-generated system."
	1.2 Was the allocation sequence concealed until participants were recruited and assigned to interventions?	PY	"All study personnel in contact with the participants wre unaware of the randomisation sequence"

the randomization process	1.3 Were there baseline imbalances that suggest a problem with the randomization process?	PN	No characteristic of participants but the authors stated that no significant differences between two groups.
	**Risk of bias judgement**	**Low**	This study employed adequate randomisation methods in the trial and represents low risk of bias in the randomisation process.
**Bias due to deviations from intended interventions**	2.1 Were participants aware of their assigned intervention during the trial?	N	Double-blind<br>"tablets were identical in appearance and ..."
	2.2 Were carers and trial personnel aware of participants' assigned intervention during the trial?	PN	
	2.3 If Y/PY/NI to 2.1 or 2.2: Were there deviations from the intended intervention beyond what would be expected in usual practice?	NA	
	2.4 If Y/PY to 2.3: Were these deviations from intended intervention unbalanced between groups and likely to have affected the outcome?	NA	
	2.5 If N/PN/NI to 2.4: Were these deviations likely to have affected the outcome?	N	No evidence of switching interventions
	2.6 If Y/PY/NI to 2.5: Was there potential for a substantial impact (on the estimated effect of intervention) of analysing participants in the wrong group?	NA	
	**Risk of bias judgement**	**Low**	Double-blind design and no evidence of switching interventions in the analysis in this trial put low risk of bias in deviation from intended interventions.
**Bias due to missing outcome data**	3.1 Were outcome data available for all, or nearly all, participants randomized?	N	Drop-out: 4/31 (NAL) and 7/28 (PGB)
	3.2 If N/PN/NI to 3.1: Are the proportions of missing outcome data and reasons for missing outcome data similar across intervention groups?	NI	"The overall rate of study discontinuation due to adverse event was 3.2% in the PRE group and 17.8% in the NAL group."
	3.3 If N/PN/NI to 3.1: Is there evidence that results were robust to the presence of missing outcome data?	N	
	**Risk of bias judgement**	**Some concerns**	High porportion of missing data and no detailed reasons for missing data put "some concerns" in this domain.
**Bias in measurement of the outcome**	4.1 Were outcome assessors aware of the intervention received by study participants?	N	double-blind; abstinence was evaluated by self-evaluation and family member interview.
	4.2 If Y/PY/NI to 4.1: Was the assessment of the outcome likely to be influenced by knowledge of intervention received?	NA	
	**Risk of bias judgement**	**Low**	Double-blind design and self-reporting outcome (confirmed by relatives) put this domain as low risk of bias.
**Bias in selection of the reported result**	Are the reported outcome data likely to have been selected, on the basis of the results, from…		
	5.1. ... multiple outcome measurements (e.g. scales, definitions, time points) within the outcome domain?	PN	
	5.2 ... multiple analyses of the data?	PN	
	**Risk of bias judgement**	**Low**	No protocol was found but nature of outcome, abstinence, presents low risk of selected reported results and the authors described the method section clearly. Thus, we rated "Low" risk of bias in this domain.
**Overall bias**	**Risk of bias judgement**	**Some concerns**	Some concerns overall due to lack of details in missing data.

**Reference**	Moncini 2000	**Aim**	assignment to intervention (the 'intention-to-treat' effect)	**Source**	Journal article(s) with results of the trial
**Outcome**	Abstinence (6 months)	**Results**	6/9 (GHB 50 mg/kg) vs 4/8 (Placebo)		

Domain	Signalling question	Response	Comments
**Bias arising from the randomization process**	1.1 Was the allocation sequence random?	NI	Only stated "random"; "When discharged from the hospital, the patients were randomly divided into two groups, A and B"
	1.2 Was the allocation sequence concealed until participants were recruited and assigned to interventions?	PY	"Might indicate a sign of sequence concealment: "They were randomly divided into two groups (group A and group B) and, when the code was opened, group A proved to have been treated with GHB (mean daily oral dose 50 mg/kg) and group B with placebo."<br>"when the code was opeened, A provided to have been treated with GHB, B with placebo" suggests allocation concealment but no details
	1.3 Were there baseline imbalances that suggest a problem with the randomization process?	NI	Did not provide characteristics of participants
	**Risk of bias judgement**	**Low**	No details were given regarding randomisation generation but some indications regarding allocation sequence concealment - "Low" risk of bias
**Bias due to deviations from intended interventions**	2.1 Were participants aware of their assigned intervention during the trial?	PN	stated "double-blind study"
	2.2 Were carers and trial personnel aware of participants' assigned intervention during the trial?	PN	
	2.3 If Y/PY/NI to 2.1 or 2.2: Were there deviations from the intended intervention beyond what would be expected in usual practice?	NA	
	2.4 If Y/PY to 2.3: Were these deviations from intended intervention unbalanced between groups and likely to have affected the outcome?	NA	
	2.5 If N/PN/NI to 2.4: Were these deviations likely to have affected the outcome?	N	No evidence of switching participants
	2.6 If Y/PY/NI to 2.5: Was there potential for a substantial impact (on the estimated effect of intervention) of analysing participants in the wrong group?	NA	
	**Risk of bias judgement**	**Low**	Double-blind design put "Low" risk of bias in deviation from intended interventions.
**Bias due to missing outcome data**	3.1 Were outcome data available for all, or nearly all, participants randomized?	PN	Table 3; the drop-out rates were low (2/9, group A and 2/8, group B) but did not indicate the reasons.
	3.2 If N/PN/NI to 3.1: Are the proportions of missing outcome data and reasons for missing outcome data similar across intervention groups?	NI	The proportions of missing data were similar but no information regarding their reasons
	3.3 If N/PN/NI to 3.1: Is there evidence that results were robust to the presence of missing outcome data?	PN	Small study
	**Risk of bias judgement**	**Some concerns**	There were some missing data. Although the numbers of missing data were similar, no details and small study effects put "some concerns" in this domain.
**Bias in measurement of the outcome**	4.1 Were outcome assessors aware of the intervention received by study participants?	PN	Double-blind
	4.2 If Y/PY/NI to 4.1: Was the assessment of the outcome likely to be influenced by knowledge of intervention received?	NA	
	**Risk of bias judgement**	**Low**	Double-blind design put this domain as low risk of bias.
**Bias in selection of the reported result**	Are the reported outcome data likely to have been selected, on the basis of the results, from…		
	5.1. ... multiple outcome measurements (e.g. scales, definitions, time points) within the outcome domain?	PN	
	5.2 ... multiple analyses of the data?	PN	
	**Risk of bias judgement**	**Low**	No protocol was found but nature of outcome, abstinence, presents low risk of selected reported results. Thus, we rated "Low" risk of bias in this domain.
**Overall bias**	**Risk of bias judgement**	**Some concerns**	Some concerns with missing data due to no details of missing data and a small study.

Reference	Moraes 2010	Aim	assignment to intervention (the 'intention-to-treat' effect)	Source	Journal article(s) with results of the trial
**Outcome**	Abstinence (12 weeks)	**Results**	25/58 (TAU) vs 36/62 (Home visit)		

Domain	Signalling question	Response	Comments
Bias arising from the randomization process	1.1 Was the allocation sequence random?	Y	"...with the use of a table of random numbers." "To avoid selection bias, the randomization was carried out by a UNIAD employee not involved in the study, and who did not have access to any information regarding the research or the patients."
	1.2 Was the allocation sequence concealed until participants were recruited and assigned to interventions?	Y	
	1.3 Were there baseline imbalances that suggest a problem with the randomization process?	PN	"There was no difference between the HV treatment and CT in any of the variables analyzed in the beginning of the treatment (P>0.05), which guaranteed the homogeneity between the groups."
	**Risk of bias judgement**	**Low**	This study employed adequate randomisation methods in the trial and represents low risk of bias in the randomisation process.
Bias due to deviations from intended interventions	2.1 Were participants aware of their assigned intervention during the trial?	Y	It was impossible to blind participants due to nature of interventions.
	2.2 Were carers and trial personnel aware of participants' assigned intervention during the trial?	Y	
	2.3 If Y/PY/NI to 2.1 or 2.2: Were there deviations from the intended intervention beyond what would be expected in usual practice?	NI	None reported.
	2.4 If Y/PY to 2.3: Were these deviations from intended intervention unbalanced between groups and likely to have affected the outcome?	NA	
	2.5 If N/PN/NI to 2.4: Were these deviations likely to have affected the outcome?	PN	Appears that there were no incorrectly analysed patients -> no evidence of switches
	2.6 If Y/PY/NI to 2.5: Was there potential for a substantial impact (on the estimated effect of intervention) of analysing participants in the wrong group?	NA	
	**Risk of bias judgement**	**Some concerns**	The difference between interventions in this trial might prompt deviations from the intended interventions, contributing to "some concerns" in this domain.
Bias due to missing outcome data	3.1 Were outcome data available for all, or nearly all, participants randomized?	N	There were more participant lost to follow-up in CT group.
	3.2 If N/PN/NI to 3.1: Are the proportions of missing outcome data and reasons for missing outcome data similar across intervention groups?	N	22 lost (CT) vs 9 lost (HV)
	3.3 If N/PN/NI to 3.1: Is there evidence that results were robust to the presence of missing outcome data?	N	ITT analyses were used but huge imbalance in missing data between group so the reviewer rated as "no"
	**Risk of bias judgement**	**High**	High porportion of missing data and imbalanced missing data without sensitivity analysis put "high" risk of bias in this domain.
Bias in measurement of the outcome	4.1 Were outcome assessors aware of the intervention received by study participants?	PY	Self reported (presumed) alcohol consumption.
	4.2 If Y/PY/NI to 4.1: Was the assessment of the outcome likely to be influenced by knowledge of intervention received?	PY	Subjective outcome, and potential for recall bias based on treatment received.
	**Risk of bias judgement**	**High**	Lack of blinding to outcome assessors (participants themself) and self-reporting outcomes, which put "some concerns" in this domain.
Bias in selection of the reported result	Are the reported outcome data likely to have been selected, on the basis of the results, from…		
	5.1. ... multiple outcome measurements (e.g. scales, definitions, time points) within the outcome domain?	PN	
	5.2 ... multiple analyses of the data?	PN	
	**Risk of bias judgement**	**Low**	No protocol was found but nature of outcome, abstinence, presents low risk of selected reported results and the authors described the method section clearly. Thus, we rated "Low" risk of bias in this domain.
**Overall bias**	**Risk of bias judgement**	**High**	High risk of bias in the missing data and imbalanced missing data and outcome measurement contributed to "High" risk of bias in overall bias.

Reference	Mueller 1997	Aim	assignment to intervention (the 'intention-to-treat' effect)	Source	Journal article(s) with results of the trial
**Outcome**	Abstinence (12 months)	**Results**	2/13 (Carbamazepine 600 mg) vs 4/16 (Placebo)		

Domain	Signalling question	Response	Comments
Bias arising from the randomization process	1.1 Was the allocation sequence random?	NI	"Once meeting eligibility criteria and providing signed consent, subjects were randomized to either carbamazepine or placebo"
	1.2 Was the allocation sequence concealed until participants were recruited and assigned to interventions?	NI	No detailed info on randomisation or on allocation concealment
	1.3 Were there baseline imbalances that suggest a problem with the randomization process?	PN	High risk of bias in the measurement of the outcome and some concerns in deviations from the intended interventions contributed to "High" risk of bias in overall bias.
	**Risk of bias judgement**	**Some concerns**	No details were given regarding randomisation process, contributed to "some concerns" in this domain.
Bias due to deviations from intended interventions	2.1 Were participants aware of their assigned intervention during the trial?	PN	"double-blind"; "A medical physician who was not blind to the study protocol and who was never in contact with subjects, monitored levels during the treatment phase..."
	2.2 Were carers and trial personnel aware of participants' assigned intervention during the trial?	PN	
	2.3 If Y/PY/NI to 2.1 or 2.2: Were there deviations from the intended intervention beyond what would be expected in usual practice?	NA	
	2.4 If Y/PY to 2.3: Were these deviations from intended intervention unbalanced between groups and likely to have affected the outcome?	NA	
	2.5 If N/PN/NI to 2.4: Were these deviations likely to have affected the outcome?	N	"Analyses were conducted for all subjects based on treatment assignment regardless of whether or when they stopped taking the study drug (intent-to-treat analysis)."
	2.6 If Y/PY/NI to 2.5: Was there potential for a substantial impact (on the estimated effect of intervention) of analysing participants in the wrong group?	NA	
	**Risk of bias judgement**	**Low**	Double-blind design and ITT analysis employed in this trial put "Low" risk of bias in deviation from intended interventions.
Bias due to missing outcome data	3.1 Were outcome data available for all, or nearly all, participants randomized?	N	There were 8/16 and 8/13 missing data.
	3.2 If N/PN/NI to 3.1: Are the proportions of missing outcome data and reasons for missing outcome data similar across intervention groups?	PN	The number of missing data was similar between groups but there could be more participants dropped in crbamazepine group due to medication toxicity.
	3.3 If N/PN/NI to 3.1: Is there evidence that results were robust to the presence of missing outcome data?	N	No sensitivity analyses performed
	**Risk of bias judgement**	**High**	High porportion of missing data and imbalanced missing data without sensitivity analysis put "High" risk of bias in this domain.
Bias in measurement of the outcome	4.1 Were outcome assessors aware of the intervention received by study participants?	N	"double-blind"; self-report
	4.2 If Y/PY/NI to 4.1: Was the assessment of the outcome likely to be influenced by knowledge of intervention received?	NA	
	**Risk of bias judgement**	**Low**	Double-blind design put this domain as low risk of bias.

**Bias in selection of the reported result**	Are the reported outcome data likely to have been selected, on the basis of the results, from…		
	5.1. ... multiple outcome measurements (e.g. scales, definitions, time points) within the outcome domain?	PN	
	5.2 ... multiple analyses of the data?	PN	
	**Risk of bias judgement**	**Low**	No protocol was found but nature of outcome, abstinence, presents low risk of selected reported results and the authors described the method section clearly. Thus, we rated "Low" risk of bias in this domain.
**Overall bias**	**Risk of bias judgement**	**High**	High risk of bias in missing data contributed to "High" risk of bias overall.

**Reference**	BACLAD study	**Aim**	assignment to intervention (the 'intention-to-treat' effect)	**Source**	Journal article(s) with results of the trial
**Outcome**	Abstinence (24 weeks)	**Results**	10/28 (Baclofen 270 mg) vs 3/28 (Placebo)		

Domain	Signalling question	Response	Comments
**Bias arising from the randomization process**	1.1 Was the allocation sequence random?	Y	"...accoring to a computer-generated randomization list (in blocks of 4; stratfification with regard to sex)."<br>"The ranomization list was kept by the biometrician and the study pharmacist who prepared the study medication packages. The study pharmacist did not have any further role in the trial."<br>"Sealed envelopes containing study medication details were kept at the outpatient unit to be opened by a staff member in case of a study drug-related emergency...."
	1.2 Was the allocation sequence concealed until participants were recruited and assigned to interventions?	PY	
	1.3 Were there baseline imbalances that suggest a problem with the randomization process?	N	No significant difference between groups.
	**Risk of bias judgement**	**Low**	This study employed adequate randomisation methods in the trial and represents low risk of bias in the randomisation process.
**Bias due to deviations from intended interventions**	2.1 Were participants aware of their assigned intervention during the trial?	N	double-blind; identifical placebo
	2.2 Were carers and trial personnel aware of participants' assigned intervention during the trial?	PN	
	2.3 If Y/PY/NI to 2.1 or 2.2: Were there deviations from the intended intervention beyond what would be expected in usual practice?	NA	
	2.4 If Y/PY to 2.3: Were these deviations from intended intervention unbalanced between groups and likely to have affected the outcome?	NA	
	2.5 If N/PN/NI to 2.4: Were these deviations likely to have affected the outcome?	N	No evidence of switching
	2.6 If Y/PY/NI to 2.5: Was there potential for a substantial impact (on the estimated effect of intervention) of analysing participants in the wrong group?	NA	
	**Risk of bias judgement**	**Low**	Double-blind design employed in this trial put "Low" risk of bias in deviation from intended interventions.
**Bias due to missing outcome data**	3.1 Were outcome data available for all, or nearly all, participants randomized?	PN	Some drop-outs observed in Figure 2
	3.2 If N/PN/NI to 3.1: Are the proportions of missing outcome data and reasons for missing outcome data similar across intervention groups?	PY	The numbers of drop-outs were small in each category.
	3.3 If N/PN/NI to 3.1: Is there evidence that results were robust to the presence of missing outcome data?	PY	The results remained in considering of missing data.
	**Risk of bias judgement**	**Low**	Although there were some missing data, results still stood in consideration of missing data. "Low" risk of bias in this domain was rated.
**Bias in measurement of the outcome**	4.1 Were outcome assessors aware of the intervention received by study participants?	N	Abstinence was assessed by subjective report plus negative breathalyzer test as well as a level of carbohydrate-deficient transferrin (CDT) within the normal range, or, if increased, lower compared to the baseline level.
	4.2 If Y/PY/NI to 4.1: Was the assessment of the outcome likely to be influenced by knowledge of intervention received?	NA	
	**Risk of bias judgement**	**Low**	Double-blind design and self-reporting outcome (confirmed by biochemistry results) put this domain as low risk of bias.
**Bias in selection of the reported result**	Are the reported outcome data likely to have been selected, on the basis of the results, from…		
	5.1. ... multiple outcome measurements (e.g. scales, definitions, time points) within the outcome domain?	PN	
	5.2 ... multiple analyses of the data?	PN	
	**Risk of bias judgement**	**Low**	No protocol was found but nature of outcome, abstinence, presents low risk of selected reported results and the authors described the method section clearly. Thus, we rated "Low" risk of bias in this domain.
**Overall bias**	**Risk of bias judgement**	**Low**	Low risk of bias

**Reference**	Oslin 2005	**Aim**	assignment to intervention (the 'intention-to-treat' effect)	**Source**	Journal article(s) with results of the trial
**Outcome**	Abstinence (3 months)	**Results**	16/37 (Naltrexone 50 mg) vs 20/37 (Placebo)		

Domain	Signalling question	Response	Comments
**Bias arising from the randomization process**	1.1 Was the allocation sequence random?	Y	"Randomisation was stratifited by gender and recruitment site in a block design."
	1.2 Was the allocation sequence concealed until participants were recruited and assigned to interventions?	NI	
	1.3 Were there baseline imbalances that suggest a problem with the randomization process?	N	"Table 1" & "There were no significantdifferences between treatment groups on any of the demographic variables."
	**Risk of bias judgement**	**Some concerns**	Uncertain with the allocation concealment put this domain "some concerns"
**Bias due to deviations from intended interventions**	2.1 Were participants aware of their assigned intervention during the trial?	NI	Not stated.
	2.2 Were carers and trial personnel aware of participants' assigned intervention during the trial?	NI	
	2.3 If Y/PY/NI to 2.1 or 2.2: Were there deviations from the intended intervention beyond what would be expected in usual practice?	PN	"Overall, 83.8% of subjects completed 3 months of psychosocial treatment, as defined by attending at least 80% of the weekly therapy visits. There was no difference between treatment groups in the proportion of subjects completing treatment (89.2% for the placebo group and 81.1% for the naltrexone group; Wald χ2 [1]=0.042; odds ratio (OR): 1.16; 95% confidence interval (CI): 0.28–4.91; p=0.838)."<br>"There was no difference between treatment groups in the proportion of subjects adherent to naltrexone/placebo (Wald χ2 [1]=0.029; OR: 1.11; 95% CI: 0.32–3.84; p=0.864) or sertraline (Wald χ2 [1]=0.511; OR: 1.54; 95% CI: 0.47–5.07; p=0.475)."
	2.4 If Y/PY to 2.3: Were these deviations from intended intervention unbalanced between groups and likely to have affected the outcome?	NA	
	2.5 If N/PN/NI to 2.4: Were these deviations likely to have affected the outcome?	N	No evidence that patients recieved a treatment other than the one they were assigned to.

Domain	Signalling question	Response	Comments
	2.6 If Y/PY/NI to 2.5: Was there potential for a substantial impact (on the estimated effect of intervention) of analysing participants in the wrong group?	NA	
	**Risk of bias judgement**	**Low**	There was no complete information regarding blinding but no difference in attendance of psychosocial treatment and sertraline adherence, suggesting "Low" deviations from intended interventions.
**Bias due to missing outcome data**	3.1 Were outcome data available for all, or nearly all, participants randomized?	PY	
	3.2 If N/PN/NI to 3.1: Are the proportions of missing outcome data and reasons for missing outcome data similar across intervention groups?	NA	
	3.3 If N/PN/NI to 3.1: Is there evidence that results were robust to the presence of missing outcome data?	NA	
	**Risk of bias judgement**	**Low**	The authors did not report drop-outs so we assumed that no missing data - "Low" risk of bias in this domain.
**Bias in measurement of the outcome**	4.1 Were outcome assessors aware of the intervention received by study participants?	NI	No mention of blinding
	4.2 If Y/PY/NI to 4.1: Was the assessment of the outcome likely to be influenced by knowledge of intervention received?	PY	By Time-Line-Follow-Back (patient self-report), placebo controlled trial, so knowldge of intervention may have effected the results of the TLFB , with those on active treatment understating their alcohol consumption
	**Risk of bias judgement**	**High**	No mention of blinding to outcome assessors (participants themselves) and possible chance of knowldge of intervention may have effected outcomes - "High" risk of bias
**Bias in selection of the reported result**	Are the reported outcome data likely to have been selected, on the basis of the results, from…		
	5.1. ... multiple outcome measurements (e.g. scales, definitions, time points) within the outcome domain?	PN	
	5.2 ... multiple analyses of the data?	PN	
	**Risk of bias judgement**	**Low**	No protocol was found but nature of outcome, abstinence, presents low risk of selected reported results. Thus, we rated "Low" risk of bias in this domain.
**Overall bias**	**Risk of bias judgement**	**High**	Lack of detailed methods for the randomisation process and potential bias in outcome measurement, together, these contributed to "High" in overall bias for this trial.

**Reference**	Paille 1995	**Aim**	assignment to intervention (the 'intention-to-treat' effect)	**Source**	Journal article(s) with results of the trial
**Outcome**	Abstinence (360 days)	**Results**	67/361 (Combined: Acamprosae High+Low doses) vs 20/177 (Placebo)		

Domain	Signalling question	Response	Comments
**Bias arising from the randomization process**	1.1 Was the allocation sequence random?	PY	"According to a predetermined randomization list, eligible patients were assigned treatment with either 333 mg Acamprosate tablets on a dosage of four or six tablets per day in divided doses, or a matching placebo tablet."
	1.2 Was the allocation sequence concealed until participants were recruited and assigned to interventions?	NI	
	1.3 Were there baseline imbalances that suggest a problem with the randomization process?	PN	"no significant differences were found between the three groups on any of the variables measured at baseline"
	**Risk of bias judgement**	**Some concerns**	No details were given regarding allocation concealment process, contributed to "some concerns" in this domain.
**Bias due to deviations from intended interventions**	2.1 Were participants aware of their assigned intervention during the trial?	N	"double-blind"
	2.2 Were carers and trial personnel aware of participants' assigned intervention during the trial?	PN	
	2.3 If Y/PY/NI to 2.1 or 2.2: Were there deviations from the intended intervention beyond what would be expected in usual practice?	NA	
	2.4 If Y/PY to 2.3: Were these deviations from intended intervention unbalanced between groups and likely to have affected the outcome?	NA	
	2.5 If N/PN/NI to 2.4: Were these deviations likely to have affected the outcome?	N	"Data were therefore analysed on an intention-to-treat basis. All patients who had taken the treatment at least once were included in the analysis of treatment success or failure." No evidence that patients anlysed in incorrect group.
	2.6 If Y/PY/NI to 2.5: Was there potential for a substantial impact (on the estimated effect of intervention) of analysing participants in the wrong group?	NA	
	**Risk of bias judgement**	**Low**	Double-blind design and ITT analysis employed in this trial put "Low" risk of bias in deviation from intended interventions.
**Bias due to missing outcome data**	3.1 Were outcome data available for all, or nearly all, participants randomized?	PN	PLA (35%) vs ACA (48.5%)
	3.2 If N/PN/NI to 3.1: Are the proportions of missing outcome data and reasons for missing outcome data similar across intervention groups?	PN	More participants in placebo group refused to the treatment or noncompliance
	3.3 If N/PN/NI to 3.1: Is there evidence that results were robust to the presence of missing outcome data?	PN	No sensitivity analyses but the authors treated drop-outs as not abstinent
	**Risk of bias judgement**	**High**	High porportion of missing data and imbalanced missing data without sensitivity analysis put "high" risk of bias in this domain.
**Bias in measurement of the outcome**	4.1 Were outcome assessors aware of the intervention received by study participants?	N	Reported by patients and double-blind
	4.2 If Y/PY/NI to 4.1: Was the assessment of the outcome likely to be influenced by knowledge of intervention received?	NA	
	**Risk of bias judgement**	**Low**	Double-blind design and self-reporting outcome (confirmed by relatives and biochemistry results) put this domain as low risk of bias.
**Bias in selection of the reported result**	Are the reported outcome data likely to have been selected, on the basis of the results, from…		
	5.1. ... multiple outcome measurements (e.g. scales, definitions, time points) within the outcome domain?	PN	
	5.2 ... multiple analyses of the data?	PN	
	**Risk of bias judgement**	**Low**	No protocol was found but nature of outcome, abstinence, presents low risk of selected reported results and the authors described the method section clearly. Thus, we rated "Low" risk of bias in this domain.
**Overall bias**	**Risk of bias judgement**	**High**	Lack of detailed methods for the randomisation process and potential bias due to imbalanced missing data, together, these contributed to "High" risk of bias in overall bias for this trial.

**Reference**	Pelc 1992	**Aim**	assignment to intervention (the 'intention-to-treat' effect)	**Source**	Journal article(s) with results of the trial; Other systematic review and meta-anaylsis
**Outcome**	Abstinence (180 days)	**Results**	14/55 (Acamprosate 1999 mg) vs 2/47 (Placebo)		

Domain	Signalling question	Response	Comments
**Bias arising from the randomization process**	1.1 Was the allocation sequence random?	NI	Not information on randomisation or concealment
	1.2 Was the allocation sequence concealed until participants were recruited and assigned to interventions?	NI	
	1.3 Were there baseline imbalances that suggest a problem with the randomization process?	NI	

	**Risk of bias judgement**	**Some concerns**	No details were given regarding randomisation process, contributed to "some concerns" in this domain.
**Bias due to deviations from intended interventions**	2.1 Were participants aware of their assigned intervention during the trial?	PN	"double-blind"
	2.2 Were carers and trial personnel aware of participants' assigned intervention during the trial?	PN	
	2.3 If Y/PY/NI to 2.1 or 2.2: Were there deviations from the intended intervention beyond what would be expected in usual practice?	NA	
	2.4 If Y/PY to 2.3: Were these deviations from intended intervention unbalanced between groups and likely to have affected the outcome?	NA	
	2.5 If N/PN/NI to 2.4: Were these deviations likely to have affected the outcome?	PN	Not clear from content but no suggestion of this.
	2.6 If Y/PY/NI to 2.5: Was there potential for a substantial impact (on the estimated effect of intervention) of analysing participants in the wrong group?	NA	
	**Risk of bias judgement**	**Low**	Double-blind design (though very little information) employed in this trial put "Low" risk of bias in deviation from intended interventions.
**Bias due to missing outcome data**	3.1 Were outcome data available for all, or nearly all, participants randomized?	N	80% for placebo vs ~50% for intervention
	3.2 If N/PN/NI to 3.1: Are the proportions of missing outcome data and reasons for missing outcome data similar across intervention groups?	N	More drop-out in placebo group
	3.3 If N/PN/NI to 3.1: Is there evidence that results were robust to the presence of missing outcome data?	PN	No information how did the authors handle the data
	**Risk of bias judgement**	**High**	High porportion of missing data and no detailed methods for missing data put "High" risk of bias in this domain.
**Bias in measurement of the outcome**	4.1 Were outcome assessors aware of the intervention received by study participants?	PN	Did not indicate the methods for assessing abstinence but the authors stated this was a double-blind study
	4.2 If Y/PY/NI to 4.1: Was the assessment of the outcome likely to be influenced by knowledge of intervention received?	NA	
	**Risk of bias judgement**	**Low**	Double-blind design put this domain as low risk of bias.
**Bias in selection of the reported result**	Are the reported outcome data likely to have been selected, on the basis of the results, from…		
	5.1. ... multiple outcome measurements (e.g. scales, definitions, time points) within the outcome domain?	NI	
	5.2 ... multiple analyses of the data?	NI	
	**Risk of bias judgement**	**Some concerns**	No protocol was found and the authors did not describe the method for abstinence clearly. Thus, we rated "Some concerns" risk of bias in this domain.
**Overall bias**	**Risk of bias judgement**	**High**	High risk of bias in missing data, some concerns in randomisation process and little information regarding the methods and analyses contributed to "High" risk of bias in overall bias.

**Reference**	Pelc 2005	**Aim**	assignment to intervention (the 'intention-to-treat' effect)	**Source**	Journal article(s) with results of the trial
**Outcome**	Abstinence (26 weeks)	**Results**	8/50 (Acamprosate) vs 16/50 (Acamprosate + Nurse follow-up)		

Domain	Signalling question	Response	Comments
**Bias arising from the randomization process**	1.1 Was the allocation sequence random?	Y	"Investigators telephoned the centre before the inclusion of each subject, to obtain a randomization number defining the group to which the patient was to be assigned."
	1.2 Was the allocation sequence concealed until participants were recruited and assigned to interventions?	Y	
	1.3 Were there baseline imbalances that suggest a problem with the randomization process?	PN	There were a few differences in the educational status among two groups and marital status but these should be caused by chance rather than a problem with randoimization process.
	**Risk of bias judgement**	**Low**	This study employed adequate randomisation methods in the trial and represents low risk of bias in the randomisation process.
**Bias due to deviations from intended interventions**	2.1 Were participants aware of their assigned intervention during the trial?	Y	An open study.
	2.2 Were carers and trial personnel aware of participants' assigned intervention during the trial?	Y	
	2.3 If Y/PY/NI to 2.1 or 2.2: Were there deviations from the intended intervention beyond what would be expected in usual practice?	PY	There was a high attendence in the self-group participantion in the "no nurse follow-up" group.
	2.4 If Y/PY to 2.3: Were these deviations from intended intervention unbalanced between groups and likely to have affected the outcome?	PY	The subgroup analysis showed that self-help group participation had interaction with treatment<br><br>"Although the results should be interpreted cautiously on account of the relatively low number of patients, significant treatment interactions were observed between gender and participation in self-help groups."
	2.5 If N/PN/NI to 2.4: Were these deviations likely to have affected the outcome?	N	ITT analysis
	2.6 If Y/PY/NI to 2.5: Was there potential for a substantial impact (on the estimated effect of intervention) of analysing participants in the wrong group?	NA	
	**Risk of bias judgement**	**High**	Open-label design and possible deviations from intended interventions contributed to "High" risk of bias
**Bias due to missing outcome data**	3.1 Were outcome data available for all, or nearly all, participants randomized?	N	High proportion of missing data (Figure 1)
	3.2 If N/PN/NI to 3.1: Are the proportions of missing outcome data and reasons for missing outcome data similar across intervention groups?	PN	Numbers and reasons were different between groups.
	3.3 If N/PN/NI to 3.1: Is there evidence that results were robust to the presence of missing outcome data?	PN	No sensitivity analysis
	**Risk of bias judgement**	**High**	High porportion of missing data and imbalanced missing data without sensitivity analysis put "high" risk of bias in this domain.
**Bias in measurement of the outcome**	4.1 Were outcome assessors aware of the intervention received by study participants?	Y	Should be patient self-report and this trial is an open trial so outcome assessors were aware of the intervention received by study participants.
	4.2 If Y/PY/NI to 4.1: Was the assessment of the outcome likely to be influenced by knowledge of intervention received?	PY	Possible influence by the knowledge of interventions
	**Risk of bias judgement**	**High**	This is an open study and the outcome (abstinence) could be influenced by the knowledge of interventions. Therefore, we rated "High" in this domain.
**Bias in selection of the reported result**	Are the reported outcome data likely to have been selected, on the basis of the results, from…		
	5.1. ... multiple outcome measurements (e.g. scales, definitions, time points) within the outcome domain?	PN	
	5.2 ... multiple analyses of the data?	PN	
	**Risk of bias judgement**	**Low**	No protocol was found but nature of outcome, abstinence, presents low risk of selected reported results and the authors described the method section clearly. Thus, we rated "Low" risk of bias in this domain.
**Overall bias**	**Risk of bias judgement**	**High**	High risk of bias in the deviations from the intended interventions, some concerns in missing data and outcome measurements put "High" risk of bias in overall bias.

**Reference**	Pelc 1997	**Aim**	assignment to intervention (the 'intention-to-treat' effect)	**Source**	Journal article(s) with results of the trial
**Outcome**	Abstinence (90 days)	**Results**	60/126 (Combined: Acamprosate High+Low doses) vs 16/62 (Placebo)		

Domain	Signalling question	Response	Comments
**Bias arising from the randomization process**	1.1 Was the allocation sequence random?	NI	Only stated "Random"; "The patients were randomly assigned to one of the three treatment groups..."
	1.2 Was the allocation sequence concealed until participants were recruited and assigned to interventions?	NI	
	1.3 Were there baseline imbalances that suggest a problem with the randomization process?	N	Equal number of participants in each group and no difference among characteristics of participants.<br>"A total of 188 patients were included in the trial: 62 were randomised to placebo (placebo), 63 to acamprosate 1332 mg/day (acamp. 1332) and 63 to acamprosate 1998 mg/day (acamp. 1998). No statistical difference was present for any criterion at inclusion."
	**Risk of bias judgement**	**Some concerns**	No details were given regarding randomisation process, contributed to "some concerns" in this domain.
**Bias due to deviations from intended interventions**	2.1 Were participants aware of their assigned intervention during the trial?	PN	"double-blind"
	2.2 Were carers and trial personnel aware of participants' assigned intervention during the trial?	PN	
	2.3 If Y/PY/NI to 2.1 or 2.2: Were there deviations from the intended intervention beyond what would be expected in usual practice?	NA	
	2.4 If Y/PY to 2.3: Were these deviations from intended intervention unbalanced between groups and likely to have affected the outcome?	NA	
	2.5 If N/PN/NI to 2.4: Were these deviations likely to have affected the outcome?	N	ITT analysis
	2.6 If Y/PY/NI to 2.5: Was there potential for a substantial impact (on the estimated effect of intervention) of analysing participants in the wrong group?	NA	
	**Risk of bias judgement**	**Low**	Double-blind design and ITT analysis employed in this trial put "Low" risk of bias in deviation from intended interventions.
**Bias due to missing outcome data**	3.1 Were outcome data available for all, or nearly all, participants randomized?	PN	loss to follow up 24% control, 9.5% acamp 1332, 9.5% acadmp 1998. so uneven and reasons not given. total 32% lost from placebo (excl relapse) ~20% acadmp 1332 and 17% from acamp 1998
	3.2 If N/PN/NI to 3.1: Are the proportions of missing outcome data and reasons for missing outcome data similar across intervention groups?	PN	Difference in the proportions of missing data was observed and no details were given
	3.3 If N/PN/NI to 3.1: Is there evidence that results were robust to the presence of missing outcome data?	PN	No sensitivity analysis was performed. The authors treated participants dropped out as non abstinent
	**Risk of bias judgement**	**High**	High porportion of missing data and imbalanced missing data without sensitivity analysis put "high" risk of bias in this domain.
**Bias in measurement of the outcome**	4.1 Were outcome assessors aware of the intervention received by study participants?	N	"double-blind"; alcohol consumption was assessed by review of patients' diary consumption cards and confirmed by urine test at each test.
	4.2 If Y/PY/NI to 4.1: Was the assessment of the outcome likely to be influenced by knowledge of intervention received?	NA	
	**Risk of bias judgement**	**Low**	Double-blind design and self-reporting outcome (confirmed by biochemistry results) put this domain as low risk of bias.
**Bias in selection of the reported result**	Are the reported outcome data likely to have been selected, on the basis of the results, from…		
	5.1. ... multiple outcome measurements (e.g. scales, definitions, time points) within the outcome domain?	PN	
	5.2 ... multiple analyses of the data?	PN	
	**Risk of bias judgement**	**Low**	No protocol was found but nature of outcome, abstinence, presents low risk of selected reported results and the authors described the method section clearly. Thus, we rated "Low" risk of bias in this domain.
**Overall bias**	**Risk of bias judgement**	**High**	Lack of detailed methods for the randomisation process and imbalanced missing data, together, these contributed to "High" in overall bias for this trial.

**Reference**	Poldrugo 1997	**Aim**	assignment to intervention (the 'intention-to-treat' effect)	**Source**	Journal article(s) with results of the trial
**Outcome**	Abstinence (12 months)	**Results**	53/122 (Acamprosate) vs 37/124 (Placebo)		

Domain	Signalling question	Response	Comments
**Bias arising from the randomization process**	1.1 Was the allocation sequence random?	PY	Did not stated the method but "Patients were randomized by individual subject randomization to the acamprosate group..."
	1.2 Was the allocation sequence concealed until participants were recruited and assigned to interventions?	PY	
	1.3 Were there baseline imbalances that suggest a problem with the randomization process?	N	No significant difference found between groups.
	**Risk of bias judgement**	**Low**	
**Bias due to deviations from intended interventions**	2.1 Were participants aware of their assigned intervention during the trial?	PN	"double-blind"
	2.2 Were carers and trial personnel aware of participants' assigned intervention during the trial?	PN	
	2.3 If Y/PY/NI to 2.1 or 2.2: Were there deviations from the intended intervention beyond what would be expected in usual practice?	NA	
	2.4 If Y/PY to 2.3: Were these deviations from intended intervention unbalanced between groups and likely to have affected the outcome?	NA	
	2.5 If N/PN/NI to 2.4: Were these deviations likely to have affected the outcome?	N	
	2.6 If Y/PY/NI to 2.5: Was there potential for a substantial impact (on the estimated effect of intervention) of analysing participants in the wrong group?	NA	
	**Risk of bias judgement**	**Low**	
**Bias due to missing outcome data**	3.1 Were outcome data available for all, or nearly all, participants randomized?	N	There were 46.7% (Ac) and 62.1% (Pl) withdrawn in the group and more during the follow-up periods.
	3.2 If N/PN/NI to 3.1: Are the proportions of missing outcome data and reasons for missing outcome data similar across intervention groups?	PN	More participants in placebo group refused to continue and severe relapsed.
	3.3 If N/PN/NI to 3.1: Is there evidence that results were robust to the presence of missing outcome data?	N	Although the authors used ITT analyses, the imbalanced missing data could not premit robust results.
	**Risk of bias judgement**	**High**	Imbalanced missing outcome data across the groups led to high risk of bias
**Bias in measurement of the outcome**	4.1 Were outcome assessors aware of the intervention received by study participants?	PN	"double-blind"
	4.2 If Y/PY/NI to 4.1: Was the assessment of the outcome likely to be influenced by knowledge of intervention received?	NA	
	**Risk of bias judgement**	**Low**	

**Bias in selection of the reported result**	Are the reported outcome data likely to have been selected, on the basis of the results, from…		
	5.1. ... multiple outcome measurements (e.g. scales, definitions, time points) within the outcome domain?	N	
	5.2 ... multiple analyses of the data?	N	
	**Risk of bias judgement**	**Low**	No protocol was found but nature of outcome, abstinence, presents low risk of selected reported results. Thus, we rated "Low" risk of bias in this domain.
**Overall bias**	**Risk of bias judgement**	**High**	High risk of bias due to imbalanced missing outcome data.

**Reference**	Ponce 2005	**Aim**	assignment to intervention (the 'intention-to-treat' effect)	**Source**	Journal article(s) with results of the trial
**Outcome**	Abstinence (12 weeks)	**Results**	38/50 (Naltrexone 50mg) vs 21/50 (TAU)		

**Domain**	**Signalling question**	**Response**	**Comments**
**Bias arising from the randomization process**	1.1 Was the allocation sequence random?	NI	Only stated "random"
	1.2 Was the allocation sequence concealed until participants were recruited and assigned to interventions?	NI	
	1.3 Were there baseline imbalances that suggest a problem with the randomization process?	N	No significant difference found between groups at baseline (table 1)
	**Risk of bias judgement**	**Some concerns**	No details were given regarding randomisation process, contributed to "some concerns" in this domain.
**Bias due to deviations from intended interventions**	2.1 Were participants aware of their assigned intervention during the trial?	PN	"single-blind" but it was impossible to blind participants as this trial did not provide placebo.
	2.2 Were carers and trial personnel aware of participants' assigned intervention during the trial?	PY	
	2.3 If Y/PY/NI to 2.1 or 2.2: Were there deviations from the intended intervention beyond what would be expected in usual practice?	NI	
	2.4 If Y/PY to 2.3: Were these deviations from intended intervention unbalanced between groups and likely to have affected the outcome?	NA	
	2.5 If N/PN/NI to 2.4: Were these deviations likely to have affected the outcome?	N	No switching
	2.6 If Y/PY/NI to 2.5: Was there potential for a substantial impact (on the estimated effect of intervention) of analysing participants in the wrong group?	NA	
	**Risk of bias judgement**	**Some concerns**	It was impossible to blind participants in this trial due to nature of interventions employed, which potentially induce deviations from intended interventions. On the other hand, the authors applied ITT analyses. Together, these contributed to "some concerns" in this domain.
**Bias due to missing outcome data**	3.1 Were outcome data available for all, or nearly all, participants randomized?	N	19 (NAT) and 8 (Non) abandoned the trial. (27/100 abandoned treatment.)
	3.2 If N/PN/NI to 3.1: Are the proportions of missing outcome data and reasons for missing outcome data similar across intervention groups?	N	More patients left the trial in no treatment group.
	3.3 If N/PN/NI to 3.1: Is there evidence that results were robust to the presence of missing outcome data?	PN	No sensitivity analysis
	**Risk of bias judgement**	**High**	High porportion of missing data and imbalanced missing data without sensitivity analysis put "high" risk of bias in this domain.
**Bias in measurement of the outcome**	4.1 Were outcome assessors aware of the intervention received by study participants?	PN	Participants was blinded
	4.2 If Y/PY/NI to 4.1: Was the assessment of the outcome likely to be influenced by knowledge of intervention received?	NA	
	**Risk of bias judgement**	**Low**	Single-blind design and self-reporting outcome (confirmed by relatives and biochemistry results) put this domain as low risk of bias.
**Bias in selection of the reported result**	Are the reported outcome data likely to have been selected, on the basis of the results, from…		
	5.1. ... multiple outcome measurements (e.g. scales, definitions, time points) within the outcome domain?	PN	
	5.2 ... multiple analyses of the data?	PN	
	**Risk of bias judgement**	**Low**	No protocol was found but nature of outcome, abstinence, presents low risk of selected reported results. Thus, we rated "Low" risk of bias in this domain.
**Overall bias**	**Risk of bias judgement**	**High**	High risk of bias derived from the missing data, deviations from the intended interventions and lack of details in the randomisation process, together, these put "High" risk of bias.

**Reference**	Richter 2012	**Aim**	assignment to intervention (the 'intention-to-treat' effect)	**Source**	Journal article(s) with results of the trial
**Outcome**	Abstinence (16 weeks)	**Results**	33/95 (Levetiracetam 2000 mg) vs 36/106 (Placebo)		

**Domain**	**Signalling question**	**Response**	**Comments**
**Bias arising from the randomization process**	1.1 Was the allocation sequence random?	Y	"Randomization was computerized, central and independent of the center, and blinded for physician and participants."
	1.2 Was the allocation sequence concealed until participants were recruited and assigned to interventions?	Y	
	1.3 Were there baseline imbalances that suggest a problem with the randomization process?	N	No significant difference between groups (Table 1).
	**Risk of bias judgement**	**Low**	This study employed adequate randomisation methods in the trial and represents low risk of bias in the randomisation process.
**Bias due to deviations from intended interventions**	2.1 Were participants aware of their assigned intervention during the trial?	N	"double-blind"; "Randomization was computerized, central and independent of the center, and blinded for physician and participants."
	2.2 Were carers and trial personnel aware of participants' assigned intervention during the trial?	N	
	2.3 If Y/PY/NI to 2.1 or 2.2: Were there deviations from the intended intervention beyond what would be expected in usual practice?	NA	
	2.4 If Y/PY to 2.3: Were these deviations from intended intervention unbalanced between groups and likely to have affected the outcome?	NA	
	2.5 If N/PN/NI to 2.4: Were these deviations likely to have affected the outcome?	N	ITT analysis
	2.6 If Y/PY/NI to 2.5: Was there potential for a substantial impact (on the estimated effect of intervention) of analysing participants in the wrong group?	NA	
	**Risk of bias judgement**	**Low**	Double-blind design and ITT analysis employed in this trial put "Low" risk of bias in deviation from intended interventions.

Bias due to missing outcome data	3.1 Were outcome data available for all, or nearly all, participants randomized?	N	"Overall, 79% patients (n = 158) completed the trial per protocol: 80 (75%) of 106 in the placebo and 78 (82%) of 95 in the LEV group."
	3.2 If N/PN/NI to 3.1: Are the proportions of missing outcome data and reasons for missing outcome data similar across intervention groups?	PN	"Dropout reasons in the placebo group (n = 26) were depression with suicidal tendency (severe AE), a panic attack (severe AE), 9 side effects, a refusal of participation, 8 noncompliances, 2 other causes, 4 unknown reasons. In the LEV group (n = 12), we counted 2 side effects, a refusal of participation, 8 noncompliances, and 1 unknown reason as dropouts."
	3.3 If N/PN/NI to 3.1: Is there evidence that results were robust to the presence of missing outcome data?	PN	No sensitivity analysis but the authors treated drop-outs as non abstinent
	**Risk of bias judgement**	**High**	High porportion of missing data and imbalanced missing data without sensitivity analysis put "high" risk of bias in this domain.
Bias in measurement of the outcome	4.1 Were outcome assessors aware of the intervention received by study participants?	N	"double-blind"
	4.2 If Y/PY/NI to 4.1: Was the assessment of the outcome likely to be influenced by knowledge of intervention received?	NA	
	**Risk of bias judgement**	**Low**	Double-blind design and self-reporting outcome (confirmed by relatives and biochemistry results) put this domain as low risk of bias.
Bias in selection of the reported result	Are the reported outcome data likely to have been selected, on the basis of the results, from…		
	5.1. ... multiple outcome measurements (e.g. scales, definitions, time points) within the outcome domain?	PN	
	5.2 ... multiple analyses of the data?	PN	
	**Risk of bias judgement**	**Low**	No protocol was found but nature of outcome, abstinence, presents low risk of selected reported results and the authors described the method section clearly. Thus, we rated "Low" risk of bias in this domain.
**Overall bias**	**Risk of bias judgement**	**High**	High risk of bias in overall bias domain due to imbalanced missing data.

**Reference**	Rubio 2005	**Aim**	assignment to intervention (the 'intention-to-treat' effect)	**Source**	Journal article(s) with results of the trial
**Outcome**	Abstinence (12 weeks)	**Results**	111/168 (Naltrexone 50 mg) vs 95/168 (TAU)		

Domain	Signalling question	Response	Comments
Bias arising from the randomization process	1.1 Was the allocation sequence random?	NI	Only stated "random"
	1.2 Was the allocation sequence concealed until participants were recruited and assigned to interventions?	NI	
	1.3 Were there baseline imbalances that suggest a problem with the randomization process?	N	No significant difference between groups (Table 1).
	**Risk of bias judgement**	**Some concerns**	No details were given regarding randomisation process, contributed to "some concerns" in this domain.
Bias due to deviations from intended interventions	2.1 Were participants aware of their assigned intervention during the trial?	Y	physician and patient being open to the study medication... ...more closely mirrors routine clinical practice in Spain, than a double-blind study...'
	2.2 Were carers and trial personnel aware of participants' assigned intervention during the trial?	Y	
	2.3 If Y/PY/NI to 2.1 or 2.2: Were there deviations from the intended intervention beyond what would be expected in usual practice?	NI	
	2.4 If Y/PY to 2.3: Were these deviations from intended intervention unbalanced between groups and likely to have affected the outcome?	NA	
	2.5 If N/PN/NI to 2.4: Were these deviations likely to have affected the outcome?	PN	seems to be ITT analysis
	2.6 If Y/PY/NI to 2.5: Was there potential for a substantial impact (on the estimated effect of intervention) of analysing participants in the wrong group?	NA	
	**Risk of bias judgement**	**Some concerns**	The open label design in this trial might prompt deviations from the intended interventions, contributing to "some concerns" in this domain.
Bias due to missing outcome data	3.1 Were outcome data available for all, or nearly all, participants randomized?	N	high rate of drop outs (27.98% in NAL and 34.52% in control)
	3.2 If N/PN/NI to 3.1: Are the proportions of missing outcome data and reasons for missing outcome data similar across intervention groups?	NI	The proportion of drop-outs did not differ between groups but there was no reasons reported.
	3.3 If N/PN/NI to 3.1: Is there evidence that results were robust to the presence of missing outcome data?	PN	No information regarding the imputation methods
	**Risk of bias judgement**	**Some concerns**	There were some missing data, which were not evenly distributed in both groups and no details reported. "Some concerns" was rated in this domain.
Bias in measurement of the outcome	4.1 Were outcome assessors aware of the intervention received by study participants?	Y	"...on the basis of the participant's self data on alcohol intake and consumption pattern (Miller, 1996)"; "In addition, the following biological parameters of alcohol use were used: serum aspartate aminotransferase (AST), alanine aminotransferase (ALT), gamma-glutamyltransferase (GGT), and carbohydrate-deficient transferrin (CDT)."
	4.2 If Y/PY/NI to 4.1: Was the assessment of the outcome likely to be influenced by knowledge of intervention received?	PN	"...on the basis of the participant's self data on alcohol intake and consumption pattern (Miller, 1996)"; "In addition, the following biological parameters of alcohol use were used: serum aspartate aminotransferase (AST), alanine aminotransferase (ALT), gamma-glutamyltransferase (GGT), and carbohydrate-deficient transferrin (CDT)."
	**Risk of bias judgement**	**Low**	Although this was an open-label study, outcome was confirmed by biochemistry methods.
Bias in selection of the reported result	Are the reported outcome data likely to have been selected, on the basis of the results, from…		
	5.1. ... multiple outcome measurements (e.g. scales, definitions, time points) within the outcome domain?	PN	
	5.2 ... multiple analyses of the data?	PN	
	**Risk of bias judgement**	**Low**	No protocol was found but nature of outcome, abstinence, presents low risk of selected reported results and the authors described the method section clearly. Thus, we rated "Low" risk of bias in this domain.
**Overall bias**	**Risk of bias judgement**	**Some concerns**	Some concerns due to lack of details in randomisaion process, open-label design and no details in missing data.

**Reference**	Sass 1996	**Aim**	assignment to intervention (the 'intention-to-treat' effect)	**Source**	Journal article(s) with results of the trial
**Outcome**	Abstinence (48 weeks)	**Results**	61/136 (Acamprosate 1998 mg) vs 34/136 (Placebo)		

Domain	Signalling question	Response	Comments
Bias arising from the randomization process	1.1 Was the allocation sequence random?	Y	"Sealed envelope randomization with balance by blocks of 8 (4 per study medication) was used to obtain equal numbers per treatment group at each center."
	1.2 Was the allocation sequence concealed until participants were recruited and assigned to interventions?	Y	
	1.3 Were there baseline imbalances that suggest a problem with the randomization process?	N	

	**Risk of bias judgement**	**Low**	This study employed adequate randomisation methods in the trial and represents low risk of bias in the randomisation process.
**Bias due to deviations from intended interventions**	2.1 Were participants aware of their assigned intervention during the trial?	N	"double-blind"; Treatment consisted of counseling or psychotherapy..., to which blinded study medication...."
	2.2 Were carers and trial personnel aware of participants' assigned intervention during the trial?	N	
	2.3 If Y/PY/NI to 2.1 or 2.2: Were there deviations from the intended intervention beyond what would be expected in usual practice?	NA	
	2.4 If Y/PY to 2.3: Were these deviations from intended intervention unbalanced between groups and likely to have affected the outcome?	NA	
	2.5 If N/PN/NI to 2.4: Were these deviations likely to have affected the outcome?	N	ITT analyses condcuted
	2.6 If Y/PY/NI to 2.5: Was there potential for a substantial impact (on the estimated effect of intervention) of analysing participants in the wrong group?	NA	
	**Risk of bias judgement**	**Low**	Double-blind design and ITT analysis employed in this trial put "Low" risk of bias in deviation from intended interventions.
**Bias due to missing outcome data**	3.1 Were outcome data available for all, or nearly all, participants randomized?	N	"33% of placeob treated patients unwilling to continue vs 14.7% of acamprosate treated patients. large proportion withdrawn, 42% acamprosate; 60% placebo."<br>Appears that 16% more patients treated with placebo relapsed and withdrew.
	3.2 If N/PN/NI to 3.1: Are the proportions of missing outcome data and reasons for missing outcome data similar across intervention groups?	PN	As above
	3.3 If N/PN/NI to 3.1: Is there evidence that results were robust to the presence of missing outcome data?	PN	The authors presented PP and ITT results. No sensitivity analysis but treated drop-outs as non abstinent
	**Risk of bias judgement**	**High**	High porportion of missing data and imbalanced missing data without sensitivity analysis put "high" risk of bias in this domain.
**Bias in measurement of the outcome**	4.1 Were outcome assessors aware of the intervention received by study participants?	N	"Double-blind"; "A patient's declaration of drinking behavior was verified by the results of a breathalyzer test and GGT levels in every case and by interviewing a family member if possible."
	4.2 If Y/PY/NI to 4.1: Was the assessment of the outcome likely to be influenced by knowledge of intervention received?	NA	
	**Risk of bias judgement**	**Low**	Double-blind design and self-reporting outcome (confirmed by relatives and biochemistry results) put this domain as low risk of bias.
**Bias in selection of the reported result**	Are the reported outcome data likely to have been selected, on the basis of the results, from…		
	5.1. ... multiple outcome measurements (e.g. scales, definitions, time points) within the outcome domain?	PN	
	5.2 ... multiple analyses of the data?	PN	
	**Risk of bias judgement**	**Low**	No protocol was found but nature of outcome, abstinence, presents low risk of selected reported results and the authors described the method section clearly. Thus, we rated "Low" risk of bias in this domain.
**Overall bias**	**Risk of bias judgement**	**High**	High risk of bias in overall bias due to imbalanced missing data.

**Reference**	Schmidt 2002	**Aim**	assignment to intervention (the 'intention-to-treat' effect)	**Source**	Journal article(s) with results of the trial
**Outcome**	Abstinence (12 months)	**Results**	8/57 (Lisuride) vs 19/63 (Placebo)		

Domain	Signalling question	Response	Comments
**Bias arising from the randomization process**	1.1 Was the allocation sequence random?	NI	Only stated "random"
	1.2 Was the allocation sequence concealed until participants were recruited and assigned to interventions?	NI	
	1.3 Were there baseline imbalances that suggest a problem with the randomization process?	N	No evidence of differences in baseline characteristics.
	**Risk of bias judgement**	**Some concerns**	No details were given regarding randomisation process, contributed to "some concerns" in this domain.
**Bias due to deviations from intended interventions**	2.1 Were participants aware of their assigned intervention during the trial?	PN	"double-blind"
	2.2 Were carers and trial personnel aware of participants' assigned intervention during the trial?	PN	
	2.3 If Y/PY/NI to 2.1 or 2.2: Were there deviations from the intended intervention beyond what would be expected in usual practice?	NA	
	2.4 If Y/PY to 2.3: Were these deviations from intended intervention unbalanced between groups and likely to have affected the outcome?	NA	
	2.5 If N/PN/NI to 2.4: Were these deviations likely to have affected the outcome?	N	"intend to treat analysis" and no evidence of swapping patients
	2.6 If Y/PY/NI to 2.5: Was there potential for a substantial impact (on the estimated effect of intervention) of analysing participants in the wrong group?	NA	
	**Risk of bias judgement**	**Low**	Double-blind design and ITT analysis employed in this trial put "Low" risk of bias in deviation from intended interventions.
**Bias due to missing outcome data**	3.1 Were outcome data available for all, or nearly all, participants randomized?	N	There were some missing data (Table 2)
	3.2 If N/PN/NI to 3.1: Are the proportions of missing outcome data and reasons for missing outcome data similar across intervention groups?	N	Similar proportions of missing outcome data but more participants in lisuride group (n = 9) dropped out due to adverse event compared to placebo group (n = 3)
	3.3 If N/PN/NI to 3.1: Is there evidence that results were robust to the presence of missing outcome data?	PN	No sensitivity analysis
	**Risk of bias judgement**	**High**	The authors removed 16 patients from the ITT analysis and imbalanced missing data in adverse events put this domain in "High" risk of bias.
**Bias in measurement of the outcome**	4.1 Were outcome assessors aware of the intervention received by study participants?	PN	"double-blind"
	4.2 If Y/PY/NI to 4.1: Was the assessment of the outcome likely to be influenced by knowledge of intervention received?	NA	
	**Risk of bias judgement**	**Low**	Double-blind design and self-reporting outcome put this domain as low risk of bias.
**Bias in selection of the reported result**	Are the reported outcome data likely to have been selected, on the basis of the results, from…		
	5.1. ... multiple outcome measurements (e.g. scales, definitions, time points) within the outcome domain?	PN	
	5.2 ... multiple analyses of the data?	PN	
	**Risk of bias judgement**	**Low**	No protocol was found but nature of outcome, abstinence, presents low risk of selected reported results and the authors described the method section clearly. Thus, we rated "Low" risk of bias in this domain.
**Overall bias**	**Risk of bias judgement**	**High**	Lack of detailed methods for the randomisation process contributed to "High" risk of bias in overall bias for this trial.

Reference	Stella 2008	Aim	assignment to intervention (the 'intention-to-treat' effect)	Source	Journal article(s) with results of the trial
Outcome	Abstinence (6 months)	Results	2/12 (Escitalopram 20 mg) vs 6/12 (GHB 75 mg/kg + EST 20 mg) vs 4/12 (NTX 50mg + EST 20 mg) vs 10/12 (NTX 50mg + GHB 75mg/kg + ETP 20 mg)		

Domain	Signalling question	Response	Comments
Bias arising from the randomization process	1.1 Was the allocation sequence random?	NI	Only stated "random", "They were randomized into four groups."
	1.2 Was the allocation sequence concealed until participants were recruited and assigned to interventions?	NI	
	1.3 Were there baseline imbalances that suggest a problem with the randomization process?	PN	Same number in each group. Some difference in education status and employment but might be from by chance
	**Risk of bias judgement**	**Some concerns**	No details were given regarding randomisation process, contributed to "some concerns" in this domain.
Bias due to deviations from intended interventions	2.1 Were participants aware of their assigned intervention during the trial?	Y	"open trial"
	2.2 Were carers and trial personnel aware of participants' assigned intervention during the trial?	PY	
	2.3 If Y/PY/NI to 2.1 or 2.2: Were there deviations from the intended intervention beyond what would be expected in usual practice?	NI	They all received pharmacological and psychological interventions.
	2.4 If Y/PY to 2.3: Were these deviations from intended intervention unbalanced between groups and likely to have affected the outcome?	NA	
	2.5 If N/PN/NI to 2.4: Were these deviations likely to have affected the outcome?	N	Seemed to be ITT analysis
	2.6 If Y/PY/NI to 2.5: Was there potential for a substantial impact (on the estimated effect of intervention) of analysing participants in the wrong group?	NA	
	**Risk of bias judgement**	**Some concerns**	The open label design in this trial might prompt deviations from the intended interventions, contributing to "some concerns" in this domain.
Bias due to missing outcome data	3.1 Were outcome data available for all, or nearly all, participants randomized?	PY	Did not provide information regarding missing outcome data.
	3.2 If N/PN/NI to 3.1: Are the proportions of missing outcome data and reasons for missing outcome data similar across intervention groups?	NA	
	3.3 If N/PN/NI to 3.1: Is there evidence that results were robust to the presence of missing outcome data?	NA	
	**Risk of bias judgement**	**Low**	The authors did not provide missing data so the reviewer assumed there was no missing data, especially this trial involved small numbers of participants. "Low" risk of bias
Bias in measurement of the outcome	4.1 Were outcome assessors aware of the intervention received by study participants?	Y	Open study
	4.2 If Y/PY/NI to 4.1: Was the assessment of the outcome likely to be influenced by knowledge of intervention received?	PY	
	**Risk of bias judgement**	**High**	Open study and the outcome can be influenced by the knowledge of interventions - "High" risk of bias
Bias in selection of the reported result	Are the reported outcome data likely to have been selected, on the basis of the results, from…		
	5.1. ... multiple outcome measurements (e.g. scales, definitions, time points) within the outcome domain?	PN	
	5.2 ... multiple analyses of the data?	PN	
	**Risk of bias judgement**	**Low**	No protocol was found but nature of outcome, abstinence, presents low risk of selected reported results and the authors described the method section clearly. Thus, we rated "Low" risk of bias in this domain.
**Overall bias**	**Risk of bias judgement**	**High**	Lack of information in randomisation process and open-label design prompted "High" risk of bias in this trial.

Reference	Tempesta 2000	Aim	assignment to intervention (the 'intention-to-treat' effect)	Source	Journal article(s) with results of the trial
Outcome	Abstinence (270 days)	Results	62/164 (Acamprosate 1998 mg) vs 48/166 (Placebo)		

Domain	Signalling question	Response	Comments
Bias arising from the randomization process	1.1 Was the allocation sequence random?	PY	"...randomized, by sealed envelope with balance by blocks of eight..."
	1.2 Was the allocation sequence concealed until participants were recruited and assigned to interventions?	PY	
	1.3 Were there baseline imbalances that suggest a problem with the randomization process?	PN	Placebo group had higher proportion of patients with high consumption awareness and previous treatment for alcoholism, which might result by chance.
	**Risk of bias judgement**	**Low**	This study employed adequate randomisation methods in the trial and represents "Low" risk of bias in the randomisation process.
Bias due to deviations from intended interventions	2.1 Were participants aware of their assigned intervention during the trial?	N	double-blind; identical placebo
	2.2 Were carers and trial personnel aware of participants' assigned intervention during the trial?	PN	Did not mention the blinding of carers and trial personnel. Based on double-blind, the reviewer chose "probably not"
	2.3 If Y/PY/NI to 2.1 or 2.2: Were there deviations from the intended intervention beyond what would be expected in usual practice?	NA	
	2.4 If Y/PY to 2.3: Were these deviations from intended intervention unbalanced between groups and likely to have affected the outcome?	NA	
	2.5 If N/PN/NI to 2.4: Were these deviations likely to have affected the outcome?	N	"Intention to treat (ITT) statistical principles were followed"
	2.6 If Y/PY/NI to 2.5: Was there potential for a substantial impact (on the estimated effect of intervention) of analysing participants in the wrong group?	NA	
	**Risk of bias judgement**	**Low**	Double-blind design and ITT analysis employed in this trial put "Low" risk of bias in deviation from intended interventions.
Bias due to missing outcome data	3.1 Were outcome data available for all, or nearly all, participants randomized?	N	High proportion of drop-outs and lost to follow-up - 25% of patients dropped out over course of study.
	3.2 If N/PN/NI to 3.1: Are the proportions of missing outcome data and reasons for missing outcome data similar across intervention groups?	PY	Reasons and % seem similar across arms (Table 2)
	3.3 If N/PN/NI to 3.1: Is there evidence that results were robust to the presence of missing outcome data?	PN	No sensitivity analysis but the authors treated "drop-out" as non abstinent
	**Risk of bias judgement**	**Low**	Although there were some missing data, results still stood in consideration of balanced missing data. "Low" risk of bias in this domain was rated.
Bias in measurement of the outcome	4.1 Were outcome assessors aware of the intervention received by study participants?	N	Double-blind design
	4.2 If Y/PY/NI to 4.1: Was the assessment of the outcome likely to be influenced by knowledge of intervention received?	NA	
	**Risk of bias judgement**	**Low**	Double-blind design put this domain as low risk of bias.
	Are the reported outcome data likely to have been selected, on the basis of the results, from…		

Bias in selection of the reported result	5.1. ... multiple outcome measurements (e.g. scales, definitions, time points) within the outcome domain?	PN	
	5.2 ... multiple analyses of the data?	PN	
	**Risk of bias judgement**	**Low**	No protocol was found but nature of outcome, abstinence, presents low risk of selected reported results and the authors described the method section clearly. Thus, we rated "Low" risk of bias in this domain.
**Overall bias**	**Risk of bias judgement**	**Low**	Low risk of bias

**Reference**	Ulrichsen 2010	**Aim**	assignment to intervention (the 'intention-to-treat' effect)	**Source**	Journal article(s) with results of the trial
**Outcome**	Abstinence (6 months)	**Results**	4/20 (TAU) vs 5/19 (Disulfiram 229 mg)		

Domain	Signalling question	Response	Comments
**Bias arising from the randomization process**	1.1 Was the allocation sequence random?	Y	"A nurse from the department not participating in the study performed randomization using sealed envelopes containing a label for one of the two treatment conditions."<br>"In order to ensure a balanced number of patients in each group the envelopes were arranged in blocks of 6–10. In each block, half were labelled "disulfiram" and half were labelled "control".<br>"The envelopes were arranged and sealed by a secretary not participating in the study who was instructed to arrange the labels in a random order."
	1.2 Was the allocation sequence concealed until participants were recruited and assigned to interventions?	Y	
	1.3 Were there baseline imbalances that suggest a problem with the randomization process?	N	No significant difference between groups (Table 1)
	**Risk of bias judgement**	**Low**	This study employed adequate randomisation methods in the trial and represents "Low" risk of bias in the randomisation process.
**Bias due to deviations from intended interventions**	2.1 Were participants aware of their assigned intervention during the trial?	Y	open-trial
	2.2 Were carers and trial personnel aware of participants' assigned intervention during the trial?	Y	
	2.3 If Y/PY/NI to 2.1 or 2.2: Were there deviations from the intended intervention beyond what would be expected in usual practice?	NI	
	2.4 If Y/PY to 2.3: Were these deviations from intended intervention unbalanced between groups and likely to have affected the outcome?	NA	
	2.5 If N/PN/NI to 2.4: Were these deviations likely to have affected the outcome?	N	"All data were used on an intention-to-treat basis."
	2.6 If Y/PY/NI to 2.5: Was there potential for a substantial impact (on the estimated effect of intervention) of analysing participants in the wrong group?	NA	
	**Risk of bias judgement**	**Some concerns**	The open label design in this trial might prompt deviations from the intended interventions, contributing to "some concerns" in this domain.
**Bias due to missing outcome data**	3.1 Were outcome data available for all, or nearly all, participants randomized?	N	"Whereas 17 patients in the disulfi ram group started group treatment, only seven completed it (41%). In the control group fewer started, i.e. 15, but 10 of these completed group treatment (67%)."
	3.2 If N/PN/NI to 3.1: Are the proportions of missing outcome data and reasons for missing outcome data similar across intervention groups?	NI	There was no statistically significant difference in the proportions of missing outcomes. No reasons were given.
	3.3 If N/PN/NI to 3.1: Is there evidence that results were robust to the presence of missing outcome data?	PN	No information regarding sensitivity analysis but the authors treated all drop-outs as relapsed.
	**Risk of bias judgement**	**Some concerns**	High porportion of missing data and no detailed reasons for missing data put "some concerns" in this domain.
**Bias in measurement of the outcome**	4.1 Were outcome assessors aware of the intervention received by study participants?	PY	No information regarding the method of assessing abstinence but since this is an open-trial, patients were aware of the intervention received.
	4.2 If Y/PY/NI to 4.1: Was the assessment of the outcome likely to be influenced by knowledge of intervention received?	PY	Outcomes can be influenced by the knowledge of interventions
	**Risk of bias judgement**	**High**	Open-label design and outcome can be influenced by the knowledge of interventions - "High" was rated.
**Bias in selection of the reported result**	Are the reported outcome data likely to have been selected, on the basis of the results, from…		
	5.1. ... multiple outcome measurements (e.g. scales, definitions, time points) within the outcome domain?	PN	
	5.2 ... multiple analyses of the data?	PN	
	**Risk of bias judgement**	**Low**	No protocol was found but nature of outcome, abstinence, presents low risk of selected reported results and the authors described the method section clearly. Thus, we rated "Low" risk of bias in this domain.
**Overall bias**	**Risk of bias judgement**	**High**	High in overall bias due to open-label design and missing data without details.

**Reference**	Volpicelli 1997	**Aim**	assignment to intervention (the 'intention-to-treat' effect)	**Source**	Journal article(s) with results of the trial
**Outcome**	Abstinence (12 weeks)	**Results**	21/48 (Naltrexone 50 mg) vs 17/49 (Placebo)		

Domain	Signalling question	Response	Comments
**Bias arising from the randomization process**	1.1 Was the allocation sequence random?	Y	"...computer randomized block of 20 subjects.."
	1.2 Was the allocation sequence concealed until participants were recruited and assigned to interventions?	NI	
	1.3 Were there baseline imbalances that suggest a problem with the randomization process?	N	"The baseline sociodemographics of the 2 study groups of the sample did not differ (Table 1)."
	**Risk of bias judgement**	**Some concerns**	No details were given regarding allocation concealment, contributed to "some concerns" in this domain.
**Bias due to deviations from intended interventions**	2.1 Were participants aware of their assigned intervention during the trial?	PN	Only stated "double-blind"
	2.2 Were carers and trial personnel aware of participants' assigned intervention during the trial?	PN	
	2.3 If Y/PY/NI to 2.1 or 2.2: Were there deviations from the intended intervention beyond what would be expected in usual practice?	NA	
	2.4 If Y/PY to 2.3: Were these deviations from intended intervention unbalanced between groups and likely to have affected the outcome?	NA	
	2.5 If N/PN/NI to 2.4: Were these deviations likely to have affected the outcome?	N	ITT analysis
	2.6 If Y/PY/NI to 2.5: Was there potential for a substantial impact (on the estimated effect of intervention) of analysing participants in the wrong group?	NA	
	**Risk of bias judgement**	**Low**	Double-blind design and ITT analysis employed in this trial put "Low" risk of bias in deviation from intended interventions.

Bias due to missing outcome data	3.1 Were outcome data available for all, or nearly all, participants randomized?	N	"Of the 49 subjects in the placebo group, 36 (73%) completed the treatment protocol, compared with 35 (73%) of 48 naltrexone-treated subjects."
	3.2 If N/PN/NI to 3.1: Are the proportions of missing outcome data and reasons for missing outcome data similar across intervention groups?	NI	The proportions of drop-outs were similar but no reasons by group
	3.3 If N/PN/NI to 3.1: Is there evidence that results were robust to the presence of missing outcome data?	PN	No mention of how missing data was dealt with, and no sensitivity analysis.
	**Risk of bias judgement**	**Some concerns**	Some concerns due to missing data and no details.
Bias in measurement of the outcome	4.1 Were outcome assessors aware of the intervention received by study participants?	PN	Double-blind design
	4.2 If Y/PY/NI to 4.1: Was the assessment of the outcome likely to be influenced by knowledge of intervention received?	NA	
	**Risk of bias judgement**	**Low**	Double-blind design and self-reporting outcome (confirmed by relatives and biochemistry results) put this domain as low risk of bias.
Bias in selection of the reported result	Are the reported outcome data likely to have been selected, on the basis of the results, from…		
	5.1. ... multiple outcome measurements (e.g. scales, definitions, time points) within the outcome domain?	PN	
	5.2 ... multiple analyses of the data?	PN	
	**Risk of bias judgement**	**Low**	No protocol was found but nature of outcome, abstinence, presents low risk of selected reported results. Thus, we rated "Low" risk of bias in this domain.
**Overall bias**	**Risk of bias judgement**	**Some concerns**	Some concerns due to lack of details in randomisation process and missing data

**Reference**	Wetzel 2004	**Aim**	assignment to intervention (the 'intention-to-treat' effect)	**Source**	Journal article(s) with results of the trial
**Outcome**	Abstinence (52 weeks)	**Results**	12/53 (Nefazodone 600 mg + CBT) vs 9/50 (Nefazodone 600 mg + GC) vs 12/50 (Placebo + CBT) vs 13/47 (Placebo + GC)		

Domain	Signalling question	Response	Comments
Bias arising from the randomization process	1.1 Was the allocation sequence random?	Y	"Randomization followed a centralized assignment procedure independent of responsible or treating clinicians and hospitals."
	1.2 Was the allocation sequence concealed until participants were recruited and assigned to interventions?	PY	
	1.3 Were there baseline imbalances that suggest a problem with the randomization process?	N	
	**Risk of bias judgement**	**Low**	This study employed adequate randomisation methods in the trial and represents "Low" risk of bias in the randomisation process.
Bias due to deviations from intended interventions	2.1 Were participants aware of their assigned intervention during the trial?	PN	"double-blind"<br>"Both study medications contained riboflavin to control for medication compliance by urine samples without breaking the blind."
	2.2 Were carers and trial personnel aware of participants' assigned intervention during the trial?	PN	
	2.3 If Y/PY/NI to 2.1 or 2.2: Were there deviations from the intended intervention beyond what would be expected in usual practice?	NA	
	2.4 If Y/PY to 2.3: Were these deviations from intended intervention unbalanced between groups and likely to have affected the outcome?	NA	
	2.5 If N/PN/NI to 2.4: Were these deviations likely to have affected the outcome?	N	"All results reported are based on ITT statistics", no evidence of switches etc.
	2.6 If Y/PY/NI to 2.5: Was there potential for a substantial impact (on the estimated effect of intervention) of analysing participants in the wrong group?	NA	
	**Risk of bias judgement**	**Low**	Double-blind design and ITT analysis employed in this trial put "Low" risk of bias in deviation from intended interventions.
Bias due to missing outcome data	3.1 Were outcome data available for all, or nearly all, participants randomized?	N	High proportion of drop-outs (Figure 1).
	3.2 If N/PN/NI to 3.1: Are the proportions of missing outcome data and reasons for missing outcome data similar across intervention groups?	NI	% similar between arms. No breakdown of reasons given.
	3.3 If N/PN/NI to 3.1: Is there evidence that results were robust to the presence of missing outcome data?	N	No sensitivity anaylsis.
	**Risk of bias judgement**	**Some concerns**	High porportion of missing data and no detailed reasons for missing data put "some concerns" in this domain.
Bias in measurement of the outcome	4.1 Were outcome assessors aware of the intervention received by study participants?	N	double-blind
	4.2 If Y/PY/NI to 4.1: Was the assessment of the outcome likely to be influenced by knowledge of intervention received?	NA	
	**Risk of bias judgement**	**Low**	Double-blind design and self-reporting outcome put this domain as low risk of bias.
Bias in selection of the reported result	Are the reported outcome data likely to have been selected, on the basis of the results, from…		
	5.1. ... multiple outcome measurements (e.g. scales, definitions, time points) within the outcome domain?	PN	
	5.2 ... multiple analyses of the data?	PN	
	**Risk of bias judgement**	**Low**	No protocol was found but nature of outcome, abstinence, presents low risk of selected reported results and the authors described the method section clearly. Thus, we rated "Low" risk of bias in this domain.
**Overall bias**	**Risk of bias judgement**	**Some concerns**	Some concerns due to lack of details in missing data.

**Reference**	Whitworth 1996	**Aim**	assignment to intervention (the 'intention-to-treat' effect)	**Source**	Journal article(s) with results of the trial
**Outcome**	Abstinence (360 days)	**Results**	41/224 (Acamprosate 1998 mg) vs 16/224 (Placebo)		

Domain	Signalling question	Response	Comments
Bias arising from the randomization process	1.1 Was the allocation sequence random?	Y	"Rnadomisation was by computer-generated list organised in blocks of eight."<br>"Allocation codes were provided in sealed envelopes for each patient."
	1.2 Was the allocation sequence concealed until participants were recruited and assigned to interventions?	Y	
	1.3 Were there baseline imbalances that suggest a problem with the randomization process?	PN	"The groups were well matched in terms of demographic and alcoholrelated baseline variables on the day of selection and on day 0 (table 1)"
	**Risk of bias judgement**	**Low**	This study employed adequate randomisation methods in the trial and represents "Low" risk of bias in the randomisation process.
	2.1 Were participants aware of their assigned intervention during the trial?	PN	"The duration of double-blind treatment was 360 days

Bias due to deviations from intended interventions	2.2 Were carers and trial personnel aware of participants' assigned intervention during the trial?	PN	"The duration of double-blind treatment was 360 days
	2.3 If Y/PY/NI to 2.1 or 2.2: Were there deviations from the intended intervention beyond what would be expected in usual practice?	NA	
	2.4 If Y/PY to 2.3: Were these deviations from intended intervention unbalanced between groups and likely to have affected the outcome?	NA	
	2.5 If N/PN/NI to 2.4: Were these deviations likely to have affected the outcome?	N	Modified ITT used and no evidence of patient switching
	2.6 If Y/PY/NI to 2.5: Was there potential for a substantial impact (on the estimated effect of intervention) of analysing participants in the wrong group?	NA	
	**Risk of bias judgement**	**Low**	Double-blind design and ITT analysis employed in this trial put "Low" risk of bias in deviation from intended interventions.
**Bias due to missing outcome data**	3.1 Were outcome data available for all, or nearly all, participants randomized?	PN	At 12 months, only 40% of patients remaining. Additionally, 7 patients exlcuded under mITT protocol
	3.2 If N/PN/NI to 3.1: Are the proportions of missing outcome data and reasons for missing outcome data similar across intervention groups?	PY	%'s and reasons for missingness is similar between groups.
	3.3 If N/PN/NI to 3.1: Is there evidence that results were robust to the presence of missing outcome data?	N	No sensitivity analysis
	**Risk of bias judgement**	**Low**	Although there were some missing data, results still stood in consideration of balanced missing data between groups. "Low" risk of bias in this domain was rated.
**Bias in measurement of the outcome**	4.1 Were outcome assessors aware of the intervention received by study participants?	PN	"double-blind"
	4.2 If Y/PY/NI to 4.1: Was the assessment of the outcome likely to be influenced by knowledge of intervention received?	NA	
	**Risk of bias judgement**	**Low**	Double-blind design and self-reporting put this domain as low risk of bias.
**Bias in selection of the reported result**	Are the reported outcome data likely to have been selected, on the basis of the results, from…		
	5.1. ... multiple outcome measurements (e.g. scales, definitions, time points) within the outcome domain?	PN	
	5.2 ... multiple analyses of the data?	N	
	**Risk of bias judgement**	**Low**	No protocol was found but nature of outcome, abstinence, presents low risk of selected reported results and the authors described the method section clearly. Thus, we rated "Low" risk of bias in this domain.
**Overall bias**	**Risk of bias judgement**	**Low**	Low risk of bias

**Reference**	Wiesbeck 2001	**Aim**	assignment to intervention (the 'intention-to-treat' effect)	**Source**	Journal article(s) with results of the trial
**Outcome**	Abstinence (12 months)	**Results**	34/142 (Flupenthixol 10 mg) vs 58/139 (Placebo)		

Domain	Signalling question	Response	Comments
**Bias arising from the randomization process**	1.1 Was the allocation sequence random?	NI	only stated "random"
	1.2 Was the allocation sequence concealed until participants were recruited and assigned to interventions?	NI	
	1.3 Were there baseline imbalances that suggest a problem with the randomization process?	N	"...groups were well matched" Table 1
	**Risk of bias judgement**	**Some concerns**	No details were given regarding randomisation process, contributed to "some concerns" in this domain.
**Bias due to deviations from intended interventions**	2.1 Were participants aware of their assigned intervention during the trial?	PN	"double-blind"
	2.2 Were carers and trial personnel aware of participants' assigned intervention during the trial?	PN	
	2.3 If Y/PY/NI to 2.1 or 2.2: Were there deviations from the intended intervention beyond what would be expected in usual practice?	NA	
	2.4 If Y/PY to 2.3: Were these deviations from intended intervention unbalanced between groups and likely to have affected the outcome?	NA	
	2.5 If N/PN/NI to 2.4: Were these deviations likely to have affected the outcome?	N	No evidence of patients being analysed in wrong group. mITT used.
	2.6 If Y/PY/NI to 2.5: Was there potential for a substantial impact (on the estimated effect of intervention) of analysing participants in the wrong group?	NA	
	**Risk of bias judgement**	**Low**	Double-blind design and mITT analysis employed in this trial put "Low" risk of bias in deviation from intended interventions.
**Bias due to missing outcome data**	3.1 Were outcome data available for all, or nearly all, participants randomized?	N	"Of 281 patients enrolled, 91 (32.4%) completed the trial (6 months treatment, 6 months follow-up)"
	3.2 If N/PN/NI to 3.1: Are the proportions of missing outcome data and reasons for missing outcome data similar across intervention groups?	N	More patients in FLUX group dropped out due to severe relapse.
	3.3 If N/PN/NI to 3.1: Is there evidence that results were robust to the presence of missing outcome data?	N	No sensitivity analyses
	**Risk of bias judgement**	**Some concerns**	High porportion of missing data and imbalanced missing data without sensitivity analysis put "high" risk of bias in this domain.
**Bias in measurement of the outcome**	4.1 Were outcome assessors aware of the intervention received by study participants?	PN	"double-blind"; "Outcome variables were based on absolute abstinence, which was defined as no alcohol consumption. To be considered abstinent, the patient's self-report had to be in accordance with the investigator's clinical assessment and the result of a breath analyser."
	4.2 If Y/PY/NI to 4.1: Was the assessment of the outcome likely to be influenced by knowledge of intervention received?	NA	
	**Risk of bias judgement**	**Low**	Double-blind design and self-reporting outcome (confirmed by biochemistry results) put this domain as low risk of bias.
**Bias in selection of the reported result**	Are the reported outcome data likely to have been selected, on the basis of the results, from…		
	5.1. ... multiple outcome measurements (e.g. scales, definitions, time points) within the outcome domain?	N	
	5.2 ... multiple analyses of the data?	N	
	**Risk of bias judgement**	**Low**	No protocol was found but nature of outcome, abstinence, presents low risk of selected reported results and the authors described the method section clearly. Thus, we rated "Low" risk of bias in this domain.
**Overall bias**	**Risk of bias judgement**	**Some concerns**	Lack of detailed methods for the randomisation process and substantial difference in the reasons for missing data, together, these contributed to "some concerns" in overall bias for this trial.

**Reference**	Florez 2010	**Aim**	assignment to intervention (the 'intention-to-treat' effect)	**Source**	Journal article(s) with results of the trial

Outcome	Abstinence (6 months)	Results	38/91 (Naltrexone 50 mg) vs 43/91 (Topiramate 200 mg)

Domain	Signalling question	Response	Comments
Bias arising from the randomization process	1.1 Was the allocation sequence random?	NI	Only stated "random"
	1.2 Was the allocation sequence concealed until participants were recruited and assigned to interventions?	NI	
	1.3 Were there baseline imbalances that suggest a problem with the randomization process?	PN	"At baseline, the 2 treatment groups were homogeneous with respect to sociodemographic, clinical, and alcoholrelated variables (tables 1–3)."
	**Risk of bias judgement**	**Some concerns**	No details were given regarding randomisation process, contributed to "some concerns" in this domain.
Bias due to deviations from intended interventions	2.1 Were participants aware of their assigned intervention during the trial?	Y	naturalistic design
	2.2 Were carers and trial personnel aware of participants' assigned intervention during the trial?	Y	
	2.3 If Y/PY/NI to 2.1 or 2.2: Were there deviations from the intended intervention beyond what would be expected in usual practice?	NI	
	2.4 If Y/PY to 2.3: Were these deviations from intended intervention unbalanced between groups and likely to have affected the outcome?	NA	
	2.5 If N/PN/NI to 2.4: Were these deviations likely to have affected the outcome?	N	No evidence of swapping
	2.6 If Y/PY/NI to 2.5: Was there potential for a substantial impact (on the estimated effect of intervention) of analysing participants in the wrong group?	NA	
	**Risk of bias judgement**	**Some concerns**	The open label design in this trial might prompt deviations from the intended interventions, contributing to "some concerns" in this domain.
Bias due to missing outcome data	3.1 Were outcome data available for all, or nearly all, participants randomized?	Y	"The 2 treatments did not differ with respect to treatment adherence, which was high in both groups." >90% for both at 3 months
	3.2 If N/PN/NI to 3.1: Are the proportions of missing outcome data and reasons for missing outcome data similar across intervention groups?	NA	
	3.3 If N/PN/NI to 3.1: Is there evidence that results were robust to the presence of missing outcome data?	NA	
	**Risk of bias judgement**	**Low**	The proportions of missing data were small, suggesting "Low" risk of bias.
Bias in measurement of the outcome	4.1 Were outcome assessors aware of the intervention received by study participants?	Y	"Alcohol intake was assessed at each treatment session. Both the patient and the significant other were interviewed and the higher reported intake level was used."
	4.2 If Y/PY/NI to 4.1: Was the assessment of the outcome likely to be influenced by knowledge of intervention received?	PY	Outcomes might be influenced by the knowledge of interventions.
	**Risk of bias judgement**	**High**	High risk of bias due to outcomes can be influenced by the knowledge of interventions.
Bias in selection of the reported result	Are the reported outcome data likely to have been selected, on the basis of the results, from…		
	5.1. ... multiple outcome measurements (e.g. scales, definitions, time points) within the outcome domain?	PN	"Alcohol intake was assessed at each treatment session. Both the patient and the significant other were interviewed and the higher reported intake level was used."
	5.2 ... multiple analyses of the data?	PN	
	**Risk of bias judgement**	**Low**	No protocol was found but nature of outcome, abstinence, presents low risk of selected reported results and the authors described the method section clearly. Thus, we rated "Low" risk of bias in this domain.
**Overall bias**	**Risk of bias judgement**	**High**	Lack of details in randomisation and open-label design contributed to "High" risk of bias in this trial.

Reference	GATE 2 study	Aim	assignment to intervention (the 'intention-to-treat' effect)	Source	Conference abstract(s) about the trial; Personal communication with trialist
Outcome	Abstinence (12 months)	Results	63/154 (GHB (44.3-52.5 mg/kg)) vs 48/160 (Placebo)		

Domain	Signalling question	Response	Comments
Bias arising from the randomization process	1.1 Was the allocation sequence random?	NI	
	1.2 Was the allocation sequence concealed until participants were recruited and assigned to interventions?	NI	
	1.3 Were there baseline imbalances that suggest a problem with the randomization process?	NI	
	**Risk of bias judgement**	**Some concerns**	No details were given regarding randomisation process, contributed to "some concerns" in this domain.
Bias due to deviations from intended interventions	2.1 Were participants aware of their assigned intervention during the trial?	PN	"double-blind"
	2.2 Were carers and trial personnel aware of participants' assigned intervention during the trial?	PN	
	2.3 If Y/PY/NI to 2.1 or 2.2: Were there deviations from the intended intervention beyond what would be expected in usual practice?	NA	
	2.4 If Y/PY to 2.3: Were these deviations from intended intervention unbalanced between groups and likely to have affected the outcome?	NA	
	2.5 If N/PN/NI to 2.4: Were these deviations likely to have affected the outcome?	PN	No evidence of switching patients
	2.6 If Y/PY/NI to 2.5: Was there potential for a substantial impact (on the estimated effect of intervention) of analysing participants in the wrong group?	NA	
	**Risk of bias judgement**	**Low**	Double-blind design employed in this trial put "Low" risk of bias in deviation from intended interventions.
Bias due to missing outcome data	3.1 Were outcome data available for all, or nearly all, participants randomized?	N	Very a few participants completed the trial -48% and 36% of patients left at 6 months.
	3.2 If N/PN/NI to 3.1: Are the proportions of missing outcome data and reasons for missing outcome data similar across intervention groups?	PN	No reasons were provided
	3.3 If N/PN/NI to 3.1: Is there evidence that results were robust to the presence of missing outcome data?	PN	No sensitivity analyses
	**Risk of bias judgement**	**High**	High porportion of missing data and imbalanced missing data without sensitivity analysis put "high" risk of bias in this domain.
Bias in measurement of the outcome	4.1 Were outcome assessors aware of the intervention received by study participants?	PN	"double-blind"
	4.2 If Y/PY/NI to 4.1: Was the assessment of the outcome likely to be influenced by knowledge of intervention received?	NA	
	**Risk of bias judgement**	**Low**	Double-blind design and self-reporting outcome put this domain as low risk of bias.
Bias in selection of the reported result	Are the reported outcome data likely to have been selected, on the basis of the results, from…		
	5.1. ... multiple outcome measurements (e.g. scales, definitions, time points) within the outcome domain?	NI	
	5.2 ... multiple analyses of the data?	NI	

	Risk of bias judgement	Some concerns	No full study report put this domain as "some concerns"
**Overall bias**	**Risk of bias judgement**	**High**	"High" risk of bias due to lack of full reports, details of randomisation process and missing data.

**Reference**	Barrias 1997	**Aim**	assignment to intervention (the 'intention-to-treat' effect)	**Source**	Journal article(s) with results of the trial
**Outcome**	Abstinence (360 days)	**Results**	31/152 (Placebo) vs 76/172 (Acamprosate 1998 mg)		

Domain	Signalling question	Response	Comments
**Bias arising from the randomization process**	1.1 Was the allocation sequence random?	NI	Only stated "random" but no methods were mentioned
	1.2 Was the allocation sequence concealed until participants were recruited and assigned to interventions?	NI	
	1.3 Were there baseline imbalances that suggest a problem with the randomization process?	N	No significant difference between groups (Table 3)
	**Risk of bias judgement**	**Some concerns**	No details were given regarding randomisation process, contributed to "some concerns" in this domain.
**Bias due to deviations from intended interventions**	2.1 Were participants aware of their assigned intervention during the trial?	PN	"double-blind"
	2.2 Were carers and trial personnel aware of participants' assigned intervention during the trial?	PN	
	2.3 If Y/PY/NI to 2.1 or 2.2: Were there deviations from the intended intervention beyond what would be expected in usual practice?	NA	
	2.4 If Y/PY to 2.3: Were these deviations from intended intervention unbalanced between groups and likely to have affected the outcome?	NA	
	2.5 If N/PN/NI to 2.4: Were these deviations likely to have affected the outcome?	N	No evidence of switching
	2.6 If Y/PY/NI to 2.5: Was there potential for a substantial impact (on the estimated effect of intervention) of analysing participants in the wrong group?	NA	
	**Risk of bias judgement**	**Low**	Double-blind design and ITT analysis employed in this trial put "Low" risk of bias in deviation from intended interventions.
**Bias due to missing outcome data**	3.1 Were outcome data available for all, or nearly all, participants randomized?	N	45% missing from placebo and 43% missing from ACP over course of entire study. No breakdown by time point.
	3.2 If N/PN/NI to 3.1: Are the proportions of missing outcome data and reasons for missing outcome data similar across intervention groups?	PY	Table 1 and 5, % and reasons seem comparable between treatment arms.
	3.3 If N/PN/NI to 3.1: Is there evidence that results were robust to the presence of missing outcome data?	PN	No senstivity analyses
	**Risk of bias judgement**	**Low**	Although there were some missing data, results still stood in consideration of missing data balanced betwee groups. "Low" risk of bias in this domain was rated.
**Bias in measurement of the outcome**	4.1 Were outcome assessors aware of the intervention received by study participants?	PN	"double-blind"
	4.2 If Y/PY/NI to 4.1: Was the assessment of the outcome likely to be influenced by knowledge of intervention received?	NA	
	**Risk of bias judgement**	**Low**	Double-blind design and self-reporting outcome put this domain as low risk of bias.
**Bias in selection of the reported result**	Are the reported outcome data likely to have been selected, on the basis of the results, from…		
	5.1. ... multiple outcome measurements (e.g. scales, definitions, time points) within the outcome domain?	PN	
	5.2 ... multiple analyses of the data?	PN	
	**Risk of bias judgement**	**Low**	No protocol was found but nature of outcome, abstinence, presents low risk of selected reported results and the authors described the method section clearly. Thus, we rated "Low" risk of bias in this domain.
**Overall bias**	**Risk of bias judgement**	**Some concerns**	Some concerns from lack of randomisation process details.

**Reference**	PREDICT study	**Aim**	assignment to intervention (the 'intention-to-treat' effect)	**Source**	Journal article(s) with results of the trial; Non-commercial trial registry record (e.g. ClinicalTrials.gov record); Research ethics application
**Outcome**	Abstinence (90 days)	**Results**	76/172 (Acamprosate 1998 mg) vs 73/169 (Naltrexone 100mg) vs 41/86 (Placebo)		

Domain	Signalling question	Response	Comments
**Bias arising from the randomization process**	1.1 Was the allocation sequence random?	Y	"...using an imbalanced block-randomization algorithm..."
	1.2 Was the allocation sequence concealed until participants were recruited and assigned to interventions?	PY	"the trial register stated "triple" masking, which suggest possible allocation concealment."
	1.3 Were there baseline imbalances that suggest a problem with the randomization process?	N	No significant difference between groups was found
	**Risk of bias judgement**	**Low**	This study employed adequate randomisation methods in the trial and represents "Low" risk of bias in the randomisation process.
**Bias due to deviations from intended interventions**	2.1 Were participants aware of their assigned intervention during the trial?	PN	"double-blind"
	2.2 Were carers and trial personnel aware of participants' assigned intervention during the trial?	PN	
	2.3 If Y/PY/NI to 2.1 or 2.2: Were there deviations from the intended intervention beyond what would be expected in usual practice?	NA	
	2.4 If Y/PY to 2.3: Were these deviations from intended intervention unbalanced between groups and likely to have affected the outcome?	NA	
	2.5 If N/PN/NI to 2.4: Were these deviations likely to have affected the outcome?	N	No evidence of switches.
	2.6 If Y/PY/NI to 2.5: Was there potential for a substantial impact (on the estimated effect of intervention) of analysing participants in the wrong group?	NA	
	**Risk of bias judgement**	**Low**	Double-blind design and ITT analysis employed in this trial put "Low" risk of bias in deviation from intended interventions.
**Bias due to missing outcome data**	3.1 Were outcome data available for all, or nearly all, participants randomized?	N	There were some drop-outs among groups (more than 10%)
	3.2 If N/PN/NI to 3.1: Are the proportions of missing outcome data and reasons for missing outcome data similar across intervention groups?	PY	The proportions of missing data among groups were similar between groups.
	3.3 If N/PN/NI to 3.1: Is there evidence that results were robust to the presence of missing outcome data?	PN	No sensitivity analysis
	**Risk of bias judgement**	**Low**	Although there were some missing data, results still stood in consideration of balanced missing data among groups. "Low" risk of bias in this domain was rated.

**Bias in measurement of the outcome**	4.1 Were outcome assessors aware of the intervention received by study participants?	N	"Double-blind"
	4.2 If Y/PY/NI to 4.1: Was the assessment of the outcome likely to be influenced by knowledge of intervention received?	NA	
	**Risk of bias judgement**	**Low**	Double-blind design put this domain as low risk of bias.
**Bias in selection of the reported result**	Are the reported outcome data likely to have been selected, on the basis of the results, from…		
	5.1. ... multiple outcome measurements (e.g. scales, definitions, time points) within the outcome domain?	N	Pre-registered on clincial trials.gov, but no definitely given for abstinence, as continuous abstinence not one of the outcomes (data retrieved from personal communication). Based on this, it is unlikely that selective reporting has occured.
	5.2 ... multiple analyses of the data?	N	Pre-registered on clincial trials.gov, but no definitely given for abstinence, as continuous abstinence not one of the outcomes (data retrieved from personal communication). Based on this, it is unlikely that selective reporting has occured.
	**Risk of bias judgement**	**Low**	Protocol was found on ClinicalTrial.gov. No evidence of selection of reporting due to the nature of outcome (abstinence).
**Overall bias**	**Risk of bias judgement**	**Low**	Low risk of bias

**Reference**	Coriale 2019	**Aim**	assignment to intervention (the 'intention-to-treat' effect)	**Source**	Journal article(s) with results of the trial
**Outcome**	Abstinence (365 days)	**Results**	0/43 (CBT) vs 4/47 (MET)		

**Bias arising from the randomization process**	1.1 Was the allocation sequence random?	NI	No information regarding randomisation process
	1.2 Was the allocation sequence concealed until participants were recruited and assigned to interventions?	NI	
	1.3 Were there baseline imbalances that suggest a problem with the randomization process?	PN	No significant difference between groups was found
	**Risk of bias judgement**	**Some concerns**	No details were given regarding randomisation process, contributed to "some concerns" in this domain.
**Bias due to deviations from intended interventions**	2.1 Were participants aware of their assigned intervention during the trial?	Y	Two interventions were different - MET with 3 sessions vs CBT with 5 sessions. No blinding information.
	2.2 Were carers and trial personnel aware of participants' assigned intervention during the trial?	PY	
	2.3 If Y/PY/NI to 2.1 or 2.2: Were there deviations from the intended intervention beyond what would be expected in usual practice?	PN	Although patients with MET had less theraputic sessions, it was expected in usual practice.
	2.4 If Y/PY to 2.3: Were these deviations from intended intervention unbalanced between groups and likely to have affected the outcome?	NA	
	2.5 If N/PN/NI to 2.4: Were these deviations likely to have affected the outcome?	N	No evidence of switches.
	2.6 If Y/PY/NI to 2.5: Was there potential for a substantial impact (on the estimated effect of intervention) of analysing participants in the wrong group?	NA	
	**Risk of bias judgement**	**Low**	Although it was impossible to blind participants in this trial due to nature of interventions employed, this was expected in psychosocial interventions and would not lead to deviation. On the other hand, the authors applied ITT analyses. Together, these contributed to "low" in this domain.
**Bias due to missing outcome data**	3.1 Were outcome data available for all, or nearly all, participants randomized?	N	There were some drop-outs among groups (more than 10%)
	3.2 If N/PN/NI to 3.1: Are the proportions of missing outcome data and reasons for missing outcome data similar across intervention groups?	PN	The proportions of missing data among groups were not similar between groups.
	3.3 If N/PN/NI to 3.1: Is there evidence that results were robust to the presence of missing outcome data?	PN	No sensitivity analysis
	**Risk of bias judgement**	**High**	Disproportional missing outcome data led to high risk of bias.
**Bias in measurement of the outcome**	4.1 Were outcome assessors aware of the intervention received by study participants?	Y	It was impossible to blind outcome assessors (participants).
	4.2 If Y/PY/NI to 4.1: Was the assessment of the outcome likely to be influenced by knowledge of intervention received?	PY	"Blood alcohol levels were measured in all participants by using Alcoscan AL7000" every month. However, the test only provides daily measurement and hard to know the drinking habit throughout the trial period.
	**Risk of bias judgement**	**Some concerns**	Lack of blinding to outcome assessors (participants themselve) and self-reporting outcomes, which put "some concerns" in this domain.
**Bias in selection of the reported result**	Are the reported outcome data likely to have been selected, on the basis of the results, from…		
	5.1. ... multiple outcome measurements (e.g. scales, definitions, time points) within the outcome domain?	PY	Describes itself as a 2y follow-up study but only has data for the first year. There were other follow-up time points at 45 days, 90 days, and 180 days. Continuous abstinence data is not reported for these timepoints.
	5.2 ... multiple analyses of the data?	N	
	**Risk of bias judgement**	**Some concerns**	The authors described itself as a 2y follow-up study but only has data for the first year. However, continuous abstinence data were not reported for all the timepoints, leading to some concerns.
**Overall bias**	**Risk of bias judgement**	**High**	Disporportional dropouts and unclear methods in randomisation process led to high risk of bias.

**Reference**	Jirapramukpitak 2020	**Aim**	assignment to intervention (the 'intention-to-treat' effect)	**Source**	Journal article(s) with results of the trial; Non-commercial trial registry record (e.g. ClinicalTrials.gov record)
**Outcome**	Abstinence (12 weeks)	**Results**	12/80 (Home visits) vs 10/79 (Contingency management)		

**Bias arising from the randomization process**	1.1 Was the allocation sequence random?	Y	"The unit of randomization was the individual participant. Randomization of participants to different arms was carried out at the Coordinating Centre in the Mental Health Clinic of Thammasat University Hospital using a standard randomization table (Pocock, 1983)"
	1.2 Was the allocation sequence concealed until participants were recruited and assigned to interventions?	NI	No adequate information regarding allocation concealment.
	1.3 Were there baseline imbalances that suggest a problem with the randomization process?	PN	No significant difference between groups was found
	**Risk of bias judgement**	**Some concerns**	Adequate information was given for randomisation generation, however, no information of allocation concealment was given - leading to 'some concerns'.
**Bias due to deviations from intended interventions**	2.1 Were participants aware of their assigned intervention during the trial?	PY	No blinding information was provided and it was impossible to blind the participants.
	2.2 Were carers and trial personnel aware of participants' assigned intervention during the trial?	PY	
	2.3 If Y/PY/NI to 2.1 or 2.2: Were there deviations from the intended intervention beyond what would be expected in usual practice?	PN	Not reported but unlikely
	2.4 If Y/PY to 2.3: Were these deviations from intended intervention unbalanced between groups and likely to have affected the outcome?	NA	
	2.5 If N/PN/NI to 2.4: Were these deviations likely to have affected the outcome?	N	No evidence of switches.

	2.6 If Y/PY/NI to 2.5: Was there potential for a substantial impact (on the estimated effect of intervention) of analysing participants in the wrong group?	NA	
	**Risk of bias judgement**	**Low**	Although it was impossible to blind participants in this trial due to nature of interventions employed, this was expected in psychosocial interventions and would not lead to deviation. On the other hand, the authors applied ITT analyses. Together, these contributed to "low" in this domain.
**Bias due to missing outcome data**	3.1 Were outcome data available for all, or nearly all, participants randomized?	Y	Only 1 dropout in each group (1/80 vs 1/42+37)
	3.2 If N/PN/NI to 3.1: Are the proportions of missing outcome data and reasons for missing outcome data similar across intervention groups?	NA	
	3.3 If N/PN/NI to 3.1: Is there evidence that results were robust to the presence of missing outcome data?	NA	
	**Risk of bias judgement**	**Low**	Nearly all patients were followed during the trial.
**Bias in measurement of the outcome**	4.1 Were outcome assessors aware of the intervention received by study participants?	Y	It was impossible to blind outcome assessors.
	4.2 If Y/PY/NI to 4.1: Was the assessment of the outcome likely to be influenced by knowledge of intervention received?	PN	"All negative samples were confirmed by reports of no drinking provided by participants themselves and their informants."
	**Risk of bias judgement**	**Low**	Although there were no blinding to outcome assessors, the authors sought different sources to confirm patients' abstinence status.
**Bias in selection of the reported result**	Are the reported outcome data likely to have been selected, on the basis of the results, from…		
	5.1. ... multiple outcome measurements (e.g. scales, definitions, time points) within the outcome domain?	N	Pre-registered on Thailand National trial register, but no definitely given for abstinence nor patient criteria.
	5.2 ... multiple analyses of the data?	N	
	**Risk of bias judgement**	**Low**	Protocol was found on ClinicalTrial.gov. No evidence of selection of reporting due to the nature of outcome (abstinence).
**Overall bias**	**Risk of bias judgement**	**Some concerns**	Some concerns in overall bias due to no adequate information in allocation concealment.

SUPPLEMENT 7. NETWORK META-ANALYSIS RESULTS

Abstinence up to 12 months

Figure 1. Forest plot

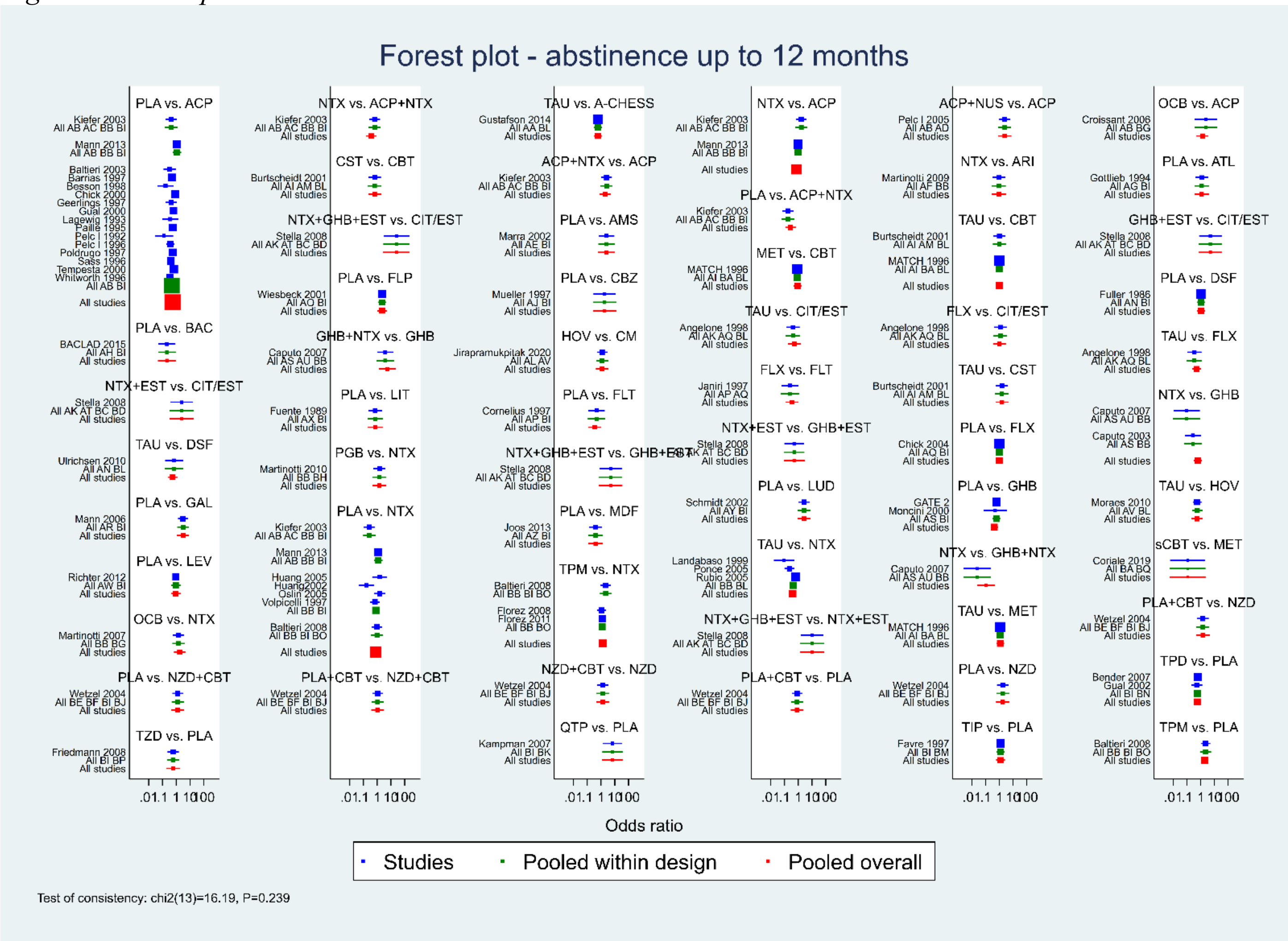

Results are displayed as a point estimate and 95% confidence interval. The blue colour represents direct evidence from each study, grouped by design. The green colour represents pooled treatment effect in each design, estimated by the inconsistency model. The red colour represents overall treatment effect, estimated by the consistency model.

Table 1. Node-splitting (abstinence)

		Direct		Indirect		Difference			
		Coef.	Std. Err.	Coef.	Std. Err.	Coef.	Std. Err.	P>z	tau
PLA	PLA+CBT	.	.	.	.	.	.	.	.
PLA	QTP	.	.	.	.	.	.	.	.
PLA	TIP	.	.	.	.	.	.	.	.
PLA	TPD	.	.	.	.	.	.	.	.
PLA	TPM	0.814736	0.509303	0.540395	0.366589	0.274342	0.629272	0.663	0.299866
PLA	TZD	.	.	.	.	.	.	.	.
A-CHESS	TAU	-0.51627	0.363653	-1.29957	1231.272	0.783306	1231.272	0.999	0.290118
ACP	PLA	-0.65397	0.117392	0.304833	0.629399	-0.9588	0.643923	0.136	0.289871
ACP	ACP+NTX	0.92132	0.547655	0.116536	0.836263	0.804784	0.99201	0.417	0.2962
ACP	ACP+NUS	0.904456	0.569991	-1.34822	1456.669	2.252678	1456.669	0.999	0.290118
ACP	NTX	0.122482	0.284545	-0.6017	0.234572	0.724177	0.36849	0.049	0.256902
ACP	OCB	0.860201	1.002166	0.076789	0.589631	0.783412	1.162756	0.5	0.294118
ACP+NTX	PLA	-1.71765	0.567955	-0.48557	0.809991	-1.23208	1.012692	0.224	0.281689
ACP+NTX	NTX	-0.41837	0.528692	-2.47313	0.84248	2.054762	0.985661	0.037	0.261944
AMS	PLA	.	.	.	.	.	.	.	.
ARI	NTX	-0.08701	0.613236	0.620899	1287.11	-0.70791	1287.11	1	0.290118
ATL	PLA	.	.	.	.	.	.	.	.
BAC	PLA	.	.	.	.	.	.	.	.
CBT	CST	-0.41689	0.611872	-0.46981	1.258119	0.052918	1.376784	0.969	0.304541
CBT	MET	-0.16173	0.361184	-0.10881	1.329843	-0.05292	1.376789	0.969	0.304544
CBT	TAU	-0.0194	0.298849	-1.67797	984.7167	1.658568	984.7168	0.999	0.290118
CBZ	PLA	.	.	.	.	.	.	.	.
CIT/EST	FLX	0.180537	0.607011	-0.95736	1.490609	1.137902	1.634749	0.486	0.291206
CIT/EST	GHB+EST	1.609438	1.008713	-1.32642	1267.588	2.935857	1267.589	0.998	0.290118
CIT/EST	NTX+EST	0.916291	1.029159	-2.01988	1274.543	2.936171	1274.544	0.998	0.290118
CIT/EST	NTX+GHB+EST	3.218876	1.133212	0.28268	1266.704	2.936196	1266.704	0.998	0.290118
CIT/EST	TAU	-0.8873	0.641468	0.250613	1.446674	-1.13792	1.634752	0.486	0.291206
CM	HOV	0.19692	0.544702	-0.08256	1848.571	0.279478	1848.571	1	0.290118
CST	TAU	0.416894	0.611873	0.363975	1.258123	0.052919	1.376789	0.969	0.304544
DSF	PLA	0.035164	0.372964	0.332208	0.88312	-0.29704	0.958646	0.757	0.301316
DSF	TAU	-0.35668	0.821413	-0.65372	0.494244	0.297045	0.958643	0.757	0.301316
FLP	PLA	.	.	.	.	.	.	.	.
FLT	PLA	-0.76029	0.762042	-1.5056	0.861604	0.745314	1.150247	0.517	0.294687
FLT	FLX	-1.41707	0.799525	-0.67175	0.82694	-0.74531	1.150248	0.517	0.294687
FLX	PLA	0.005313	0.360103	-0.13521	0.623805	0.140522	0.720282	0.845	0.300116
FLX	TAU	-1.06784	0.672678	-0.49888	0.464337	-0.56896	0.817377	0.486	0.291206
GAL	PLA	.	.	.	.	.	.	.	.
GHB	PLA	-0.50268	0.340922	-2.01097	0.660777	1.508285	0.743209	0.042	0.271361
GHB	GHB+NTX	1.360977	0.750039	5.387009	2.334613	-4.02603	2.424004	0.097	0.278033
GHB	NTX	-1.62158	0.637137	-0.11329	0.382275	-1.50829	0.743209	0.042	0.271361
GHB+EST	NTX+EST	.	.	.	.	.	.	.	.
GHB+EST	NTX+GHB+EST	.	.	.	.	.	.	.	.
GHB+NTX	NTX	-3.7281	1.190263	0.297948	1.699391	-4.02605	2.424007	0.097	0.278033
HOV	TAU	-0.60305	0.469799	-0.92794	733.2632	0.324883	733.2633	1	0.290118
LEV	PLA	.	.	.	.	.	.	.	.

LIT	PLA	.	.	.	.	.	.	.	.
LUD	PLA	.	.	.	.	.	.	.	.
MDF	PLA	.	.	.	.	.	.	.	.
MET	TAU	0.135269	0.363389	0.188184	1.328039	-0.05292	1.376788	0.969	0.304544
MET	sCBT	-2.19723	1.533743	0.553999	2815.784	-2.75122	2815.783	0.999	0.290118
NTX	PLA	-0.25331	0.204816	-0.46341	0.341154	0.210096	0.403923	0.603	0.293073
NTX	OCB	0.409785	0.556876	1.193194	1.02073	-0.78341	1.162755	0.5	0.294118
NTX	PGB	0.37078	0.602551	-0.64357	1288.186	1.014346	1288.186	0.999	0.290118
NTX	TAU	-0.91979	0.304091	-1.15391	0.605356	0.234114	0.676155	0.729	0.295614
NTX	TPM	0.330564	0.274948	0.243677	0.98146	0.086887	1.025683	0.932	0.303161
NTX+EST	NTX+GHB+EST	.	.	.	.	.	.	.	.
NZD	PLA	.	.	.	.	.	.	.	.
NZD	NZD+CBT	.	.	.	.	.	.	.	.
NZD	PLA+CBT	.	.	.	.	.	.	.	.
NZD+CBT	PLA	.	.	.	.	.	.	.	.
NZD+CBT	PLA+CBT	.	.	.	.	.	.	.	.

Figure 2. Network funnel plot (abstinence)

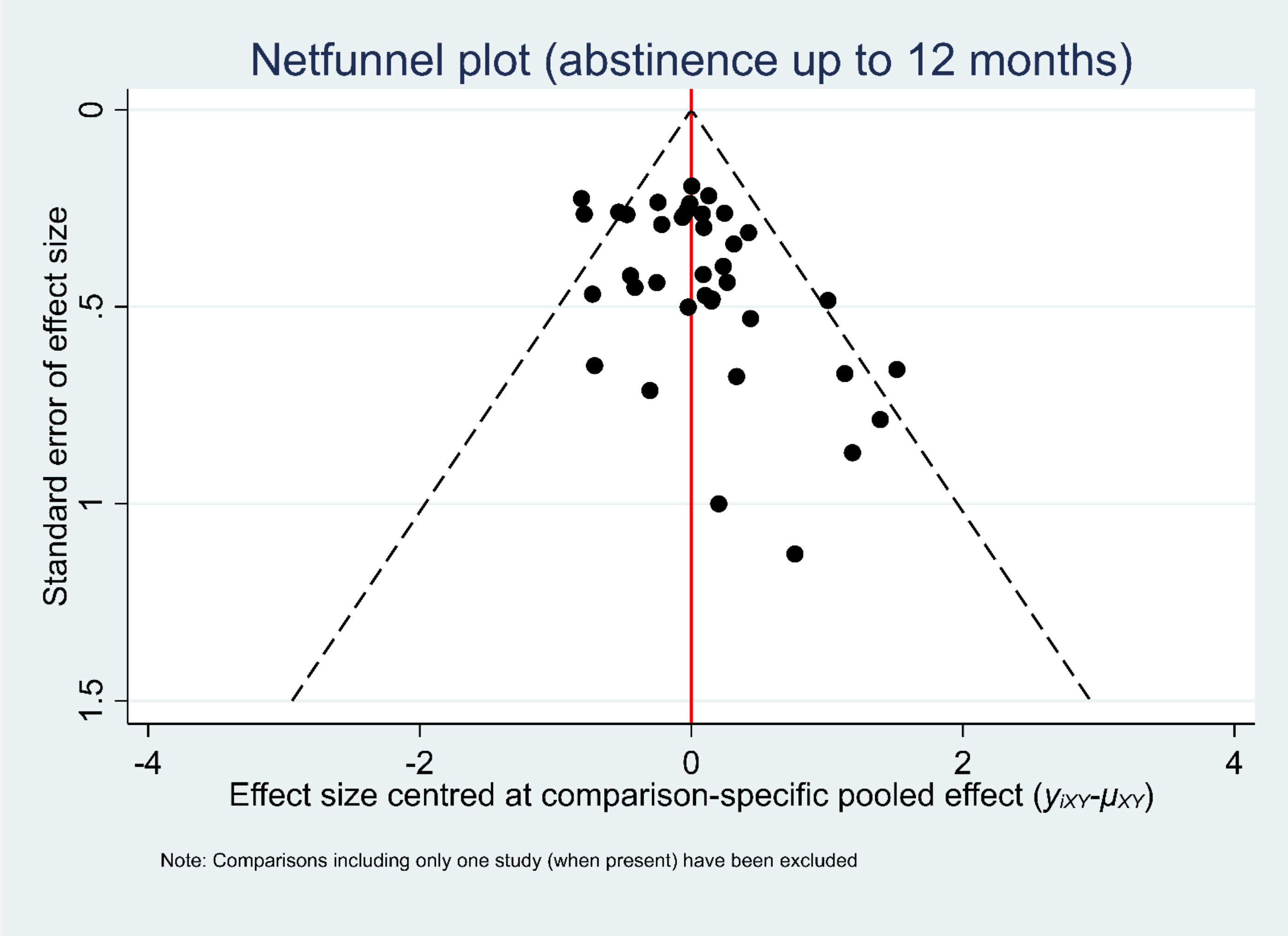

Dropout up to 12 months

Figure 3. Forest plot of drop-outs analysis

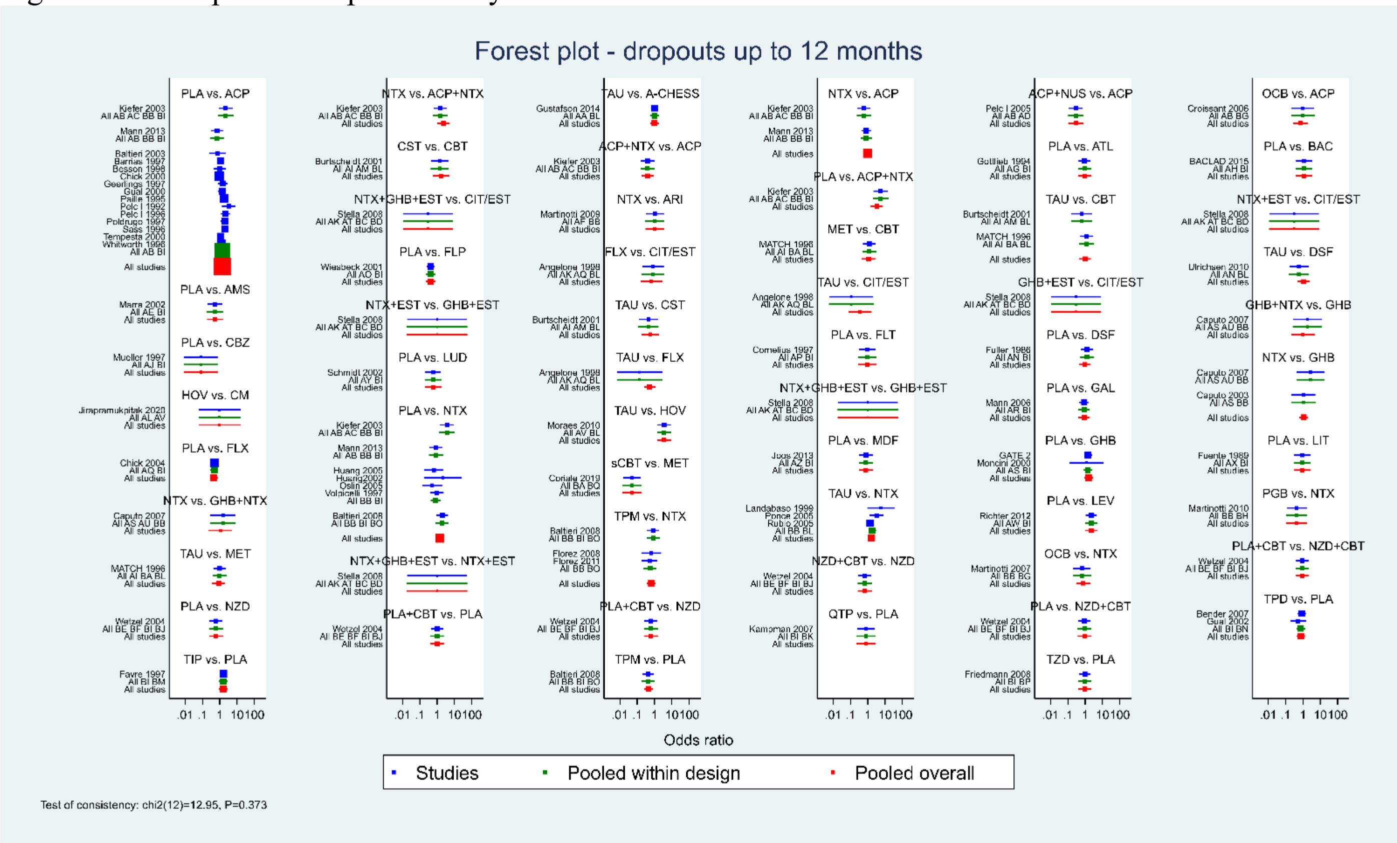

Results are displayed as a point estimate and 95% confidence interval. The blue colour represents direct evidence from each study, grouped by design. The green colour represents pooled treatment effect in each design, estimated by the inconsistency model. The red colour represents overall treatment effect, estimated by the consistency model.

Table 2. Node-splitting (dropouts)

		Direct		Indirect		Difference			
		Coef.	Std. Err	Coef.	Std. Err	Coef.	Std. Err	P>|z|	tau
PLA	PLA+CBT	.	.	.	.	.	.	.	.
PLA	QTP	.	.	.	.	.	.	.	.
PLA	TIP	.	.	.	.	.	.	.	.
PLA	TPD	.	.	.	.	.	.	.	.
PLA	TPM	-0.85717	0.438536	-0.75042	0.449083	-0.10676	0.625541	0.864	0.183587
PLA	TZD	.	.	.	.	.	.	.	.
A-CHESS	TAU	-0.00038	0.311569	0.280841	1492.644	-0.28122	1492.644	1	0.176039
ACP	PLA	0.330842	0.086642	-0.16479	0.625469	0.495634	0.633593	0.434	0.178747
ACP	ACP+NTX	-0.92132	0.495941	-0.80972	0.757769	-0.1116	0.897215	0.901	0.183754
ACP	ACP+NUS	-1.25276	0.512965	0.845478	1452.535	-2.09824	1452.535	0.999	0.176039
ACP	NTX	-0.3302	0.300366	0.135351	0.232708	-0.46555	0.379473	0.22	0.179053
ACP	OCB	-4.61E-11	0.794858	-0.47552	0.642769	0.475522	1.022228	0.642	0.178322
ACP+NTX	PLA	1.717651	0.520764	0.22224	0.739002	1.495412	0.929626	0.108	0.167246
ACP+NTX	NTX	0.418369	0.489406	2.036704	0.804256	-1.61834	0.931979	0.082	0.16917
AMS	PLA	.	.	.	.	.	.	.	.
ARI	NTX	0.04652	0.640139	-0.71762	1474.968	0.764144	1474.968	1	0.176039
ATL	PLA	.	.	.	.	.	.	.	.
BAC	PLA	.	.	.	.	.	.	.	.
CBT	CST	0.348307	0.619736	1.626864	1.57539	-1.27856	1.71627	0.456	0.178168
CBT	MET	0.210046	0.472228	-1.0685	1.639959	1.278549	1.716268	0.456	0.178167
CBT	TAU	-0.02346	0.397696	-1.52566	1013.869	1.502203	1013.869	0.999	0.176039
CBZ	PLA	.	.	.	.	.	.	.	.
CIT/EST	FLX	-0.2156	0.761052	-3.09107	3.070197	2.875473	3.198721	0.369	0.179264
CIT/EST	GHB+EST	-1.18199	1.692517	-2.02125	2695.903	0.839252	2695.903	1	0.176039
CIT/EST	NTX+EST	-1.18199	1.692517	-2.01512	2815.408	0.833126	2815.408	1	0.176039
CIT/EST	NTX+GHB+EST	-1.18199	1.692517	-2.03141	2710.643	0.849418	2710.643	1	0.176044
CIT/EST	TAU	-2.20499	1.5138	0.6705	2.070987	-2.87549	3.198729	0.369	0.17926
CM	HOV	-0.01274	1.43404	-2.4357	5676.054	2.422963	5676.054	1	0.176042
CST	TAU	-0.81093	0.682475	0.467628	1.495579	-1.27856	1.71627	0.456	0.178168
DSF	PLA	0.236114	0.464725	-0.7928	0.744771	1.028915	0.877868	0.241	0.182219
DSF	TAU	-0.539	0.677786	0.48992	0.557905	-1.02892	0.877868	0.241	0.182219
FLP	PLA	.	.	.	.	.	.	.	.
FLT	PLA	.	.	.	.	.	.	.	.
FLX	PLA	-0.72824	0.260791	-2.16598	1.577959	1.437743	1.599365	0.369	0.179264
FLX	TAU	-1.98939	1.550757	-0.55165	0.391318	-1.43775	1.599368	0.369	0.179268
GAL	PLA	.	.	.	.	.	.	.	.
GHB	PLA	0.362111	0.313136	0.867034	0.650325	-0.50492	0.721766	0.484	0.178825
GHB	GHB+NTX	-1.18199	0.587787	0.993152	-1.47256	1.670678	2.060345	2.022035	0.308
GHB	NTX	0.477011	0.624589	-0.02791	0.361717	0.504923	0.721762	0.484	0.178815
GHB+EST	NTX+EST	.	.	.	.	.	.	.	.
GHB+EST	NTX+GHB+EST	.	.	.	.	.	.	.	.
GHB+NTX	NTX	0.430783	0.870471	-1.62956	1.864688	2.060346	2.022035	0.308	0.175488
HOV	TAU	1.280591	0.483943	0.352238	1285.977	0.928353	1285.977	0.999	0.176039
LEV	PLA	.	.	.	.	.	.	.	.

LIT	PLA	.	.	.	.	.	.	.	.
LUD	PLA	.	.	.	.	.	.	.	.
MDF	PLA	.	.	.	.	.	.	.	.
MET	TAU	-0.03339	0.46286	-1.31195	1.647948	1.27856	1.716265	0.456	0.178167
MET	sCBT	-2.99906	0.637673	-0.05343	1332.038	-2.94563	1332.038	0.998	0.176039
NTX	PLA	0.275413	0.212147	0.565414	0.34505	-0.29	0.415252	0.485	0.182508
NTX	OCB	-0.42121	0.614841	0.054309	0.816653	-0.47552	1.022228	0.642	0.178322
NTX	PGB	-0.81093	0.713094	0.666773	1639.611	-1.4777	1639.611	0.999	0.176039
NTX	TAU	0.609427	0.25855	-0.5841	0.751799	1.193525	0.796821	0.134	0.18505
NTX	TPM	-0.38548	0.313498	-0.89778	0.849904	0.512298	0.906585	0.572	0.182587
NTX+EST	NTX+GHB+EST	.	.	.	.	.	.	.	.
NZD	PLA	.	.	.	.	.	.	.	.
NZD	NZD+CBT	.	.	.	.	.	.	.	.
NZD	PLA+CBT	.	.	.	.	.	.	.	.
NZD+CBT	PLA	.	.	.	.	.	.	.	.
NZD+CBT	PLA+CBT	.	.	.	.	.	.	.	.

Figure 4. Network funnel plot (dropout)

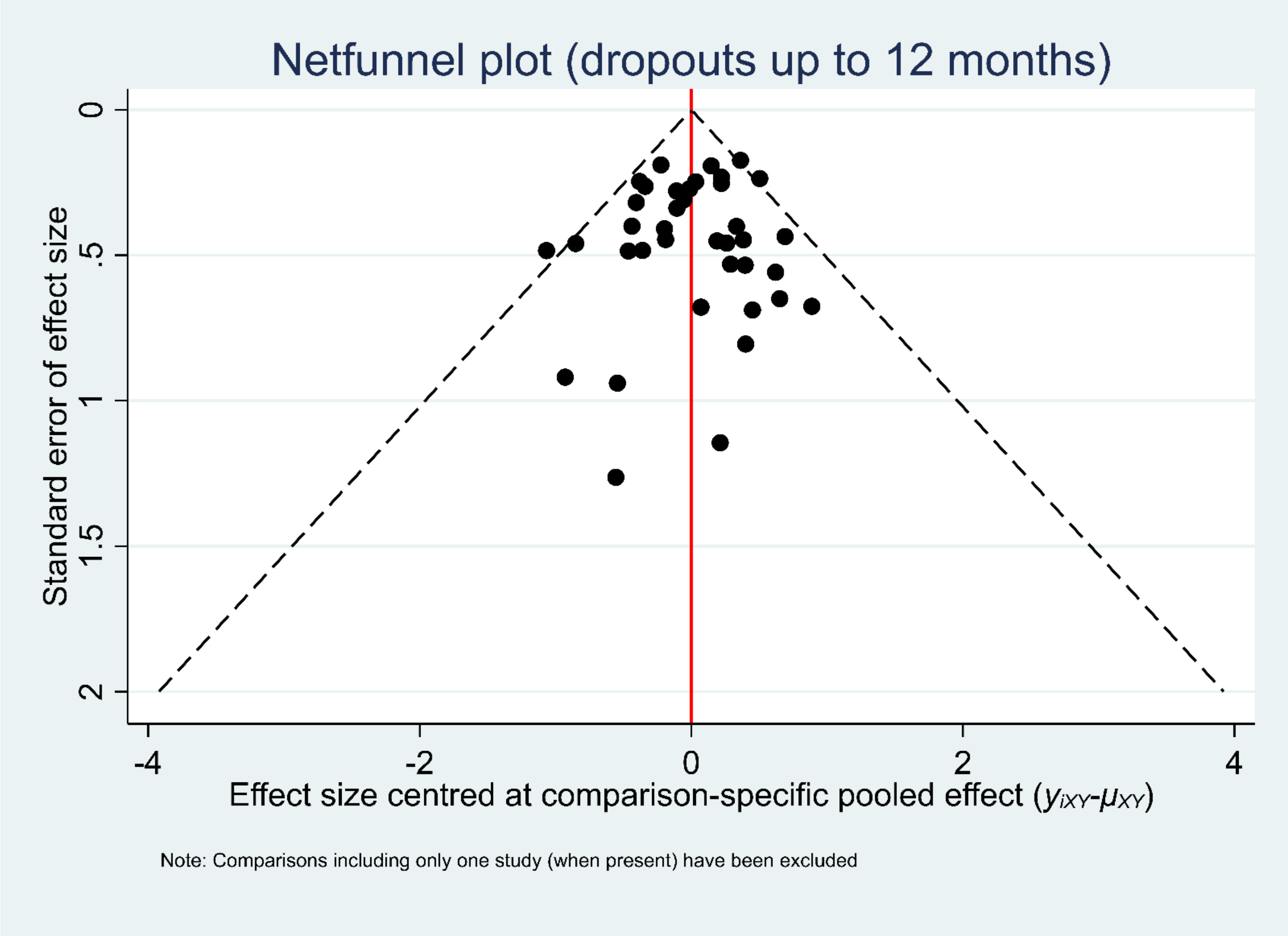

SUPPLEMENT 8. ADDITIONAL ANALYSES

In the additional analyses, we performed meta-regression on the main outcome data. We found no convincing causes of heterogeneity in intervention effects across the five variables we examined (Table 1 and Table 2). The few regression coefficients (3 out of 116) that suggested associations were compatible with chance.

Results of further sensitivity analyses focussing on different time points are presented in Figures S1-S6 for short term, Figures S7-S18 for medium term and Figures S19-S24 for long-term time points. In the medium-term analysis, there was no connected network so two subset network analyses were conducted using TAU (subset 1) and PLA (subset 2) as references.

Results for analyses based on type of interventions appear in Figures S25-S30 for psychotherapy and Figures S31-S36 for pharmacotherapy, with outcomes up to 12 months. Results were broadly in agreement.

The following abbreviations are used in the figures throughout the documents:
A-CHESS = Addiction-Comprehensive Health Enhancement Support System. ACP = acamprosate. ACP+NTX =acamprosate + naltrexone. ACP+NUS = acamprosate + nurse visits. AMS = amisulpride. ARI = aripiprazole. ATL = atenolol. BAC = baclofen. CBT = cognitive behavioural therapy. CBZ = carbamazepine. CIT/EST = citalopram or escitalopram. CST = coping skill training. DSF = disulfiram. FLP = flupenthixol. FLT = fluoxetine. FLX = fluvoixamine.
GAL = galantamine. GHB = sodium oxybate. GHB+EST = sodium oxybate + escitalopram. GHB+NTX = sodium oxybate + naltrexone. HOV = home visit. LEV = levetiracetam. LIT = lithium. LUD = lisuride. MDF = modafinil. MET = motivational enhancement therapy. NTX = naltrexone. NTX+ EST = naltrexone + escitalopram. NTX+EST+GHB = escitalopram + naltrexone + sodium oxybate. NFZ = nefazodone. NFZ+CBT = nefazodone + cognitive behavioural therapy. OCB = oxcarbazepine. PGB = pregabalin. PLA = placebo. PLA+CBT = placebo + cognitive behavioural therapy. QTP = quetiapine. sCBT = short-form CBT. TAU = treatment as usual. TIP = tiapride. TPD = tiapride. TPM = topiramate. TZD = trazodone.

The following captions for all network plots throughout the documents:
The size of circles is proportional to the number of randomised patients and the width of lines is proportional to the number of studies in each direct comparison. The colour of lines represents the overall risk of bias in the majority studies (green: low risk of bias; yellow: some concerns; red: high risk of bias).

NETWORK META-REGRESSION

Table 1. Abstinence up to 12 months

Comparisons (vs PLA)	%Female	Mean age	Detoxification settings	Detoxification methods	Continent of study sites
ACP	-1.27 (-5.14, 2.6)	-0.08 (-0.18, 0.02)	-0.40 (-0.86, 0.06)	-0.20 (-0.74, 0.34)	0.16 (-0.27, 0.58)
CBT	-1.06 (-15.34, 13.22)	0.11 (-2.26, 2.49)			0.00 (-331.51, 331.51)
CIT/EST	75.88 (-21806.16, 21957.92)	0.54 (-122.50, 123.58)	1.3 (-1250.35, 1252.94)		
DSF	-0.58 (-7.07, 5.9)	0.21 (-0.04, 0.45)	0.50 (-1.48, 2.51)	0.26 (-1.89, 2.41)	-0.36 (-2.34, 1.62)
FLT	-0.99 (-10.67, 8.68)	0.14 (-0.11, 0.38)		-0.65 (-2.95, 1.65)	0.73 (-1.62, 3.09)
FLX	7.12 (-18.67, 32.92)	**0.33 (0.04, 0.63)***	0.10 (-1.74, 1.94)	0.94 (-0.99, 2.88)	
GHB	-1.02 (-7.46, 5.42)	0.35 (-0.69, 1.39)	-0.02 (-2.16, 2.13)		
HOV	0.72 (-12057.07, 12058.51)	-0.09 (-66.07, 65.90)	-0.52 (-1722.04, 1721.01)	0.87 (-1789.65, 1791.38)	-0.18 (-758.82, 758.46)
MET	15.55 (-18501.25, 18532.35)	0.22 (-100.08, 100.52)	-1.76 (3199.45, 3195.93)	2.10 (-2724.46, 2728.66)	1.49 (-2450.35, 2453.33)
NTX	0.22 (-3.45, 3.89)	-0.03 (-0.08, 0.02)	0.67 (-0.12, 1.46)	-0.06 (-1.08, 0.96)	0.03 (-0.3, 0.35)
OCB	8.02 (-22.14, 38.18)	-0.9 (-4.57, 2.76)	1.17 (-1.18, 3.52)	-0.86 (-3.33, 1.61)	
TAU	-0.79 (-4.68, 3.10)	**0.17 (0.01, 0.32)***	-0.13 (-1.61, 1.35)	1.25 (-0.31, 2.81)	0.02 (-331.48, 331.53)
TPD	1.11 (-10.42, 12.64)	0.06 (-0.44, 0.55)	-0.13 (-1.45, 1.18)	-0.13 (-1.49, 1.22)	0.13 (-1.32, 1.58)
TPM	-3.03 (-10.71, 4.66)	-0.15 (-0.45, 0.15)	1.06 (-0.19, 2.31)		0.17 (-0.25, 0.60)

Numbers are shown in exponential coefficient (95% confidence intervals).

Table 2. Dropout up to 12 months

Comparisons (vs PLA)	%Female	Mean age	Detoxification settings	Detoxification methods	Continent of study sites
ACP	**-2.82 (-5.62, -0.03)***	0.04 (-0.06, 0.14)	-0.19 (-0.52, 0.15)	0.04 (-0.36, 0.43)	0.21 (-0.19, 0.6)
CBT	6.79 (-9.9, 23.48)	1.15 (-2.21, 4.51)			0.78 (-379.32, 380.88)
CIT/EST	100.71 (-39838, 40039.62)	0.19 (-260.26, 260.63)	2.06 (-2524.57, 2528.68)		
DSF	3.16 (-2.43, 8.75)	-0.06 (-0.31, 0.19)	-1.41 (-3.23, 0.42)	0.51 (-1.4, 2.43)	1.08 (-0.66, 2.82)
FLX	21.20 (-24.45, 66.86)	0.05 (-0.46, 0.55)	1.87 (-1.39, 5.14)	1.00 (-2.25, 4.25)	
GHB	1.17 (-4.61, 6.96)	-0.06 (-1.24, 1.11)	0.20 (-2.14, 2.54)		
HOV	11.88 (-45625.06, 45648.82)	0.31 (-196.64, 197.26)	-0.84 (-3953.41, 3951.74)	0.20 (-5510.28, 5510.67)	0.41 (-1214.97, 1215.80)
MET	6.06 (-14398.85, 14410.97)	0.48 (-122.24, 123.39)	-1.04 (-1290.66, 1288.58)	0.58 (-1296.58, 1296.58)	1.60 (-1336.98, 1340.18)
NTX	-0.49 (-3.79, 2.81)	0.02 (-0.05, 0.08)	-0.76 (-0.56, 0.44)	-0.32 (-1.23, 0.59)	-0.15 (-0.43, 0.13)
OCB	4.30 (-21.27, 29.87)	-0.91 (-4.27, 2.44)	-0.18 (-2.27, 1.90)	-0.67 (-2.82, 1.49)	
TAU	0.40 (-3.08, 3.87)	-0.13 (-0.29, 0.04)	-0.02 (-1.42, 1.38)	-1.32 (-2.73, 0.09)	0.14 (-379.96, 380.24)
TPD	4.24 (-5.97, 14.45)	0.21 (-0.32, 0.74)	-0.50 (-1.78, 0.77)	-0.50 (-1.81, 0.80)	0.50 (-0.80, 1.81)
TPM	-1.88 (-9.51, 5.75)	-0.06 (-0.42, 0.31)	-0.42 (-1.75, 0.92)		-0.01 (-0.42, 0.41)

Numbers are shown in exponential coefficient (95% confidence intervals)

SHORT-TERM

Figure S1. Network plot of abstinence analysis in short-term (3-6 months)

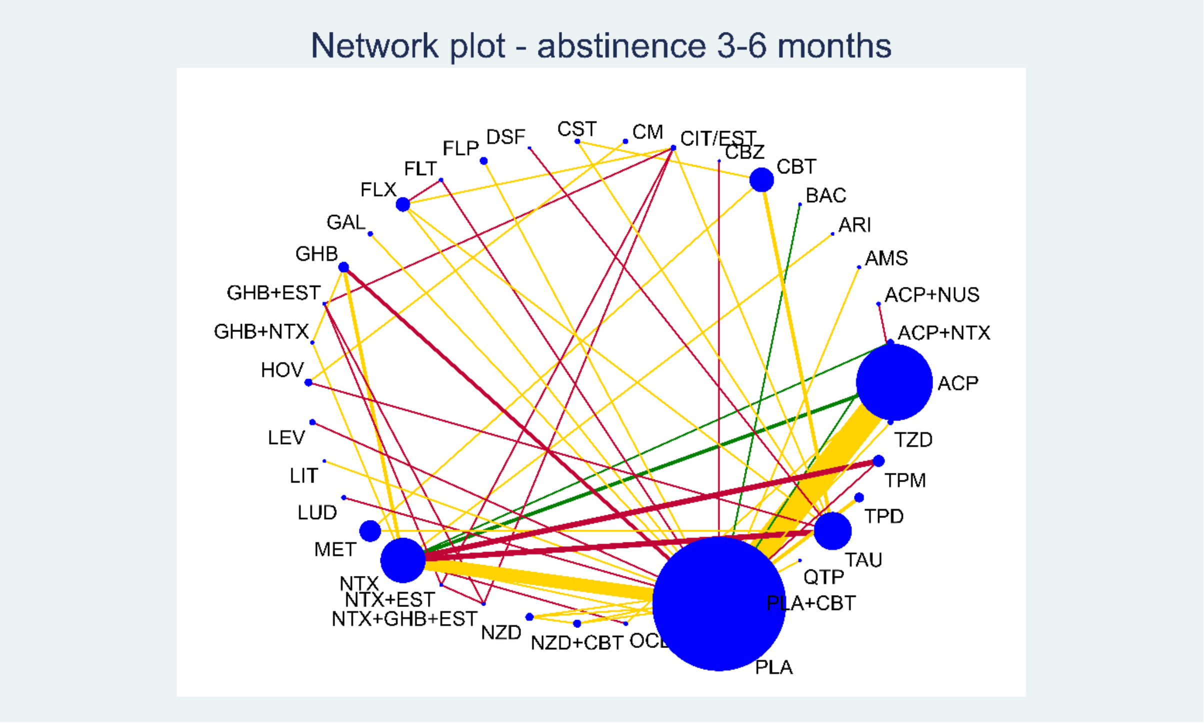

Figure S2. Interval plot of abstinence analysis in short-term (3-6 months)

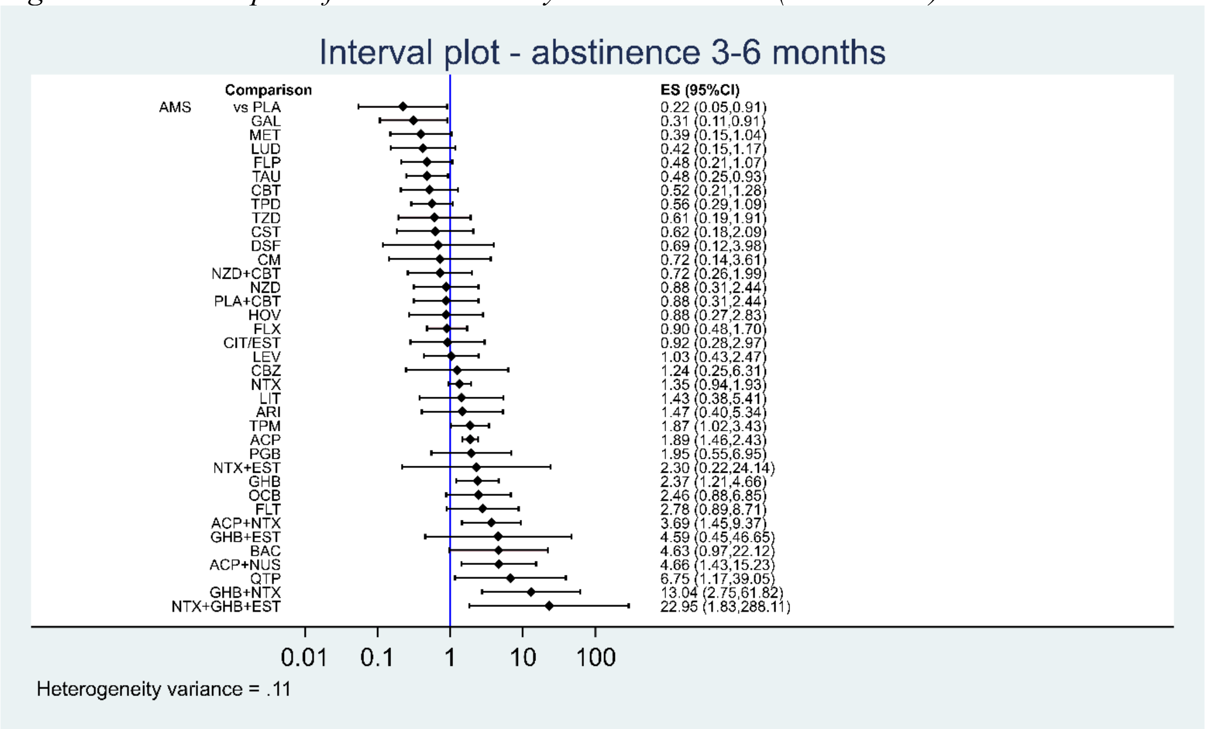

Figure S3. Forest plot of abstinence analysis in short-term (3-6 months)

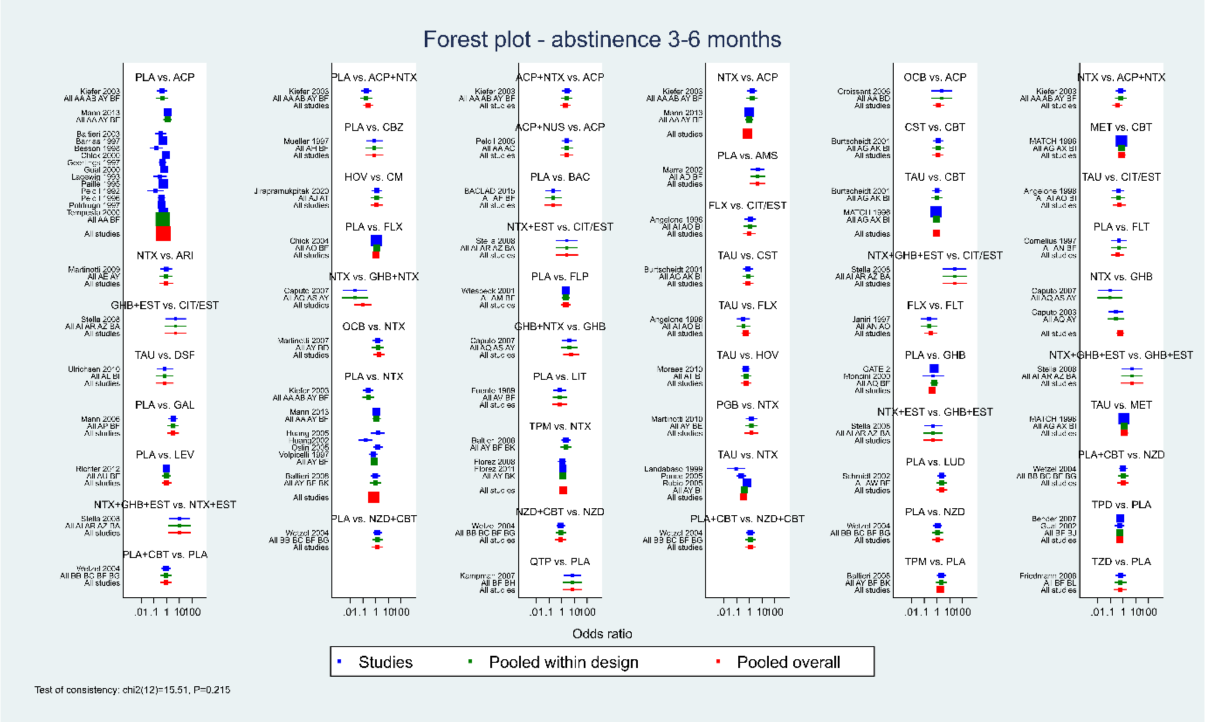

Figure S4. Network plot of drop-out analysis in short-term (3-6 months)

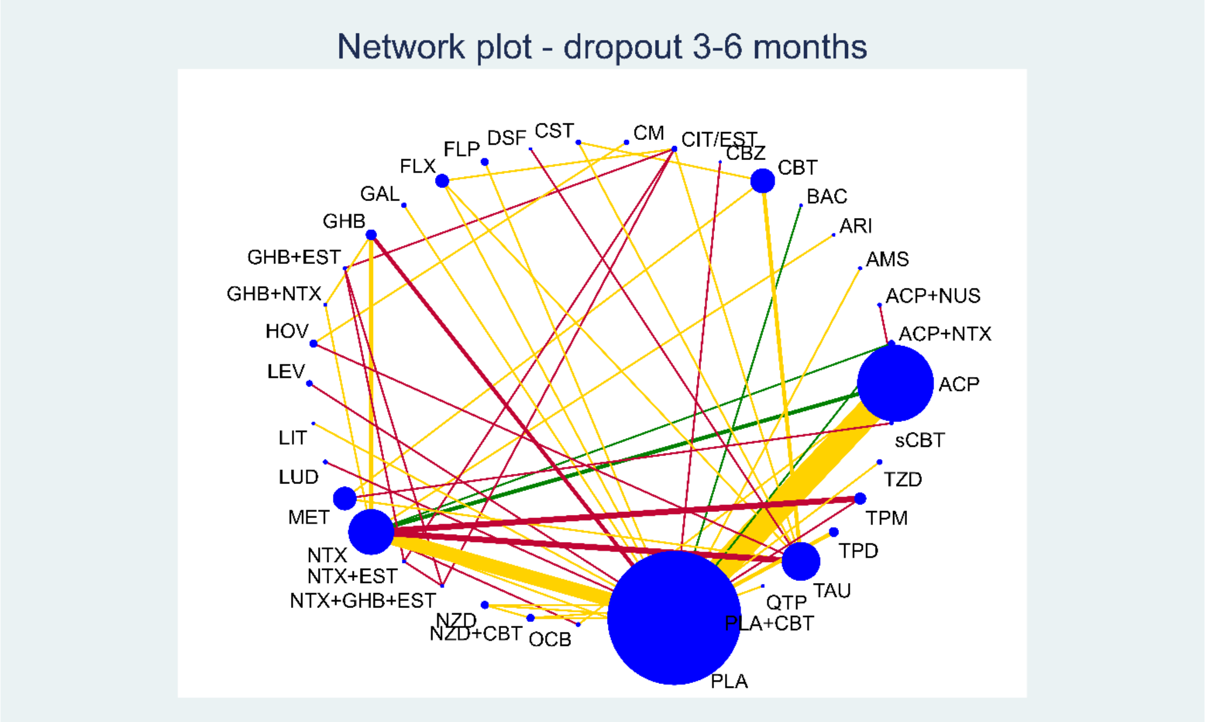

Figure S5. Interval plot of dropout analysis in short-term (3-6 months)

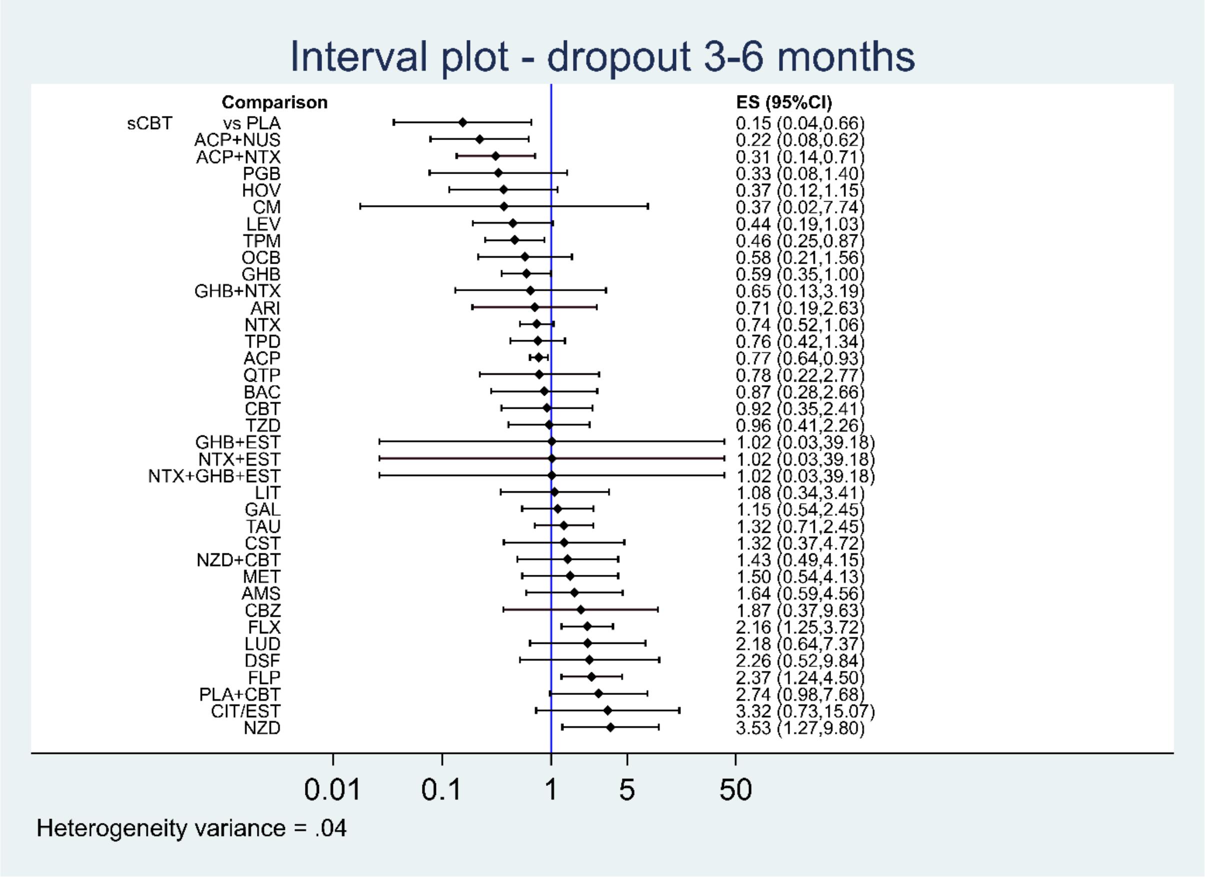

Figure S6. Forest plot of dropout analysis in short-term (3-6 months)

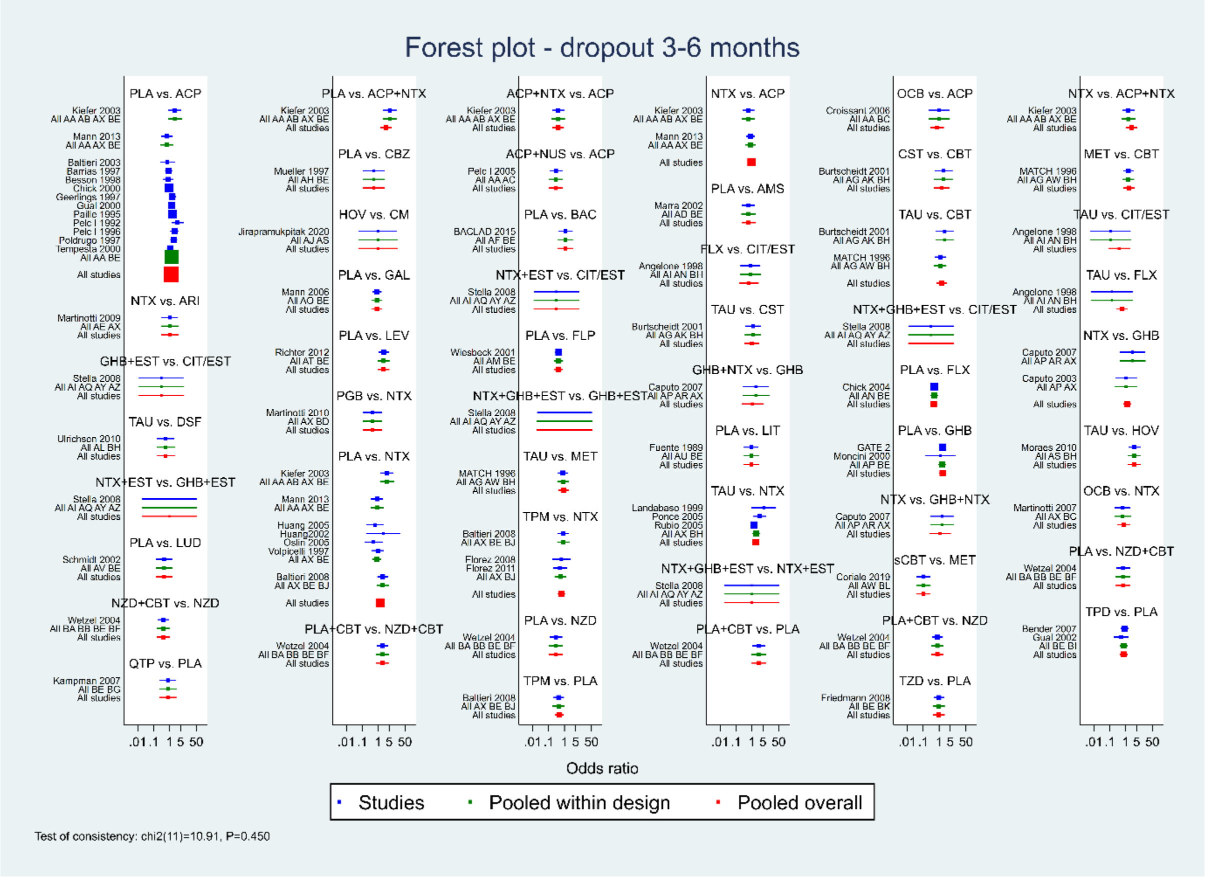

MEDIUM-TERM

Figure S7. Network plot of abstinence analysis in medium-term (6-12 months) (Subset 1)

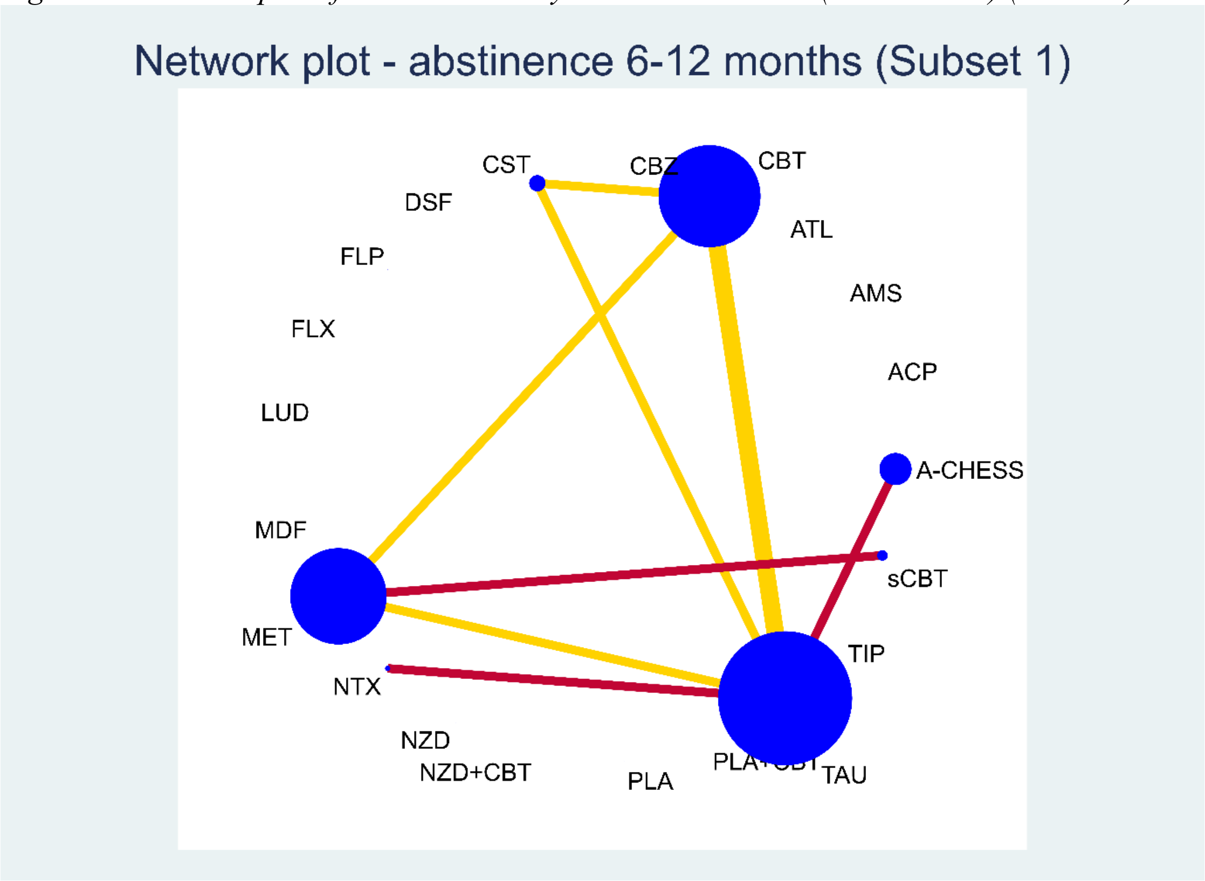

Figure S8. Interval plot of abstinence analysis in medium-term (6-12 months) (Subset 1)

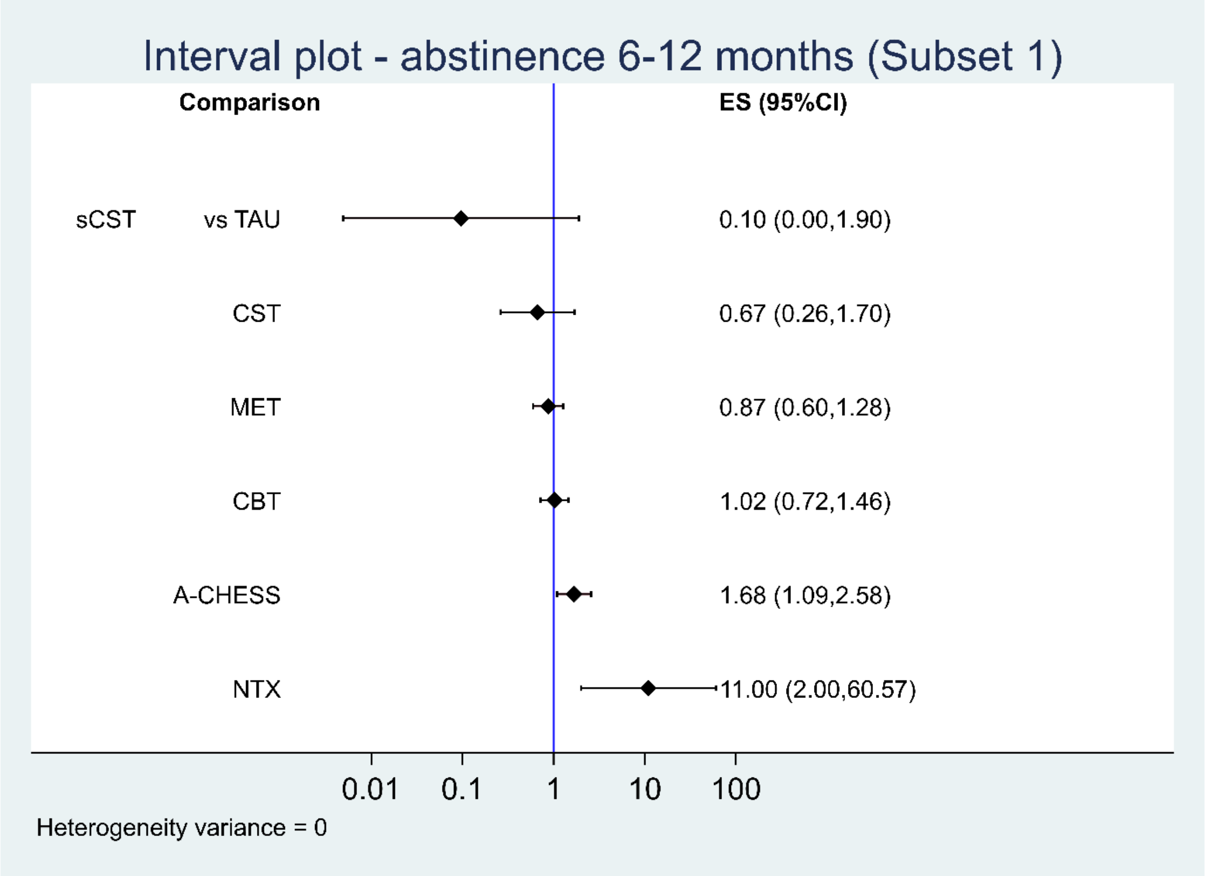

Figure S9. Forest plot for abstinence analysis in medium-term (6-12 months) (Subset 1)

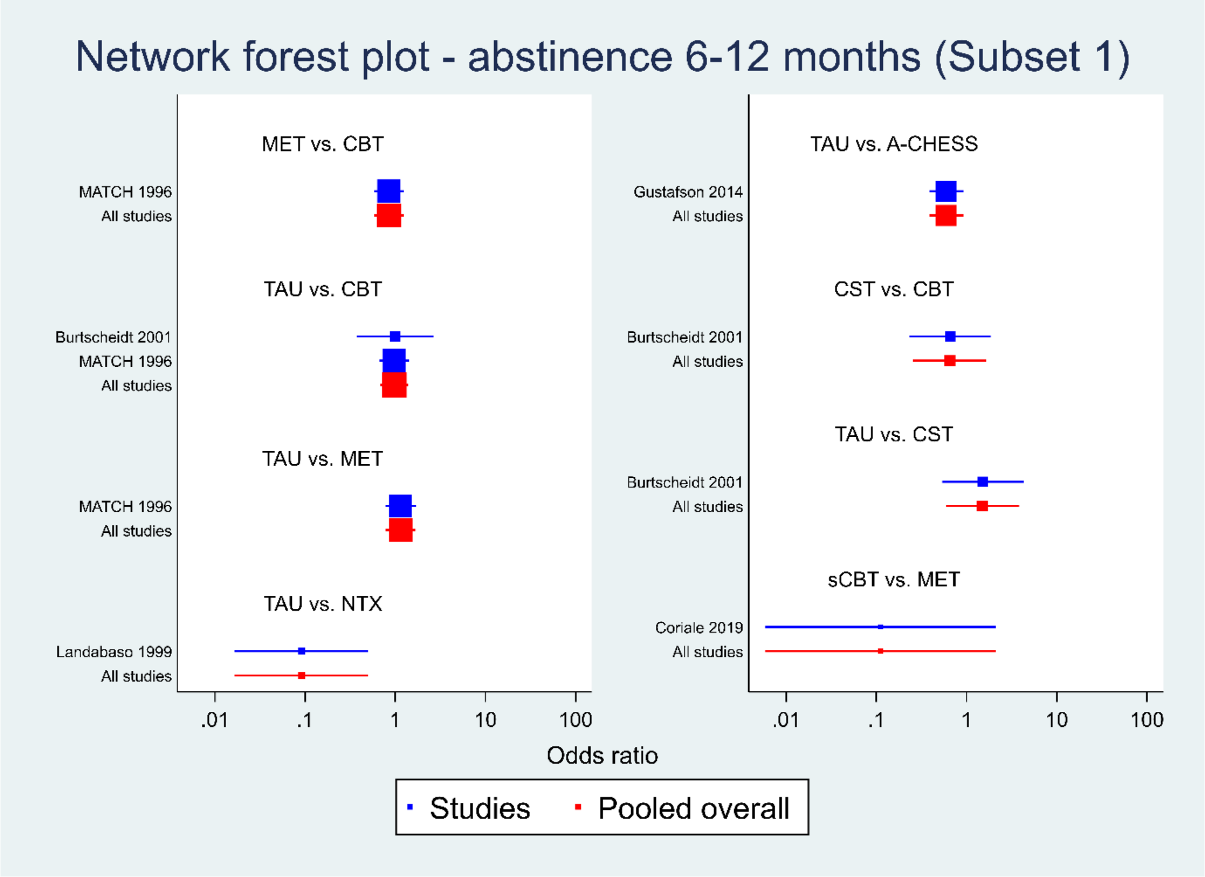

Figure S10. Network plot for abstinence analysis in medium-term (6-12 months) (Subset 2)

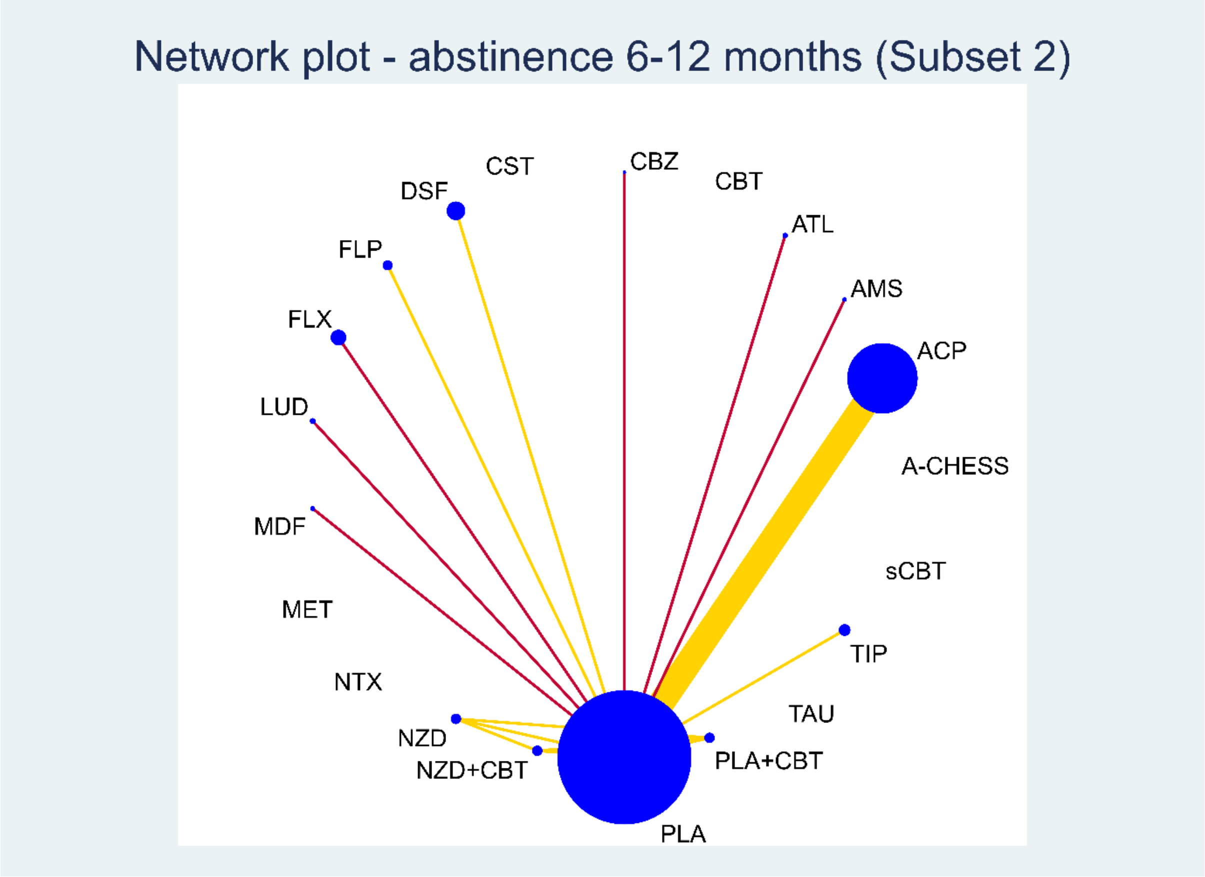

Figure S11. Interval plot for abstinence analysis in medium-term (6-12 months) (Subset 2)

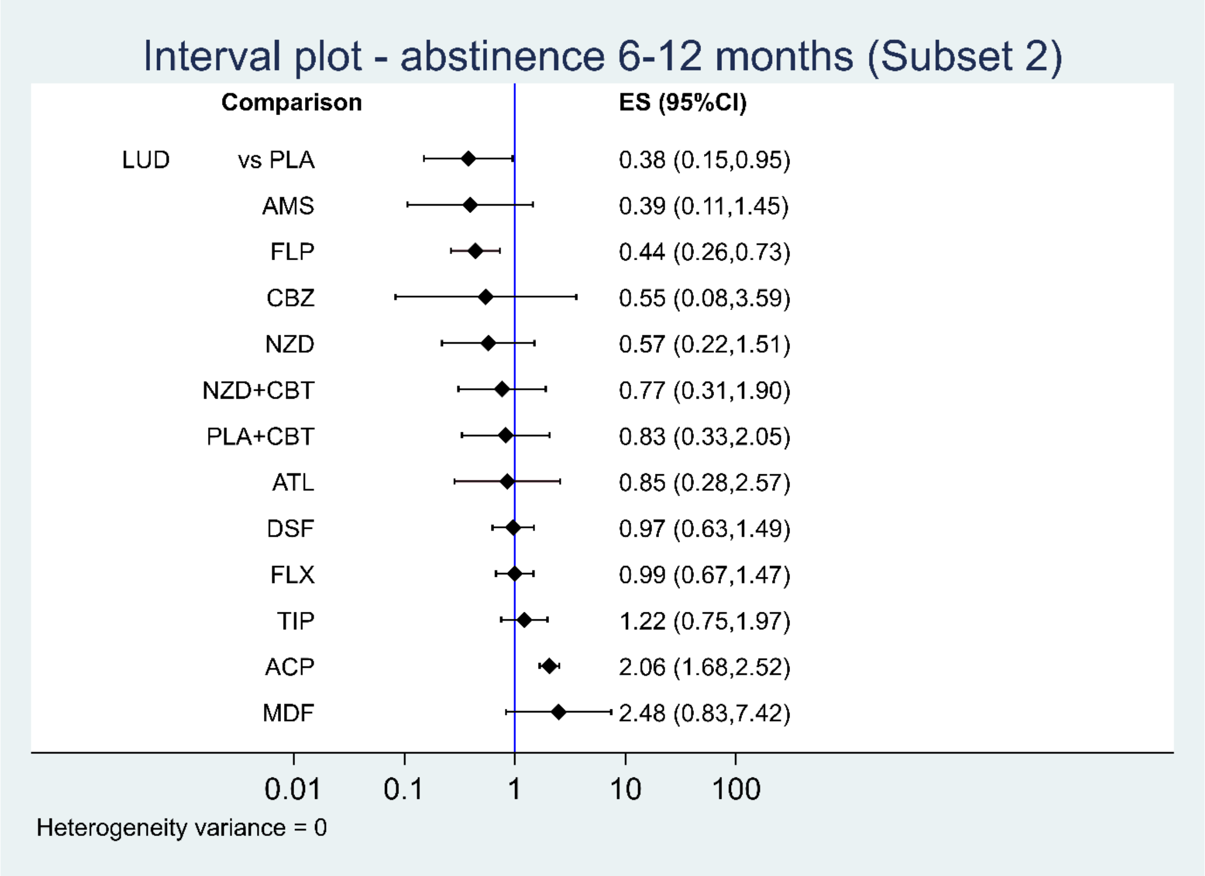

Figure S12. Forest plot for abstinence analysis in medium-term (Subset 2)

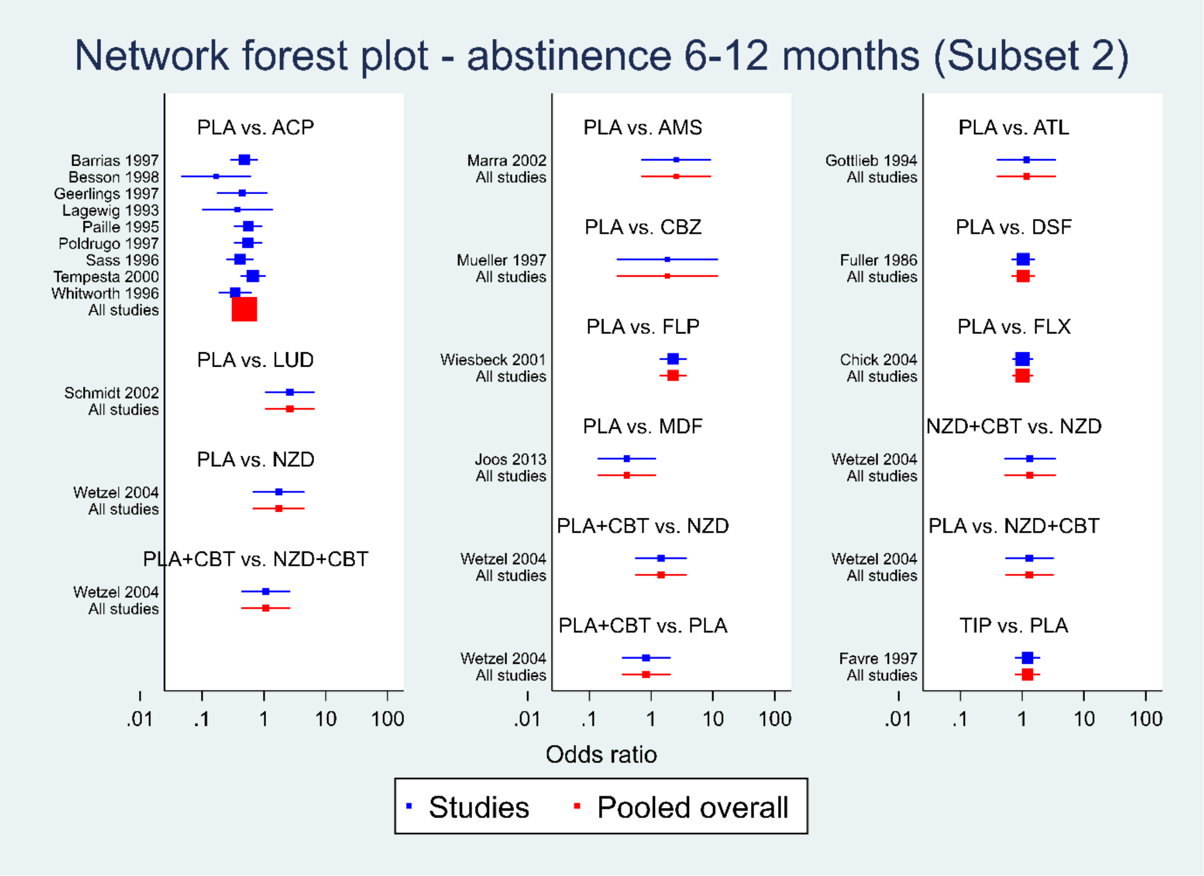

Figure S13. Network plot of dropout analysis in medium-term (6-12 months) (Subset 1)

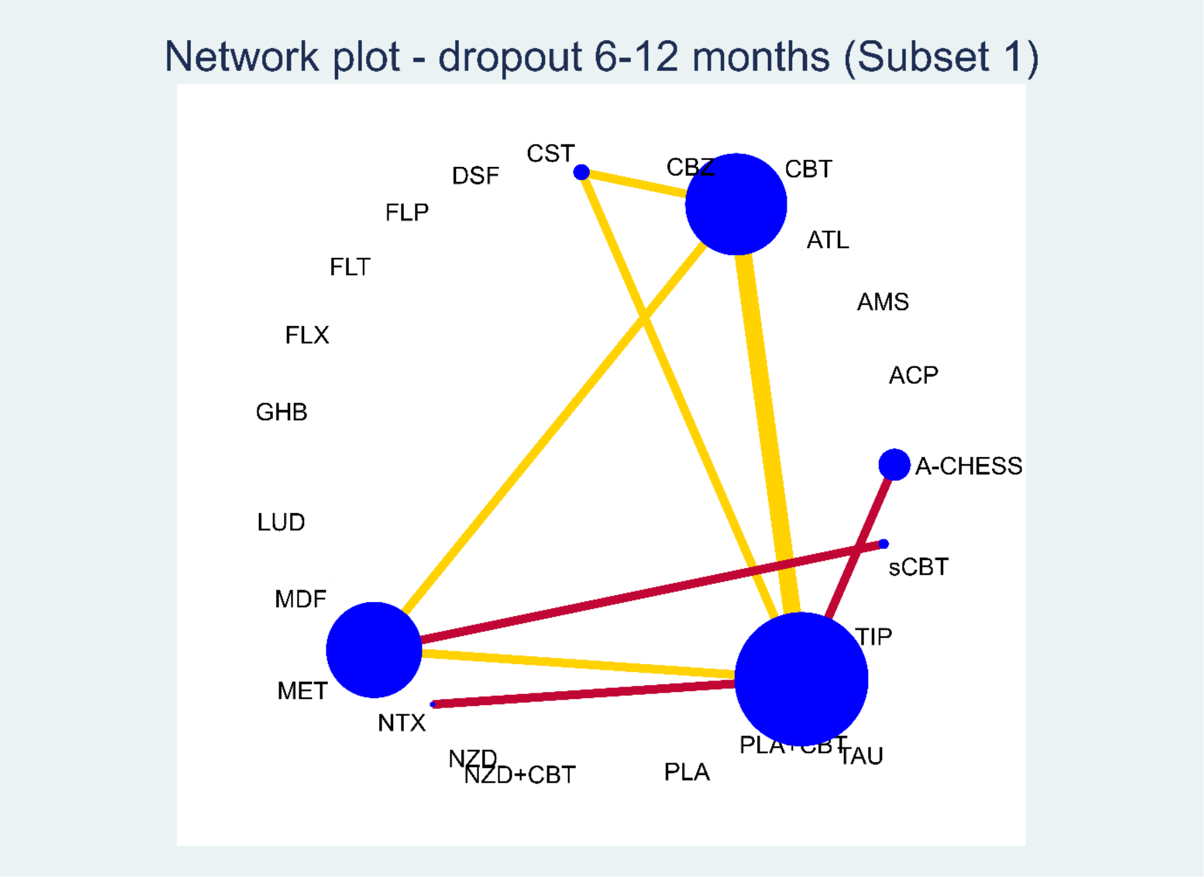

Figure S14. Interval plot of dropout analysis in medium-term (6-12 months) (Subset 1)

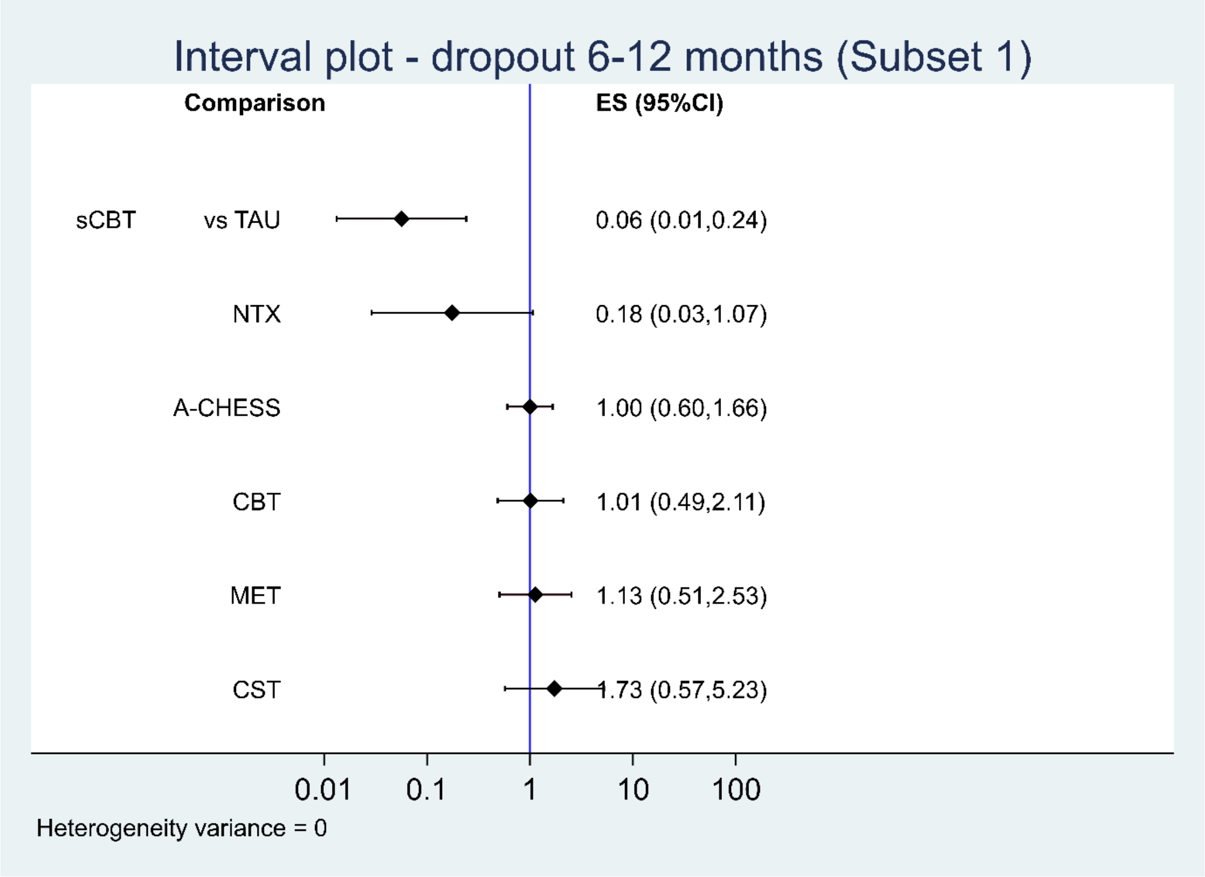

Figure S15. Forest plot of dropout analysis in medium-term (6-12 months) (Subset 1)

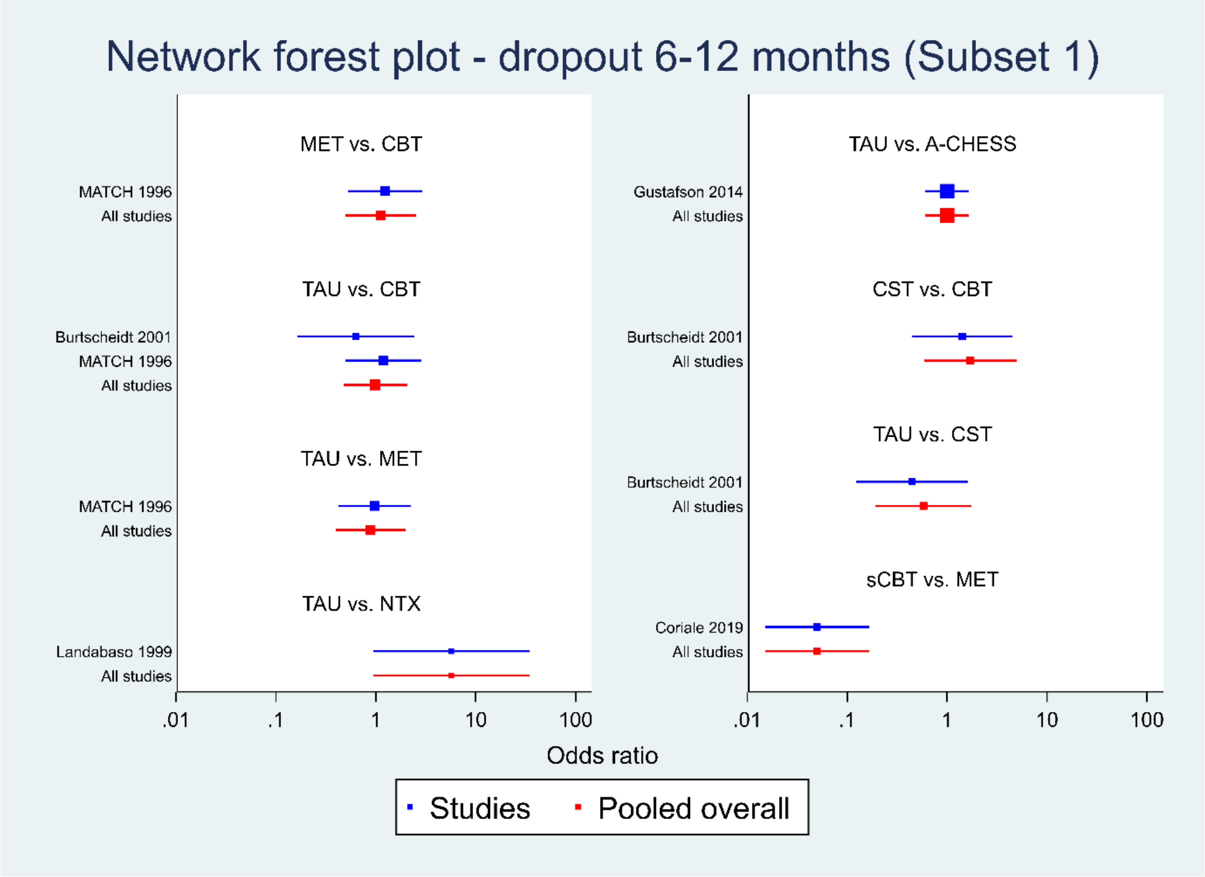

Figure S16. Network plot of dropout analysis in medium-term (6-12 months) (Subset 2)

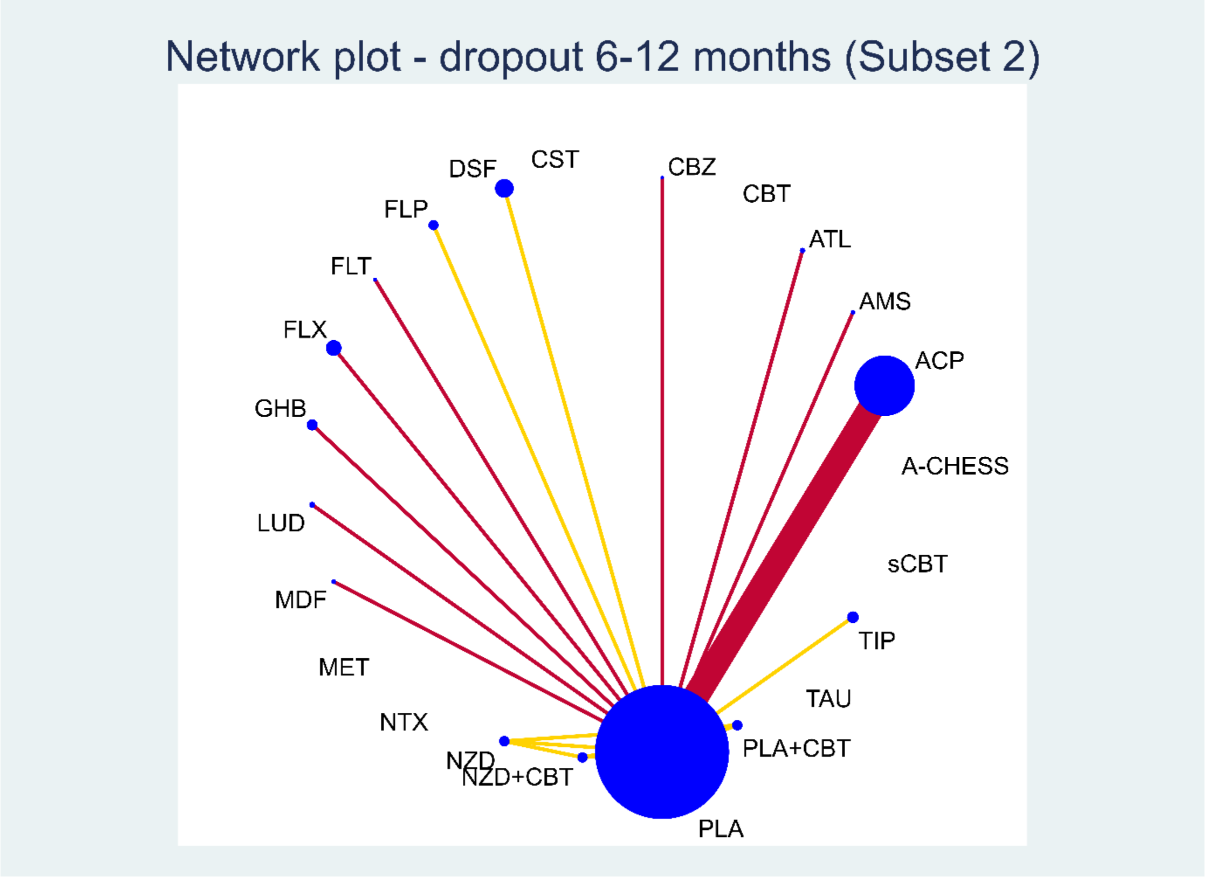

Figure S17. Interval plot of dropout analysis in medium-term (6-12 months) (Subset 2)

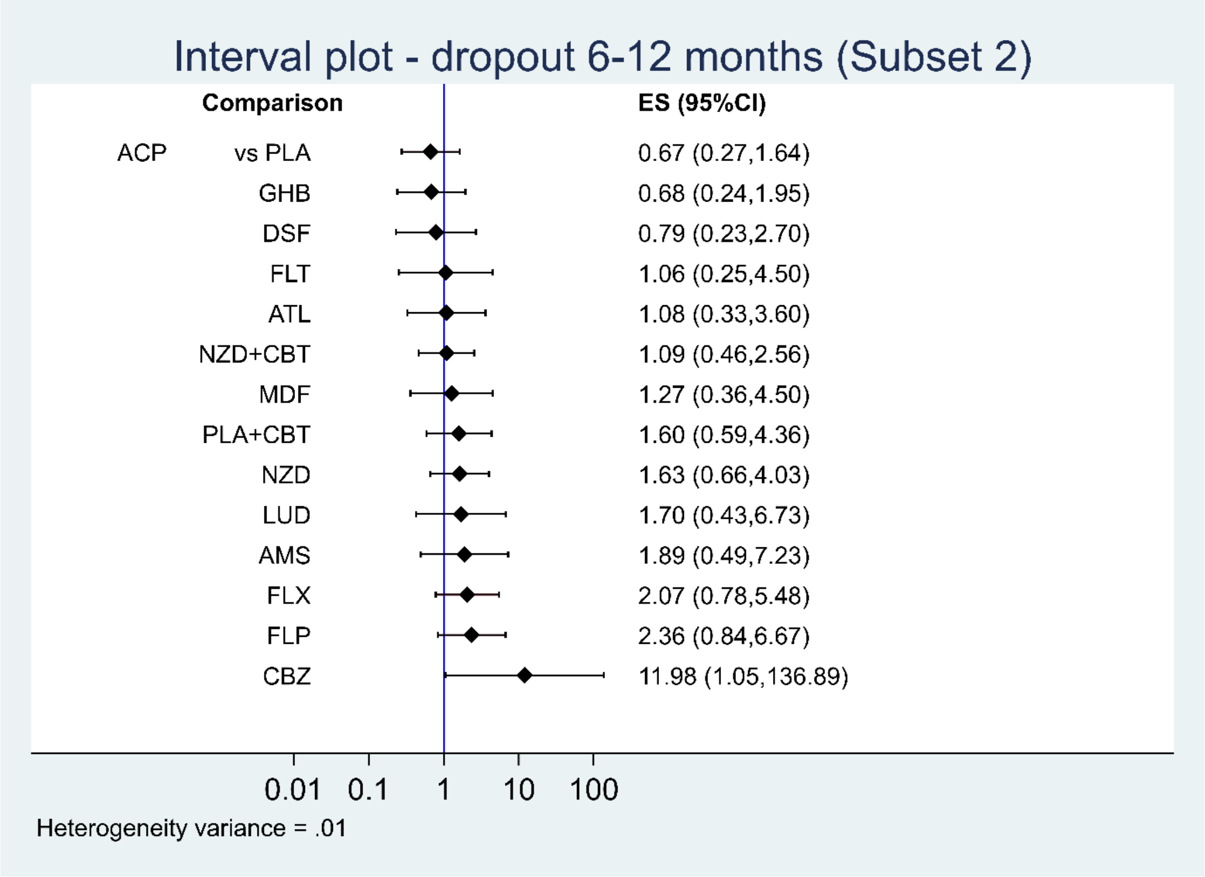

Figure S18. Forest plot of dropout analysis in medium-term (6-12 months) (Subset 2)

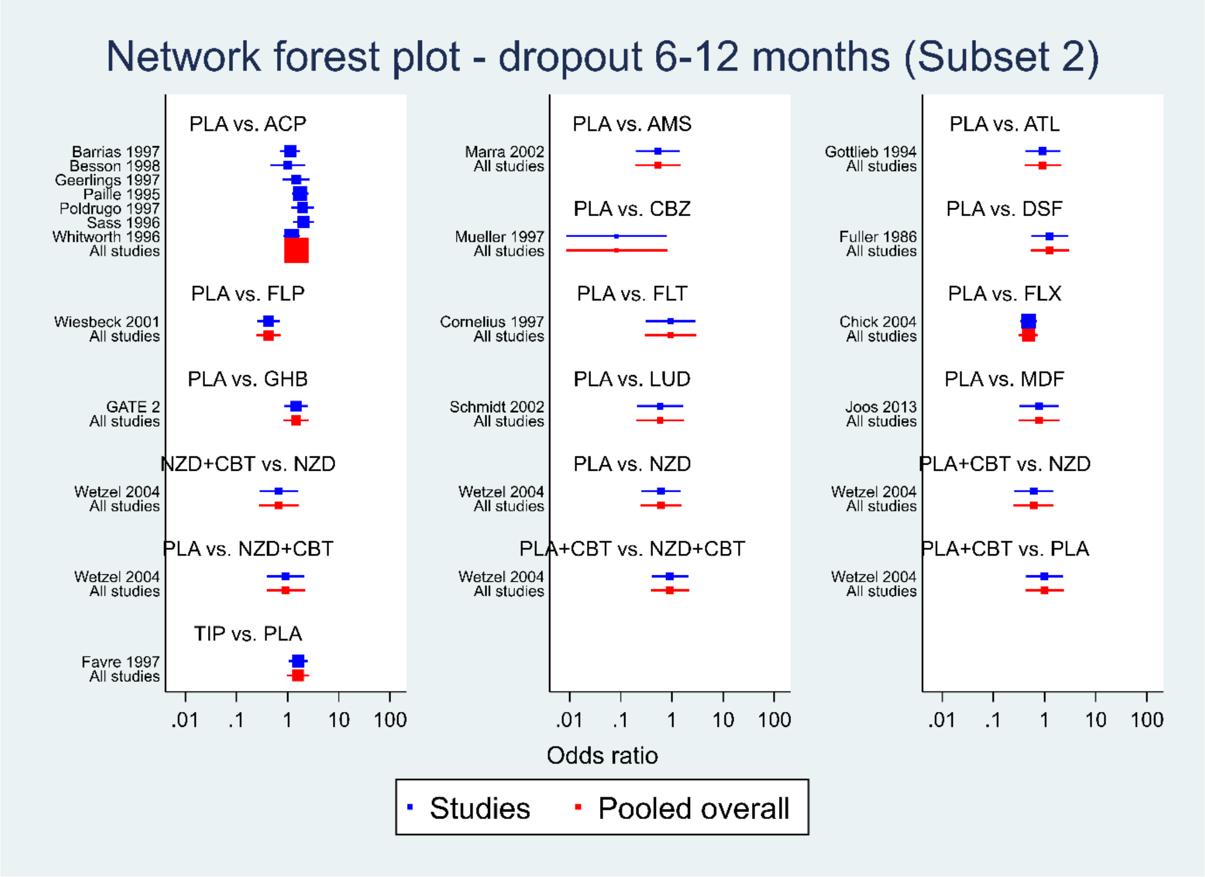

LONG-TERM

Figure S19. Network plot of abstinence analysis in long-term (12-24 months)

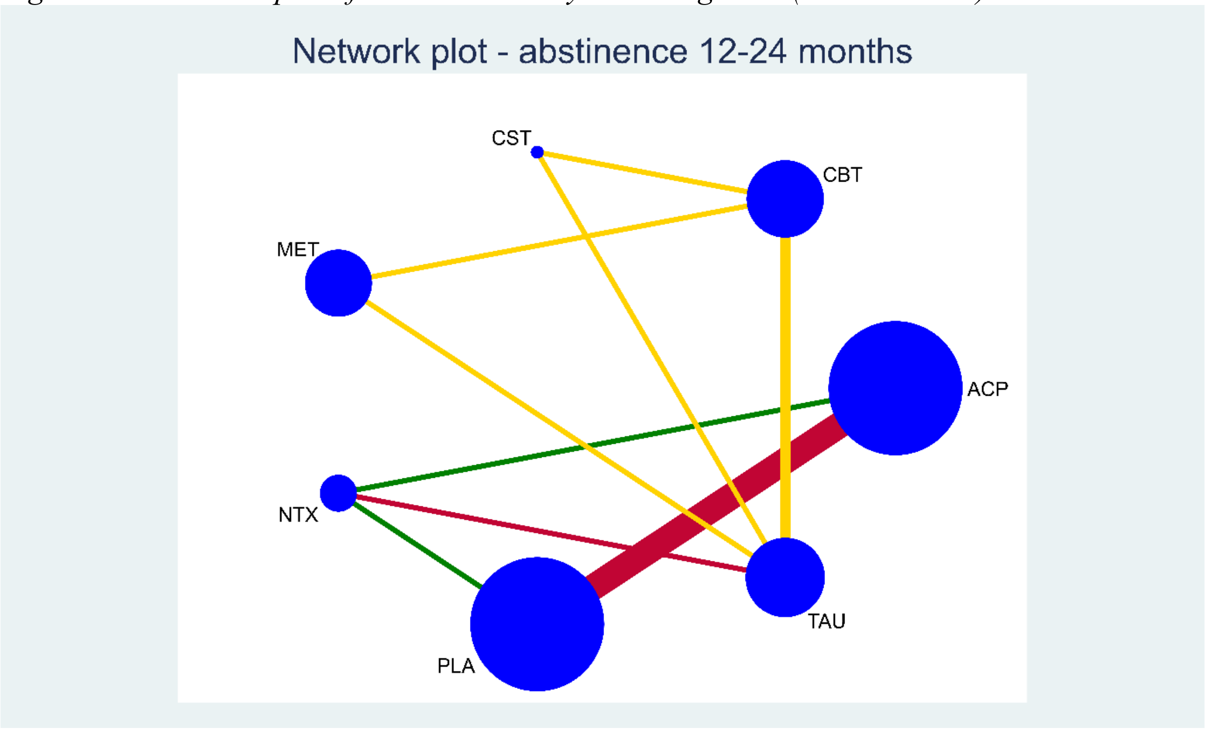

Figure S20. Interval plot of abstinence analysis in long-term (12-24 months)

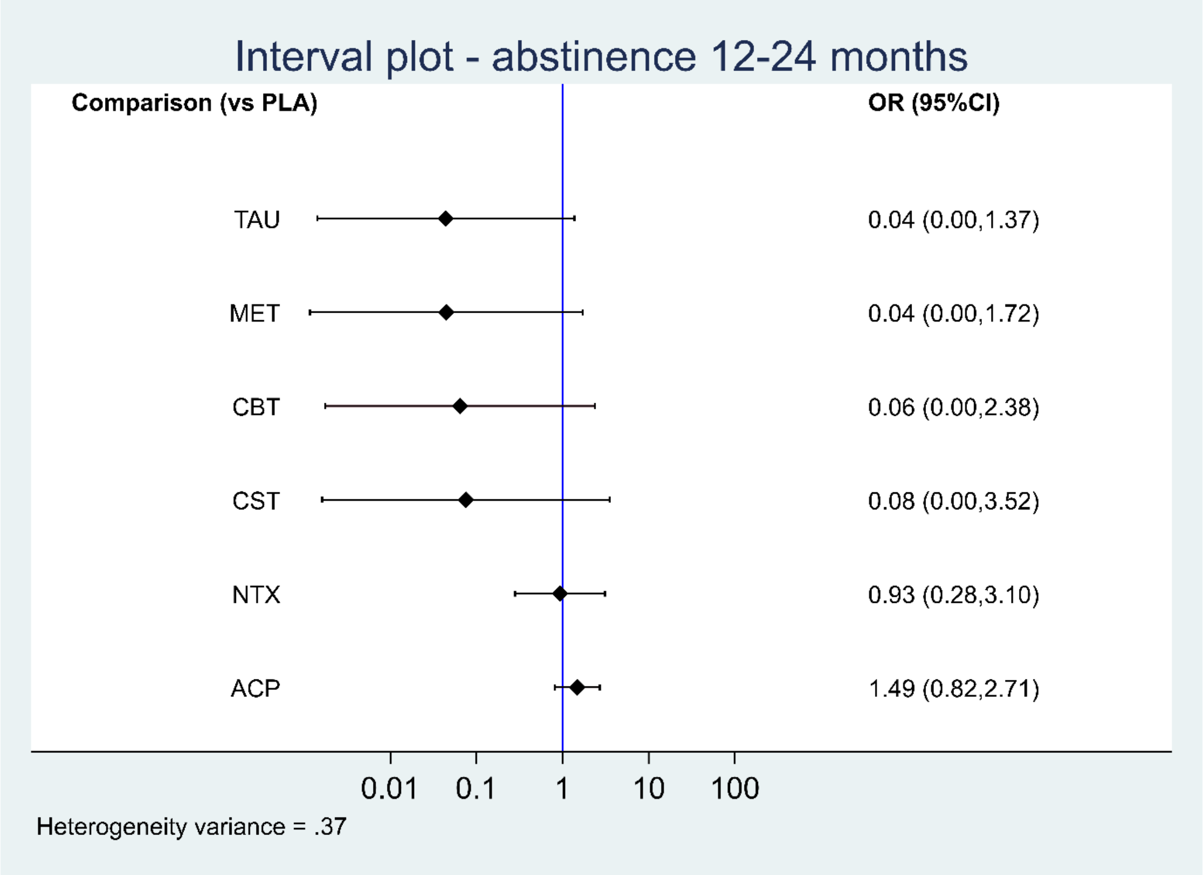

Figure S21. Forest plot of abstinence analysis in long-term (12-24 months)

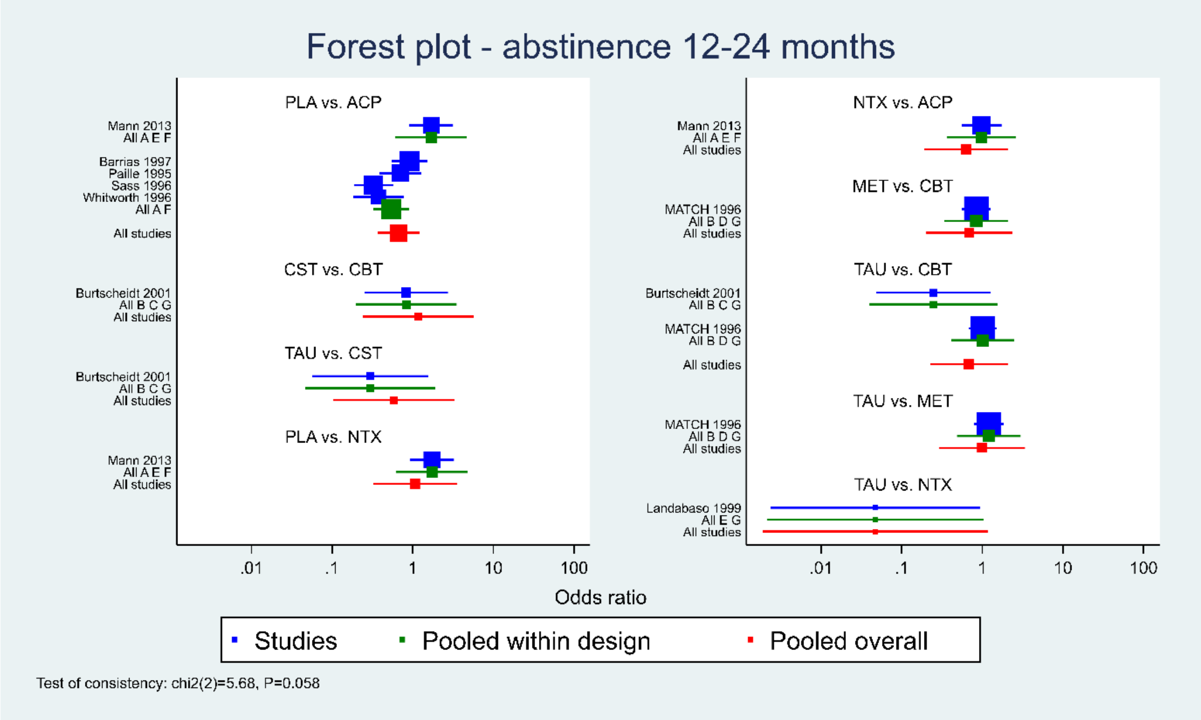

Figure S22. Network plot of dropout analysis in long-term (12-24 months)

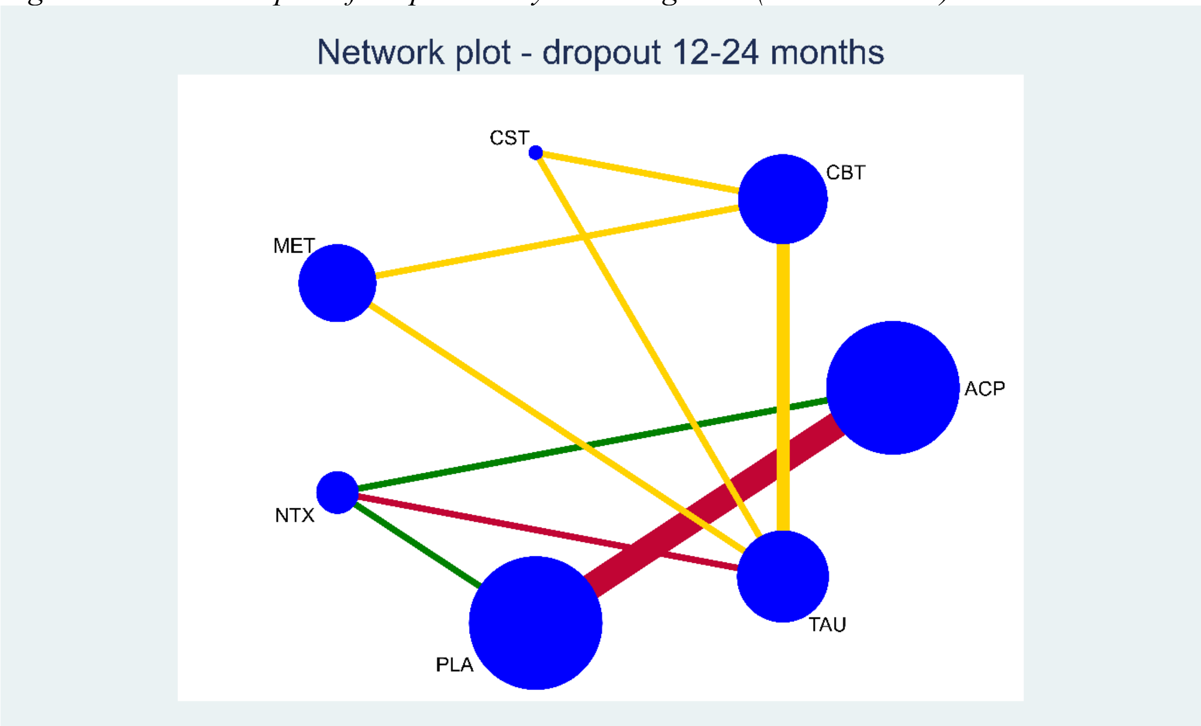

Figure S23. Interval plot of dropout analysis in long-term (12-24 months)

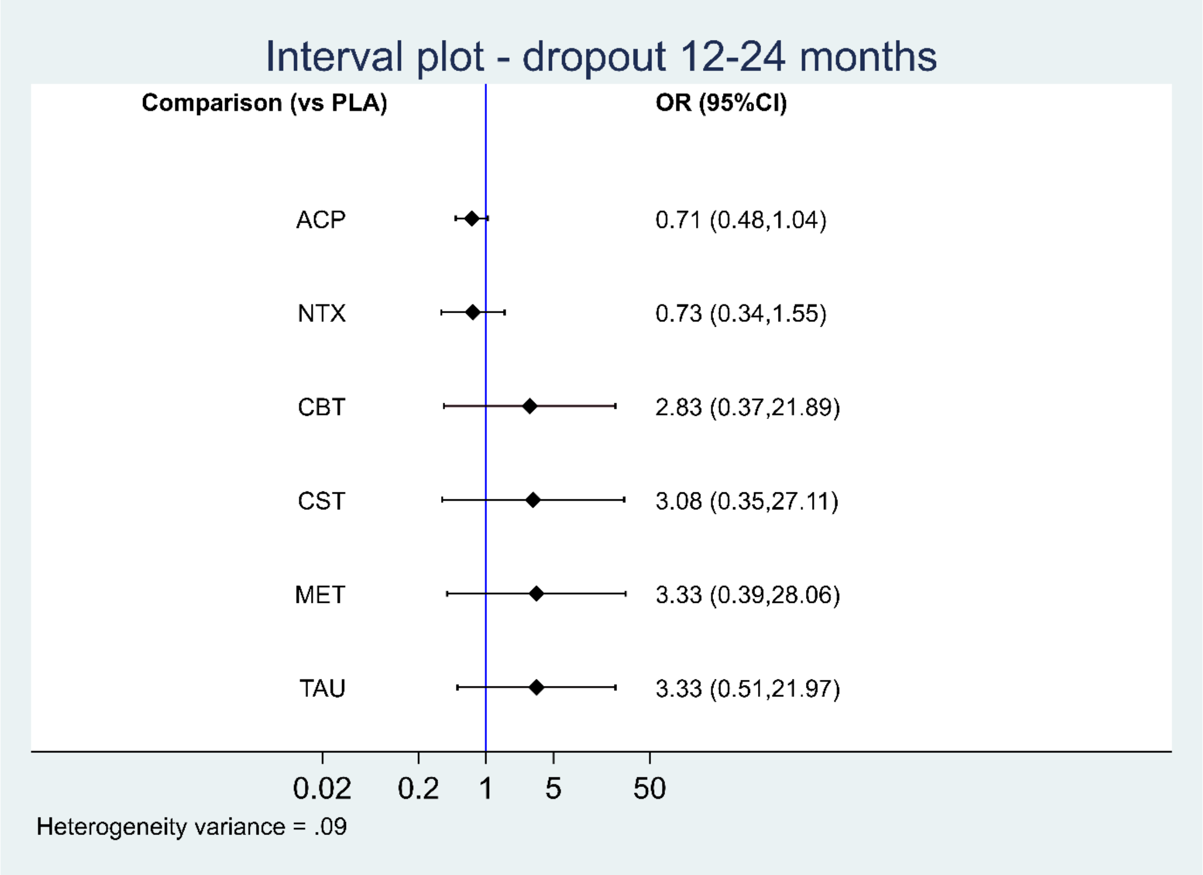

Figure S24. Forest plot of dropout analysis in long-term (12-24 months)

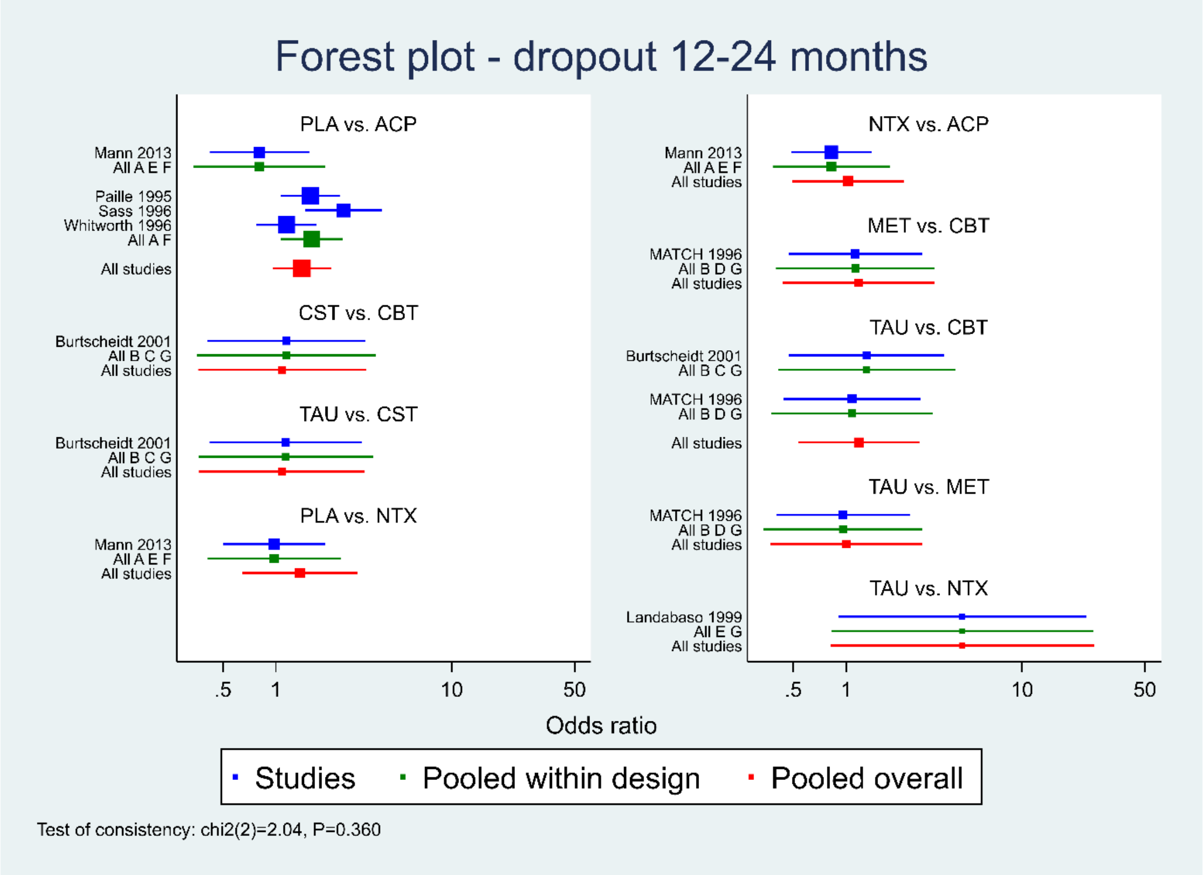

STUDIES WITH PSYCHOTHERAPY ONLY

Figure S25. Network plot of abstinence analysis up to 12 months

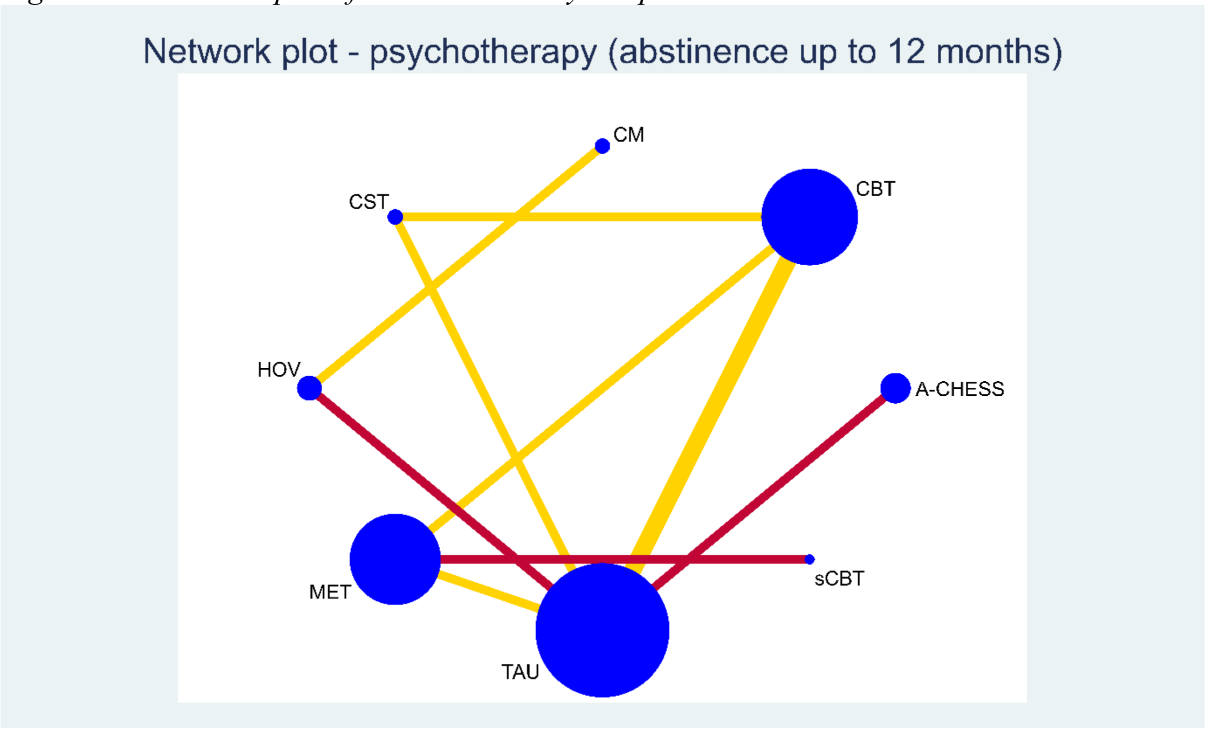

Figure S26. Interval plot of abstinence analysis up to 12 months

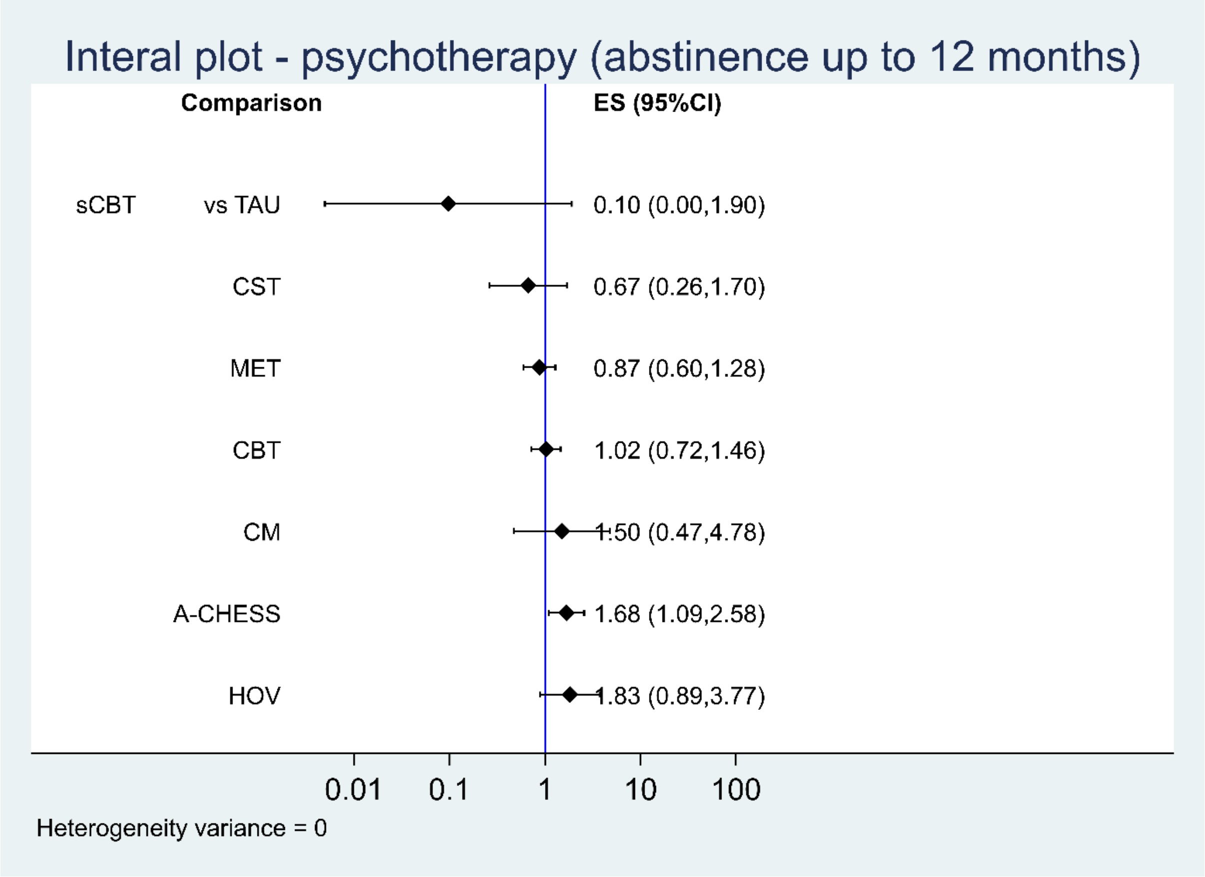

Figure S27. Forest plot of abstinence analysis up to 12 months

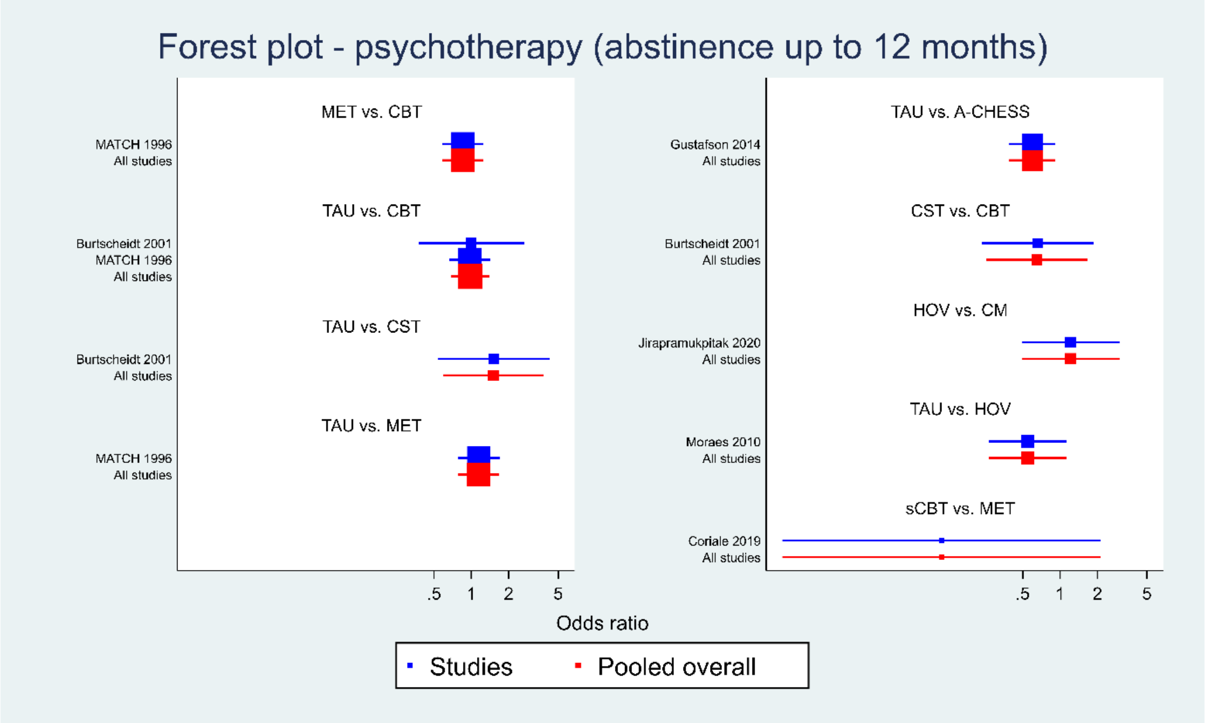

Figure S28. Network plot of dropout analysis up to 12 months

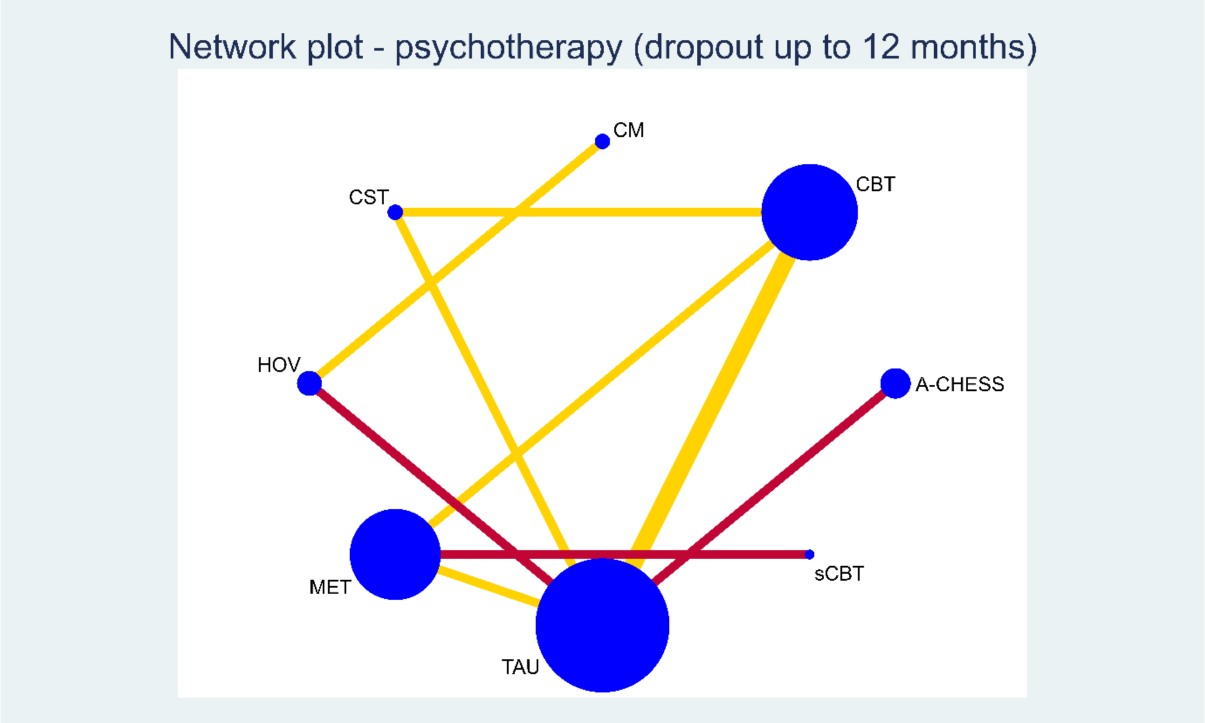

Figure S29. Interval plot of dropout analysis up to 12 months

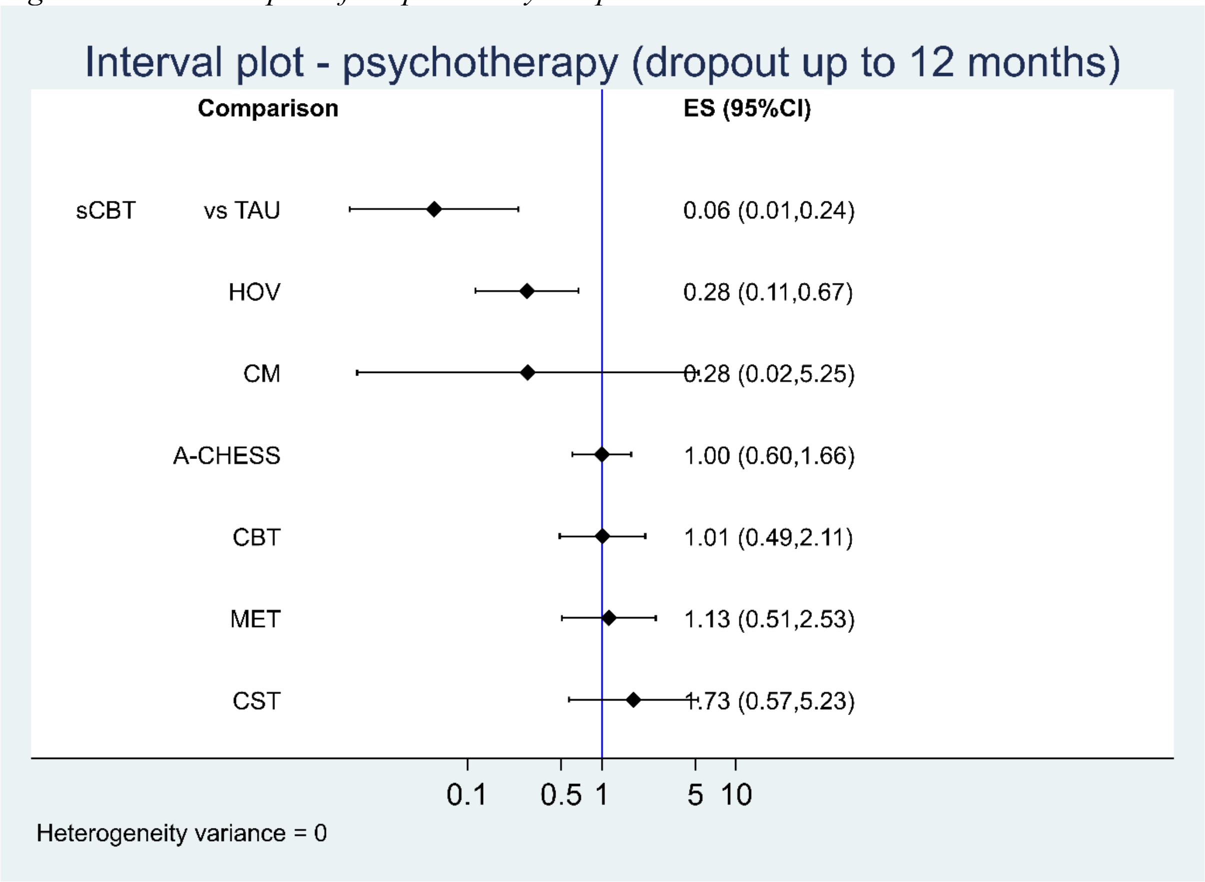

Figure S30. Forest plot of dropout analysis up to 12 months

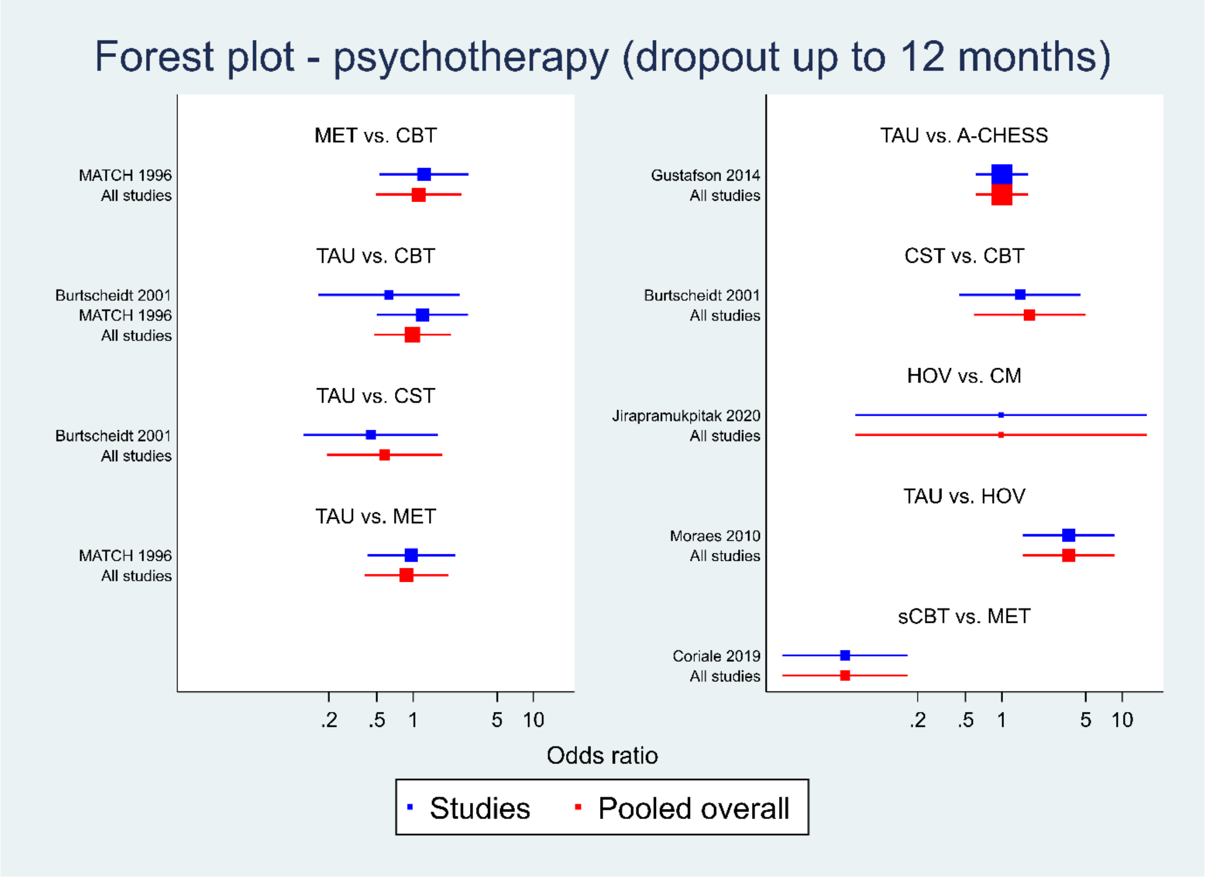

STUDIES WITH PHARMACOTHERAPY

Figure S31. Network plot of abstinence analysis up to 12 months

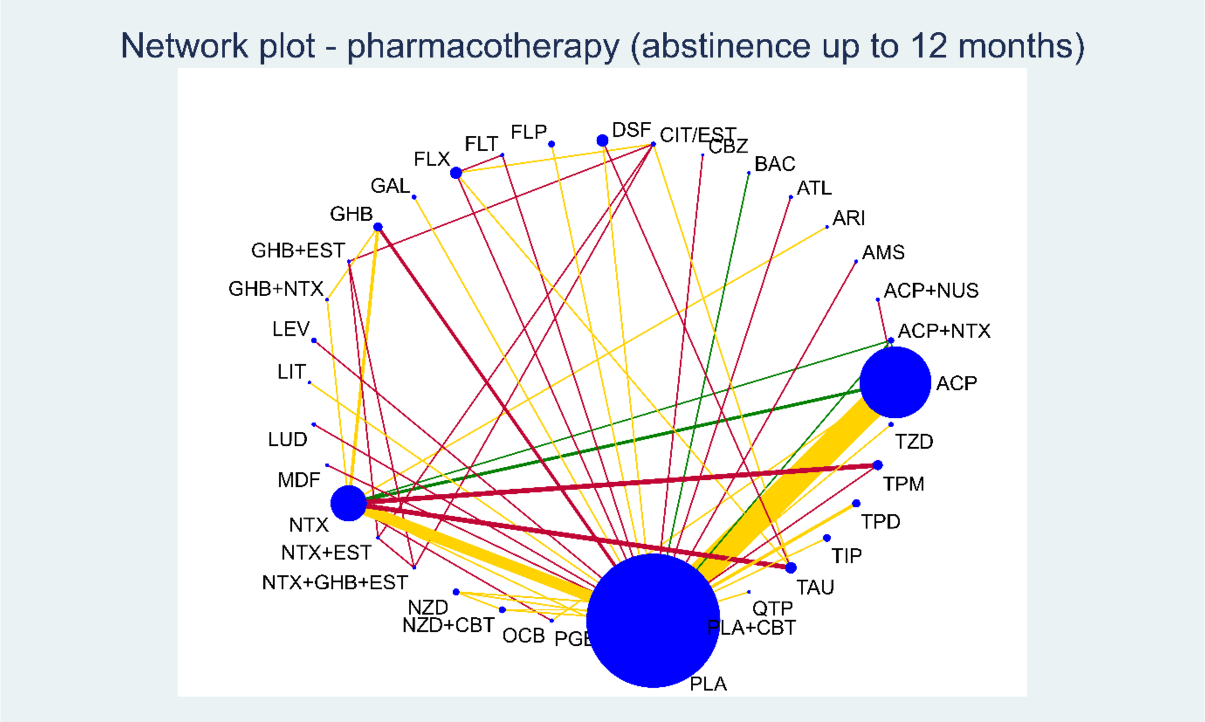

Figure S32. Interval plot of abstinence analysis up to 12 months

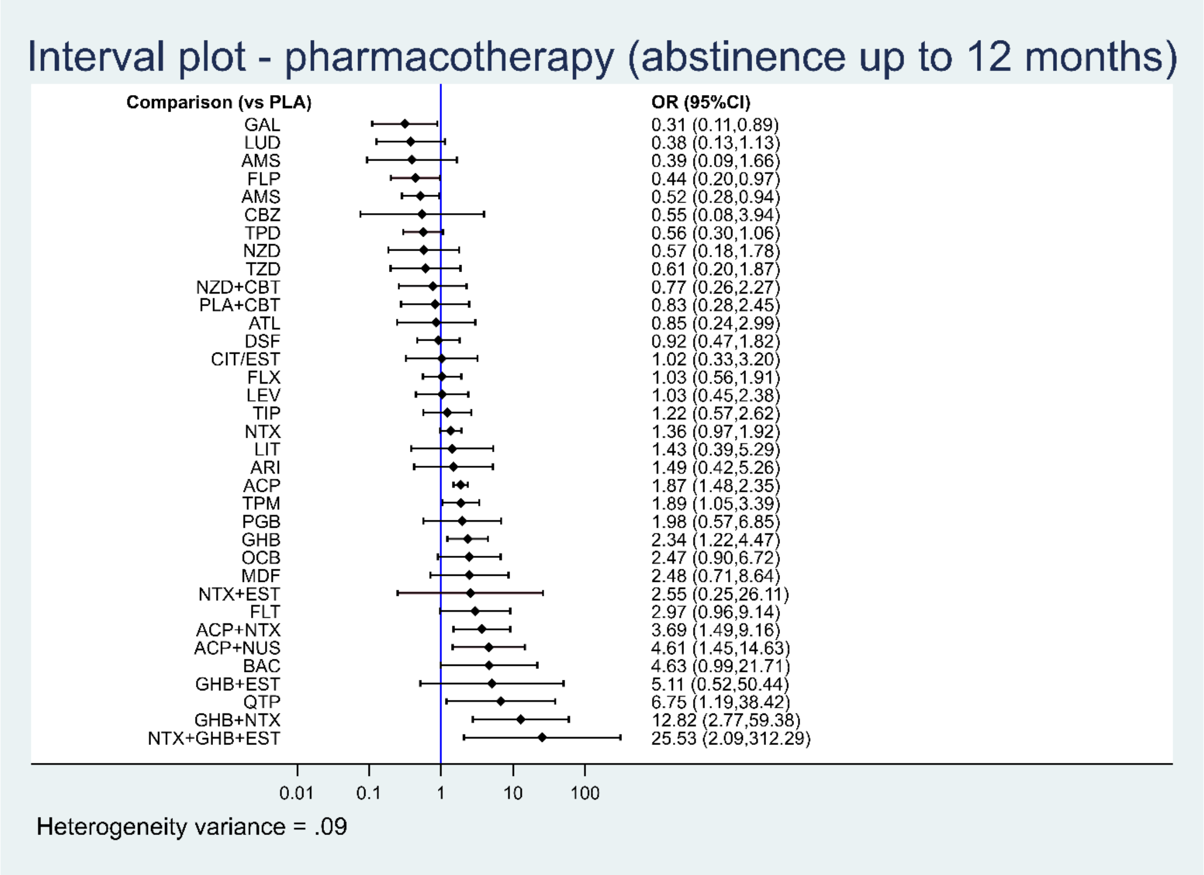

Figure S33. Forest plot of abstinence analysis up to 12 months

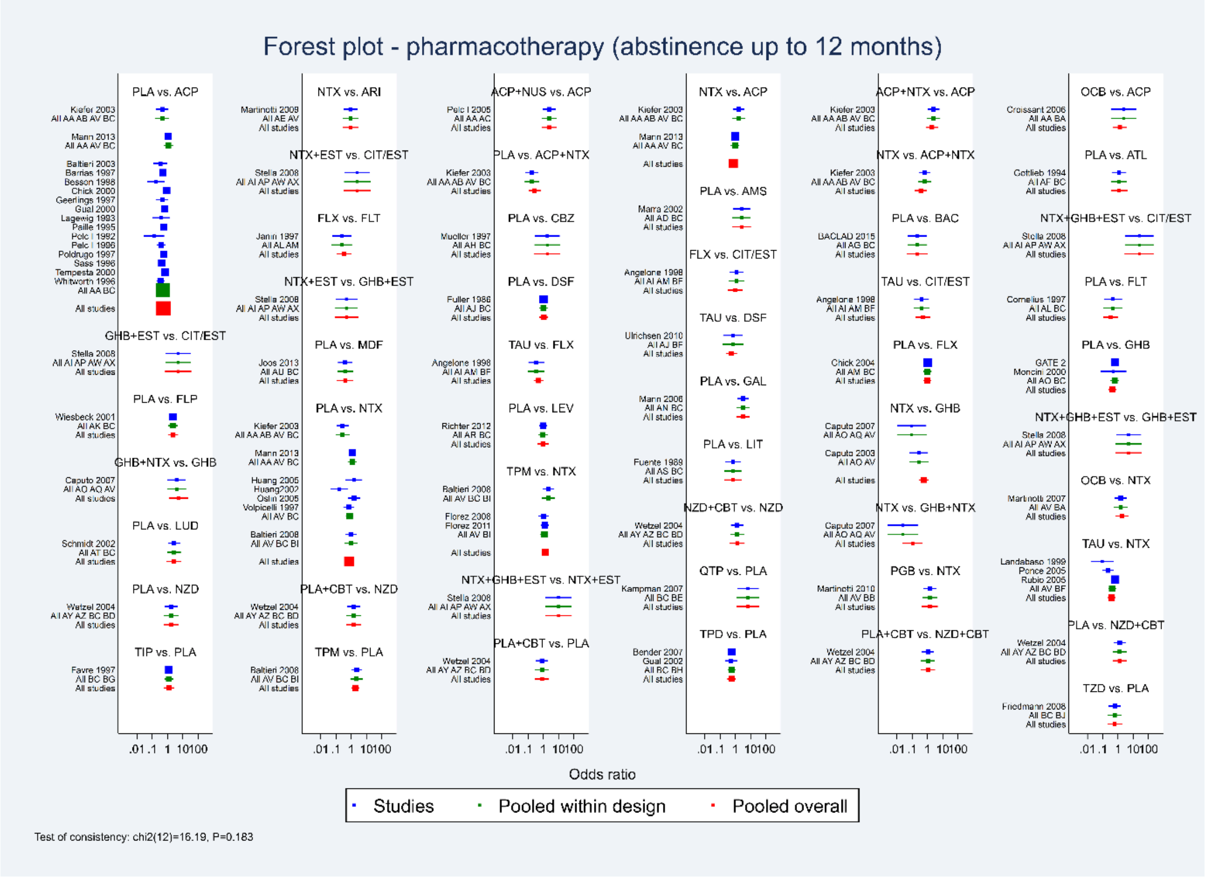

Figure S34. Network plot of dropout analysis up to 12 months

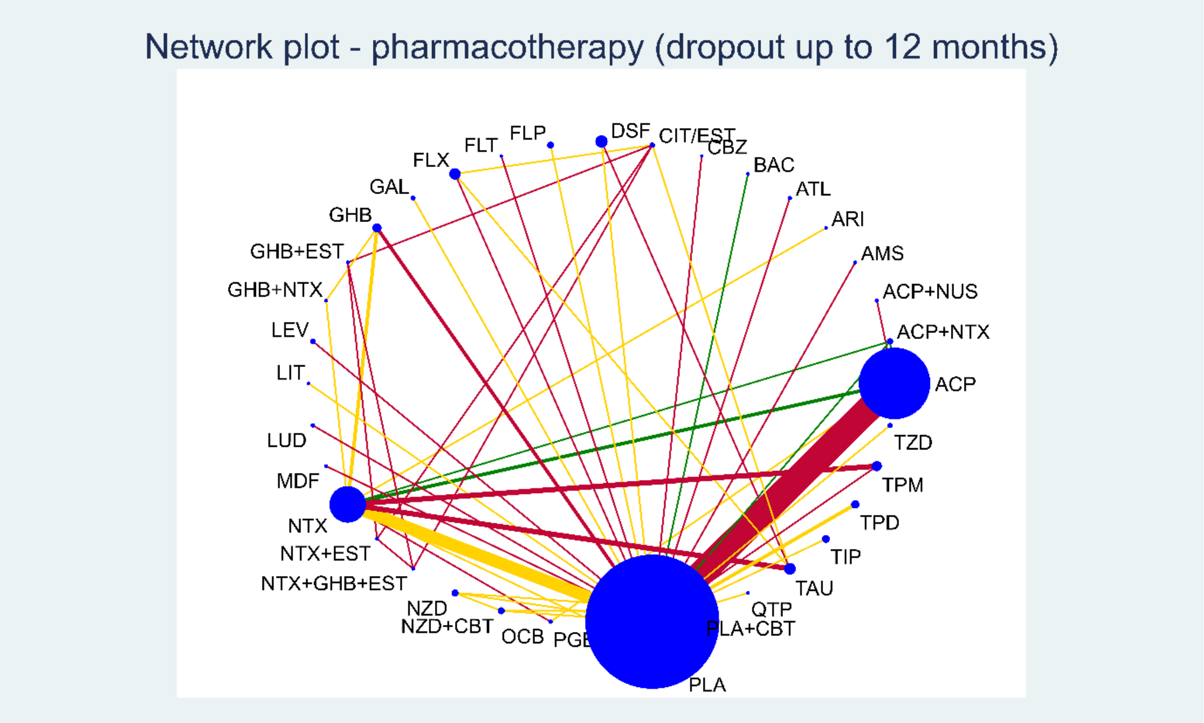

Figure S35. Interval plot of dropout analysis up to 12 months

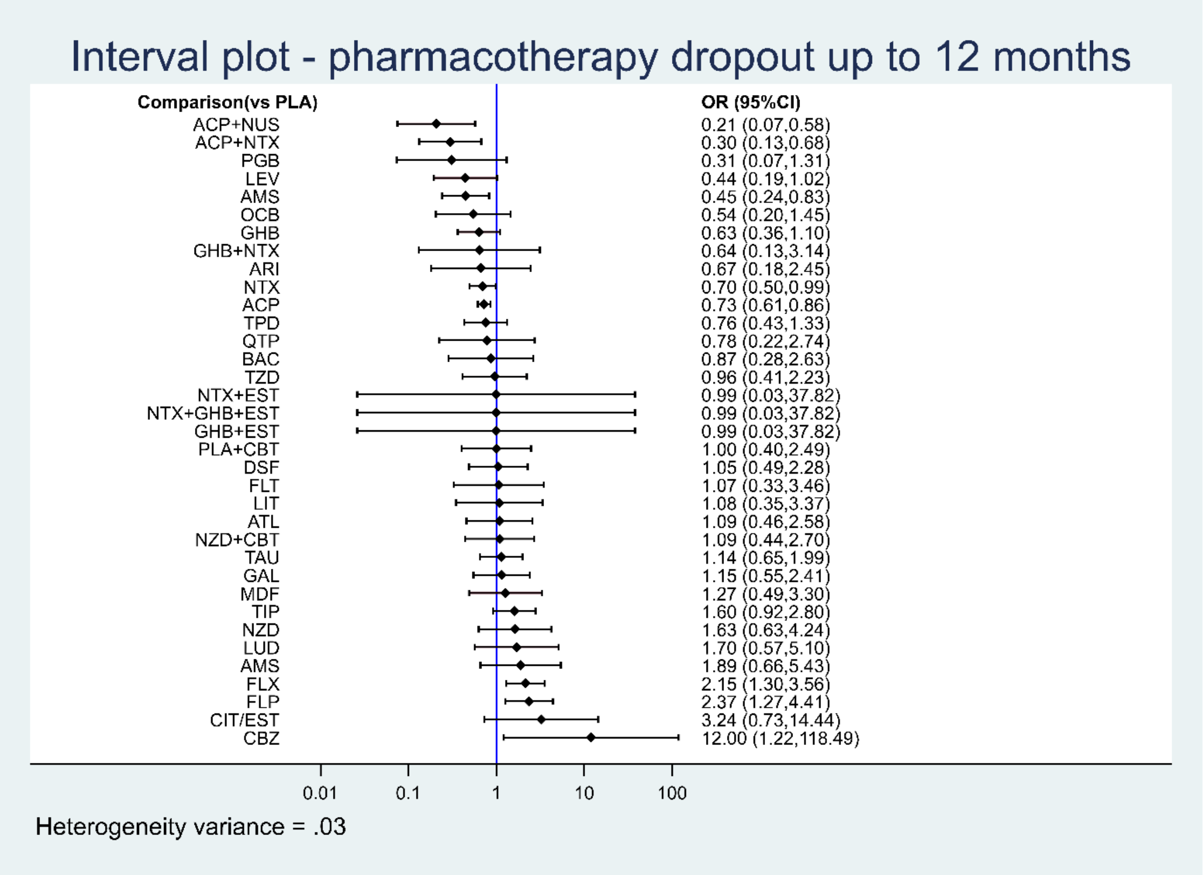

Figure S36. Forest plot of dropout analysis up to 12 months

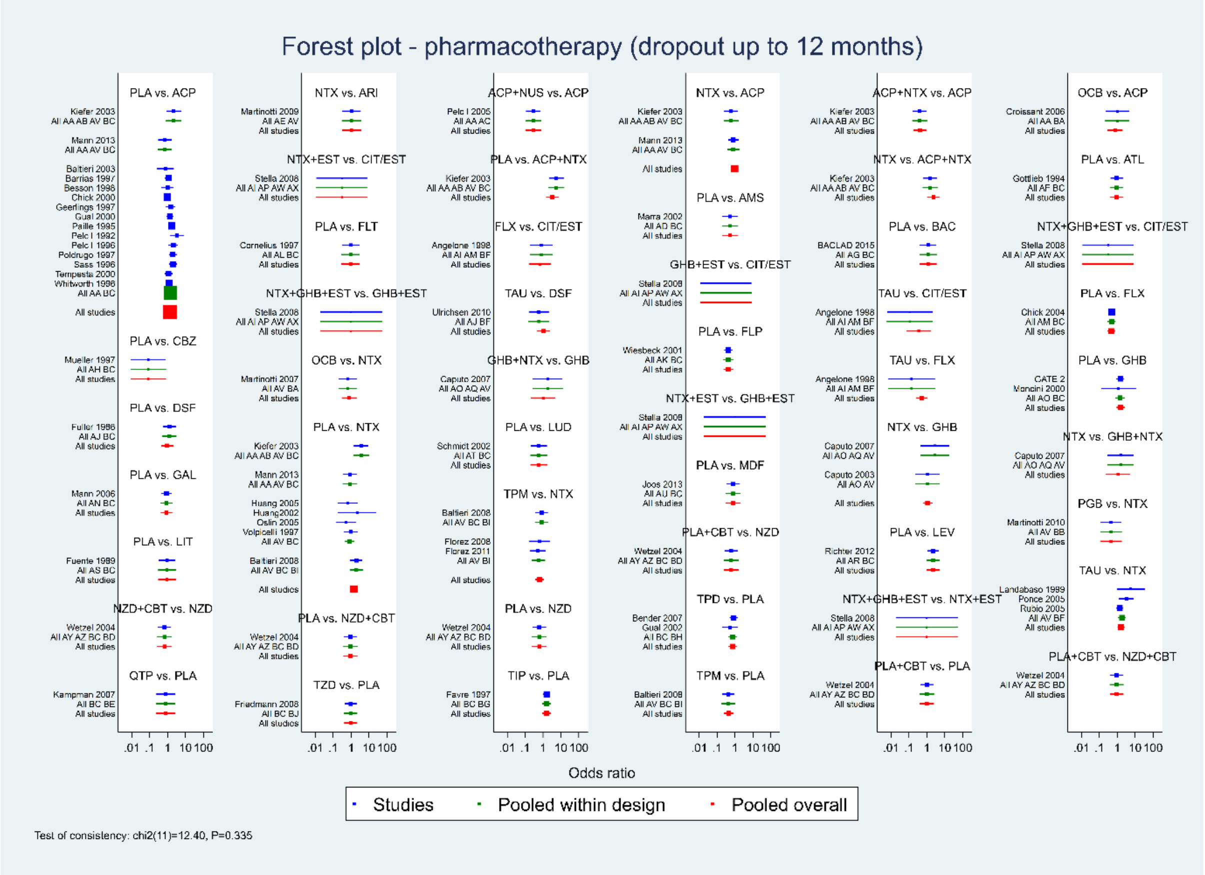

Supplement: Supplementary file 1 — Supplementary information: additional tables and figures supplied by authors [file chev054398.ww.pdf]
